# Olefination of Alkyl Halides with Aldehydes by Merging Visible-Light Photoredox Catalysis and Organophosphorus Chemistry

**DOI:** 10.1016/j.isci.2018.07.011

**Published:** 2018-07-20

**Authors:** Min Jiang, Haijun Yang, Quentin Lefebvre, Jihu Su, Hua Fu

**Affiliations:** 1 Key Laboratory of Bioorganic Phosphorus Chemistry and Chemical Biology (Ministry of Education), Department of Chemistry, Tsinghua University, Beijing 100084, China; 2 School of Chemistry, University of Bristol, Cantock's Close, Bristol BS8 1TS, UK; 3 CAS Key Laboratory of Microscale Magnetic Resonance, Department of Modern Physics, University of Science and Technology of China, Hefei 230026, China

**Keywords:** Chemistry, Catalysis, Organic Chemistry

## Abstract

Carbon-carbon double bond (C=C) formation is a crucial transformation in organic chemistry. Visible-light photoredox catalysis provides economical and sustainable opportunities for the development of novel and peculiar organic reactions. Here we report a method for the olefination of alkyl halides with aldehydes by visible-light photoredox catalysis using triphenylphosphine as a reductive quencher (103 examples). This transformation accommodates a variety of aldehydes including paraformaldehyde; aqueous formaldehyde; 2,2,2-trifluoroacetaldehyde monohydrate; 2,2,2-trifluoro-1-methoxyethanol; and other common aldehydes. The present method exhibits several advantages, including operational simplicity, mild reaction conditions, wide functional group tolerance, and amenability to gram-scale synthesis. We anticipate that it will be widely used in the synthesis of organic molecules, natural products, biological molecules, and polymers.

## Highlights

• General olefination of benzyl halides and bromoacetamides with aldehydes
• Operational simplicity and mild conditions under visible-light photoredox catalysis
• Wide substrate scope and high efficiency
• Amenability to gram-scale synthesis

## Graphical Abstract

**Figure:**
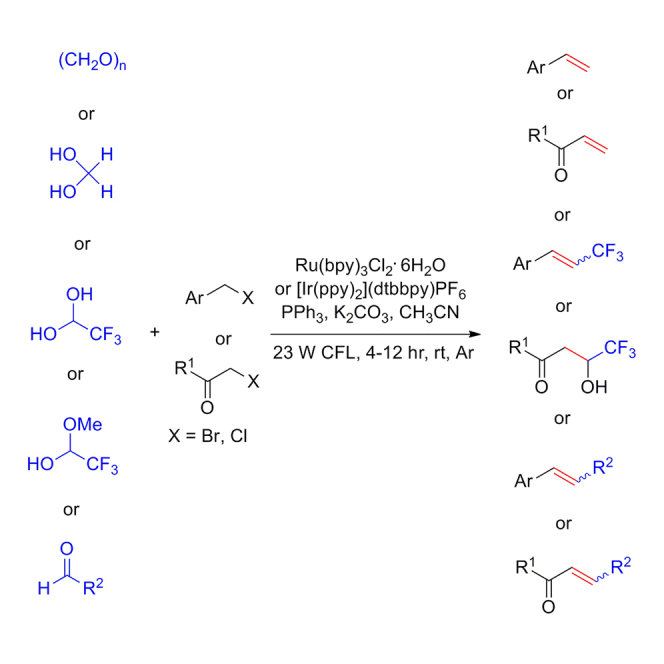

## Introduction

The formation of carbon-carbon double bonds is a key chemical transformation in organic chemistry (Liu et al., 2004, Nicolaou and Sorensen, 1996, Nicolaou and Snyder, 2003, Saklani and Kutty, 2008). Besides direct elimination (Clayden et al., 2001), four routine and reliable methods for the synthesis of alkenes are widely used: the Wittig reaction (Wittig and Geissler, 1953, Wittig and Schollkopf, 1954), the Peterson reaction (Peterson, 1968), the Julia-Lythgoe (Julia and Paris, 1973, Kocienski et al., 1978)/Julia-Kocienski (Baudin et al., 1991, Blakemore et al., 1998) olefination reactions, and alkene metathesis reactions (Calderon et al., 1967, Garber et al., 2000, Love et al., 2002; Murdzek and Schrock, 1987; Nicolaou et al., 2005, Scholl et al., 1999, Schrock, 1999, Schwab et al., 1996). In 1953, Georg Wittig discovered that treating an aldehyde or ketone with a phosphonium ylide gave an alkene (Wittig and Geissler, 1953, Wittig and Schollkopf, 1954). Since then, the Wittig reaction has been extensively used in organic synthesis (Kolodiazhnyi, 1999, Maryanoff and Reitz, 1989, Nicolaou et al., 1997). However, the classical Wittig reaction usually required heating conditions and long reaction times. Recently, photoredox catalysis has become a powerful strategy for the activation of molecules, and some unprecedented reactions have been developed, thanks to the ability of photoredox catalysts to cleanly transform visible light into prominent levels of chemical energy (Hari and König, 2013, Ravelli et al., 2009, Jin and Fu, 2017, König, 2013, Narayanam and Stephenson, 2011, Shaw et al., 2016, Shi and Xia, 2012, Xuan and Xiao, 2012, Yoon et al., 2010, Zeitler, 2009). For the past year, we have indeed developed some valuable visible-light photoredox organic reactions (Gao et al., 2016, Jiang et al., 2016a, Jiang et al., 2016b, Jiang et al., 2016c, Jiang et al., 2017, Jin et al., 2016a, Jin et al., 2016b, Jin et al., 2016c, Jin et al., 2017, Li et al., 2016). Inspired by the robustness and excellent achievements of photoredox catalysis, we hypothesized that a straightforward procedure might be developed to enable C=C bond formation via coupling of alkyl halides with aldehydes and their derivatives using triphenylphosphine as a reductive quencher. In developing a method for direct coupling of alkyl halides with aldehydes, we hoped to introduce a new paradigm for C=C bond construction that would (1) provide rapid access to terminal and internal alkenes and 3,3,3-trifluoropropenyl derivatives and (2) enable C=C bond formation in aqueous solvent mixtures.

**Figure 1:**
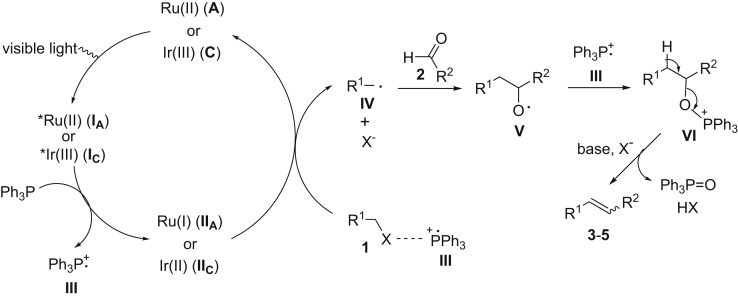
Proposed Mechanism for the Cross-Coupling of Alkyl Halides with Aldehydes

A proposed mechanism for the coupling of alkyl halides with aldehydes is described in Figure 1. Initial visible-light excitation of the photocatalyst [Ru(bpy)_3_]Cl_2_ (**A**) or [Ir(ppy)_2_](dtbbpy)PF_6_ (**C**) (dtbbpy = 4,4′-di-*tert*-butyl-2,2′-bipyridine) would yield excited-state *Ru(II) (**I**_**A**_) or *Ir(III) (**I**_**C**_) complex. The complex (**I**_**A**_ or **I**_**C**_) is a strong single-electron oxidant (half-wave redox potential E_1/2_^red^ [*Ru^II^/Ru^I^] = +0.77 V [Prier et al., 2013]; E_1/2_^red^ [*Ir^III^/Ir^II^] = + 0.66 V versus the saturated calomel electrode [SCE] in CH_3_CN [Lowry et al., 2005]) and should undergo reduction by triphenylphosphine (E_1/2_^red^ = 0.87 V versus SCE in MeCN [Yasui et al., 2000]) to give Ru(I) (**II**_**A**_) or Ir(II) (**II**_**C**_) complex and radical cation Ph_3_P^⋅+^ (**III**) (Fearnley et al., 2016). A single electron transfer from Ru(I) (**II**_**A**_) or Ir(II) (**II**_**C**_) to alkyl halide (**1**) is endergonic but might be possible under the assistance of **III** via formation of a charge-transfer complex. This should provide alkyl radical **IV** and halo anion (X^−^) and regenerate photocatalyst **A** or **C**. The resulting alkyl radical **IV** is expected to rapidly react with aldehyde **2** to produce oxygen-centered radical **V** (Kawamoto et al., 2012). This intermediate is prone to *β*-scission, but combination of **V** with Ph_3_P^⋅+^ (**III**) to give oxyphosphonium ion **VI** would drive the reaction forward. Finally, elimination of **VI** in the presence of base forges the desired C=C bond to furnish the coupled product (**3**, **4,** or **5**).

## Results and Discussion

### Optimization Study

Generally, terminal alkenes are prepared via Wittig coupling of aldehydes with methyltriphenylphosphonium halide in the presence of strong bases such as potassium *tert*-butoxide. We realized that it should be more simple, economical, and practical if they were synthesized through coupling of alkyl halides with paraformaldehyde or aqueous formaldehyde in the presence of common inorganic bases and triphenylphosphine (PPh_3_), so we subsequently started optimization of conditions on the visible-light photoredox olefination of alkyl halides (**1**) with paraformaldehyde (**2a**). As shown in Table 1, 4-bromobenzyl bromide (**1a**) was selected as the reaction partner to optimize conditions, including photocatalysts, bases, solvents, amount of triphenylphosphine, and reaction time. Four common ruthenium and iridium complexes, [Ru(bpy)_3_]Cl_2_⋅6H_2_O (**A**), [*fac*-Ir(ppy)_3_] (**B**), [Ir(ppy)_2_](dtbbpy)PF_6_ (**C**), and [Ir(dFCF_3_ppy)_2_](dtbbpy)PF_6_ (**D**), were screened as photocatalysts (entries 1–4) using Cs_2_CO_3_ as the base and acetonitrile as the solvent in the presence of 1.5 equiv of PPh_3_ under argon atmosphere at room temperature for 6 hr, and [Ru(bpy)_3_]Cl_2_⋅6H_2_O (**A**) exhibited the highest catalytic activity, providing 1-bromo-4-vinylbenzene (**3i**) in 93% yield with triphenylphosphine oxide as a by-product appearing in 95% yield (entry 1). Meanwhile, [Ir(ppy)_2_](dtbbpy)PF_6_ (**C**) afforded **3i** in 87% yield (entry 3). Other bases, K_2_CO_3_ (entry 5) and Na_2_CO_3_ (entry 6), were screened, and K_2_CO_3_ afforded the same yield as Cs_2_CO_3_ (compare entries 1 and 5) but Na_2_CO_3_ gave a slightly lower yield (entry 6). Only small amounts of target product were observed in the absence of base (entry 7). The stoichiometry of PPh_3_ was changed, and we found that 1.5 equiv of PPh_3_ was optimal (compare entries 5, 8, and 9). The reaction did not work in the absence of PPh_3_ (entry 10). We investigated reaction time (entries 11 and 12) and found that the reaction completed within 4 hr. Other solvents were tested (entries 13–16), and they were inferior to MeCN. Reactions in polar protic solvents such as ethanol, isopropanol, and *tert*-butanol did not deliver the product. Aqueous formaldehyde (**2b**) (37% aqueous solution) could be used instead of paraformaldehyde (**2a**) to give the product (**3i**) in a reasonable yield (84%) (entry 17). The reaction was carried out under irradiation of a 5-W blue light-emitting diode for 9 hr, and a yield similar to the one in entry 11 was obtained (entry 18), which indicated that the UV part of the compact fluorescent light (CFL) emission spectrum was not mandatory and that the reaction proceeded indeed under visible-light irradiation. The presence of air inhibited the reaction (entry 19). Only trace amounts of target product were observed in the absence of photocatalyst (entry 20) or visible light (entry 21). Therefore, the optimized conditions for synthesis of terminal alkenes are as follows: [Ru(bpy)_3_]Cl_2_⋅6H_2_O (**A**) as the photocatalyst, K_2_CO_3_ as the base, and 1.5 equiv of PPh_3_ in MeCN as the solvent under argon atmosphere at room temperature.

**Table 1:** Optimization of Conditions for Visible-Light Photoredox Olefination


Entry	PC	Base (equiv)	Solvent	Time (h)	Yield^a^
1	**A**	Cs_2_CO_3_	CH_3_CN	6	93
2	**B**	Cs_2_CO_3_	CH_3_CN	6	21
3	**C**	Cs_2_CO_3_	CH_3_CN	6	89
4	**D**	Cs_2_CO_3_	CH_3_CN	6	67
5	**A**	K_2_CO_3_	CH_3_CN	6	93
6	**A**	NA_2_CO_3_	CH_3_CN	6	61
7^b^	**A**	–	CH_3_CN	6	11
8^c^	**A**	K_2_CO_3_	CH_3_CN	6	92
9^d^	**A**	K_2_CO_3_	CH_3_CN	6	80
10^e^	**A**	K_2_CO_3_	CH_3_CN	6	NR
11	**A**	K_2_CO_3_	CH_3_CN	4	93
12	**A**	K_2_CO_3_	CH_3_CN	3	88
13	**A**	K_2_CO_3_	DMF	4	90
14	**A**	K_2_CO_3_	DMA	4	74
15	**A**	K_2_CO_3_	DMSO	4	43
16	**A**	K_2_CO_3_	CH_2_Cl_2_	4	45
17^f^	**A**	K_2_CO_3_	CH_3_CN	4	84
18^g^	**A**	K_2_CO_3_	CH_3_CN	9	90
19^h^	**A**	K_2_CO_3_	CH_3_CN	4	Trace
20^i^	–	K_2_CO_3_	CH_3_CN	4	Trace
21^j^	**A**	K_2_CO_3_	CH_3_CN	4	Trace

*Reaction conditions*: Ar atmosphere and irradiation of visible light with 23-W CFL, 4-bromobenzyl bromide (**1a**) (1.0 mmol), paraformaldehyde (**2a**) (2.0 mmol, relative to amount of formaldehyde), triphenylphosphine (PPh_3_) (1.5 mmol), photocatalyst (5.0 μmol), base (1.5 mmol), solvent (10 mL), temperature (room temperature ∼25 ^o^C), time 3–6 hr, in a sealed Schlenk tube.

PC, photocatalyst; CFL, compact fluorescent light; DMA, N,N-dimethylacetamide; NR, no reaction.

a Isolated yield.

b No base.

c In the presence of 2 equiv of PPh_3_.

d In the presence of 1 equiv of PPh_3_.

e No PPh_3_.

f Using aqueous formaldehyde (**2b**) (37% aqueous solution) (2.0 mmol) instead of paraformaldehyde (**2a**).

g Under irradiation of 5-W blue LED light for 9 hr.

h The reaction was carried out in air.

i No photocatalyst.

j No light.

### Scope of the Investigation

**Figure 2:**
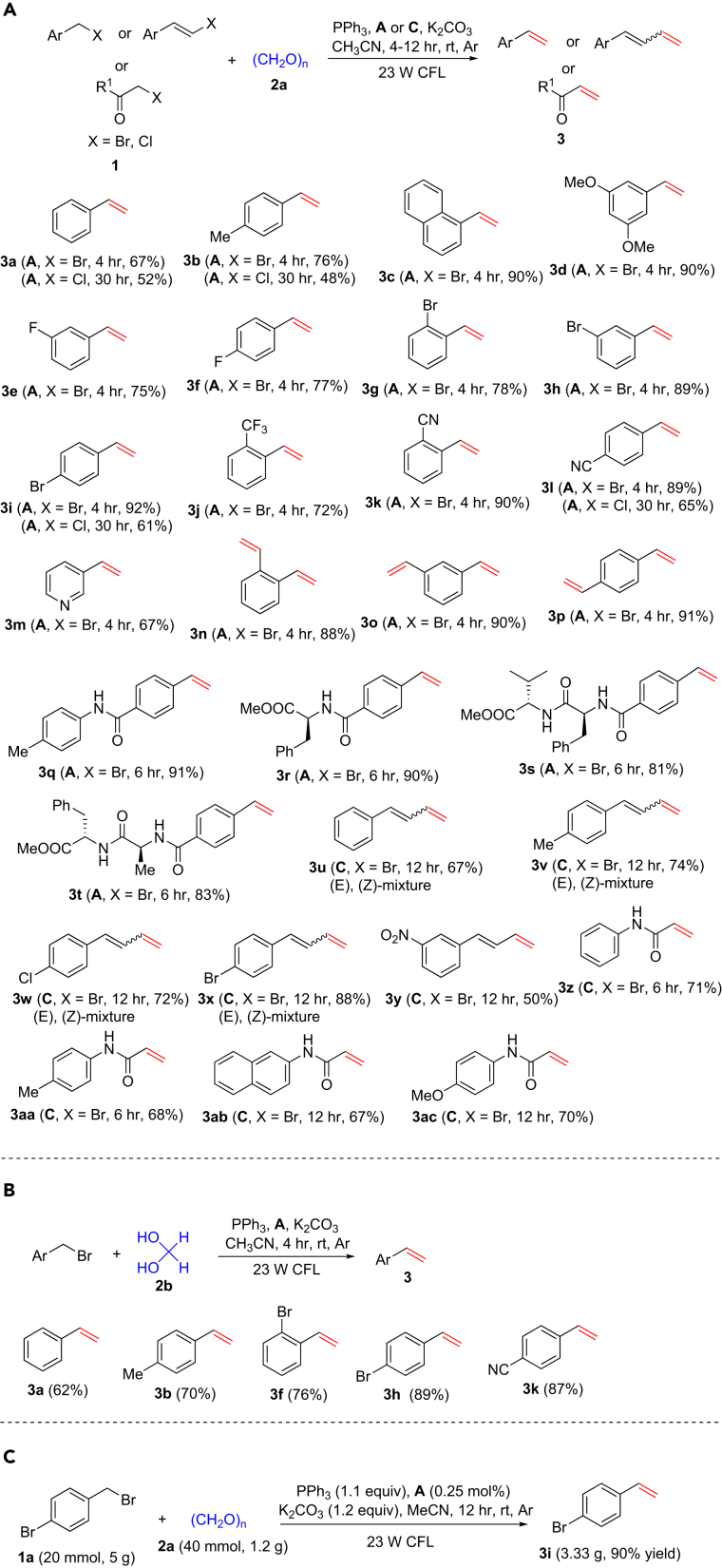
Visible-Light Photoredox Synthesis of Terminal Alkenes (A) Synthesis of terminal alkenes with paraformaldehyde (**2a**). (B) Synthesis of terminal alkenes with hydrous formaldehyde (**2b**). (C) Gram-scale synthesis of **3i**. *Reaction conditions*: Ar atmosphere and irradiation of visible light with 23-W CFL, [Ru(bpy)_3_]Cl_2_⋅6H_2_O (**A**) or [Ir(ppy)_2_]dtbbpyPF_6_ (**C**) (5.0 μmol), alkyl bromide (**1**) (1.0 mmol), paraformaldehyde (**2a**) (4.0 mmol for the synthesis of **3n**–**3p**; 2.0 mmol for the synthesis of the others, relative to the amount of formaldehyde), aqueous formaldehyde (**2b**) (37% aqueous solution) (2.0 mmol), triphenylphosphine (PPh_3_) (3.0 mmol for synthesis of **3n**–**3p**; 1.5 mmol for synthesis of the others), K_2_CO_3_ (3.0 mmol for synthesis of **3n**–**3p**; 1.5 mmol for synthesis of the others), MeCN (10 mL), temperature (room temperature [rt], ∼25°C), time, 4–12 hr, in a sealed Schlenk tube. Isolated yield. E/Z ratios were determined by ^1^H nuclear magnetic resonance spectroscopy. See Transparent Methods for experimental details.

With optimal conditions in hand, we probed the generality of this process with respect to alkyl halides (**1**) (Figure 2A). At first, various substituted benzyl bromides or chlorides were tested using **A** as the photocatalyst, and they provided the corresponding terminal alkenes in good to excellent yields (see **3a–3m**), with bromides exhibiting higher reactivity than chlorides. When 1,2-dibenzyl bromide; 1,3-dibenzyl bromide; and 1,4-dibenzyl bromide were used as the substrates, 1,2-divinylbenzene; 1,3-divinylbenzene; and 1,4-divinylbenzene were prepared in satisfactory yields (see **3n–3p**). Benzyl bromide derivatives containing amides, such as substrates derived from amino acids, also performed very well under the standard conditions (see **3q–3t**). (*E*)-(3-bromoprop-1-en-1-yl)benzene and bromoacetamide derivatives (Nakajimaet al., 2014) were used in visible-light olefination using [Ir(ppy)_2_]dtbbpyPF_6_ (**C**) as the photocatalyst and afforded the corresponding terminal alkenes uneventfully (see **3u–3aa**). Subsequently, olefination of substituted benzyl bromides with aqueous formaldehyde (**2b**) was investigated. As shown in Figure 2B, the reactions also provided the corresponding terminal alkenes in good yields. We performed a gram-scale experiment using **1a** as an example under the standard conditions, and 3.3 g of 1-bromo-4-vinylbenzene (**3i**) was obtained in 90% yield (Figure 2C). This result shows that this visible-light-mediated method is effective and practical for the synthesis of styrene and acrylamide derivatives. Unfortunately, other kinds of unactivated alkyl halides were not successful substrates under the present conditions, and further investigations are underway. The method tolerates various functional groups including ethers; aryl C-F and C-Br bonds; trifluoromethyl, cyano, and nitro groups; and *N*-heterocycles, amides, and esters, and no epimerization was observed on using amino acid-derived substrates. Notably, the olefination of alkyl halides is carried out in a one-pot system, which avoids the separate preparation of the Wittig reagents. In addition, the method displays several advantages including mild conditions; use of inexpensive and readily available paraformaldehyde, aqueous formaldehyde, and common inorganic bases; as well as easy operation and workup procedures.

**Figure 3:**
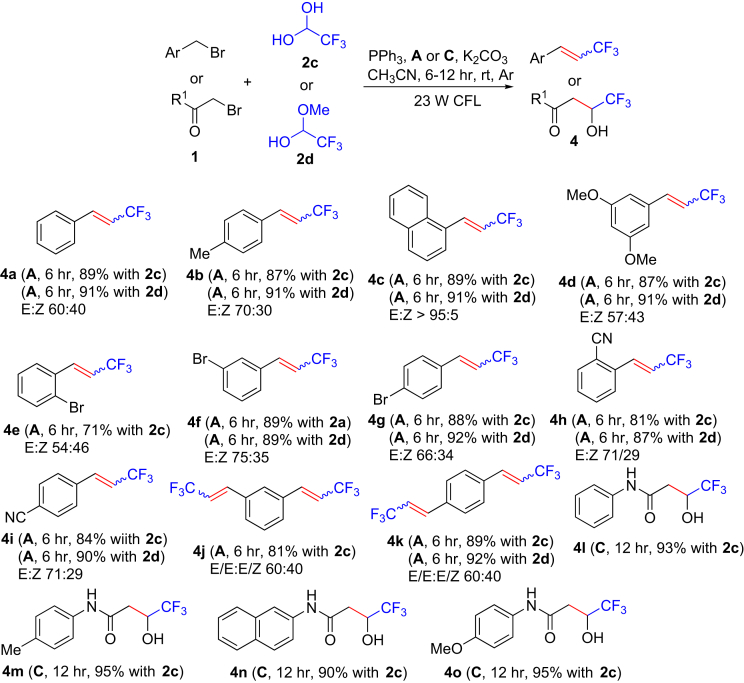
Synthesis of 3,3,3-Trifluoropropenes and 4,4,4-Trifluoro-3-hydroxybutanamides Reaction conditions: Ar atmosphere and irradiation of visible light with 23-W CFL; Ru(bpy)_3_Cl_2_⋅6H_2_O (**A**) or Ir(ppy)_2_dtbbpyPF_6_ (**C**) (5.0 μmol); alkyl bromide (**1**) (1.0 mmol); 2,2,2-trifluoroacetaldehyde hydrate (**2c**) (75% aqueous solution) (2.2 mmol for synthesis of **4j** and **4k**; 1.1 mmol for others); 2,2,2-trifluoro-1-methoxyethanol (**2d**) (1.1 mmol); triphenylphosphine (PPh_3_) (3.0 mmol for synthesis of **4j** and **4k**; 1.5 mmol for synthesis of the others); K_2_CO_3_ (3.0 mmol for synthesis of **4j** and **4k**; 1.5 mmol for synthesis of the others); MeCN (10 mL); temperature (room temperature [rt], ∼25°C); time, 6–12 hr; in a sealed Schlenk tube. Isolated yield. E/Z ratios were determined by ^1^H nuclear magnetic resonance spectroscopy. See Transparent Methods for experimental details.

It is well known that the CF_3_ group is ubiquitous in pharmaceuticals, agrochemicals, and functional materials, resulting in elevated electronegativity, hydrophobicity, metabolic stability, and bioavailability (Banks et al., 1994, Filler and Kobayashi, 1982, Jeschke, 2004, Mueller et al., 2007, Purser et al., 2008, Shimizu and Hiyama, 2005, Welch and Eswarakrishman, 1991) compared with their non-fluorinated counterparts, so it is highly desirable to develop efficient and practical methods for introducing the trifluoromethyl group into organic molecules (Liang et al., 2013, Ma and Cahard, 2007, Schlosser, 2006, Tomashenko and Grushin, 2011). Inspired by the excellent results mentioned above, we explored the coupling of benzyl halides with 2,2,2-trifluoroacetaldehyde hydrate (**2c**) (75% aqueous solution) or 2,2,2-trifluoro-1-methoxyethanol (**2d**) (Figure 3). Reaction of substituted benzyl bromides with **2c** or **2d** led to substituted 3,3,3-trifluoropropenes under similar conditions to those in Figure 2A, and treatment of bromoacetamides with **2c** provided 4,4,4-trifluorobut-2-enamides, which after *in situ* Michael addition of water afforded 4,4,4-trifluoro-3-hydroxybutanamides (**4l–4o**). This visible-light-mediated method introducing the trifluoromethyl group afforded good to excellent yields with tolerance of several functional groups, although no E/Z selectivity was observed for the synthesis of 3,3,3-trifluoropropenes.

**Figure 4:**
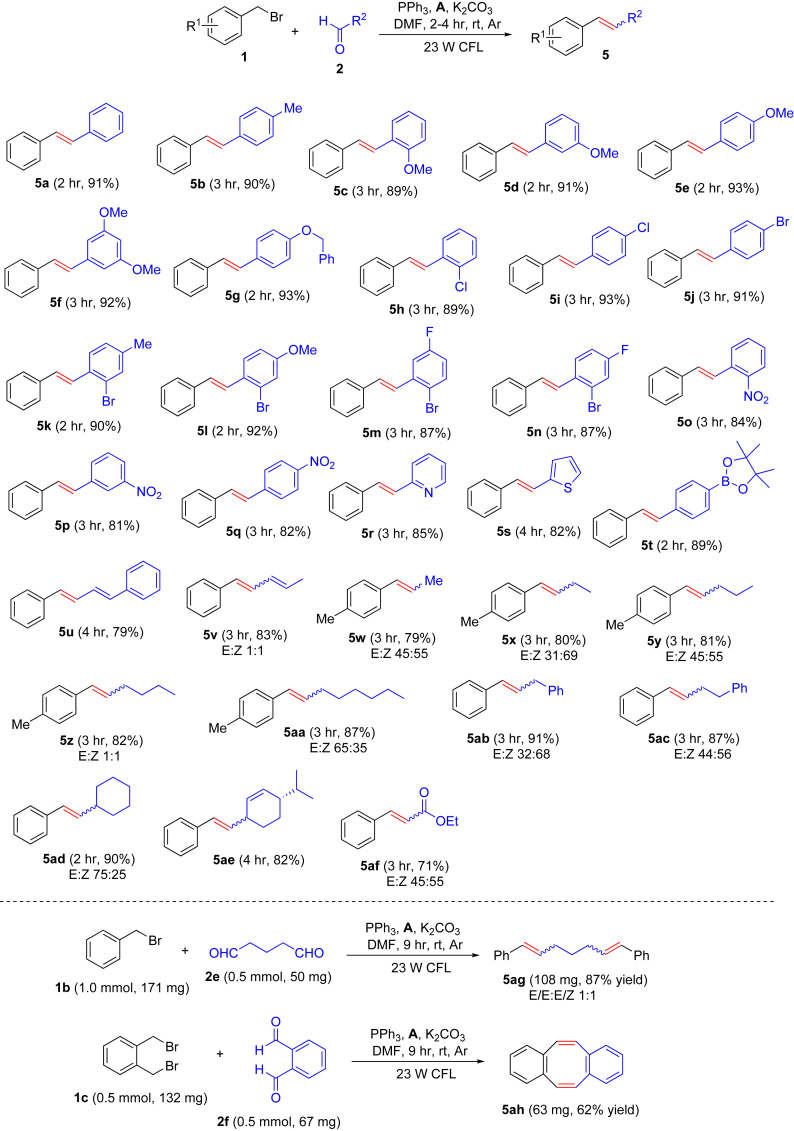
Variation of Aldehydes on Visible-Light Photoredox Olefination *Reaction conditions*: Ar atmosphere and irradiation of visible light with 23-W CFL, Ru(bpy)_3_Cl_2_⋅6H_2_O (**A**) (5.0 μmol) or Ir(ppy)_2_dtbbpyPF_6_ (**C**) (10 μmol), alkyl bromide (**1**) (1.5 mmol), aldehyde (**2**) (1.0 mmol), triphenylphosphine (PPh_3_) (1.5 mmol), K_2_CO_3_ (1.5 mmol), DMF (2.0 mL), temperature (room temperature [rt] ∼25°C), time 2–9 hr, in a sealed Schlenk tube. Isolated yield. E/Z ratios were determined by ^1^H nuclear magnetic resonance spectroscopy. See Transparent Methods for experimental details.

Next, various common aldehydes (**2**) were tested using substituted benzyl bromides (**1**) as partners (Figure 4). All benzaldehyde derivatives exhibited high reactivity, and the presence of neutral, electron-donating, and electron-withdrawing groups on the aromatic rings did not obviously affect the yields (see **5a–5t**). α,β-Unsaturated aldehydes (see **5u** and **5v**) and aliphatic aldehydes (see **5w–5ae**) also proved to be good substrates. We attempted the coupling of benzyl bromide with ethyl glyoxylate, and the target product (**5af**) was obtained in 71% yield. Interestingly, the reactions of bromomethyl arenes with aromatic aldehydes only provided *trans*-alkenes, whereas mixtures of *cis*- and *trans*-alkenes were obtained for the other substrates. Reaction of glutaraldehyde (**2e**) with 2 equiv of benzyl bromide (**1b**) gave diene **5ag** in 87% yield. Interestingly, coupling of 1,2-bis(bromomethyl)benzene (**1c**) with *o*-phthalaldehyde (**2f**) provided dibenzo[*a,e*]cyclooctene **5ah** in 62% yield. The results showed that the present method is nearly universal with respect to the aldehyde scope.

**Figure 5:**
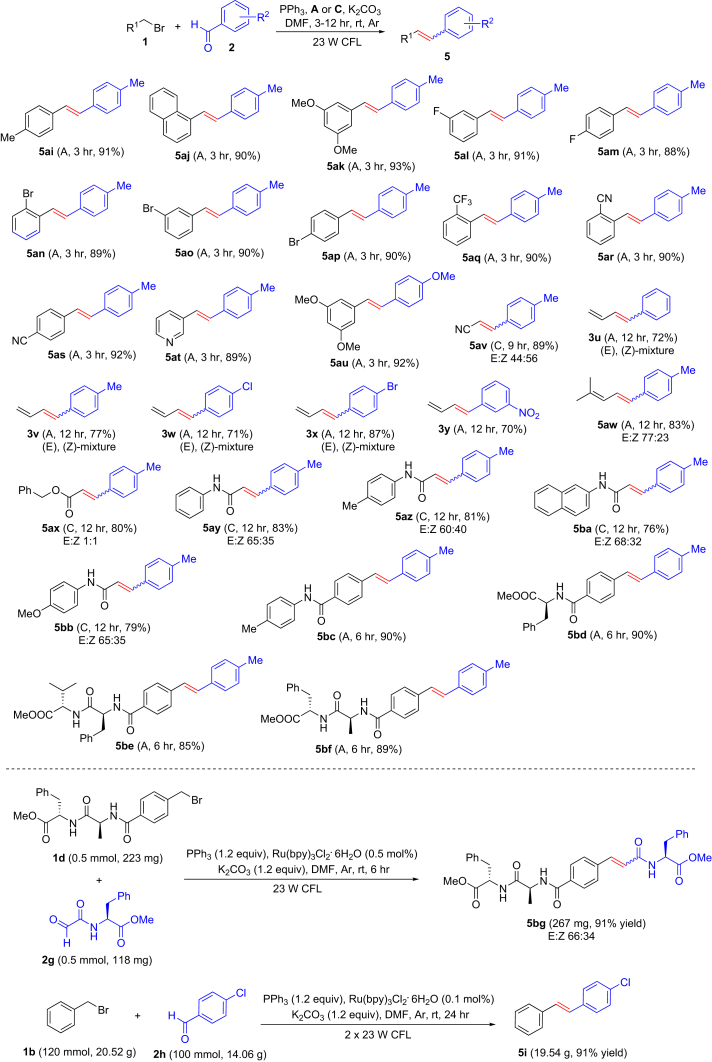
Variation of Alkyl Halides on Visible-Light Photoredox Olefination *Reaction conditions*: Ar atmosphere and irradiation of visible light with 23-W CFL, Ru(bpy)_3_Cl_2_⋅6H_2_O (**A**) (5.0 μmol) or Ir(ppy)_2_dtbbpyPF_6_ (**C**) (10 μmol), alkyl bromide (**1**) (1.5 mmol), aldehyde (**2**) (1.0 mmol), triphenylphosphine (PPh_3_) (1.5 mmol), K_2_CO_3_ (1.5 mmol), DMF (2.0 mL), temperature (room temperature [rt] ∼25°C), time 3–12 hr, in a sealed Schlenk tube. Isolated yield. E/Z ratios were determined by ^1^H nulcear magnetic resonance spectroscopy. See Transparent Methods for experimental details.

We then explored the substrate scope of alkyl halides using benzaldehyde derivatives as partners. As shown in Figure 5, various bromomethyl arenes exhibited high reactivity, and the electronic effects on the aromatic rings did not cause noticeable differences in reactivity (see **5ai–5au**). Bromoacetonitrile, allyl bromide, and 3-bromo-2-methylpropene were also suitable substrates (see **5av–5bb**). Bromoacetic acid derivatives with ester (see **5bc**) and amides (see **5bd–5bg**) were attempted as substrates and displayed high reactivity. Similarly, benzyl bromide derivatives derived from amino acids also gave the target products in high yields (see **5bh–5bk**). A one-to-one late-stage fragment coupling between dipeptide **1d** and amino acid derivative **2g** was attempted, and excitingly, conjugate **5bl** was obtained in 91% yield. In addition, a gram-scale experiment was performed using coupling of benzyl bromide (**1b**) with 4-chlorobenzaldehyde (**2h**) as an example; 1-chloro-4-(2-phenylvinyl)benzene (**5i**) (19.5 g) was obtained in 91% yield under irradiation of two 23-W CFL bulbs. The results indicate that the present method is effective for diverse alkyl halides and might be applicable to peptide stapling and bioconjugation reactions.

It should be pointed out that there are limitations and possible disadvantages to the present method, including use of excess amount of triphenylphosphine and additional photoredox catalysts.

### Mechanistic Study

**Figure 6:**
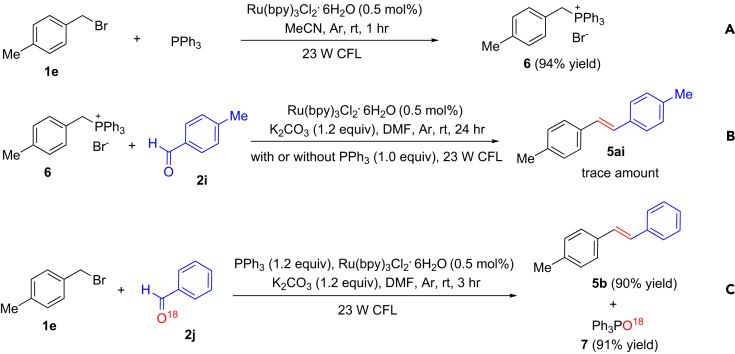
Investigation of Mechanism for the Visible-Light Photoredox Olefination (A) Treatment of 4-methylbenzyl bromide (**1e**) (0.3 mmol) with triphenylphosphine (1.0 mmol) in the absence of aldehyde and base under the standard conditions. (B) Treatment of (4-methylbenzyl)triphenylphosphonium bromide (**6**) (0.3 mmol) with **2i** (0.2 mmol) in the presence or absence of pph_3_ (0.3 mmol) under the standard conditions. (C) Treatment of 4-methylbenzyl bromide (**1e**) (1.5 mmol) with ^18^O-labeled aldehyde (**2j**) (1.0 mmol) under the standard conditions.

To explore the mechanism for the visible-light photoredox olefination, we carried out some control experiments as follows. (1) Treatment of 4-methylbenzyl bromide (**1e**) with PPh_3_ in the absence of aldehyde and base provided (4-methylbenzyl)triphenylphosphonium bromide (**6**) in 94% yield (Figure 6A), but only less amounts of product were observed in the dark. (2) Treatment of (4-methylbenzyl)triphenylphosphonium bromide (**6**) with **2i** under the standard conditions with or without addition of an extra equivalent of PPh_3_ only provided trace amounts of **5ai** (Figure 6B), which implies that triphenylphosphonium bromides are not intermediates in the visible-light photoredox olefination. The result shows that the Wittig reagents are not reduced by a reductive quenching cycle involving PPh_3_, even though their reduction potentials seem more accessible than the ones of benzyl bromides (Matschiner and Issleib, 1967). It also shows that the base does not deprotonate the ylide to do a classical Wittig reaction. (3) Reaction of **1a** with ^18^O-labeling benzaldehyde (**2j**) under the standard conditions provided **5b** and ^18^O-labeled triphenylphosphine oxide (**7**) in 90% and 91% yields, respectively (Figure 6C). The result shows that oxygen in triphenylphosphine oxide originates from the aldehyde. We also investigated types of radicals produced during the reactions by electron spin resonance (see Supplemental Information). The results above indicate that the process for the visible-light photoredox olefination in Figure 1 is reasonable (see Supplemental Information for more mechanistic studies). This report is the first example of broadly applicable reduction of simple benzyl halides by visible-light photoredox catalysis, and more detailed mechanistic studies are underway to better understand this key step in the catalytic cycle.

### Conclusion

We have developed an efficient and practical olefination of alkyl halides with aldehydes by visible-light photoredox catalysis using triphenylphosphine as a reductive quencher. The present method exhibits several advantages including operational simplicity, mild reaction conditions, wide functional group tolerance, and amenability to gram-scale synthesis. More importantly, paraformaldehyde; aqueous formaldehyde; 2,2,2-trifluoroacetaldehyde hydrate; and 2,2,2-trifluoro-1-methoxyethanol are also effective substrates, and the corresponding terminal alkenes and CF_3_-containing molecules were prepared in good to excellent yields. We believe that the present method will find wide application in the synthesis of organic molecules, natural products, biological molecules, and polymers.

## Methods

All methods can be found in the accompanying Transparent Methods supplemental file.

## Supplemental Information

Document S1. Transparent methods and Figures S1–S240

## References

[bib2] Banks R.E., Smart B.E., Tatlow J.C. (1994). Organofluorine Chemistry: Principles and Commercial Applications.

[bib3] Baudin J.B., Hareau G., Julia S.A. (1991). A direct synthesis of olefins by reaction of carbonyl-compounds with lithio derivatives of 2-[alkyl-sulfonyl or (2'-alkenyl)-sulfonyl or benzyl-sulfonyl]-benzothiazoles. Tetrahedron Lett..

[bib4] Blakemore P.R., Cole W.J., Kocienski P.J., Morley A. (1998). A stereoselective synthesis of *trans*-1,2-disubstituted alkenes based on the condensation of aldehydes with metallated 1-phenyl-1*H*-tetrazol-5-yl sulfones. Synlett.

[bib5] Calderon N., Chen H.Y., Scott K.W. (1967). Olefin metathesis - a novel reaction for skeletal transformations of unsaturated hydrocarbons. Tetrahedron Lett..

[bib6] Clayden J., Greeves N., Warren S., Wothers P. (2001). Organic Chemistry.

[bib64] Fearnley A.F., An J., Jackson M., Lindovska P., Denton R.M. (2016). Synthesis of quaternary aryl phosphonium salts: photoredox-mediated phosphine arylation. Chem. Commun..

[bib9] Filler R., Kobayashi Y. (1982). Biomedicinal Aspects of Fluorine Chemistry.

[bib10] Gao C., Li J., Yu J., Yang H., Fu H. (2016). Visible-light photoredox synthesis of internal alkynes containing quaternary carbons. Chem. Commun..

[bib11] Garber S.B., Kingsbury J.S., Gray B.L., Hoveyda A.H. (2000). Efficient and recyclable monomeric and dendritic Ru-based metathesis catalysts. J. Am. Chem. Soc..

[bib12] Hari D.P., König B. (2013). The photocatalyzed Meerwein arylation: classic reaction of aryl diazonium salts in a new light. Angew. Chem. Int. Ed..

[bib13] Jeschke P. (2004). The unique role of fluorine in the design of active ingredients for modern crop protection. ChemBioChem.

[bib14] Jiang M., Jin Y., Yang H., Fu H. (2016). Visible-light photoredox synthesis of unnatural chiral α-amino acids. Sci. Rep..

[bib15] Jiang M., Yang H., Fu H. (2016). Visible-light photoredox synthesis of chiral α-selenoamino acids. Org. Lett..

[bib16] Jiang M., Yang H., Fu H. (2016). Visible-light photoredox borylation of aryl halides and subsequent aerobic oxidative hydroxylation. Org. Lett..

[bib17] Jiang M., Li H., Yang H., Fu H. (2017). Room-temperature arylation of thiols: breakthrough with aryl chlorides. Angew. Chem. Int. Ed..

[bib18] Jin Y., Fu H. (2017). Visible-light photoredox decarboxylative couplings. Asian J. Org. Chem..

[bib19] Jin Y., Jiang M., Wang H., Fu H. (2016). Installing amino acids and peptides on *N*-heterocycles under visible-light assistance. Sci. Rep..

[bib20] Jin Y., Yang H., Fu H. (2016). An *N*-(acetoxy)phthalimide motif as a visible-light pro-photosensitizer in photoredox decarboxylative arylthiation. Chem. Commun..

[bib21] Jin Y., Yang H., Fu H. (2016). Thiophenol-catalyzed visible-light photoredox decarboxylative couplings of *N*-(acetoxy)phthalimides. Org. Lett..

[bib22] Jin Y., Ou L., Yang H., Fu H. (2017). Visible-light-mediated aerobic oxidation of N-alkylpyridinium salts under organic photocatalysis. J. Am. Chem. Soc..

[bib23] Julia M., Paris J.-M. (1973). Syntheses using sulfones.5. method for general synthesis of doubles. Tetrahedron Lett..

[bib24] Kawamoto T., Fukuyama T., Ryu I. (2012). Radical addition of alkyl halides to formaldehyde in the presence of cyanoborohydride as a radical mediator. a new protocol for hydroxymethylation reaction. J. Am. Chem.Soc..

[bib25] Kocienski P.J., Lythgoe B., Ruston S. (1978). Scope and stereochemistry of an olefin synthesis from *beta*-hydroxy-sulfones. J. Chem. Soc. Perkin Trans..

[bib26] Kolodiazhnyi O.I. (1999). Phosphorus Ylides: Chemistry and Applications in Organic Chemistry.

[bib27] König B. (2013). Chemical Photocatalysis.

[bib29] Li J., Tian H., Jiang M., Yang H., Zhao Y., Fu H. (2016). Consecutive visible-light photoredox decarboxylative couplings of adipic acid active esters with alkynyl sulfones leading to cyclic compounds. Chem. Commun..

[bib30] Liang T., Neumann C.N., Ritter T. (2013). Introduction of fluorine and fluorine-containing functional groups. Angew. Chem. Int. Ed..

[bib31] Liu K.K.-C., Li J., Sakya S. (2004). Synthetic approaches to the 2003 new drugs. Mini. Rev. Med. Chem..

[bib32] Love J.A., Morgan J.P., Trnka T.M., Grubbs R.H. (2002). A practical and highly active ruthenium-based catalyst that effects the cross metathesis of acrylonitrile. Angew. Chem. Int. Ed..

[bib33] Lowry M.S., Goldsmith J.I., Slinker J.D., Rohl R., Pascal R.A., Malliaras G.G., Bernhard S. (2005). Single-layer electroluminescent devices and photoinduced hydrogen production from an ionic iridium(III) complex. Chem. Mater..

[bib34] Ma J.-A., Cahard D. (2007). Strategies for nucleophilic, electrophilic, and radical trifluoromethylations. J. Fluorine Chem..

[bib35] Maryanoff B.E., Reitz A.B. (1989). The Wittig olefination reaction and modifications involving phosphoryl-stabilized carbanions - stereochemistry, mechanism, and selected synthetic aspects. Chem. Rev..

[bib36] Matschiner H., Issleib K. (1967). Polarographisches verhalten von organoderivaten des arsens und phosphors. III. zur frage des elektrochemischen verhaltens von phosphoniumsalzen [(C_6_H_5_)_3_PR']X an der Hg-elektrode. Z. Anorg. Allg. Chem..

[bib37] Mueller C.K., Faeh C., Diederich F. (2007). Fluorine in pharmaceuticals: looking beyond intuition. Science.

[bib65] Murdzek J.S., Schrock R.R. (1987). Well-characterized olefin metathesis catalysts that contain molybdenum. Organometallics.

[bib38] Nakajima M., Lefebvre Q., Rueping M. (2014). Visible light photoredox-catalysed intermolecular radical addition of α-halo amides to olefins. Chem. Commun..

[bib39] Narayanam J.M.R., Stephenson C.R.J. (2011). Visible light photoredox catalysis: applications in organic synthesis. Chem. Soc. Rev..

[bib40] Nicolaou K.C., Bulger P.G., Sarlah D. (2005). Metathesis reactions in total synthesis. Angew. Chem. Int. Ed..

[bib41] Nicolaou K.C., Härter M.W., Gunzner J.L., Nadin A. (1997). The Wittig and related reactions in natural product synthesis. Liebigs Ann..

[bib42] Nicolaou K.C., Snyder S.A. (2003). Classics in Total Synthesis II. More Targets, Strategies, Methods.

[bib43] Nicolaou K.C., Sorensen E.J. (1996). Classics in Total Synthesis.

[bib44] Peterson D.J. (1968). A carbonyl olefination reaction using silyl-substituted organometallic compounds. J. Org. Chem..

[bib45] Prier C.K., Rankic D.A., MacMillan D.W.C. (2013). Visible light photoredox catalysis with transition metal complexes: applications in organic synthesis. Chem. Rev..

[bib46] Purser S., Moore P.R., Swallow S., Gouverneur V. (2008). Fluorine in medicinal chemistry. Chem. Soc. Rev..

[bib47] Ravelli D., Dondi D., Fagnoni M., Albini A. (2009). Photocatalysis. A multi-faceted concept for green chemistry. Chem. Soc. Rev..

[bib48] Saklani A., Kutty S.K. (2008). Plant-derived compounds in clinical trials. Drug Discov. Today.

[bib49] Schlosser M. (2006). CF_3_-bearing aromatic and heterocyclic building blocks. Angew. Chem. Int. Ed..

[bib50] Scholl M., Ding S., Lee C.W., Grubbs R.H. (1999). Synthesis and activity of a new generation of ruthenium-based olefin metathesis catalysts coordinated with 1,3-dimesityl-4,5-dihydroimidazol-2-ylidene ligands. Org. Lett..

[bib51] Schrock R.R. (1999). Olefin metathesis by molybdenum imido alkylidene catalysts. Tetrahedron.

[bib52] Schwab P., Grubbs R.H., Ziller J.W. (1996). Synthesis and applications of RuCl_2_(=CHR')(PR_3_)_2_: the influence of the alkylidene moiety on metathesis activity. J. Am. Chem. Soc..

[bib53] Shaw M.H., Twilton J., MacMillan D.W.C. (2016). Photoredox catalysis in organic chemistry. J. Org. Chem..

[bib54] Shi L., Xia W. (2012). Photoredox functionalization of C-H bonds adjacent to a nitrogen atom. Chem. Soc. Rev..

[bib55] Shimizu M., Hiyama T. (2005). Modern synthetic methods for fluorine-substituted target molecules. Angew. Chem. Int. Ed..

[bib56] Tomashenko O.A., Grushin V.V. (2011). Aromatic trifluoromethylation with metal complexes. Chem. Rev..

[bib57] Welch J.T., Eswarakrishman S. (1991). Fluorine in Bioorganic Chemistry.

[bib58] Wittig G., Geissler G. (1953). Course of reactions of pentaphenylphosphorus and certain derivatives. Liebigs Ann. Chem..

[bib59] Wittig G., Schollkopf U. (1954). Triphenylphosphinemethylene as an olefin-forming reagent. I. Chem. Ber..

[bib60] Xuan J., Xiao W.-J. (2012). Visible-light photoredox catalysis. Angew. Chem. Int. Ed..

[bib61] Yasui S., Tsujimoto M., Itoh K., Ohno A. (2000). Quenching of a photosensitized dye through single-electron transfer from trivalent phosphorus compounds. J. Org. Chem..

[bib62] Yoon T.P., Ischay M.A., Du J. (2010). Visible light photocatalysis as a greener approach to photochemical synthesis. Nat. Chem..

[bib63] Zeitler K. (2009). Photoredox catalysis with visible light. Angew. Chem. Int. Ed..

