# Supplementary material for: Olefination of Alkyl Halides with Aldehydes by Merging Visible-Light Photoredox Catalysis and Organophosphorus Chemistry
Source: iScience. 2018 Jul 20;6:102–13. doi: 10.1016/j.isci.2018.07.011 (PMC6137709; doi:10.1016/j.isci.2018.07.011)
Supplement: Document S1. Transparent methods and Figures S1–S240 [file mmc1.pdf]

ISCI, Volume *6*

Supplemental Information

Olefination of Alkyl Halides with Aldehydes by Merging Visible-Light Photoredox Catalysis and Organophosphorus Chemistry

Min Jiang, Haijun Yang, Quentin Lefebvre, Jihu Su, and Hua Fu

Supporting Information

Olefination of alkyl halides with aldehydes by merging visible-light photoredox catalysis and organophosphorus chemistry

Min Jiang[1], Haijun Yang[1], Quentin Lefebvre[2], Jihu Su[3] and Hua Fu[1*]

[1] Key Laboratory of Bioorganic Phosphorus Chemistry and Chemical Biology (Ministry of Education), Department of Chemistry, Tsinghua University, Beijing 100084, China. [2] School of Chemistry, University of Bristol, Cantock's Close, Bristol BS8 1TS, UK. [3] CAS Key Laboratory of Microscale Magnetic Resonance, Department of Modern Physics, University of Science and Technology of China, Hefei 230026, China.

Correspondence and requests for materials should be addressed to H.F. (email: fuhua@mail.tsinghua.edu.cn)

Supplemental Figures for ^1H, ^{13}C, ^{11}B and ^{19}F NMR Spectra

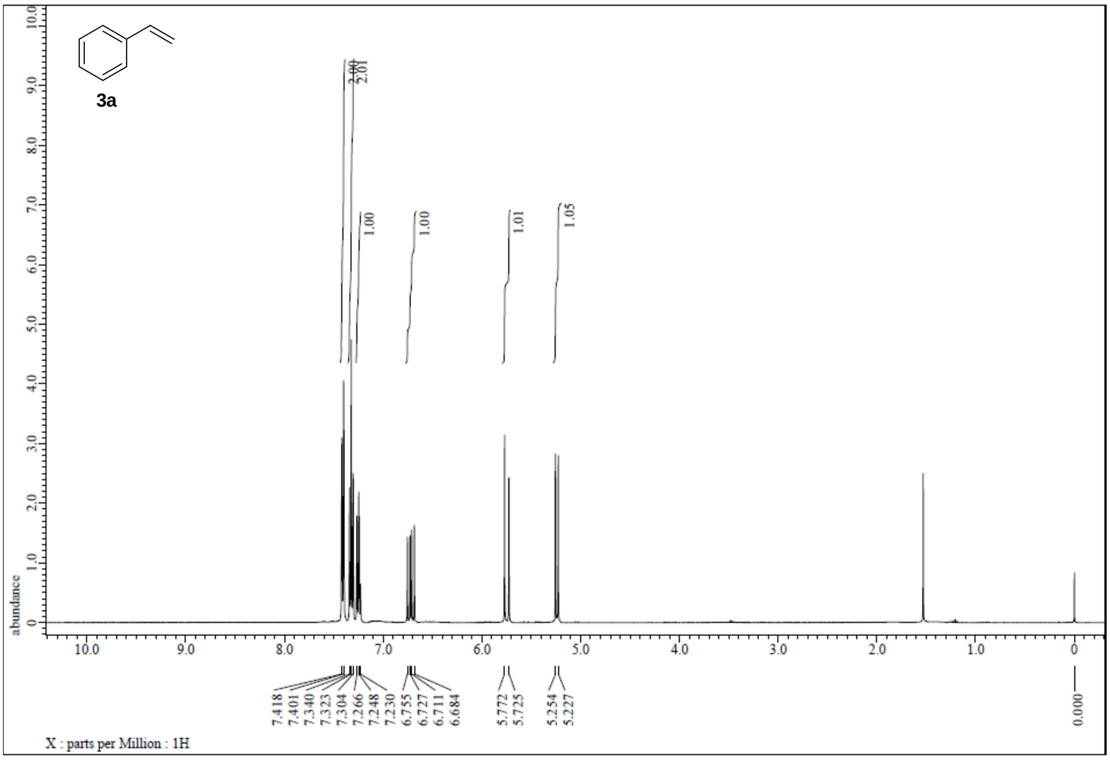

Figure S1. ^1H NMR spectrum of 3a, related to Figure 2.

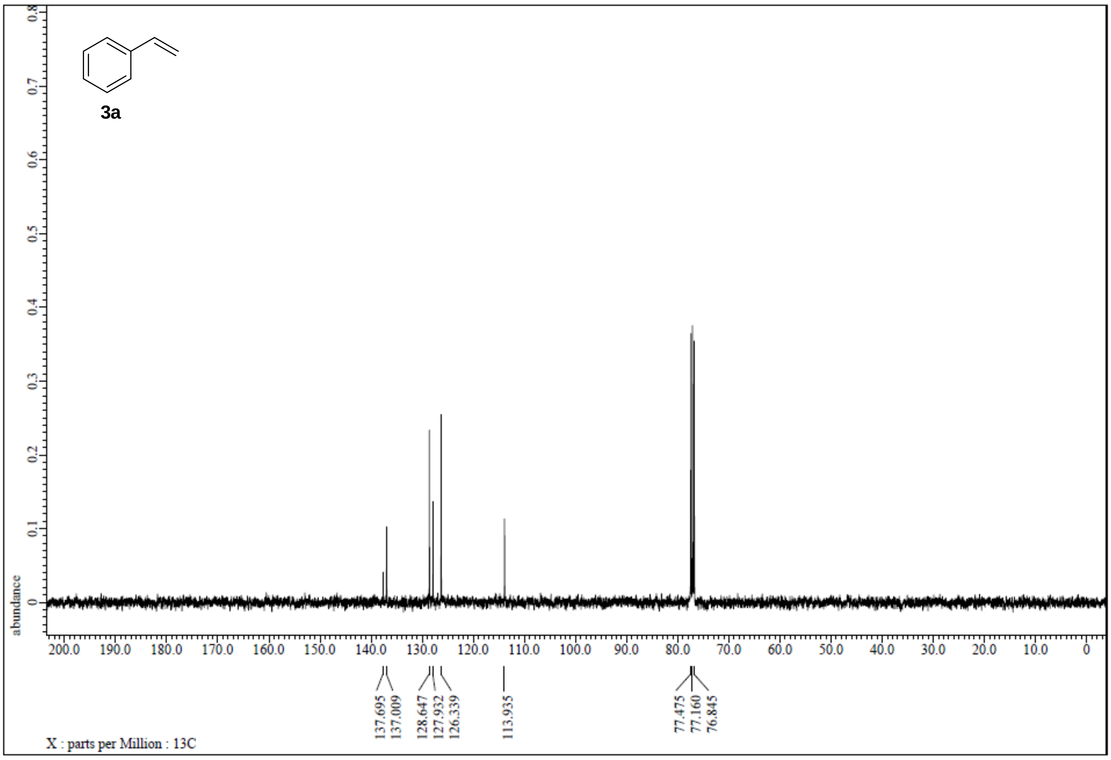

Figure S2. ^{13}C NMR spectrum of 3a, related to Figure 2.

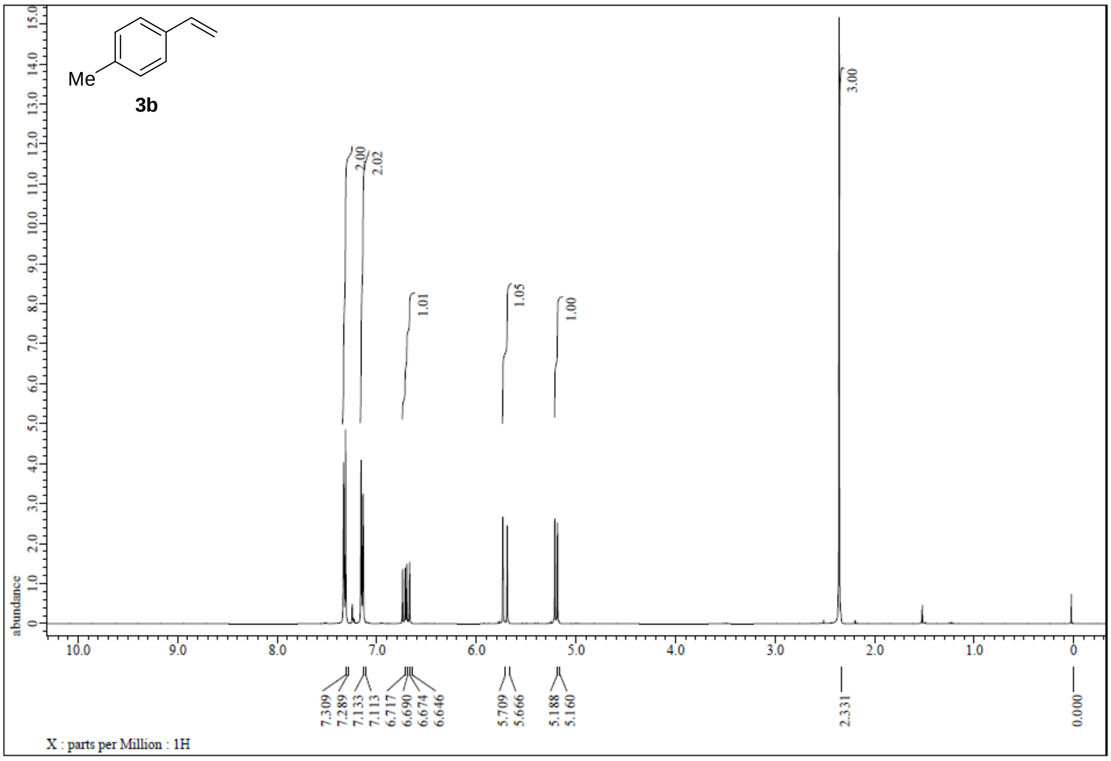

Figure S3. ^{1}H NMR spectrum of 3b, related to Figure 2.

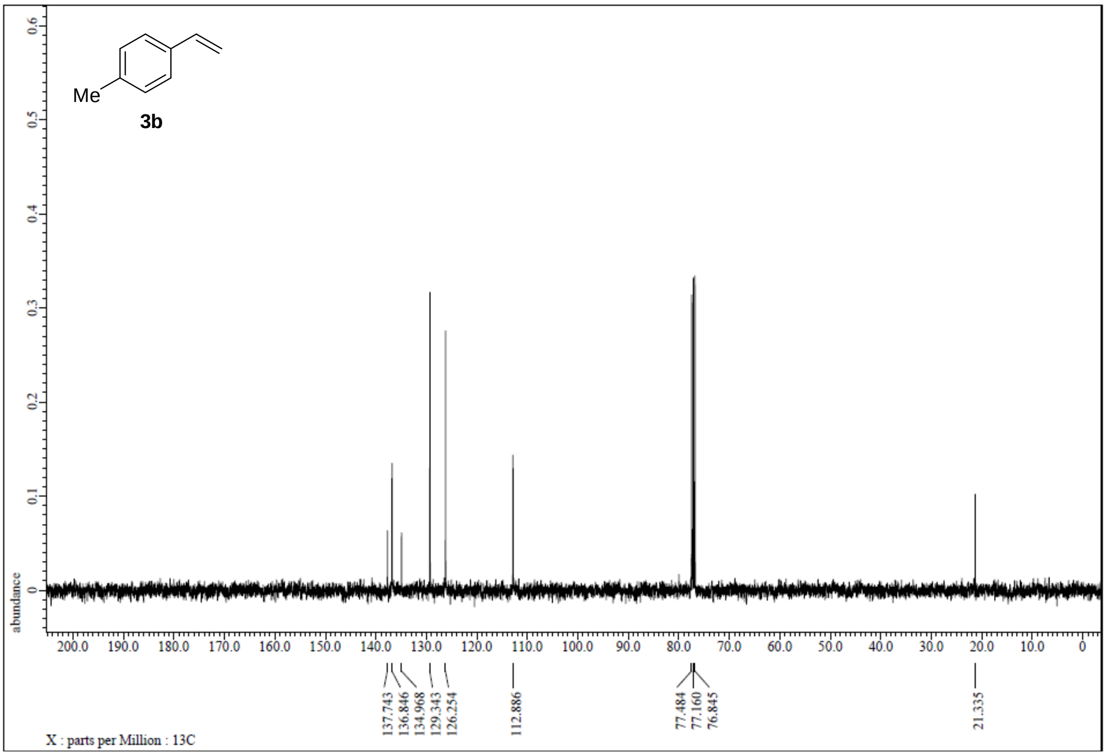

Figure S4. ^{13}C NMR spectrum of 3b, related to Figure 2.

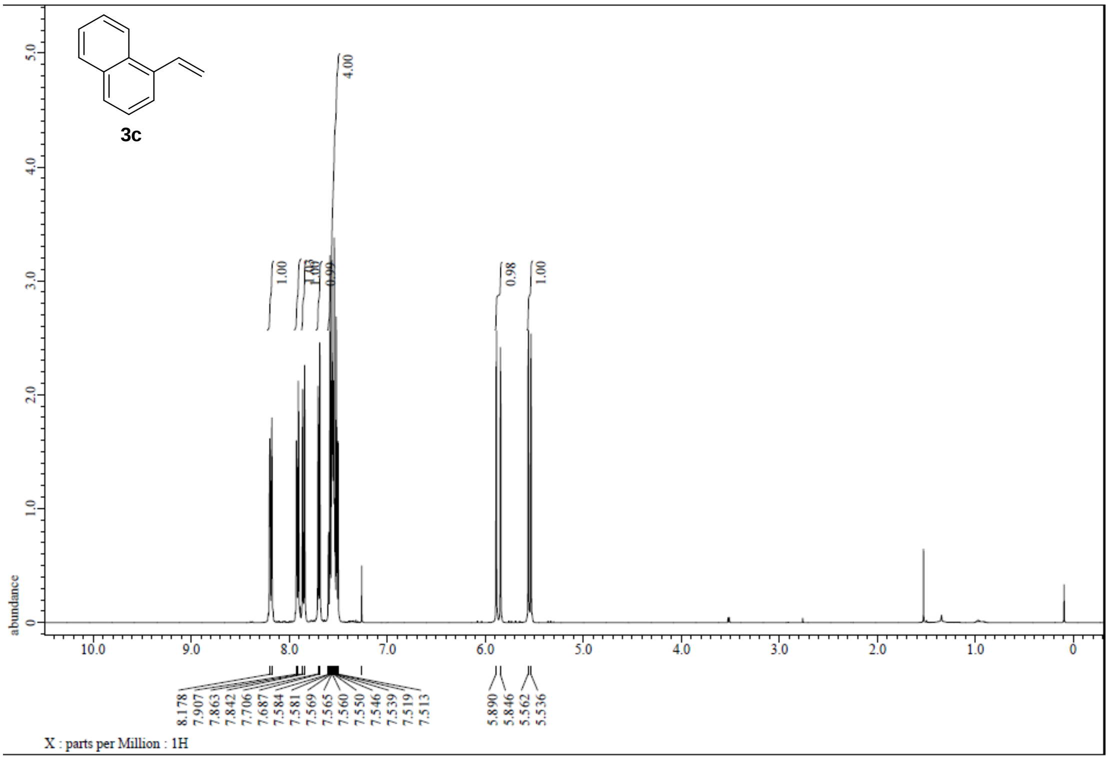

Figure S5. ^{1}**H NMR spectrum of 3c, related to Figure 2**.

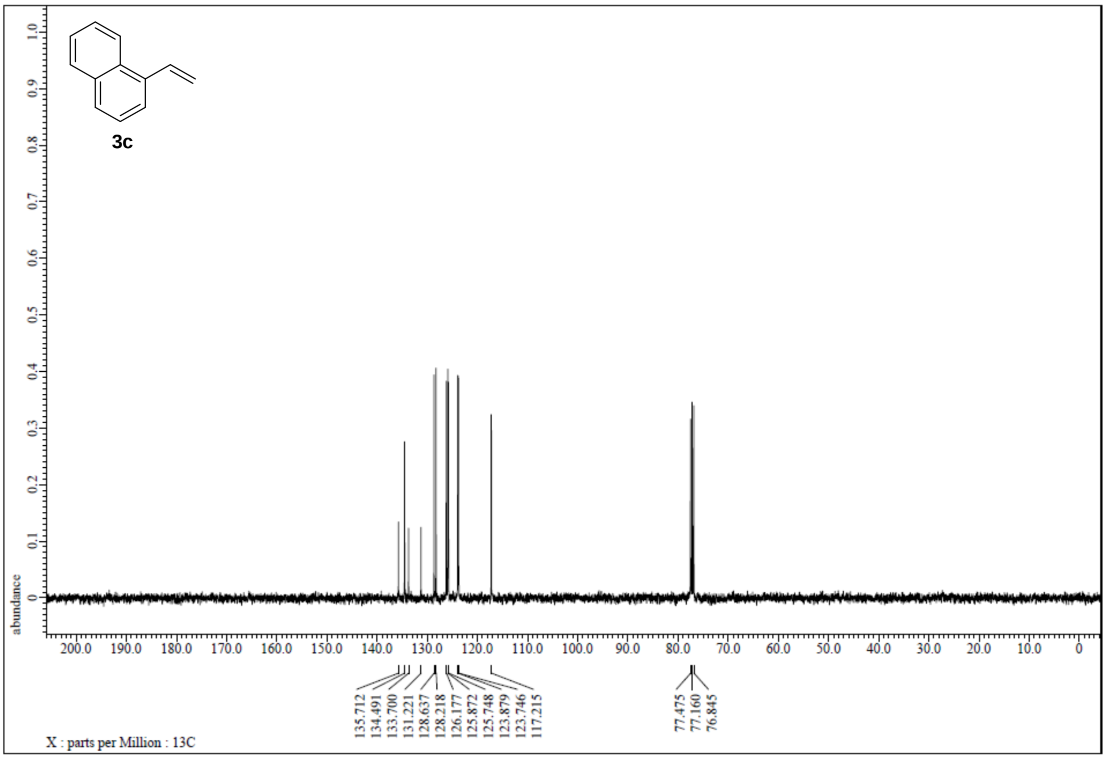

Figure S6. ^{13}**C NMR spectrum of 3c, related to Figure 2**.

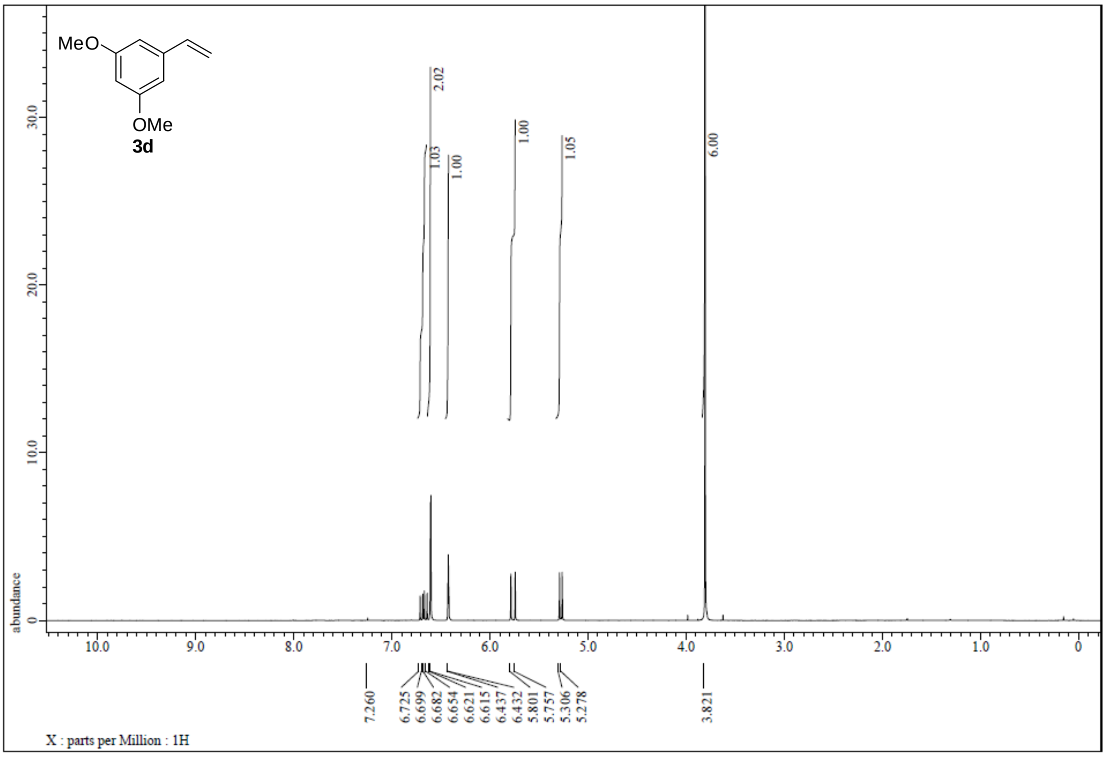

Figure S7. ^{1}H NMR spectrum of 3d, related to Figure 2.

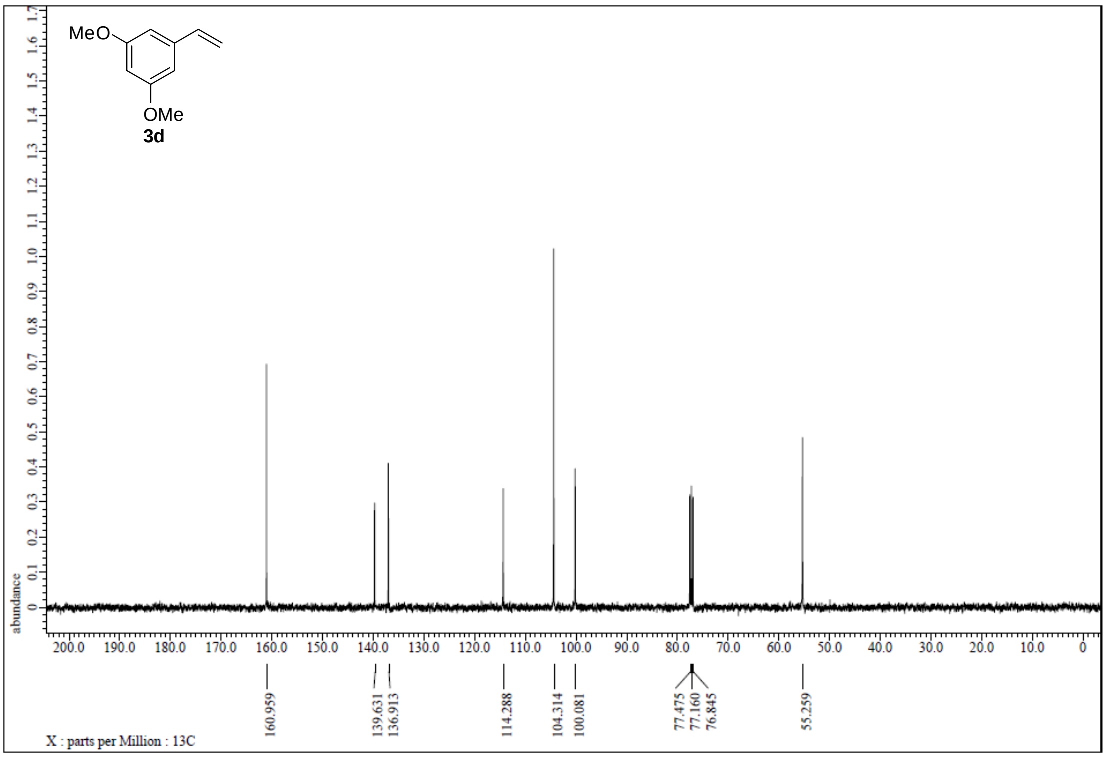

Figure S8. ^{13}C NMR spectrum of 3d, related to Figure 2.

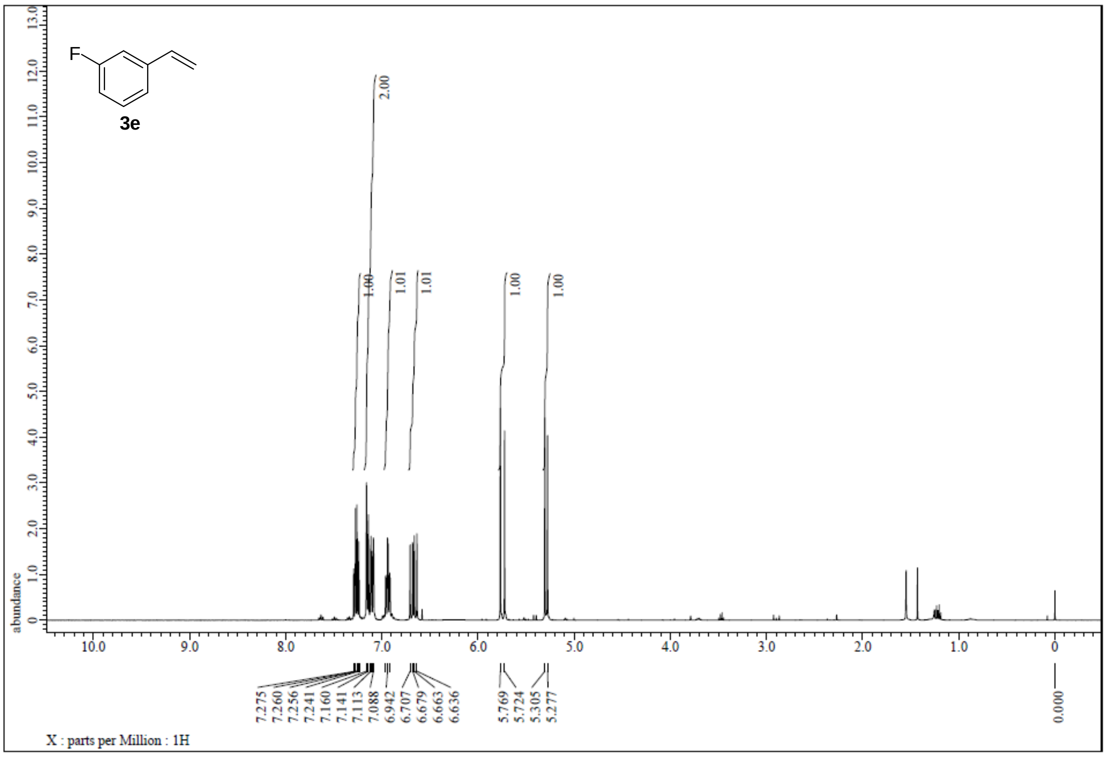

Figure S9. ^{1}H NMR spectrum of 3e, related to Figure 2.

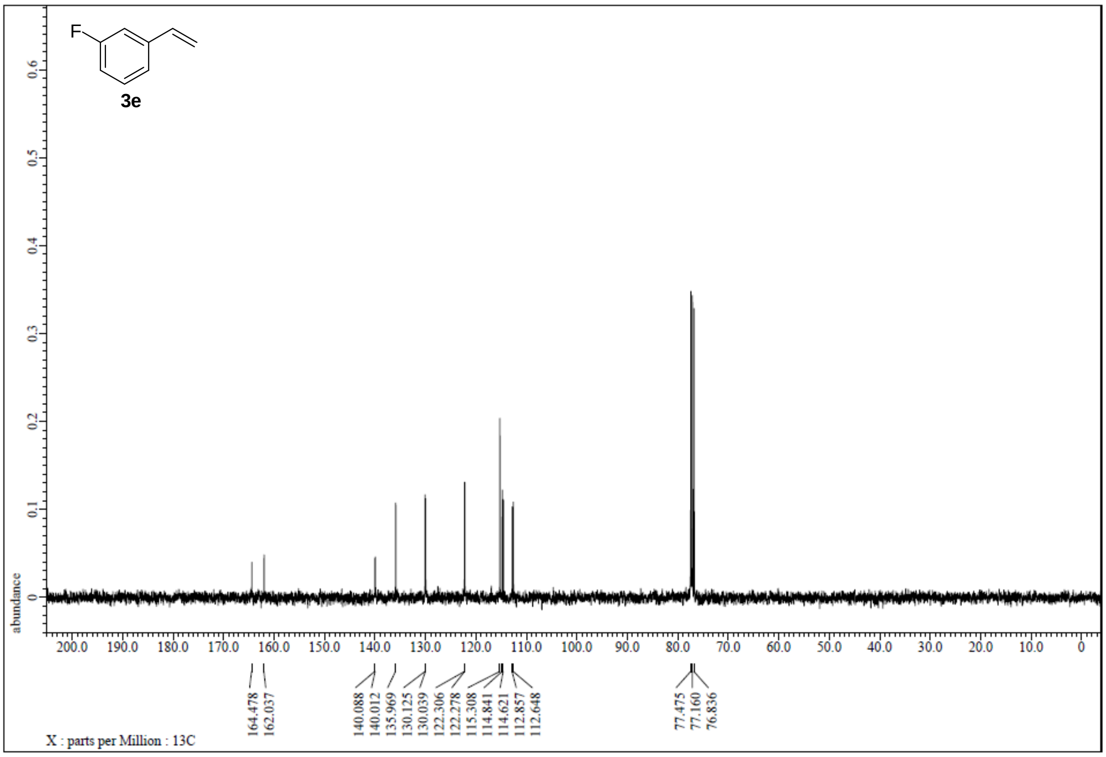

Figure S10. ^{13}C NMR spectrum of 3e, related to Figure 2.

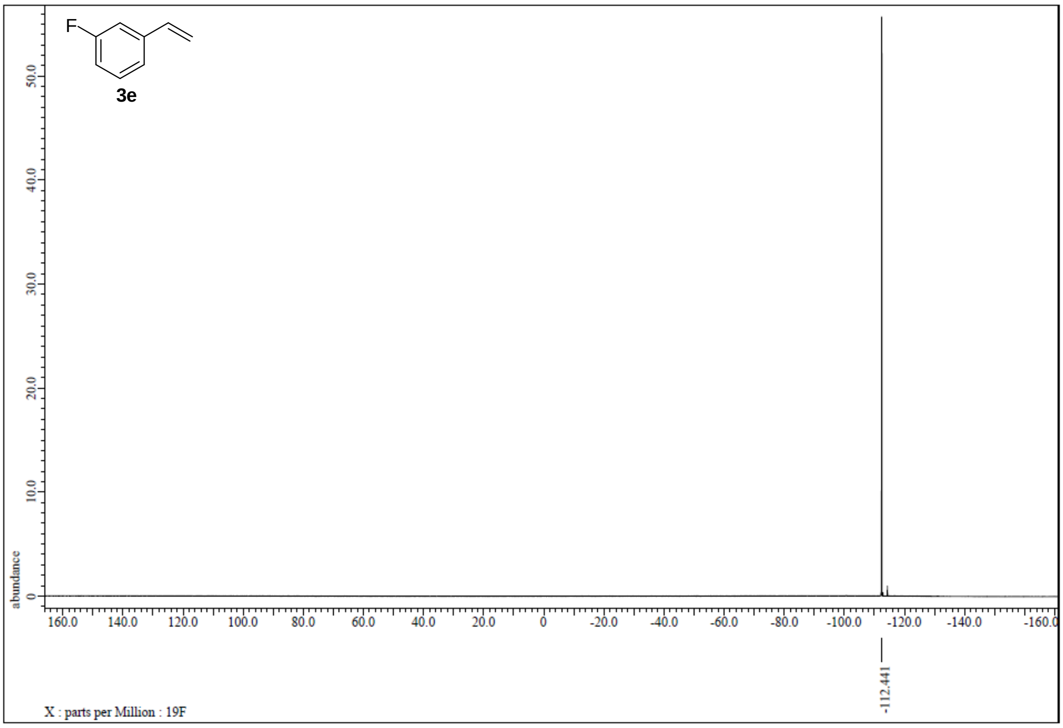

Figure S11. ^{19}F NMR spectrum of 3e, related to Figure 2.

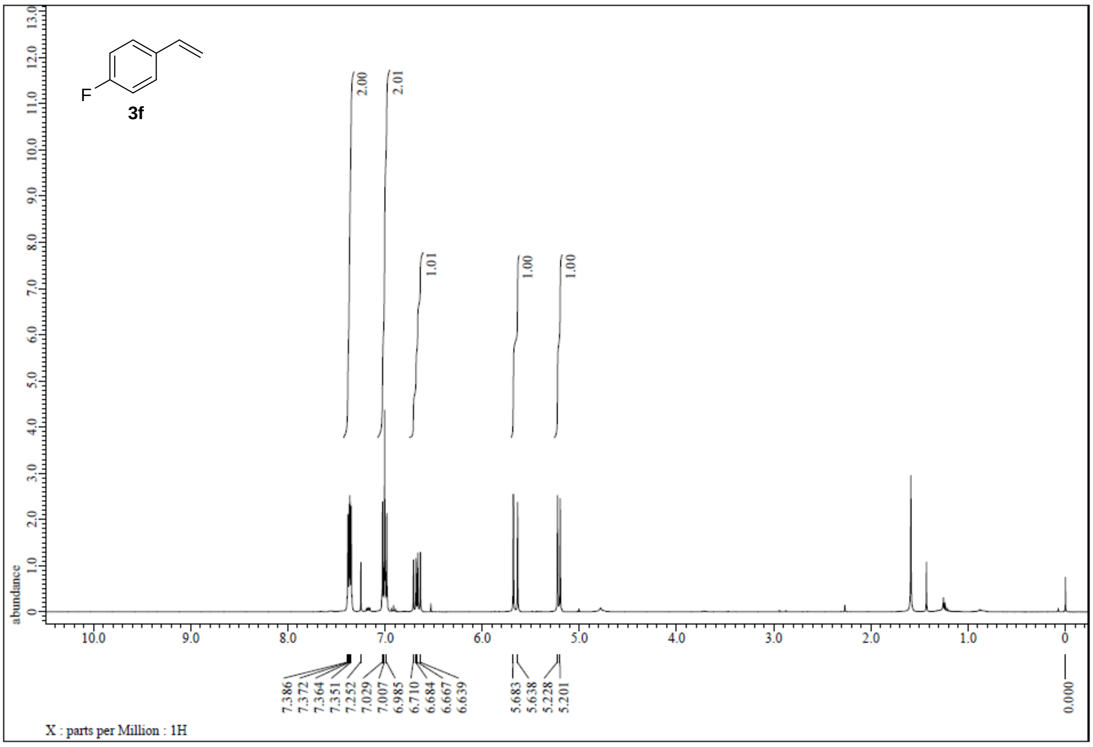

Figure S12. ^{1}H NMR spectrum of 3f, related to Figure 2.

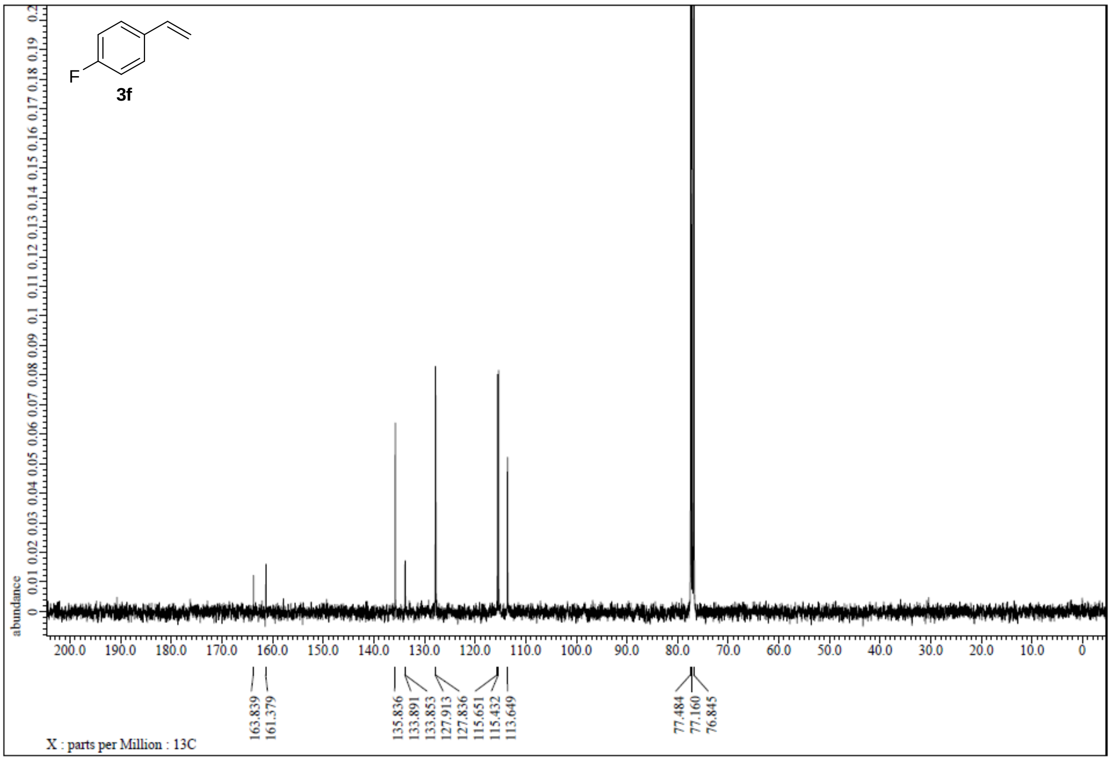

Figure S13. ^{13}C NMR spectrum of 3f, related to Figure 2.

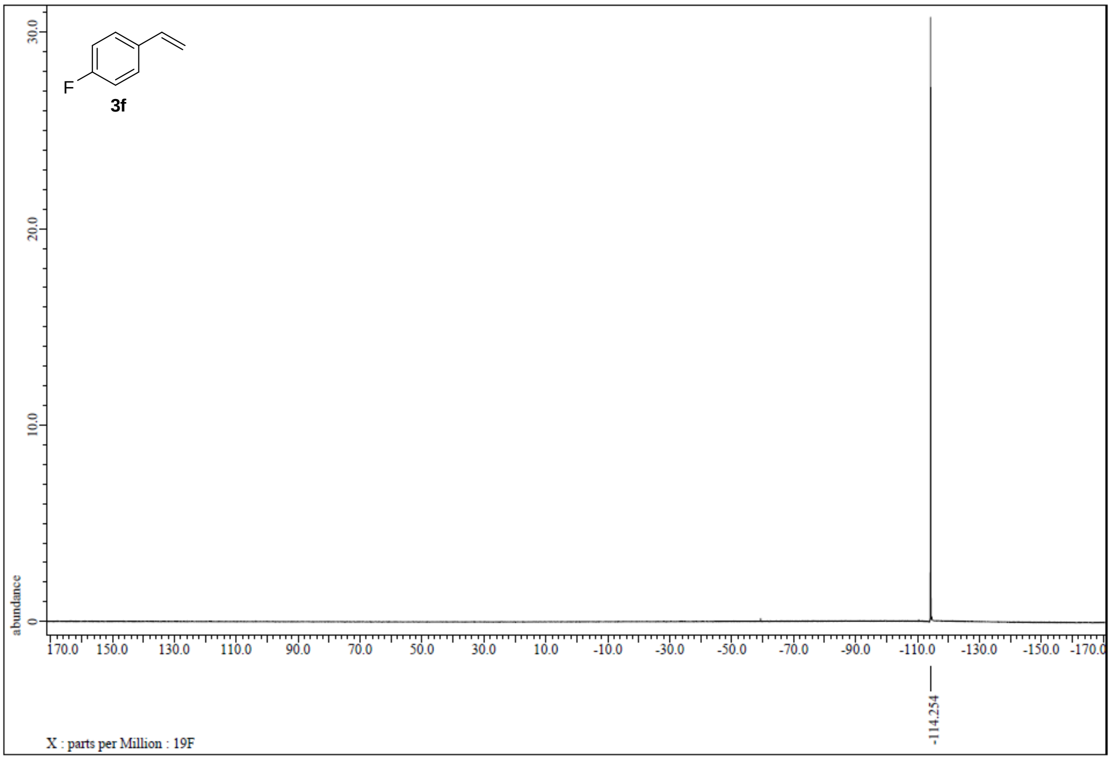

Figure S14. ^{19}F NMR spectrum of 3e, related to Figure 2.

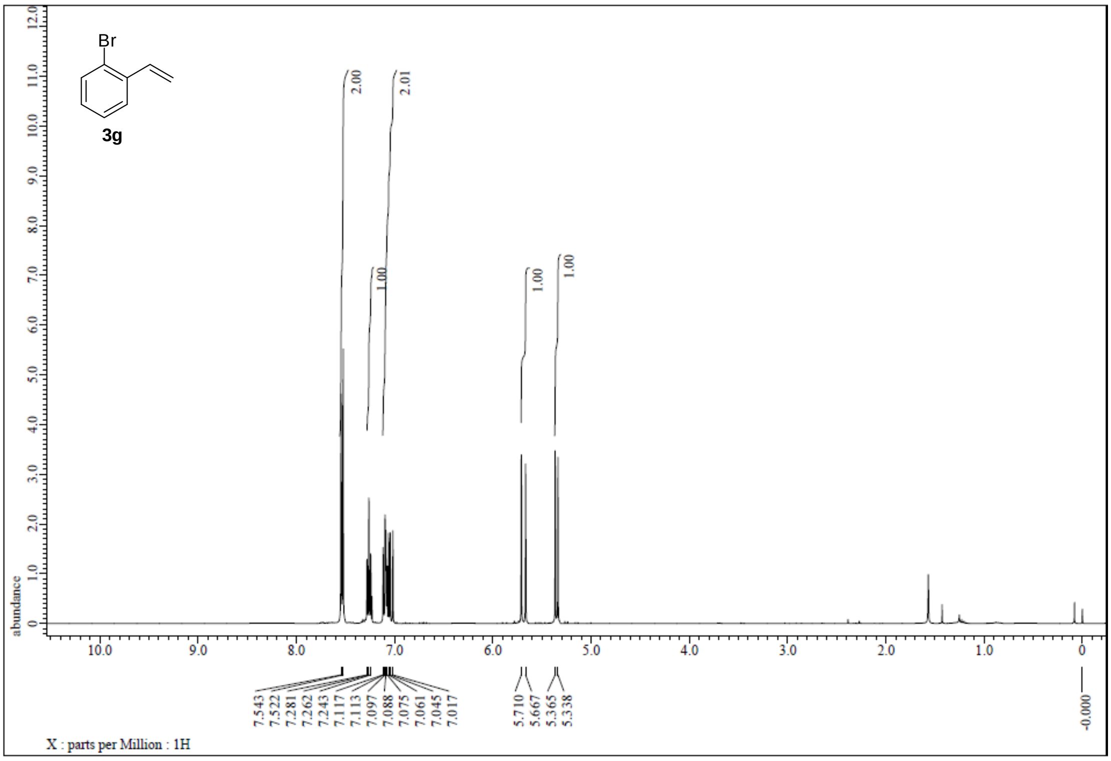

Figure S15. ^{1}H NMR spectrum of 3g, related to Figure 2.

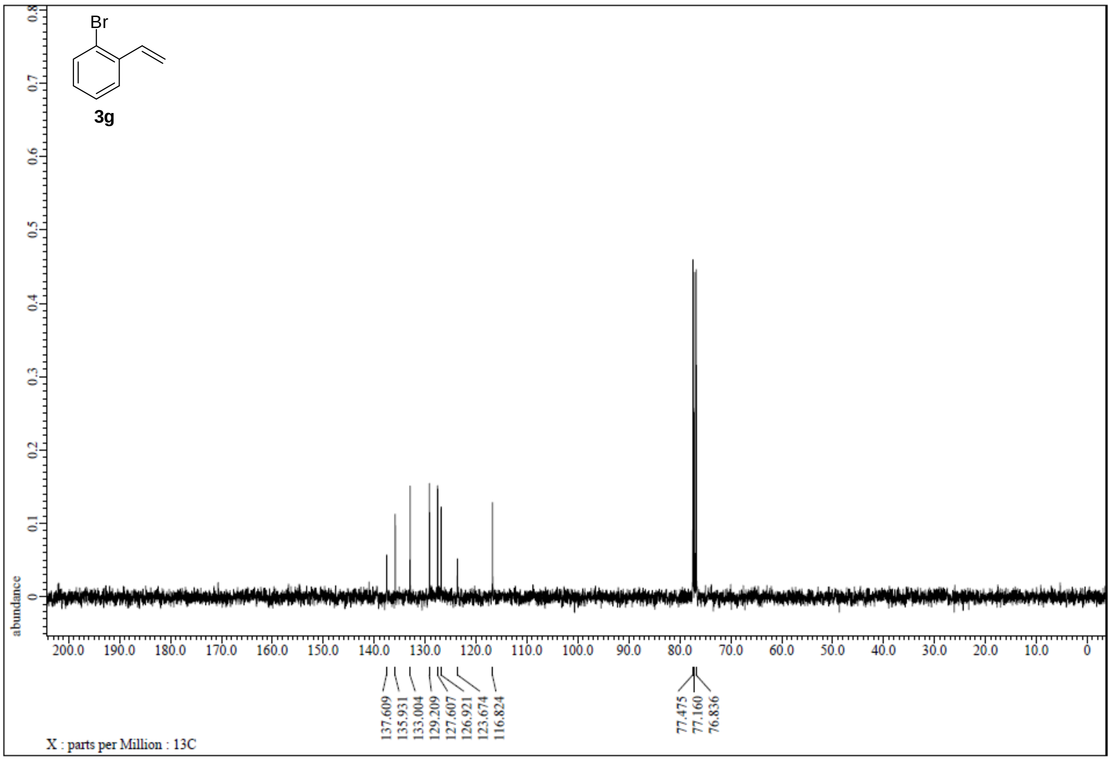

Figure S16. ^{13}C NMR spectrum of 3g, related to Figure 2.

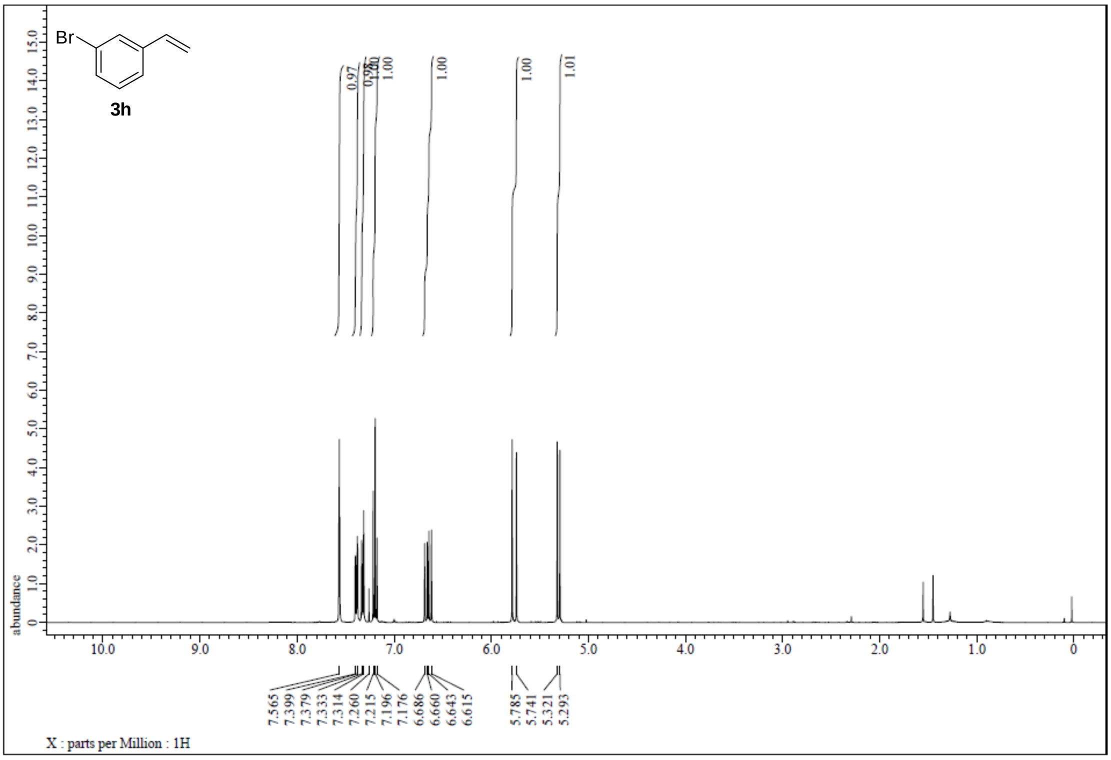

Figure S17. **^{1}H NMR spectrum of 3h, related to Figure 2**.

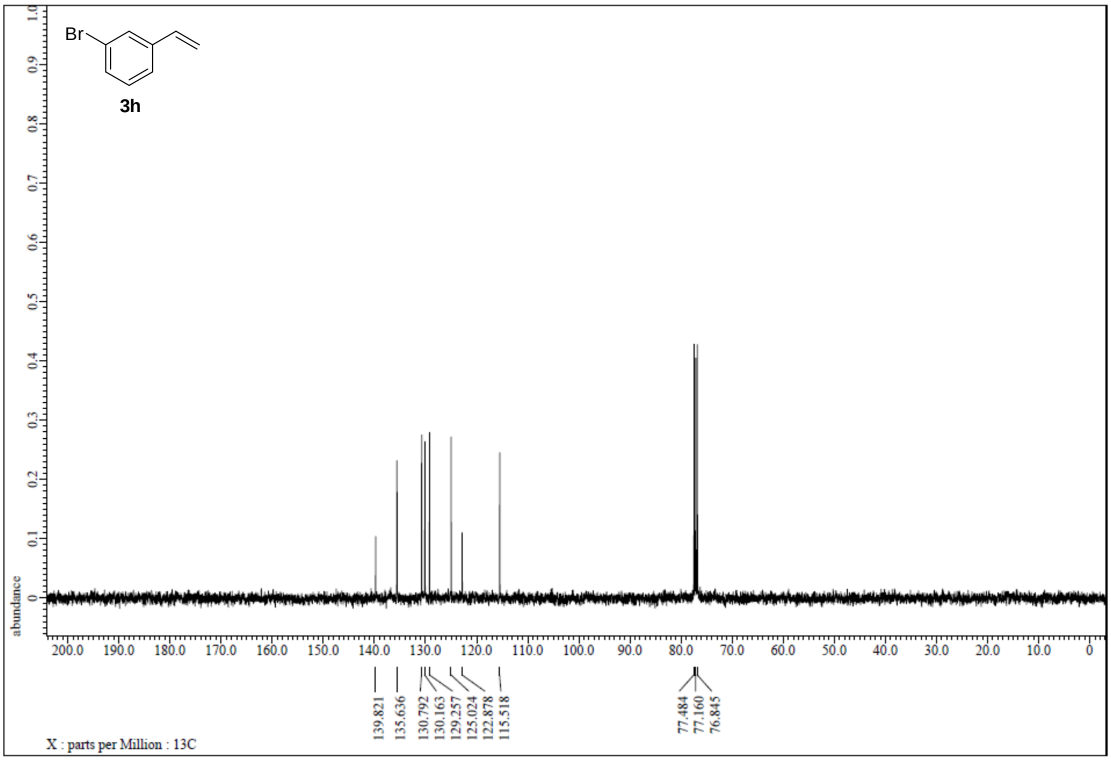

Figure S18. **^{13}C NMR spectrum of 3h, related to Figure 2**.

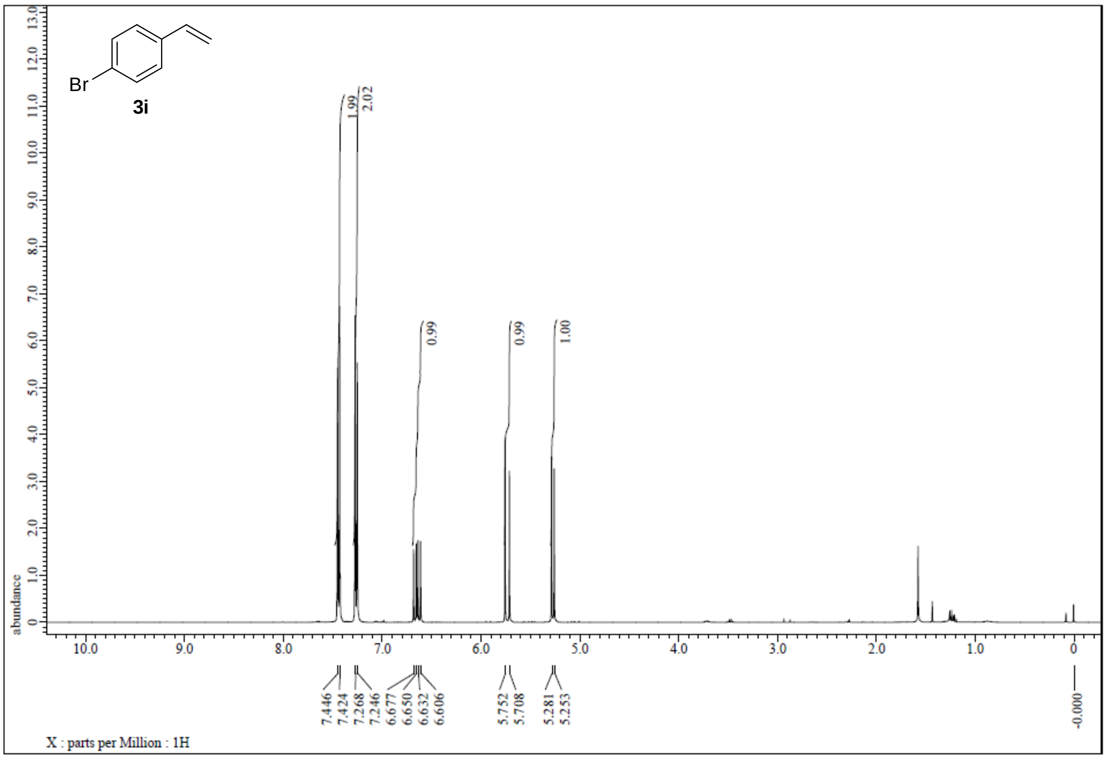

Figure S19. ^{1}H NMR spectrum of 3i, related to Figure 2.

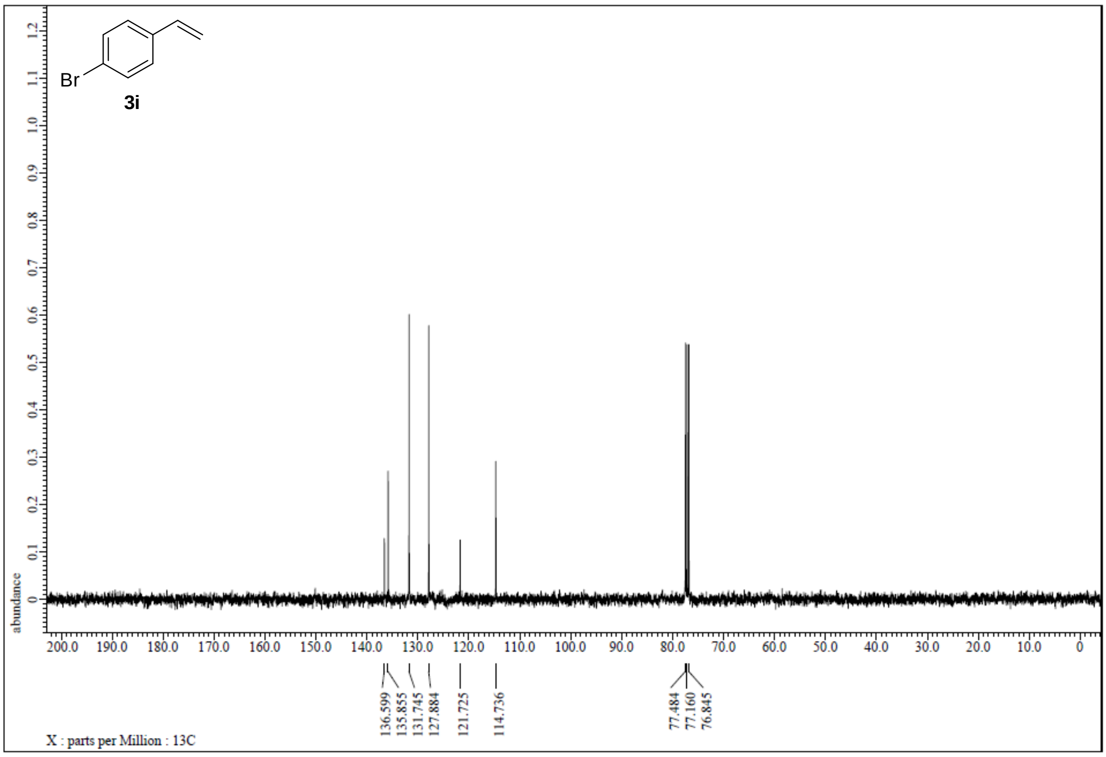

Figure S20. ^{13}C NMR spectrum of 3i, related to Figure 2.

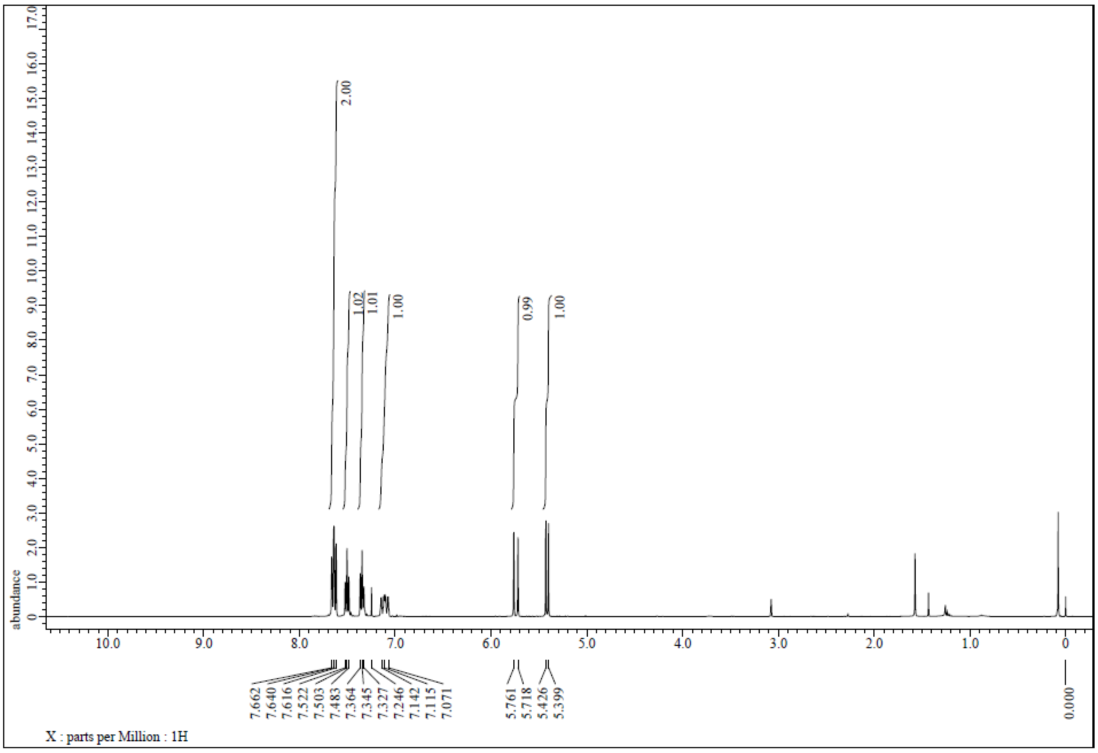

Figure S21. ^{1}H NMR spectrum of 3j, related to Figure 2.

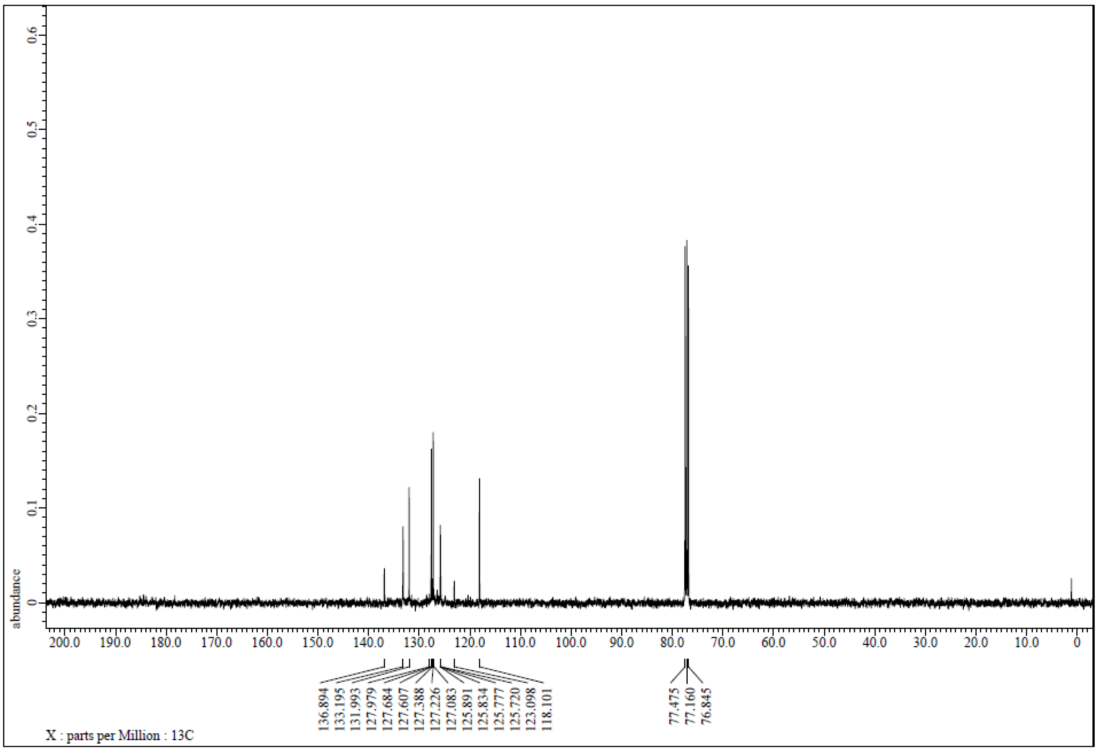

Figure S22. ^{13}C NMR spectrum of 3j, related to Figure 2.

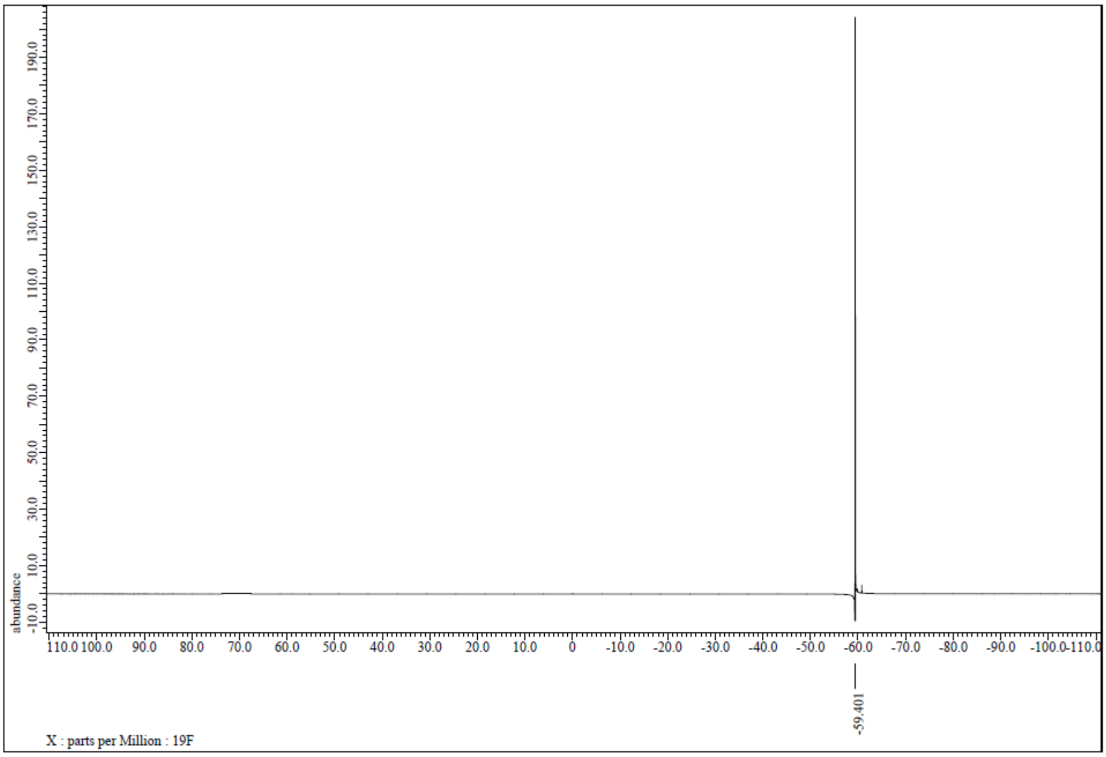

Figure S23. ^{19}F NMR spectrum of 3j, related to Figure 2.

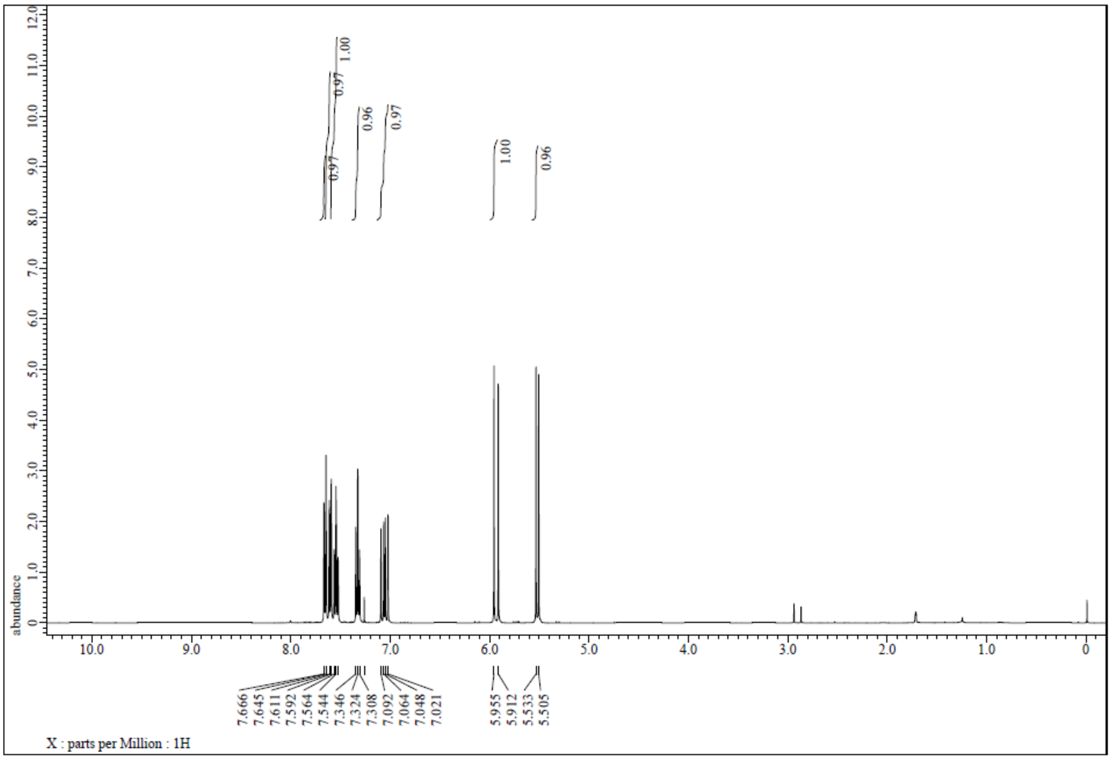

Figure S24. ^{1}H NMR spectrum of 3k, related to Figure 2.

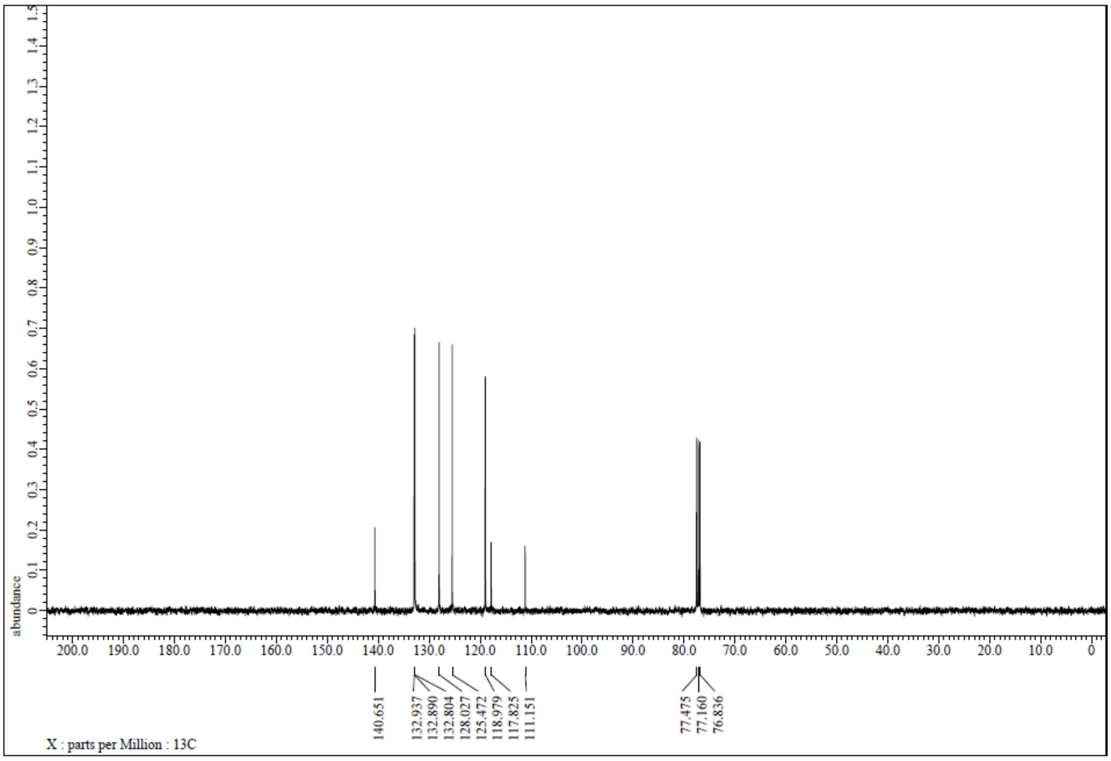

Figure S25. ^{13}C NMR spectrum of 3k, related to Figure 2.

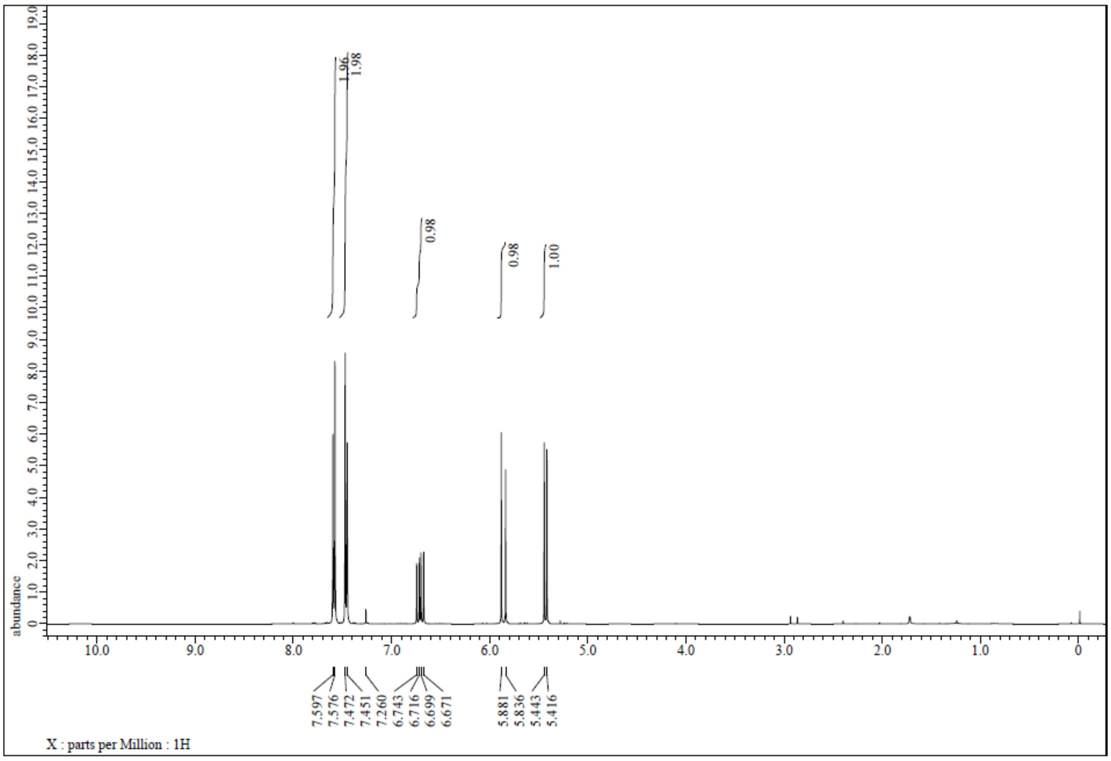

Figure S26. **^{1}H NMR spectrum of 3l, related to Figure 2**.

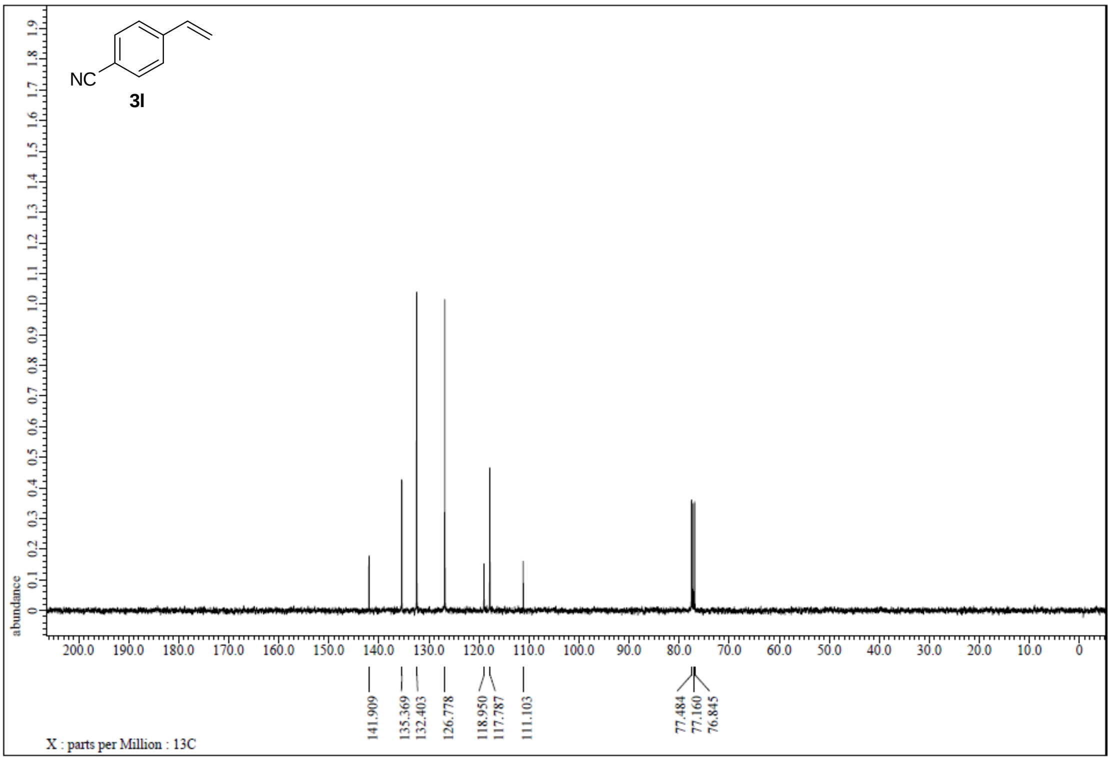

Figure S27. **^{13}C NMR spectrum of 3l, related to Figure 2**.

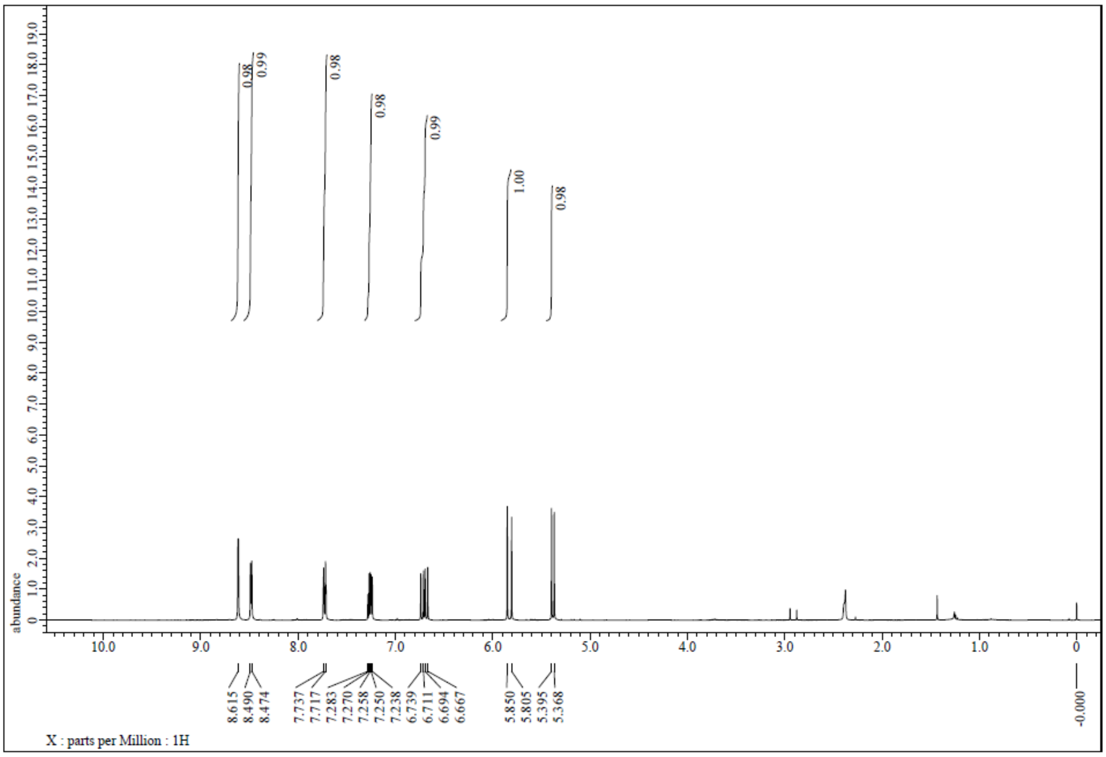

Figure S28. ^{1}**H NMR spectrum of 3m, related to Figure 2**.

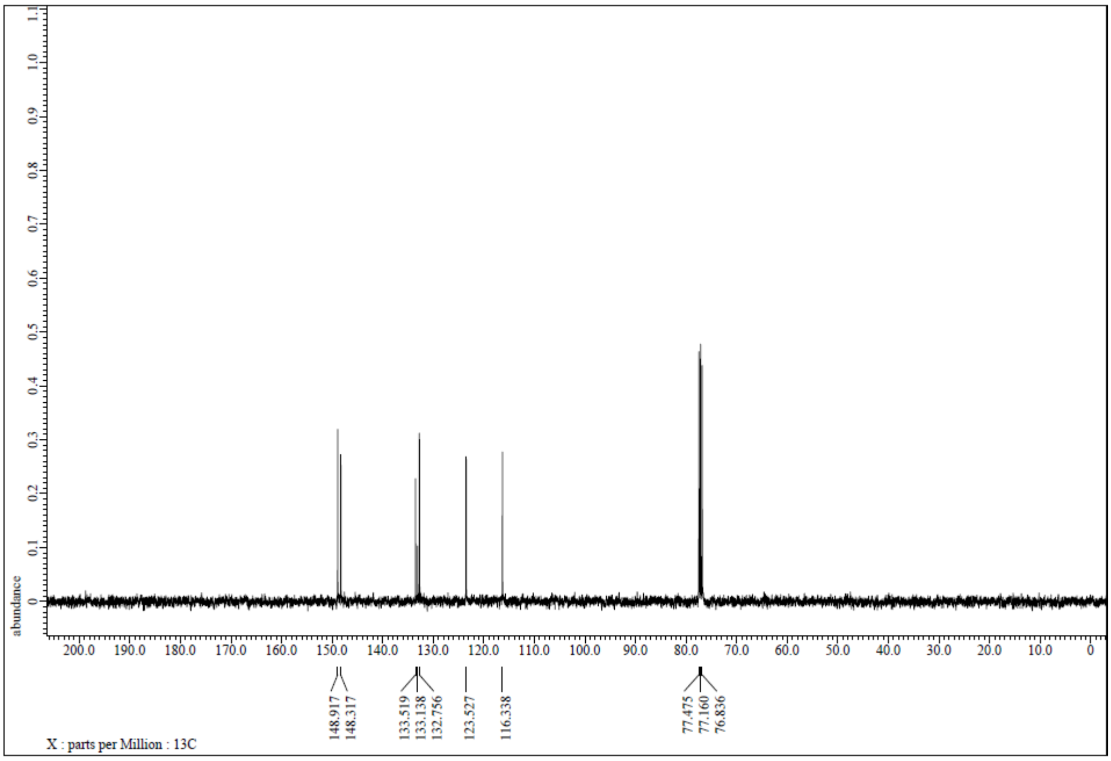

Figure S29. ^{13}**C NMR spectrum of 3m, related to Figure 2**.

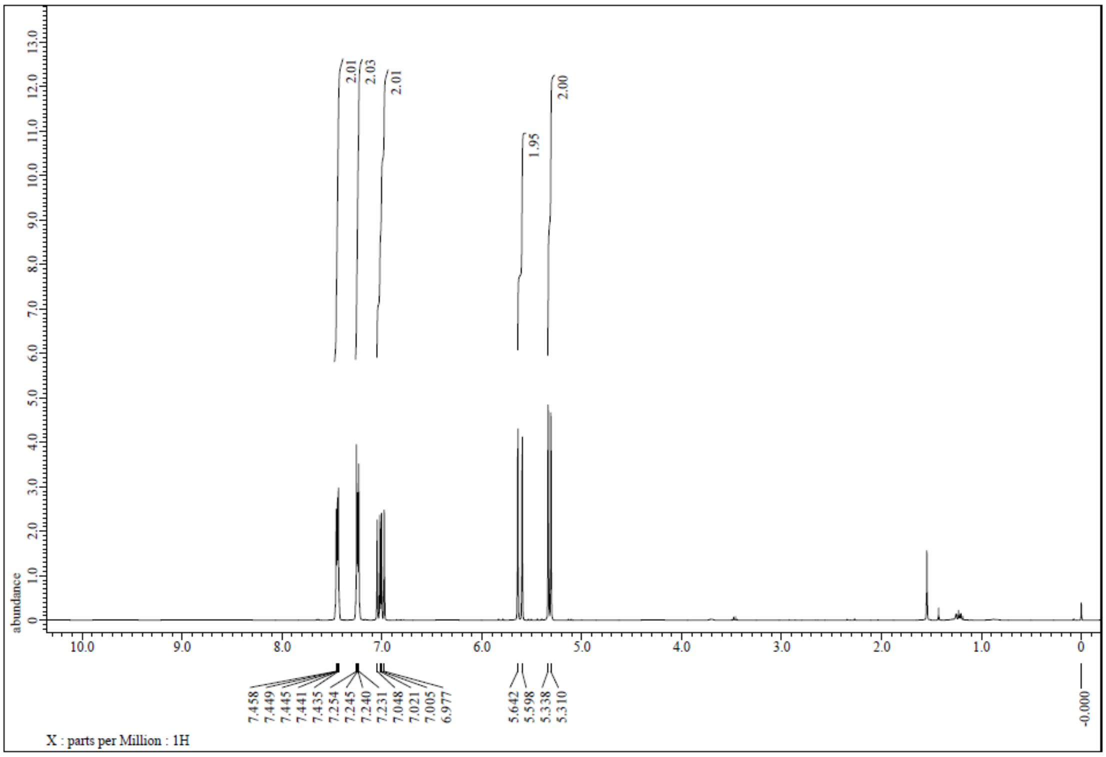

Figure S30. ^{1}H NMR spectrum of 3n, related to Figure 2.

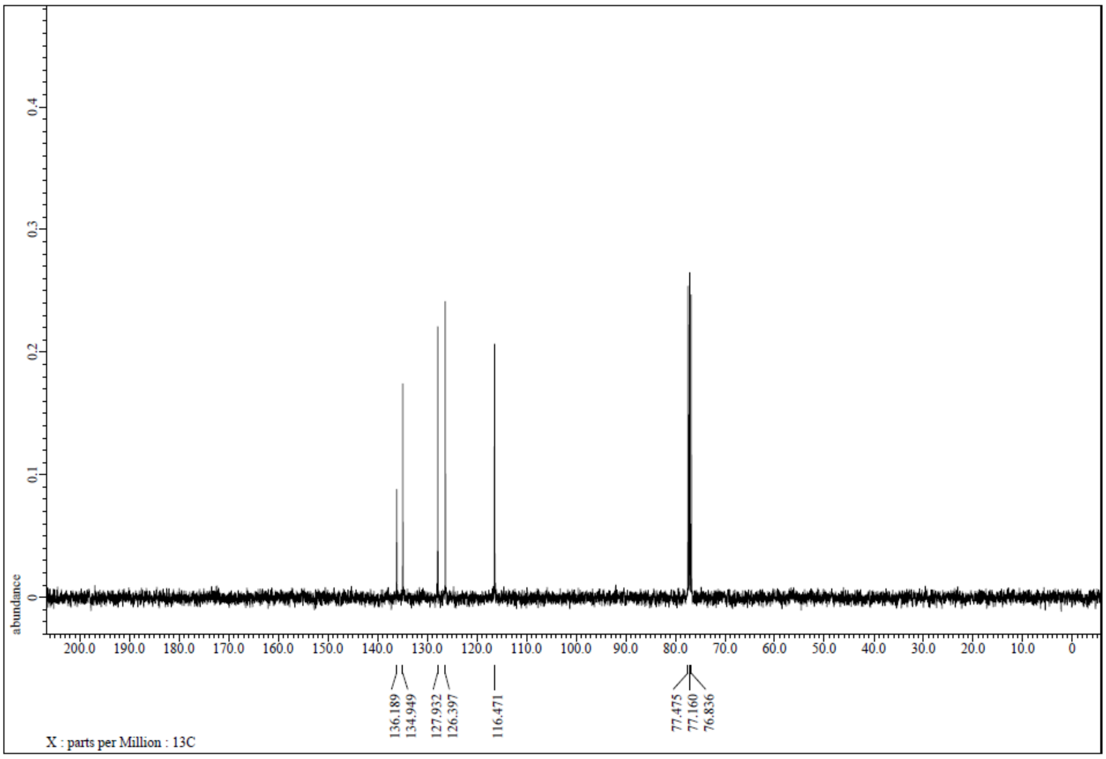

Figure S31. ^{13}C NMR spectrum of 3n, related to Figure 2.

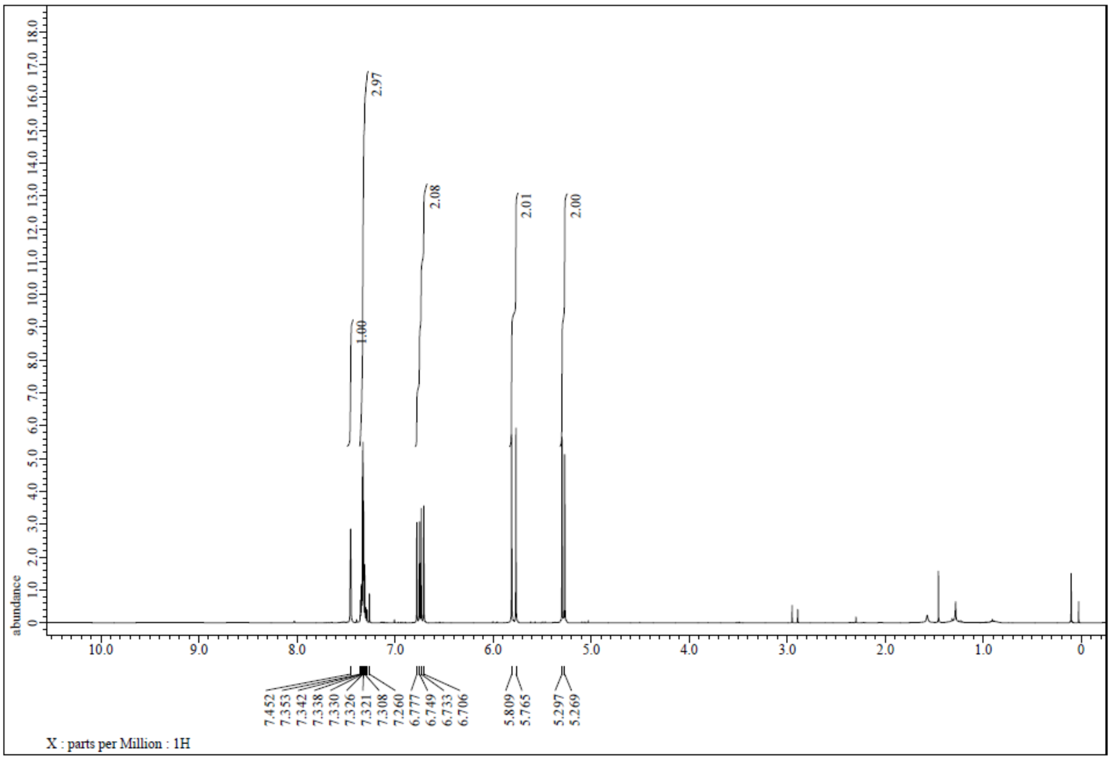

Figure S32. ^{1}H NMR spectrum of 3o, related to Figure 2.

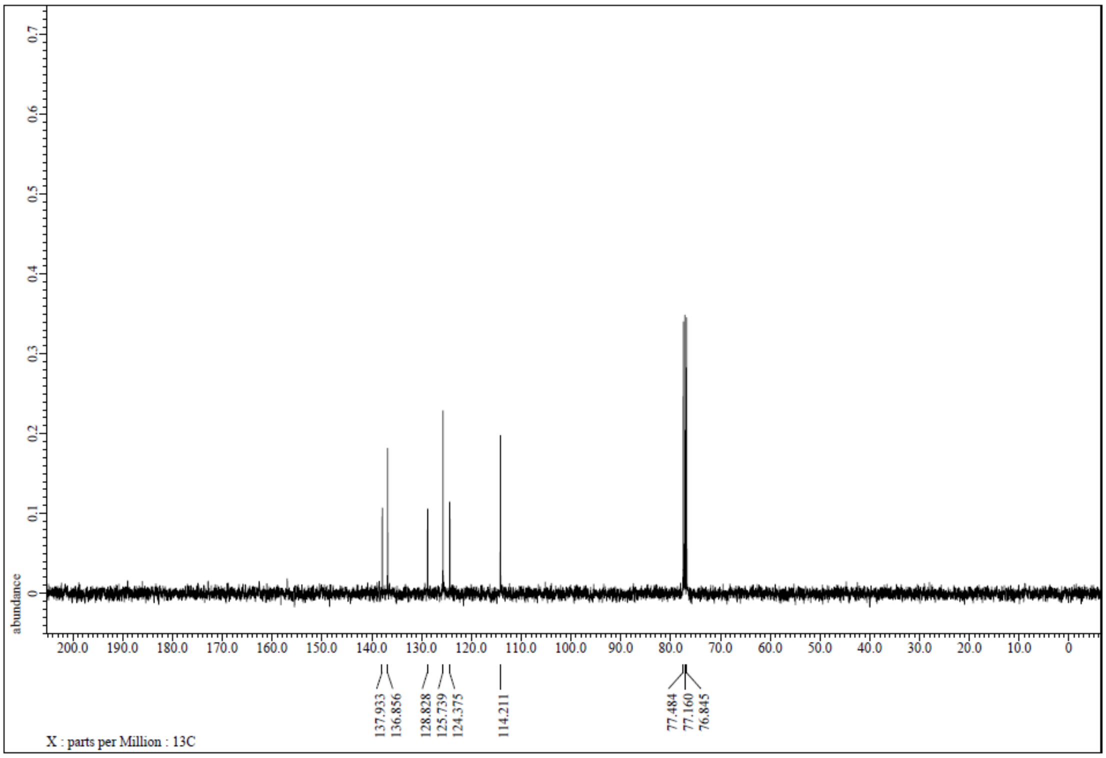

Figure S33. ^{13}C NMR spectrum of 3o, related to Figure 2.

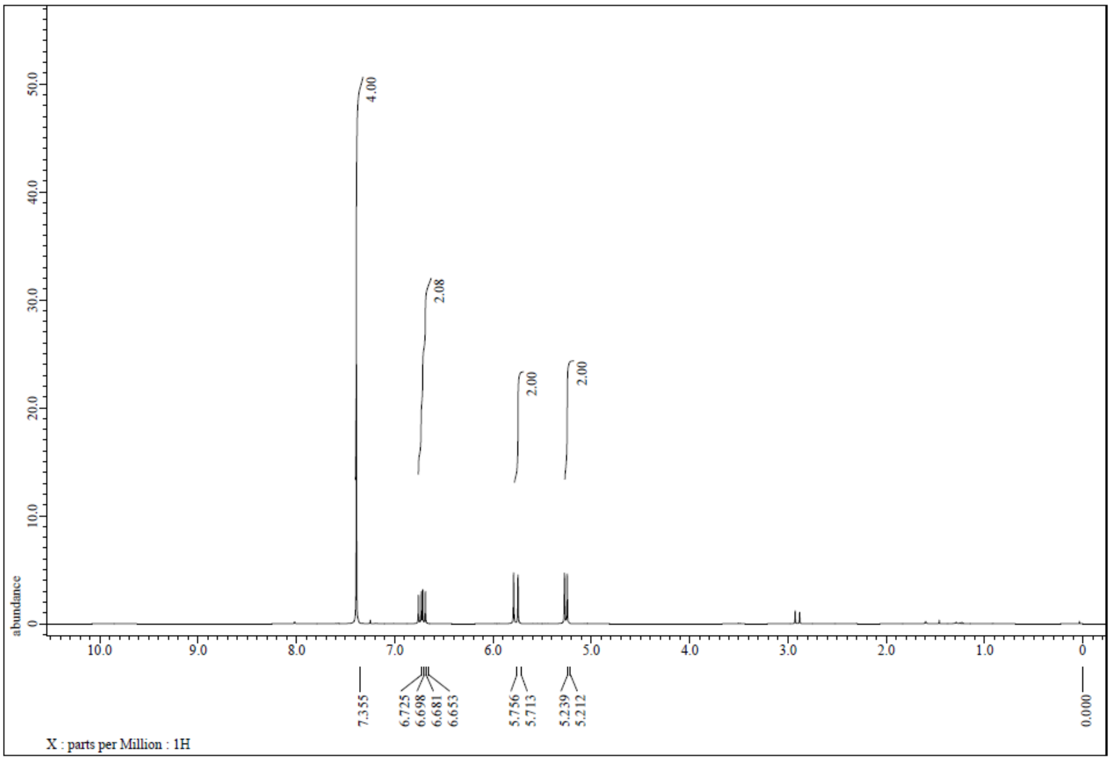

Figure S34. ^{1}H NMR spectrum of 3p, related to Figure 2.

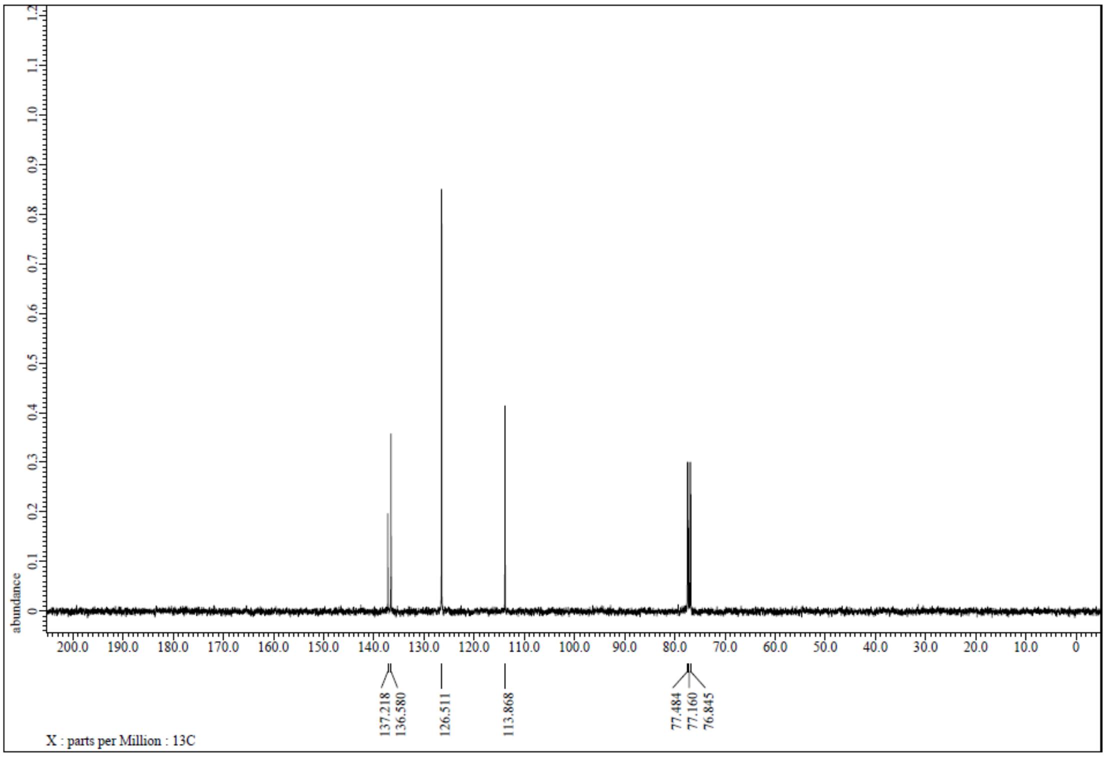

Figure S35. ^{13}C NMR spectrum of 3p, related to Figure 2.

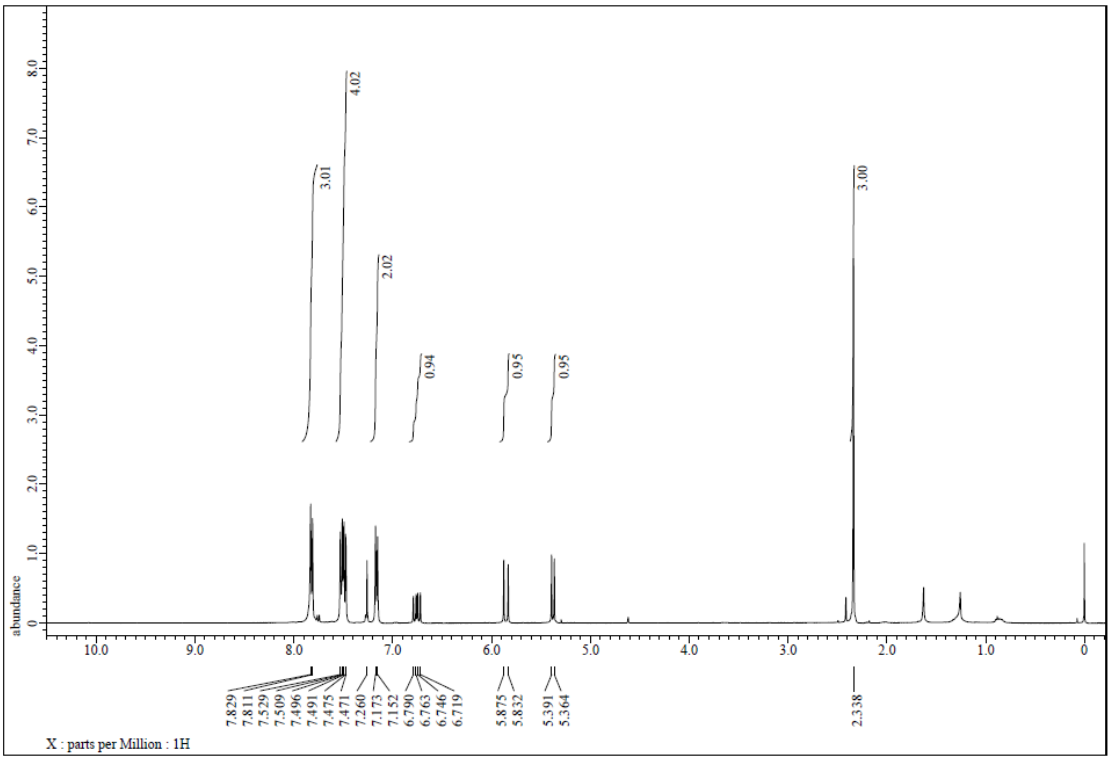

Figure S36. ^{1}H NMR spectrum of 3q, related to Figure 2.

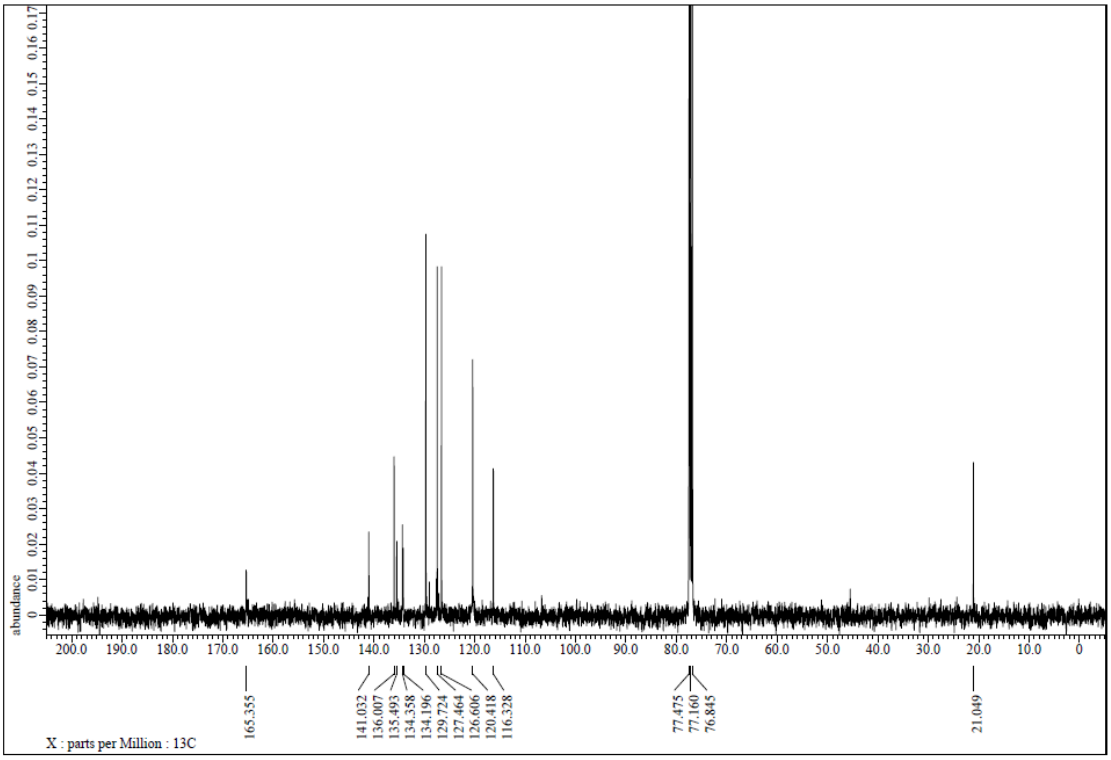

Figure S37. ^{13}C NMR spectrum of 3q, related to Figure 2.

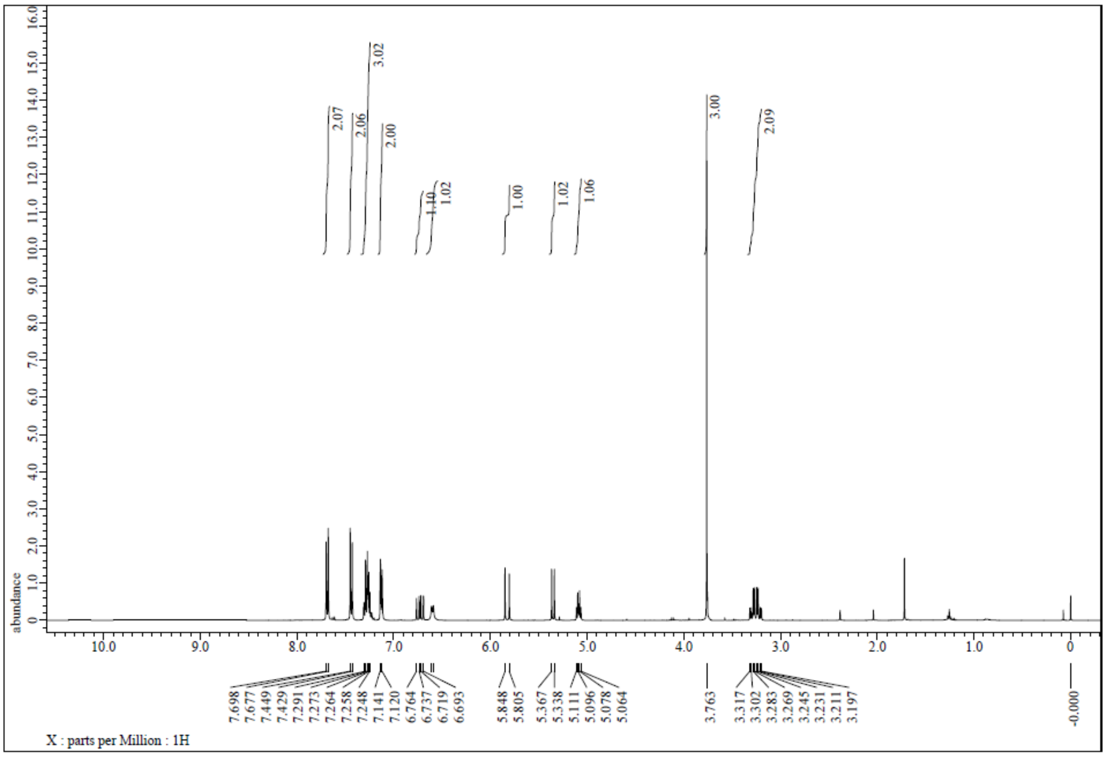

Figure S38. ^{1}H NMR spectrum of 3r, related to Figure 2.

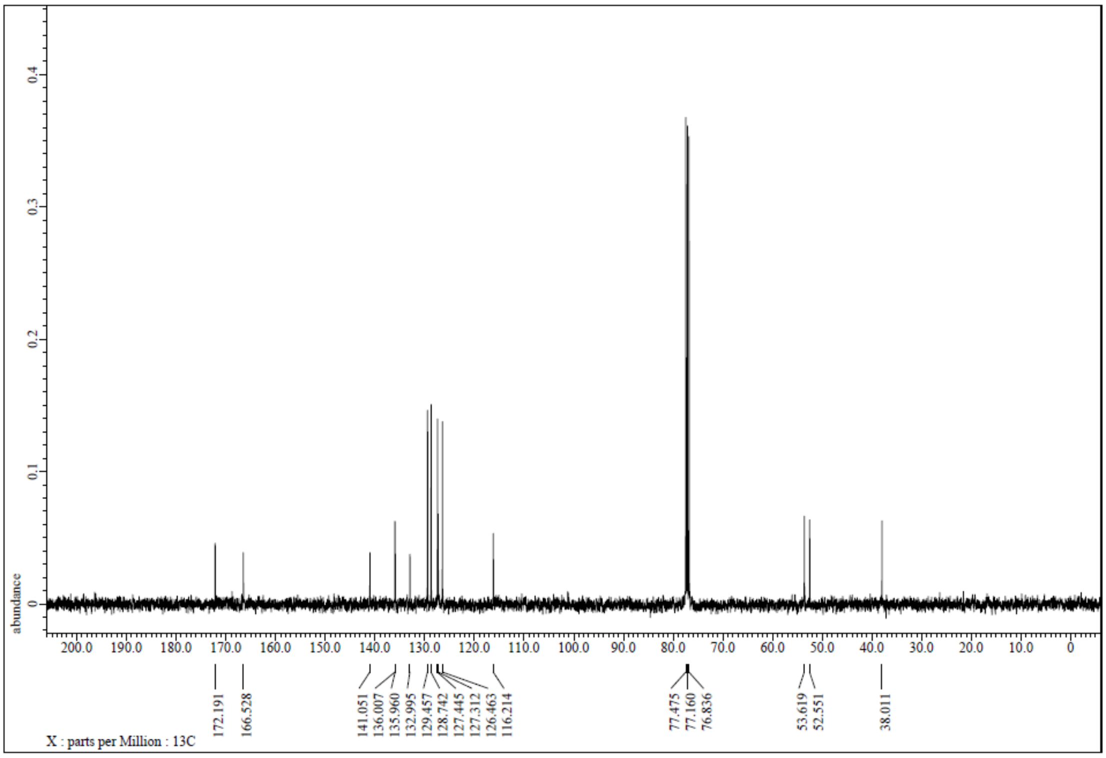

Figure S39. ^{13}C NMR spectrum of 3r, related to Figure 2.

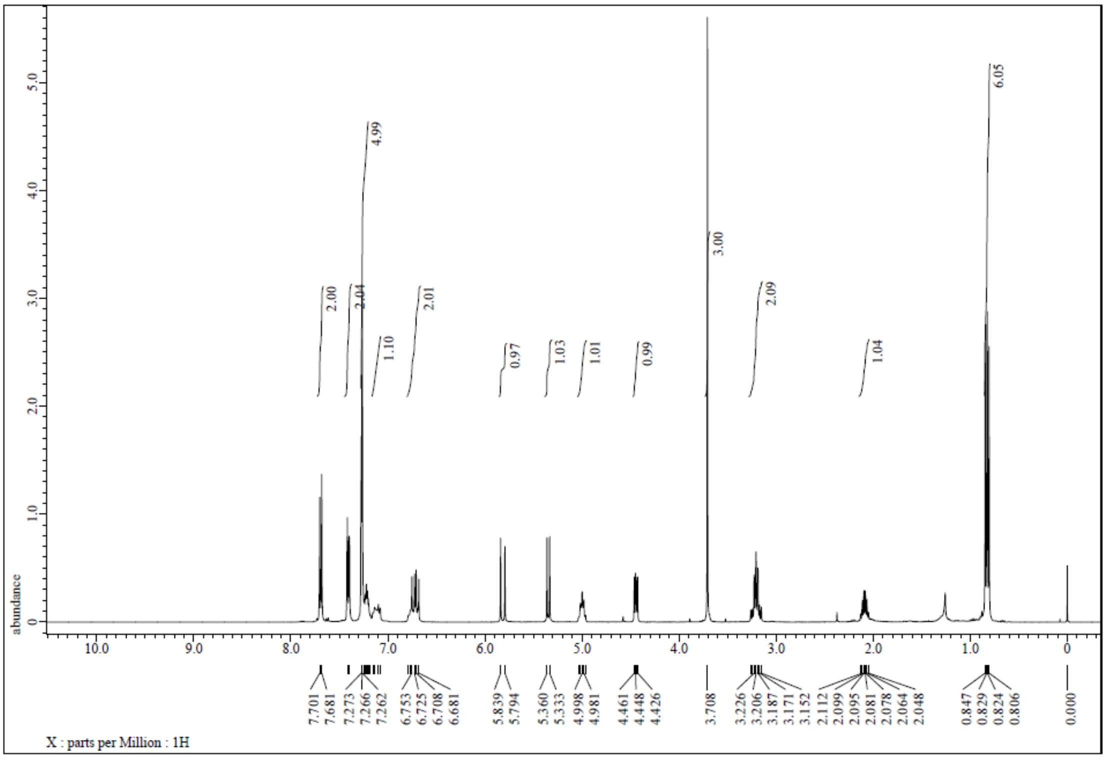

Figure S40. ^{1}H NMR spectrum of 3s, related to Figure 2.

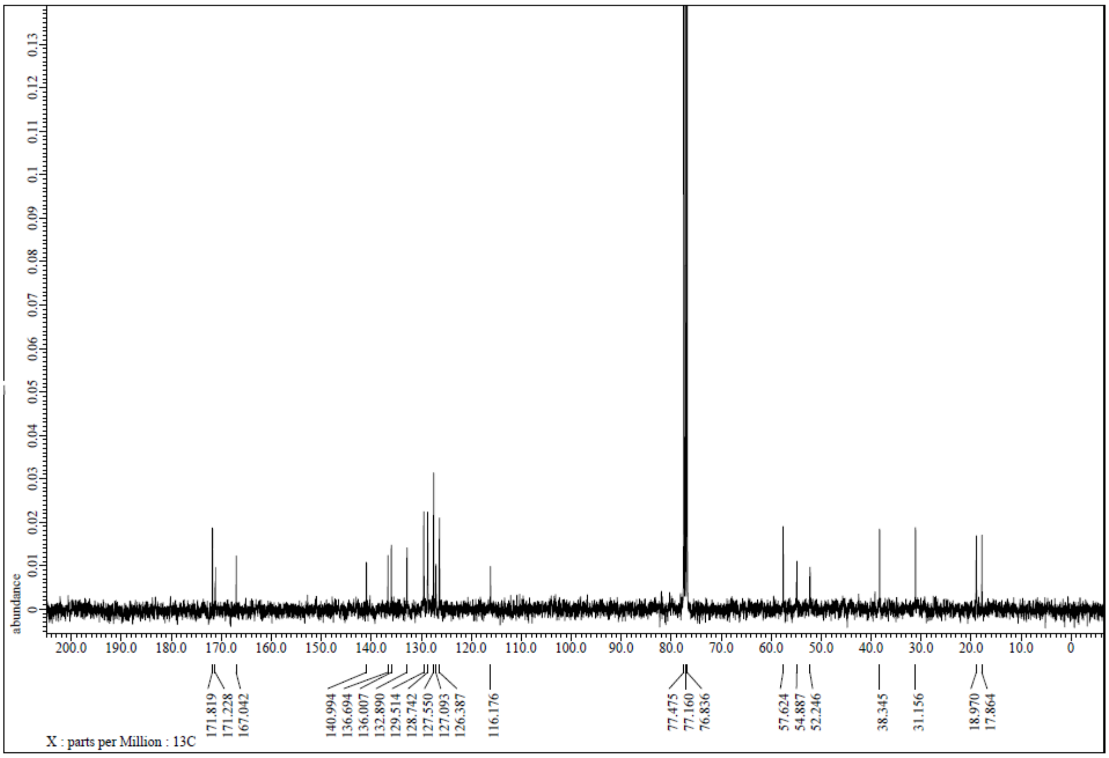

Figure S41. ^{13}C NMR spectrum of 3s, related to Figure 2.

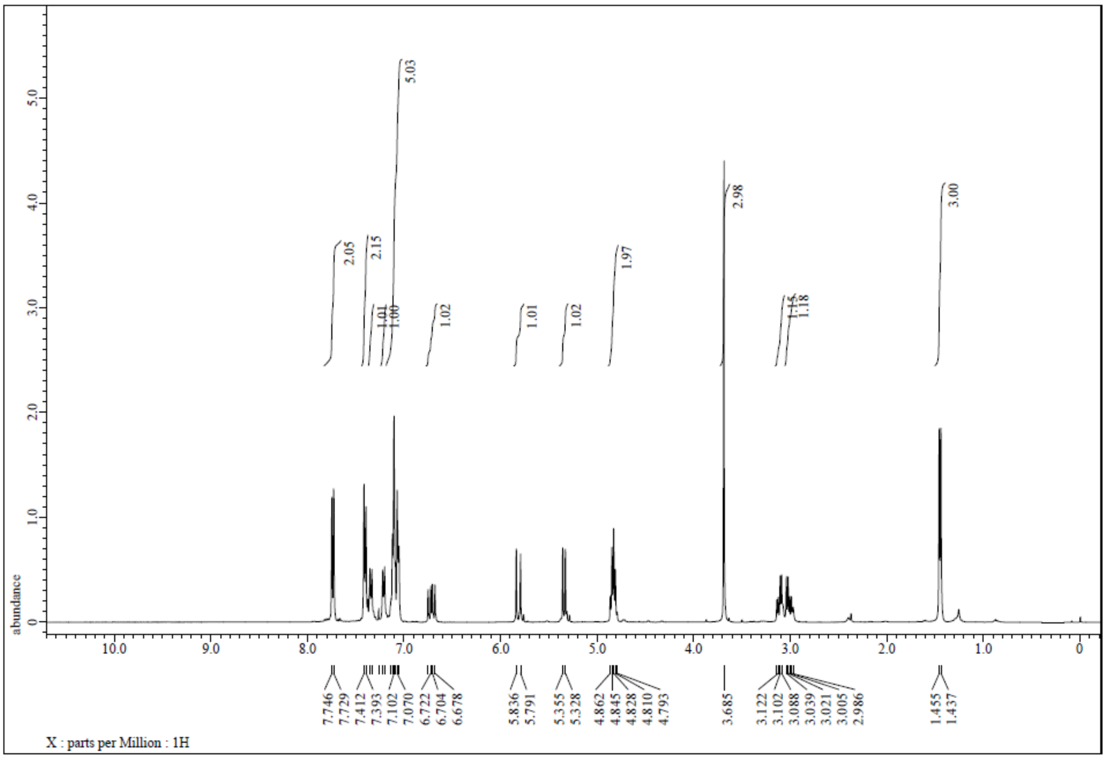

Figure S42. ^{1}H NMR spectrum of 3t, related to Figure 2.

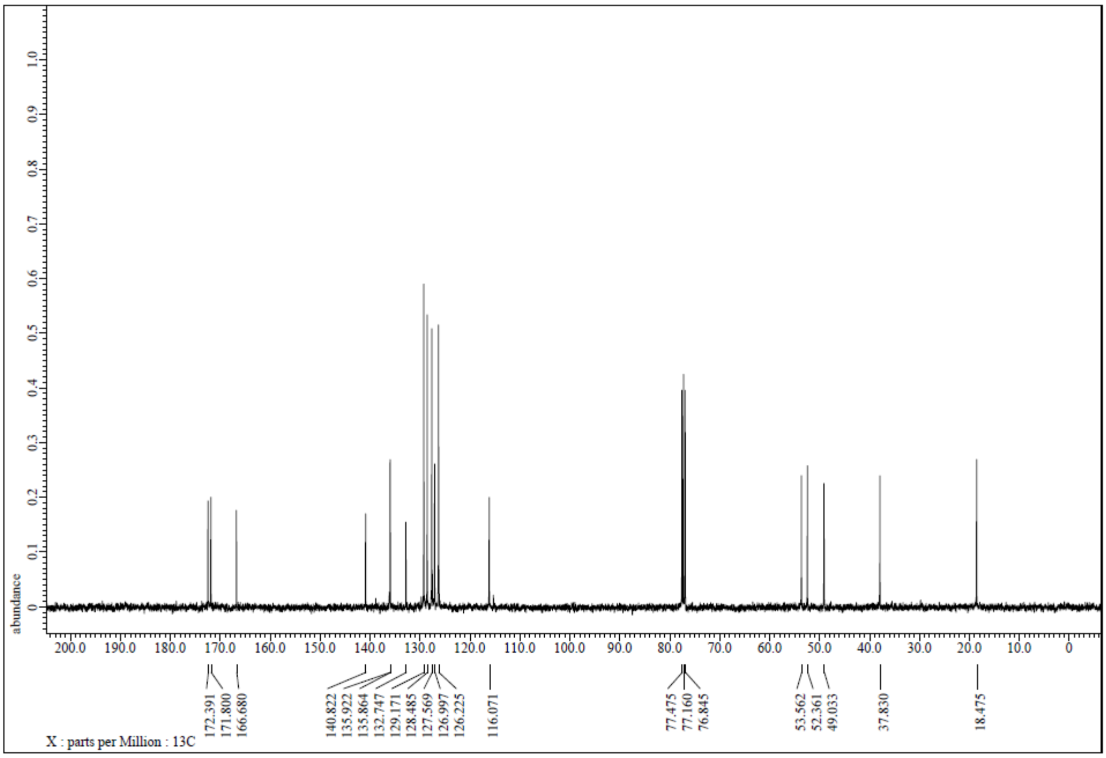

Figure S43. ^{13}C NMR spectrum of 3t, related to Figure 2.

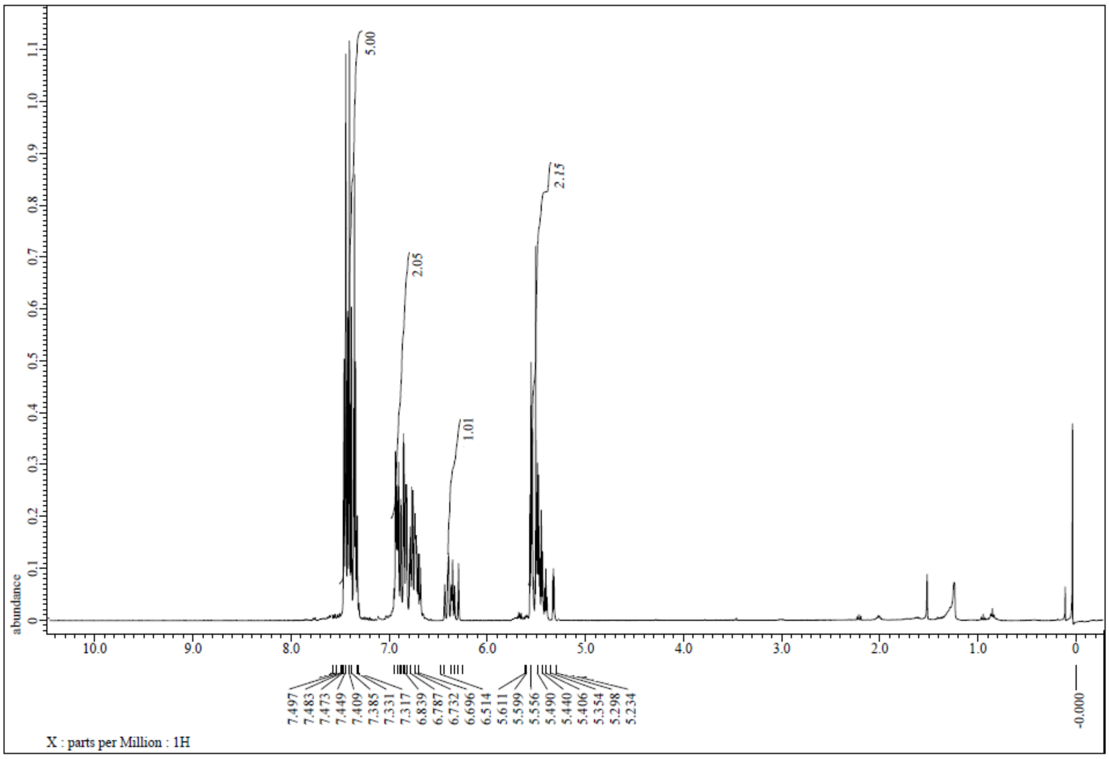

Figure S44. ^{1}H NMR spectrum of 3u, related to Figure 2.

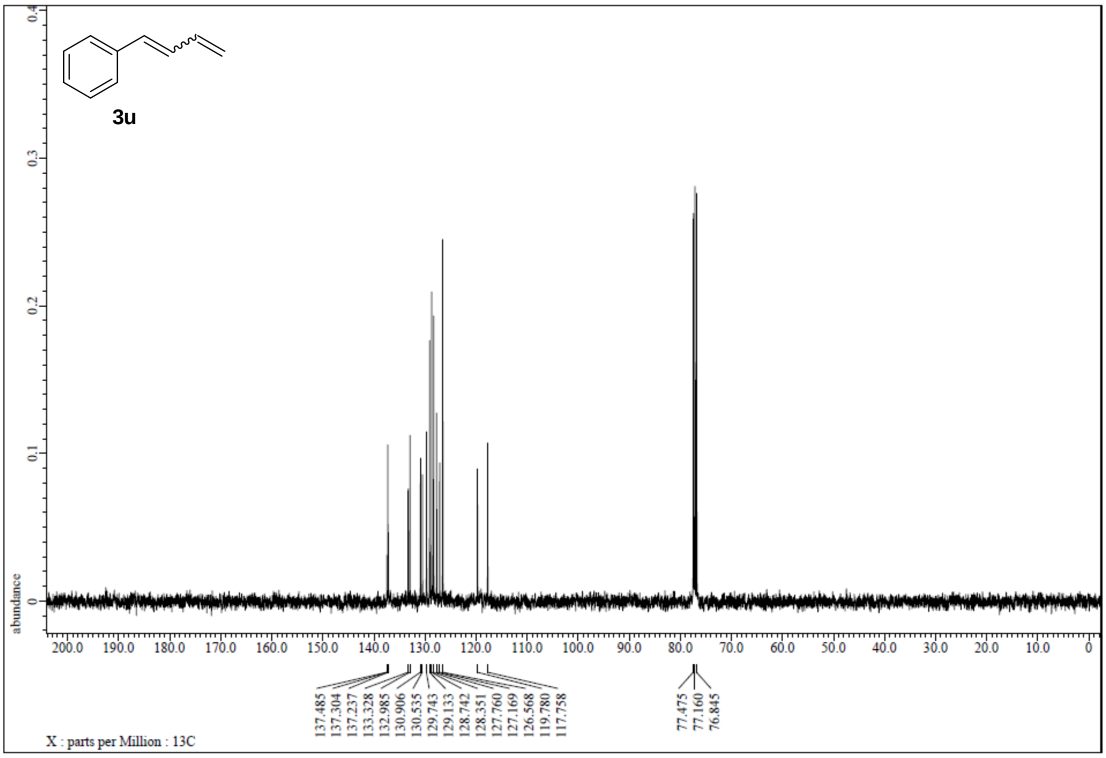

Figure S45. ^{13}C NMR spectrum of 3u, related to Figure 2.

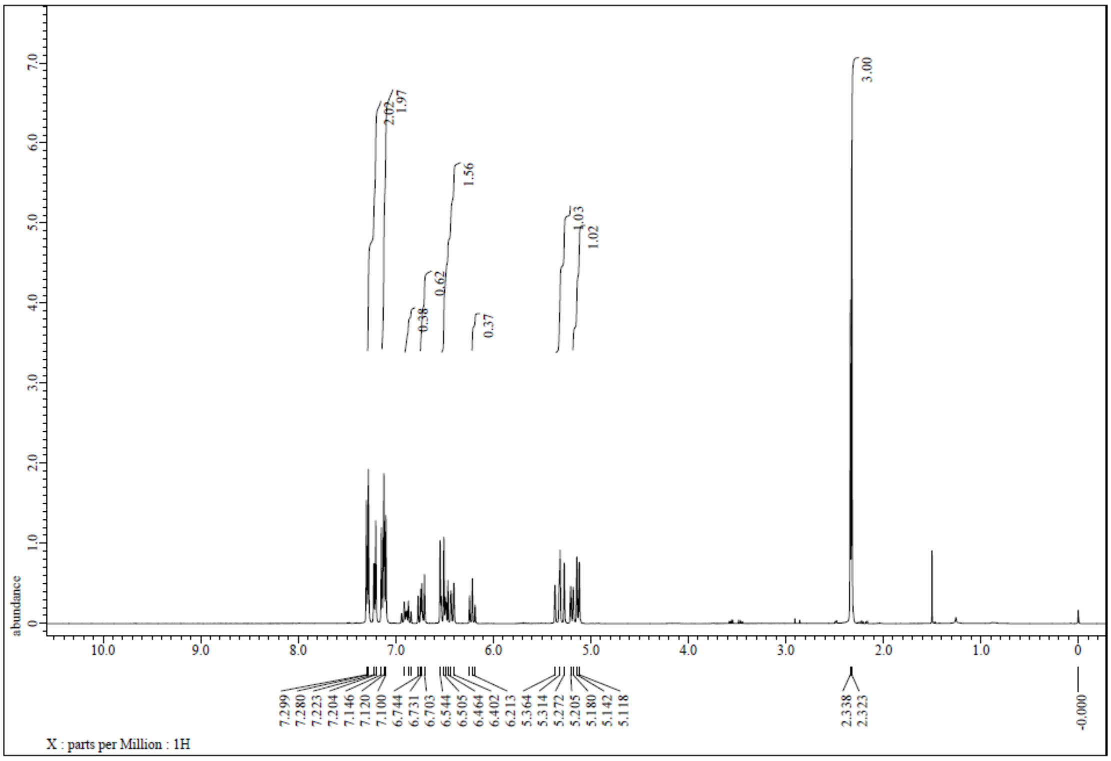

Figure S46. ^{1}**H NMR spectrum of 3v, related to Figure 2**.

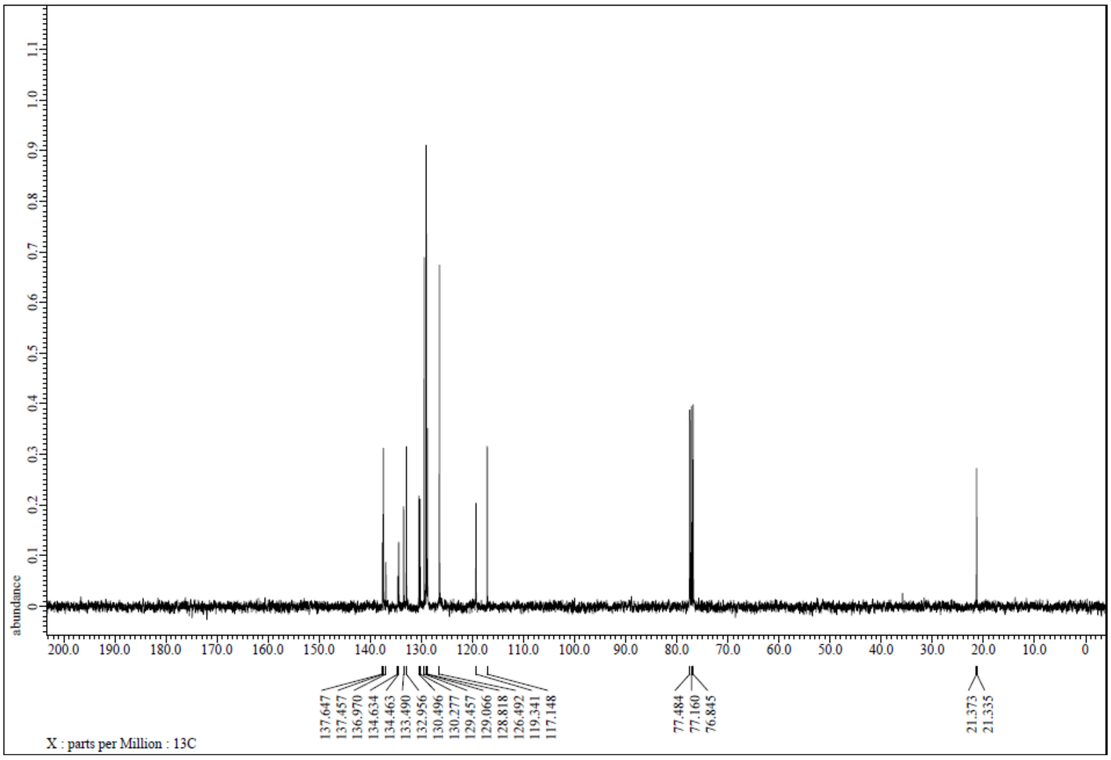

Figure S47. ^{13}**C NMR spectrum of 3v, related to Figure 2**.

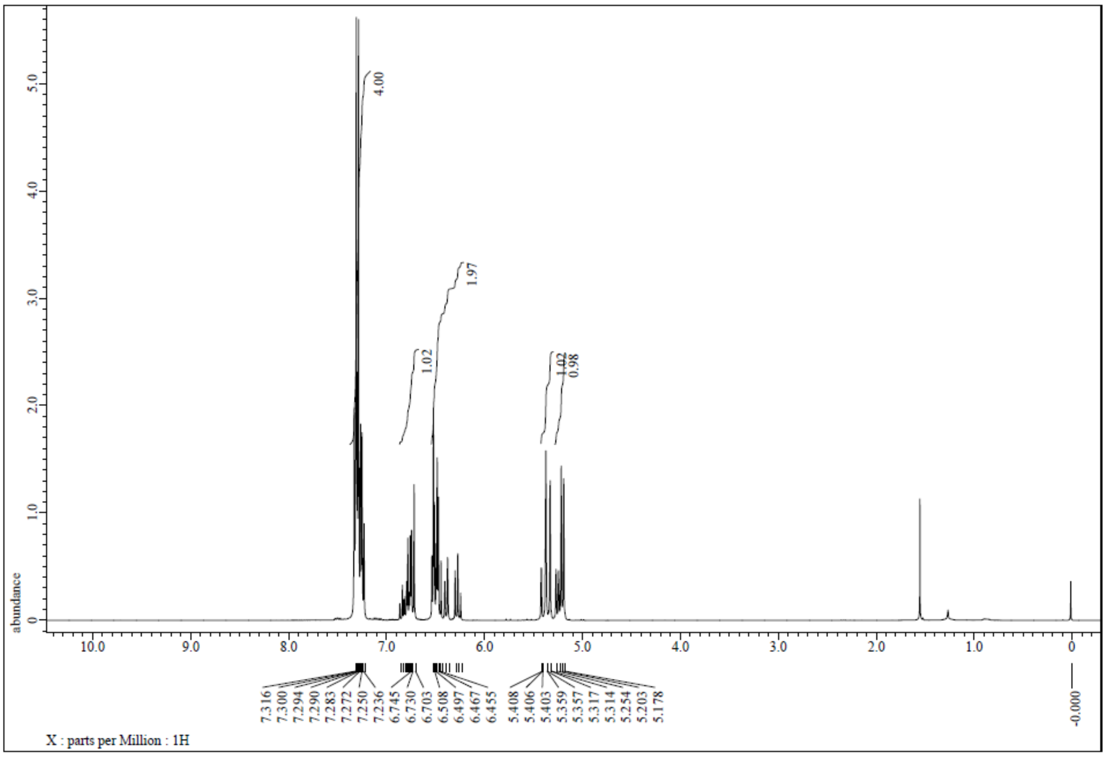

Figure S48. ^{1}H NMR spectrum of 3w, related to Figure 2.

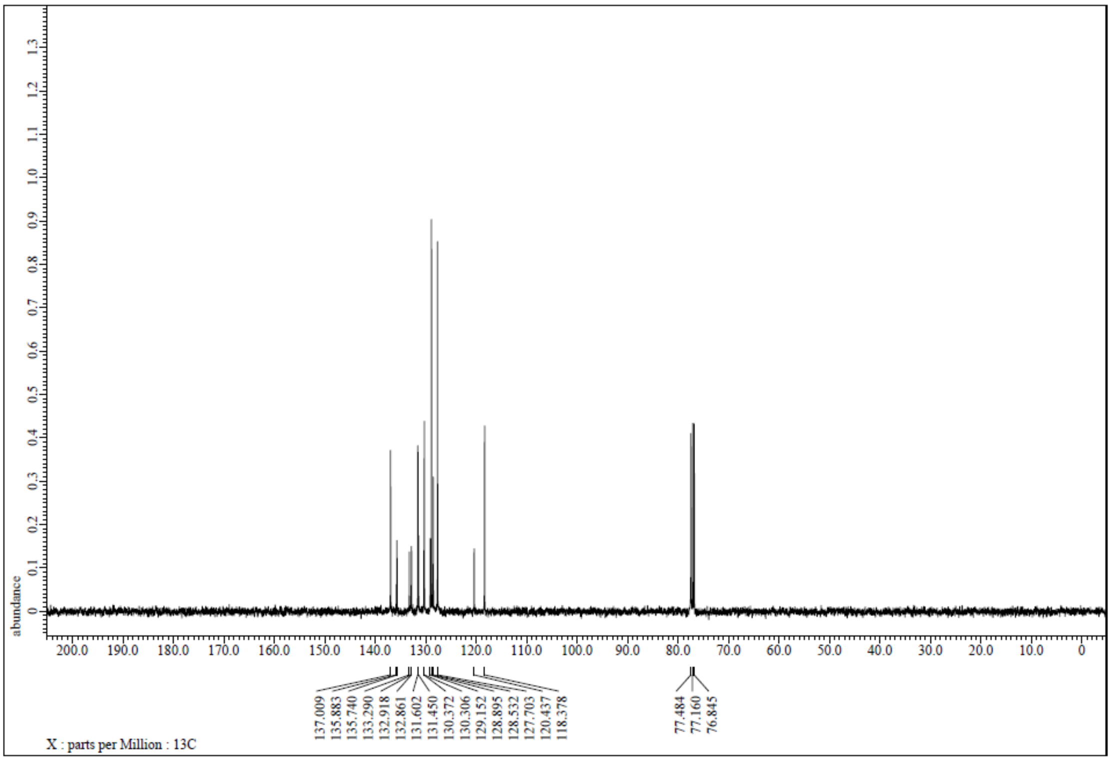

Figure S49. ^{13}C NMR spectrum of 3w, related to Figure 2.

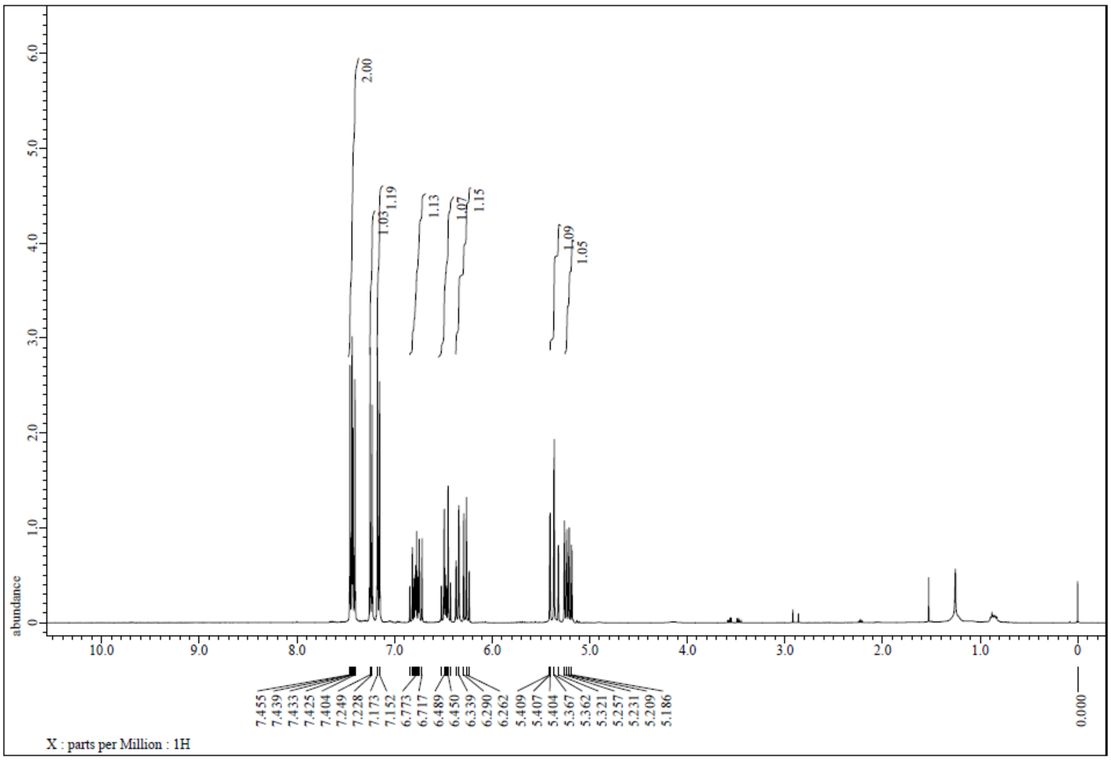

Figure S50. ^{1}H NMR spectrum of 3x, related to Figure 2.

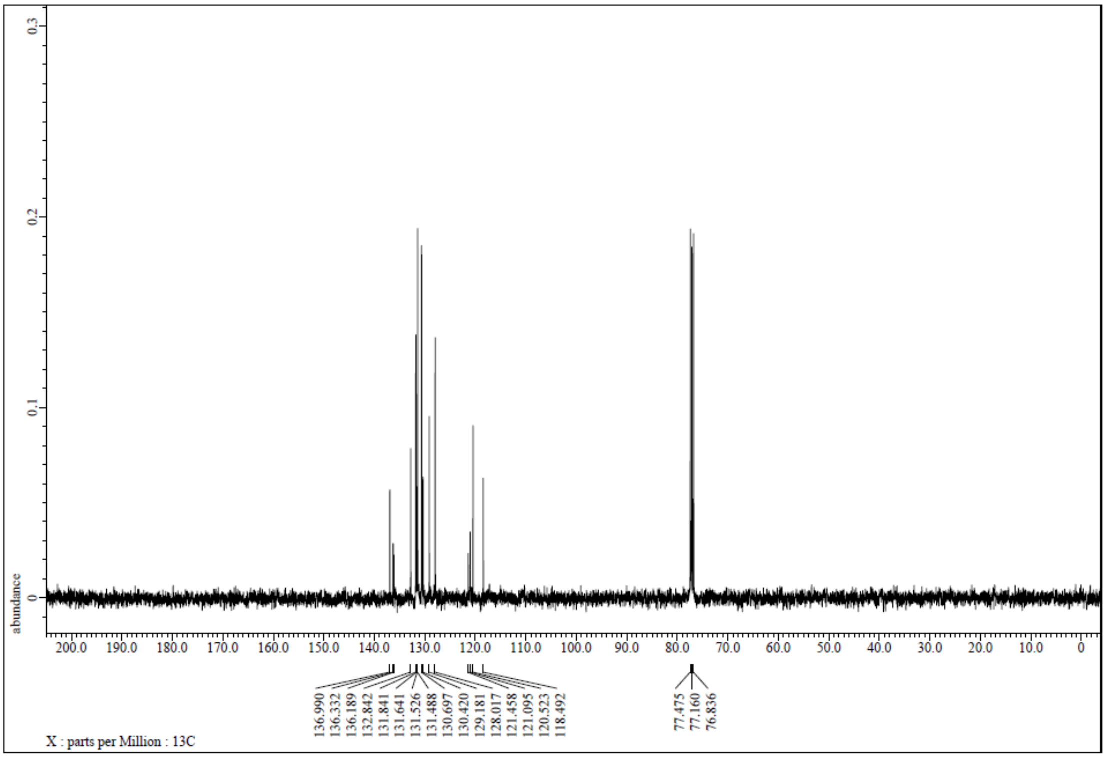

Figure S51. ^{13}C NMR spectrum of 3x, related to Figure 2.

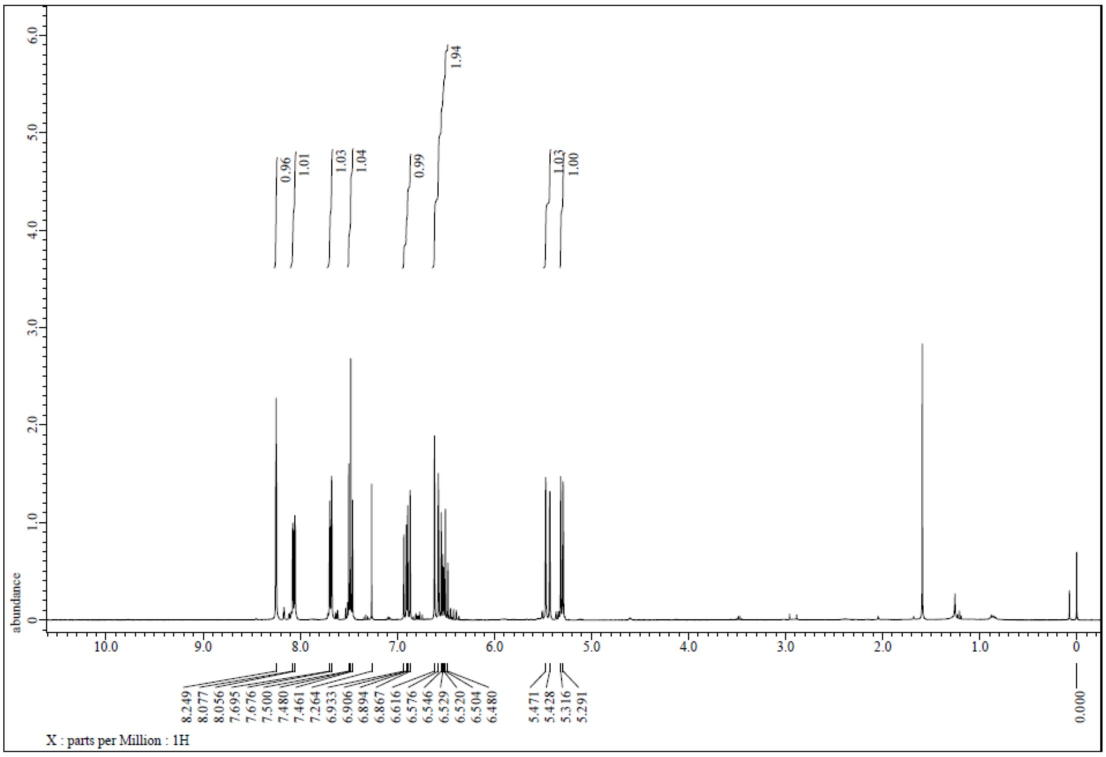

Figure S52. ^{1}H NMR spectrum of 3y, related to Figure 2.

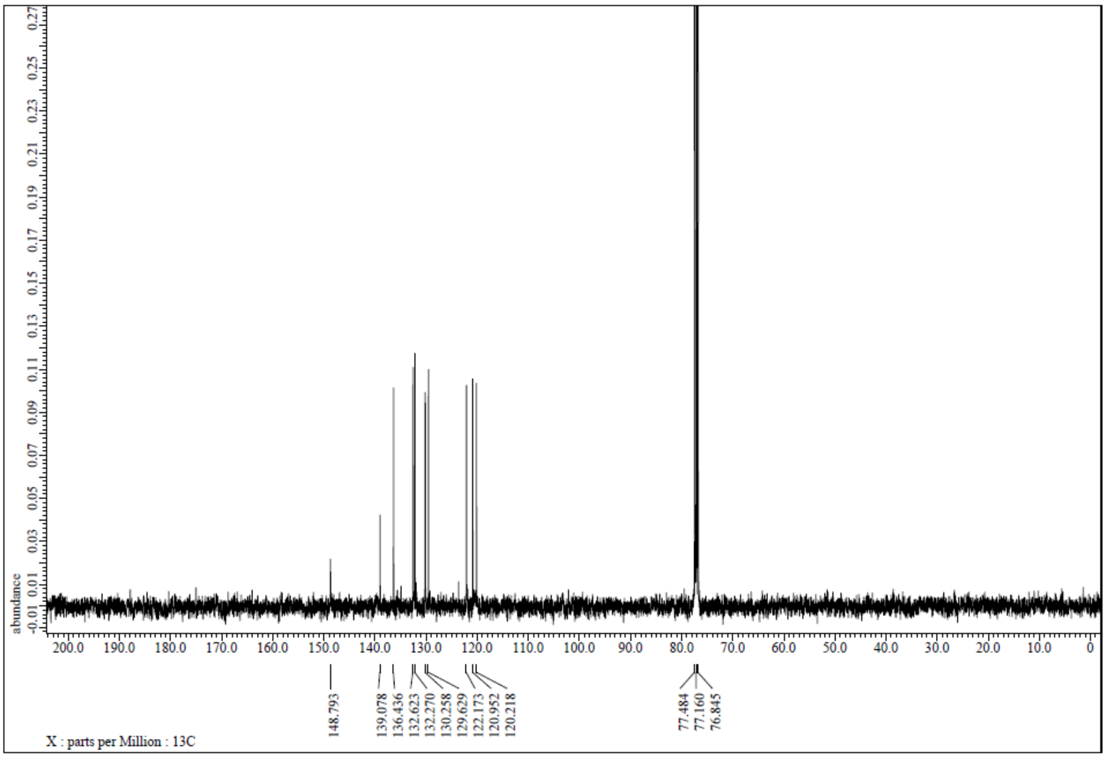

Figure S53. ^{13}C NMR spectrum of 3y, related to Figure 2.

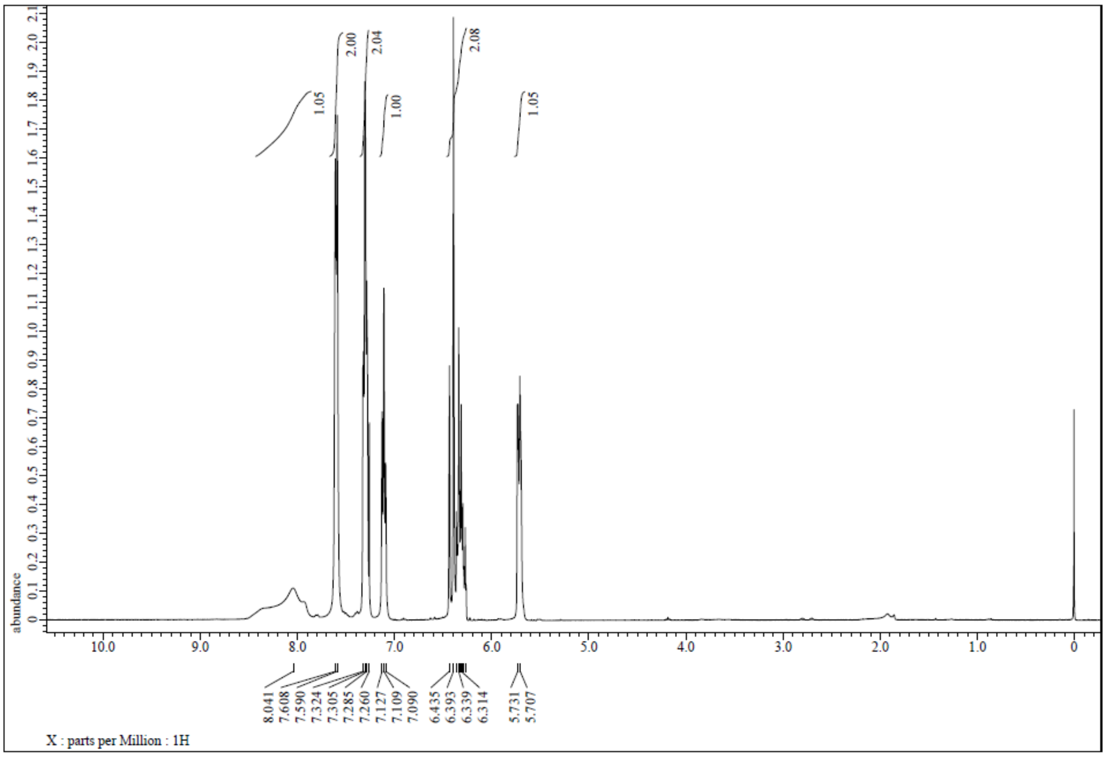

Figure S54. ^{1}H NMR spectrum of 3z, related to Figure 2.

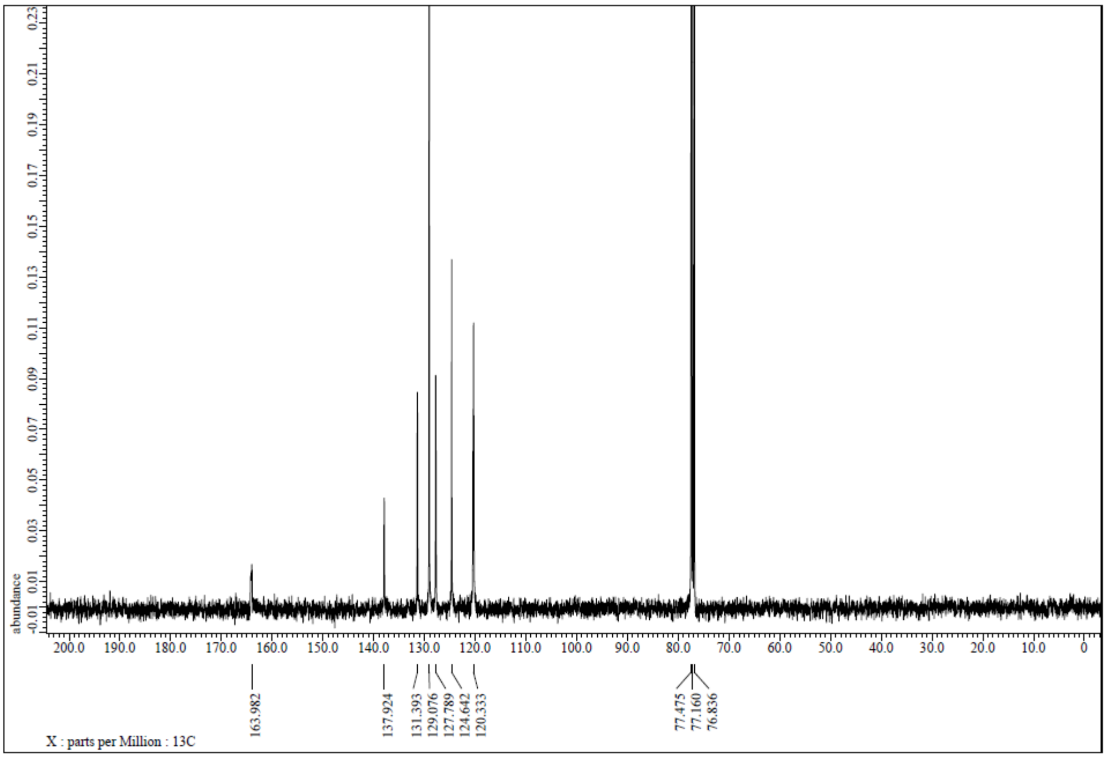

Figure S55. ^{13}C NMR spectrum of 3z, related to Figure 2.

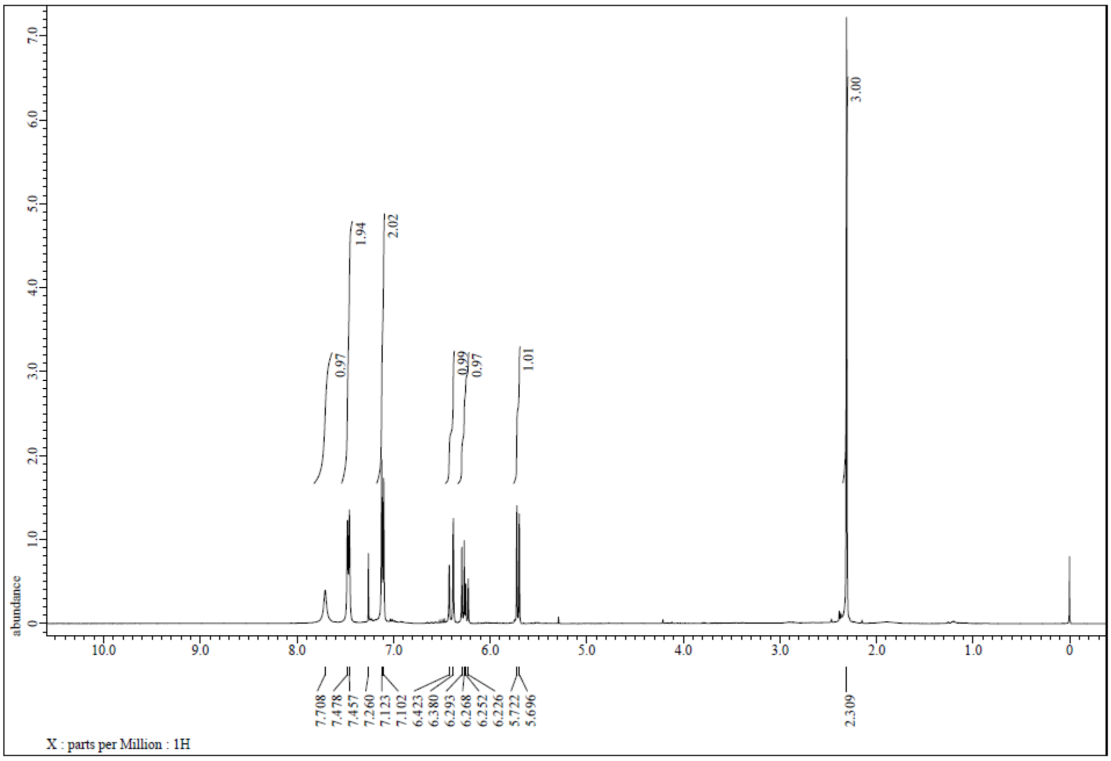

Figure S56. ^{1}H NMR spectrum of 3aa, related to Figure 2.

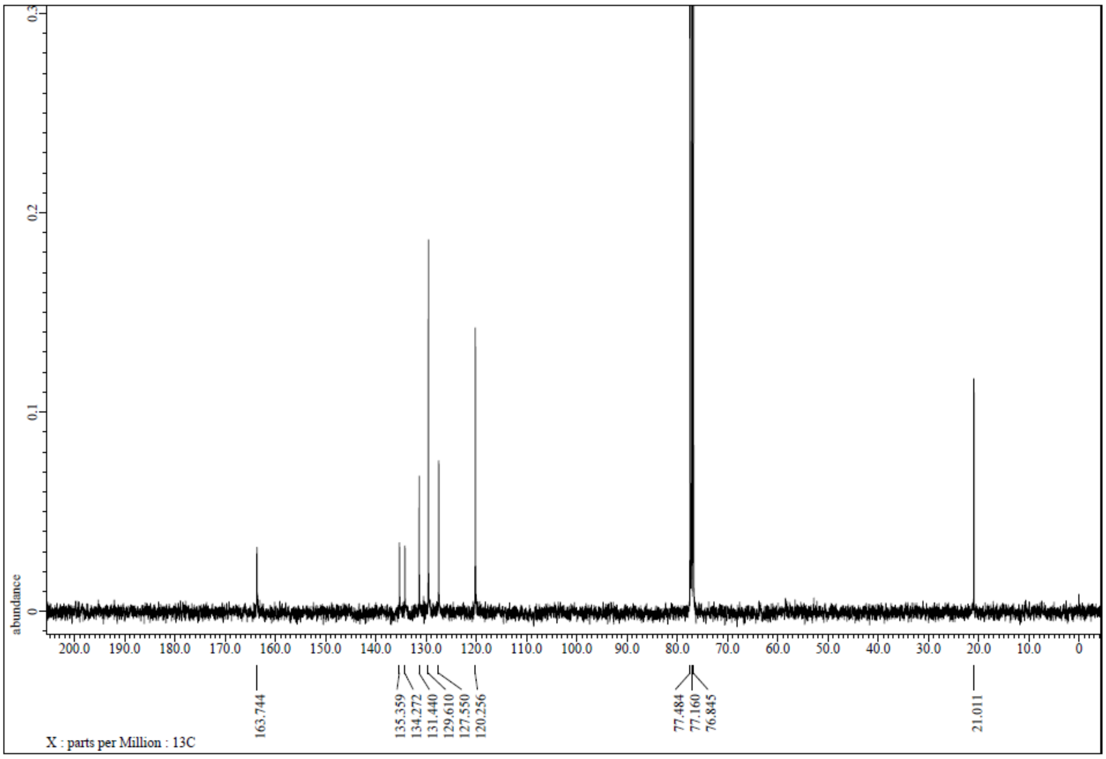

Figure S57. ^{13}C NMR spectrum of 3aa, related to Figure 2.

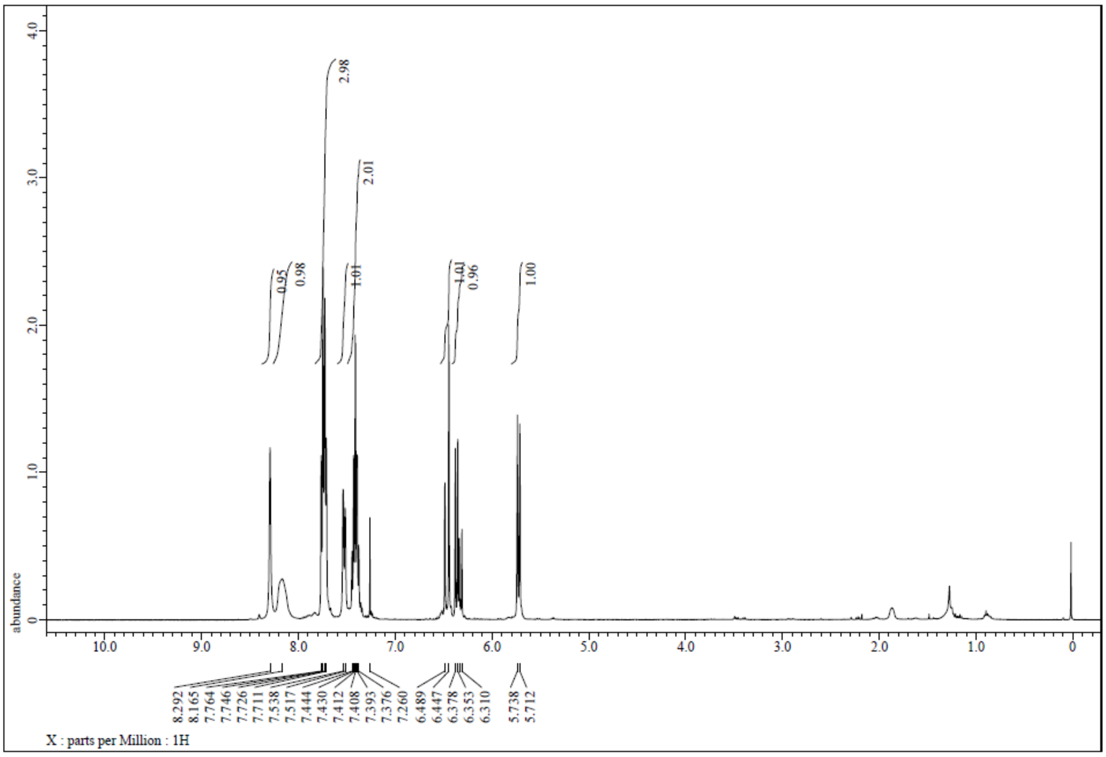

Figure S58. ^{1}H NMR spectrum of 3ab, related to Figure 2.

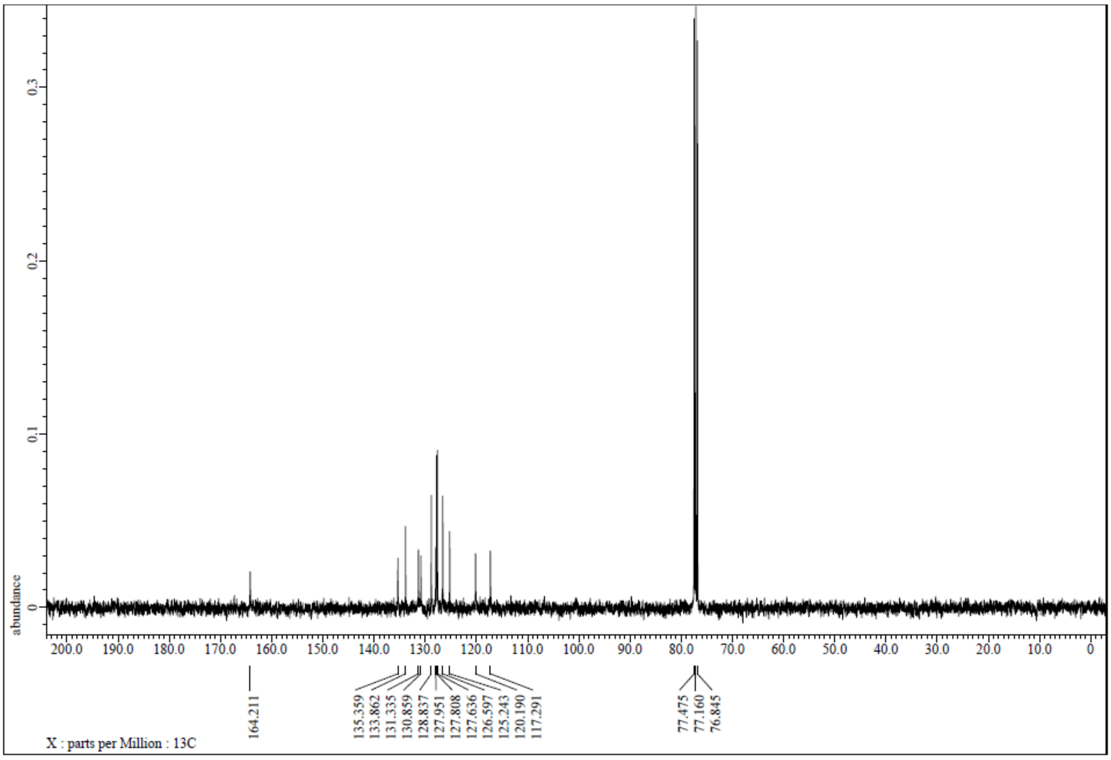

Figure S59. ^{13}C NMR spectrum of 3ab, related to Figure 2.

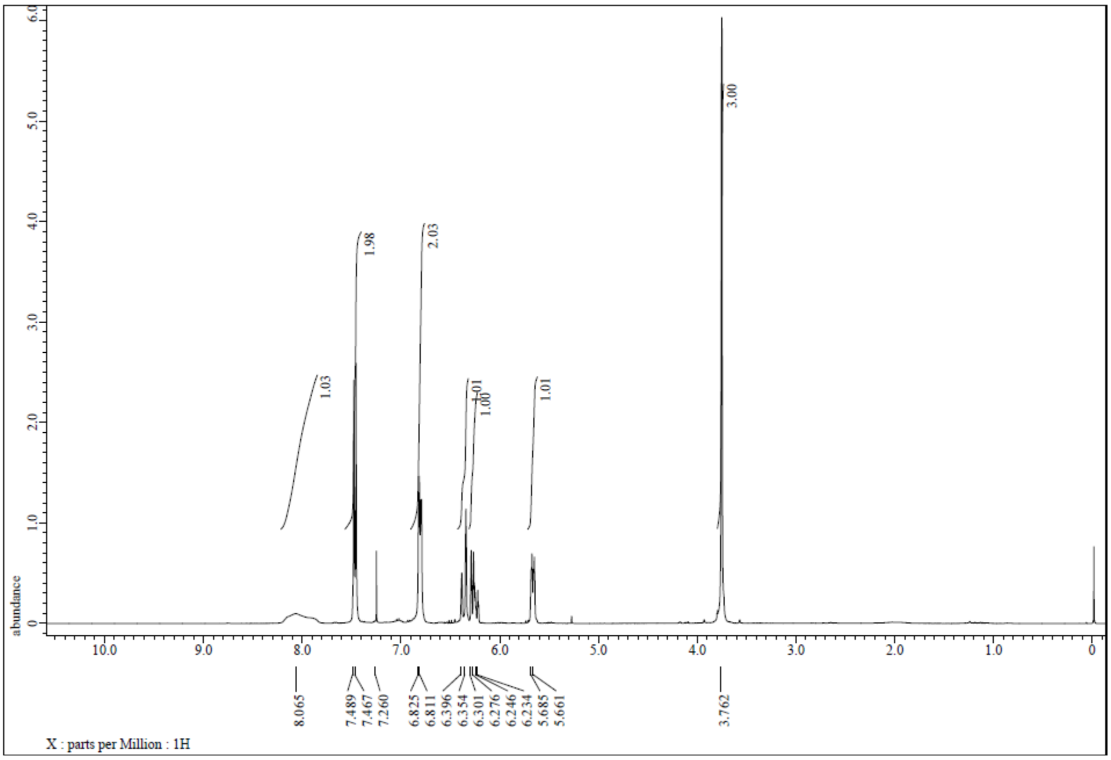

Figure S60. ^{1}H NMR spectrum of 3ac, related to Figure 2.

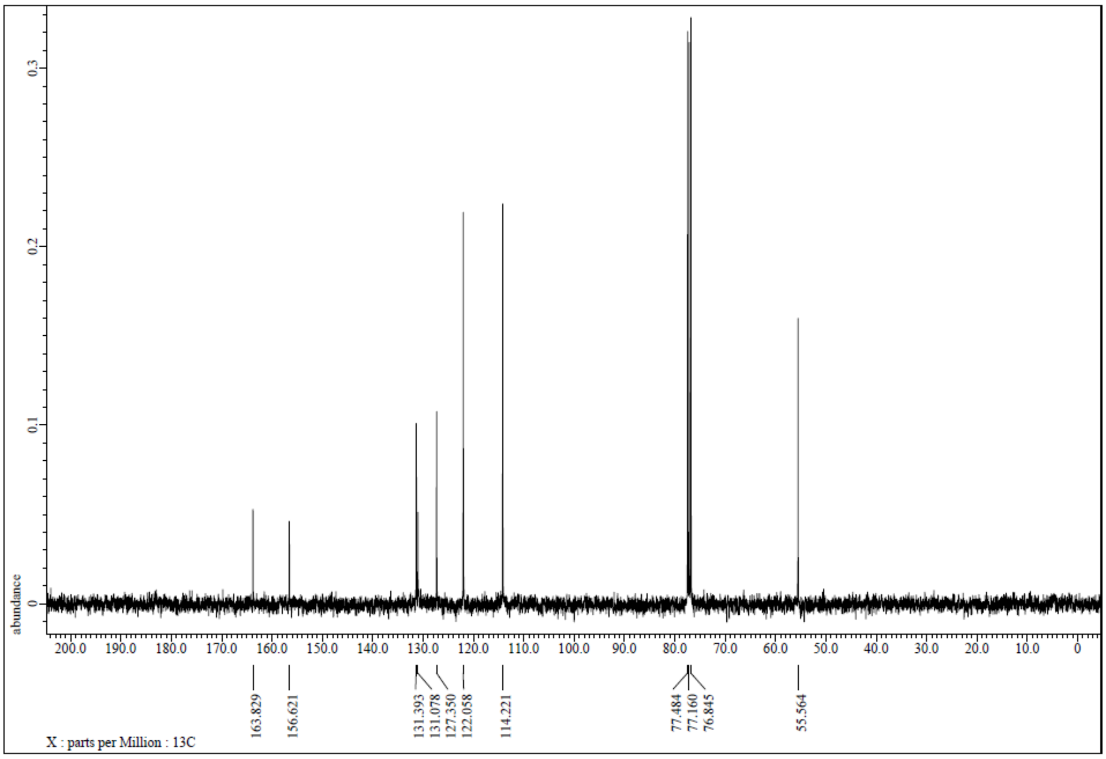

Figure S61. ^{13}C NMR spectrum of 3ac, related to Figure 2.

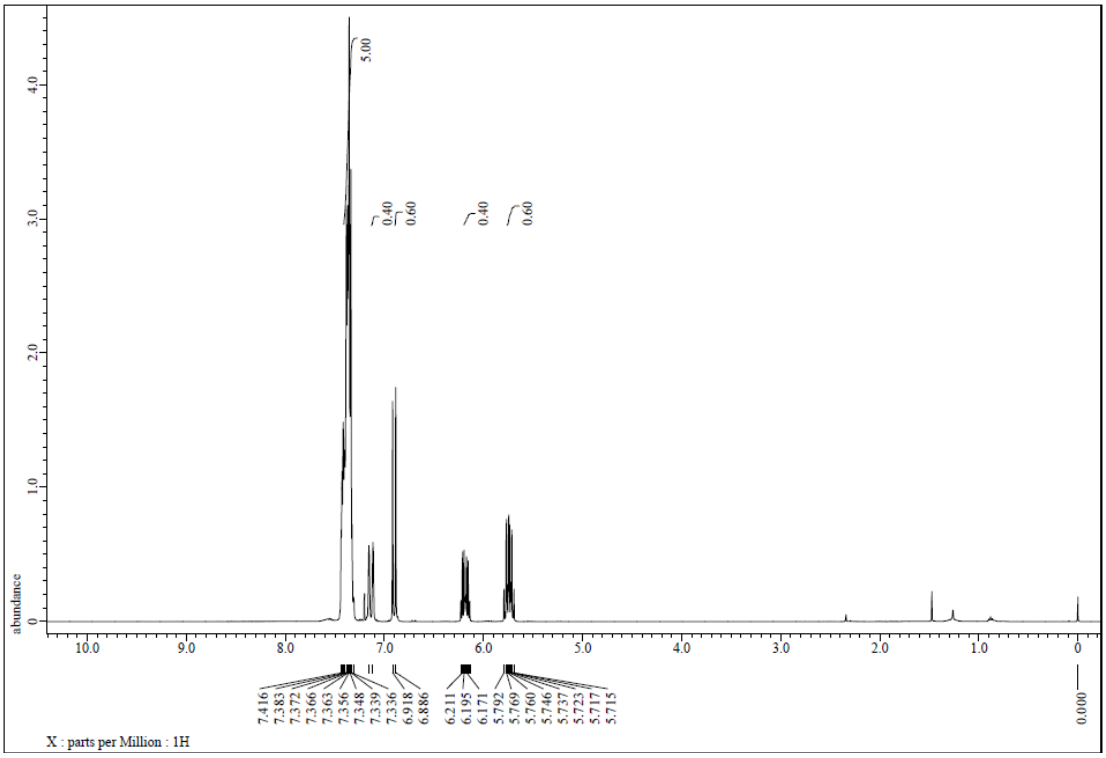

Figure S62. ^{1}H NMR spectrum of 4a, related to Figure 3.

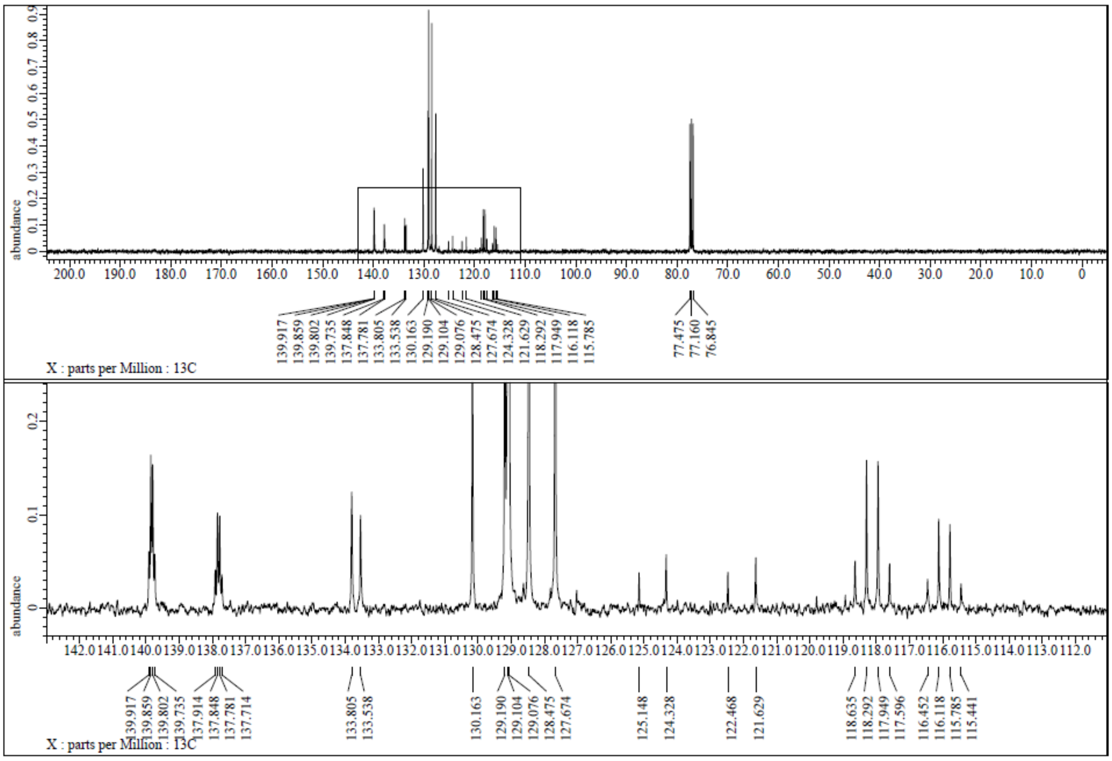

Figure S63. ^{13}C NMR spectrum of 4a, related to Figure 3.

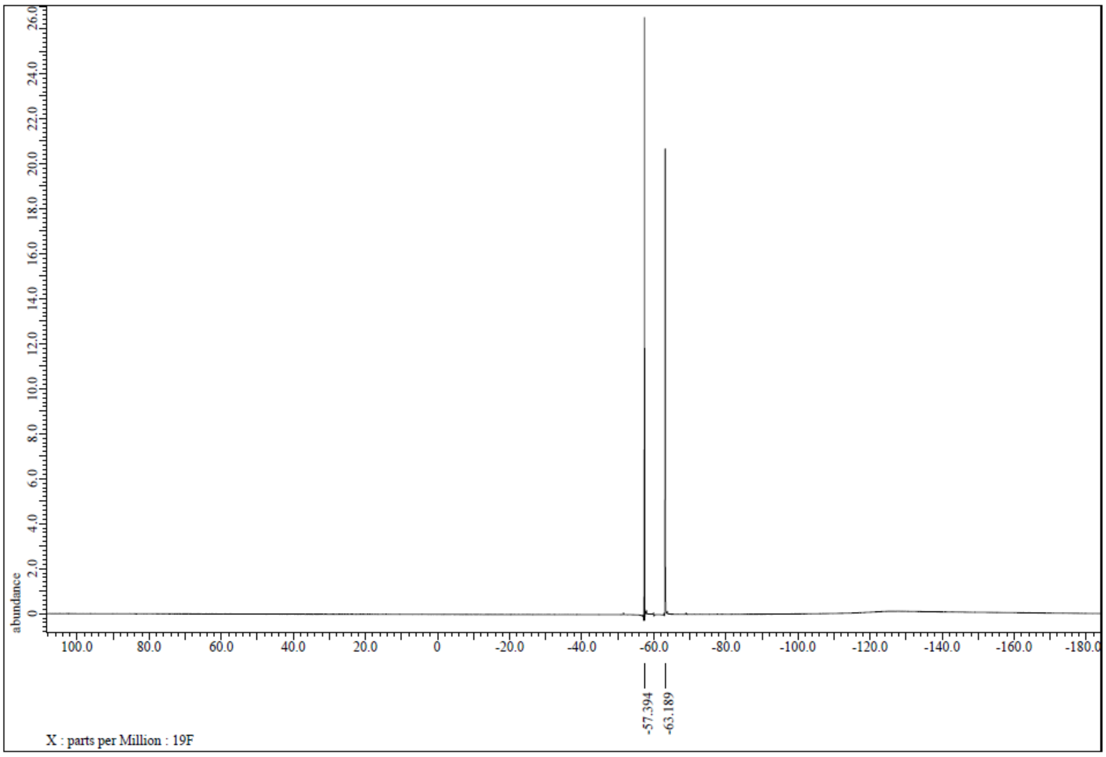

Figure S64. ^{19}F NMR spectrum of 4a, related to Figure 3.

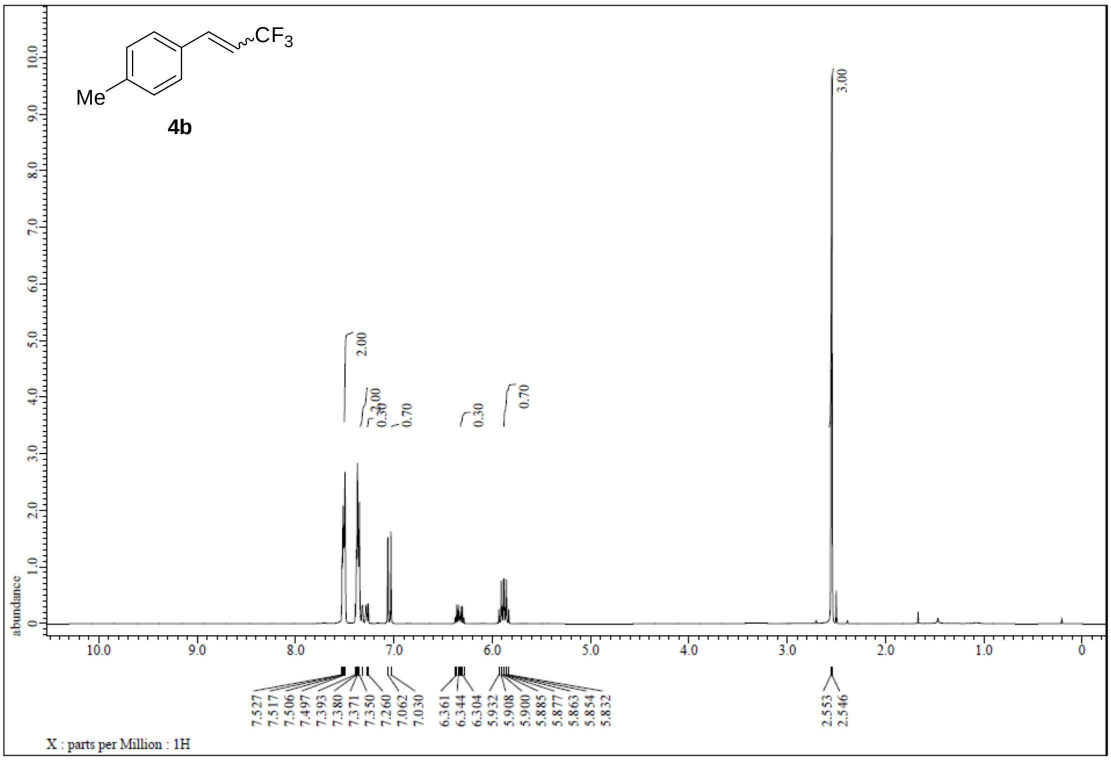

Figure S65. ^{1}H NMR spectrum of 4b, related to Figure 3.

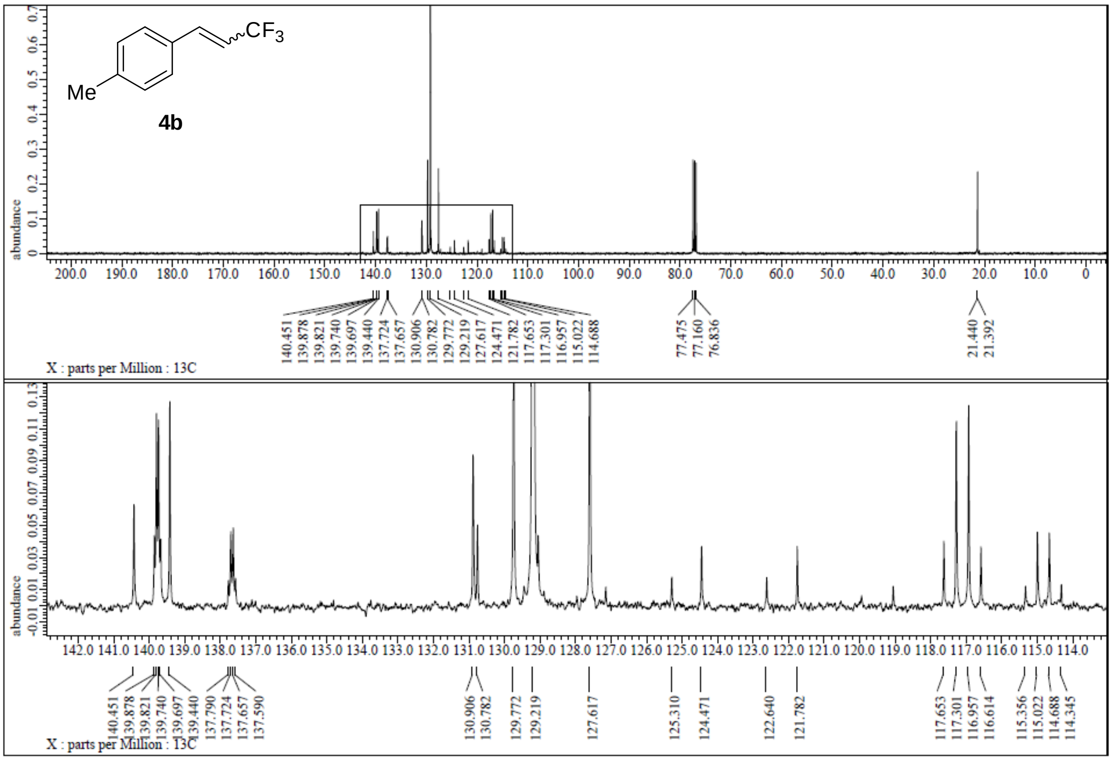

Figure S66. ^{13}C NMR spectrum of 4b, related to Figure 3.

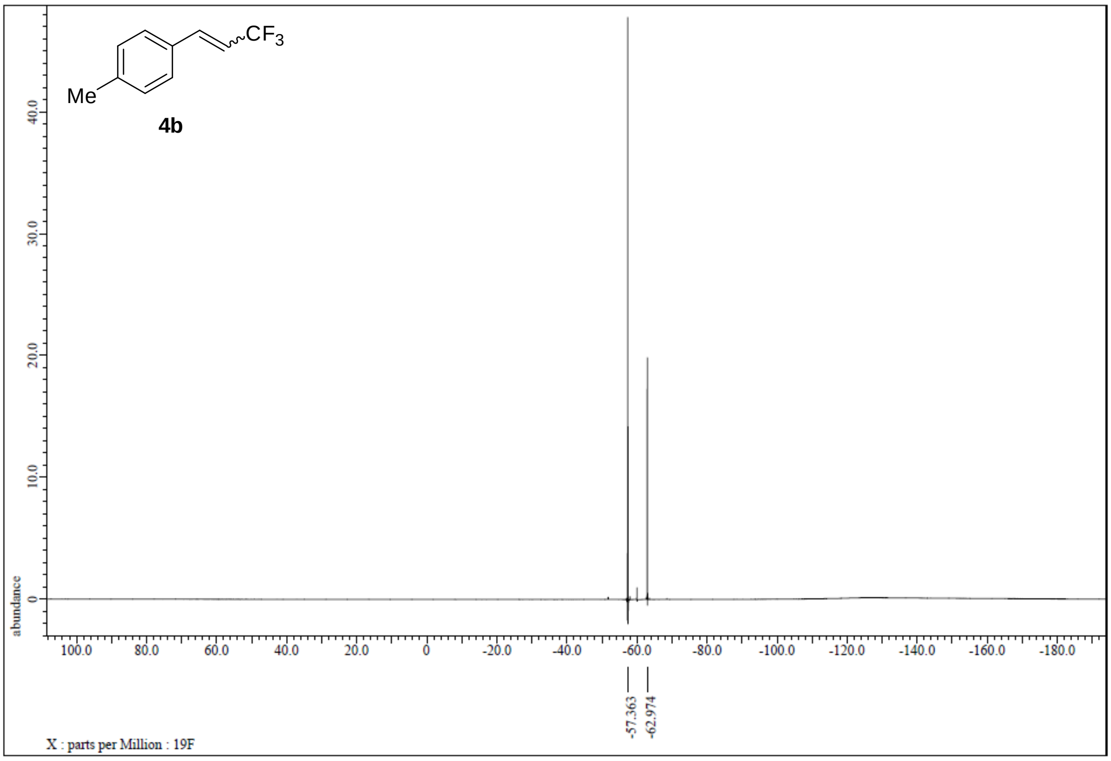

Figure S67. ^{19}F NMR spectrum of 4b, related to Figure 3.

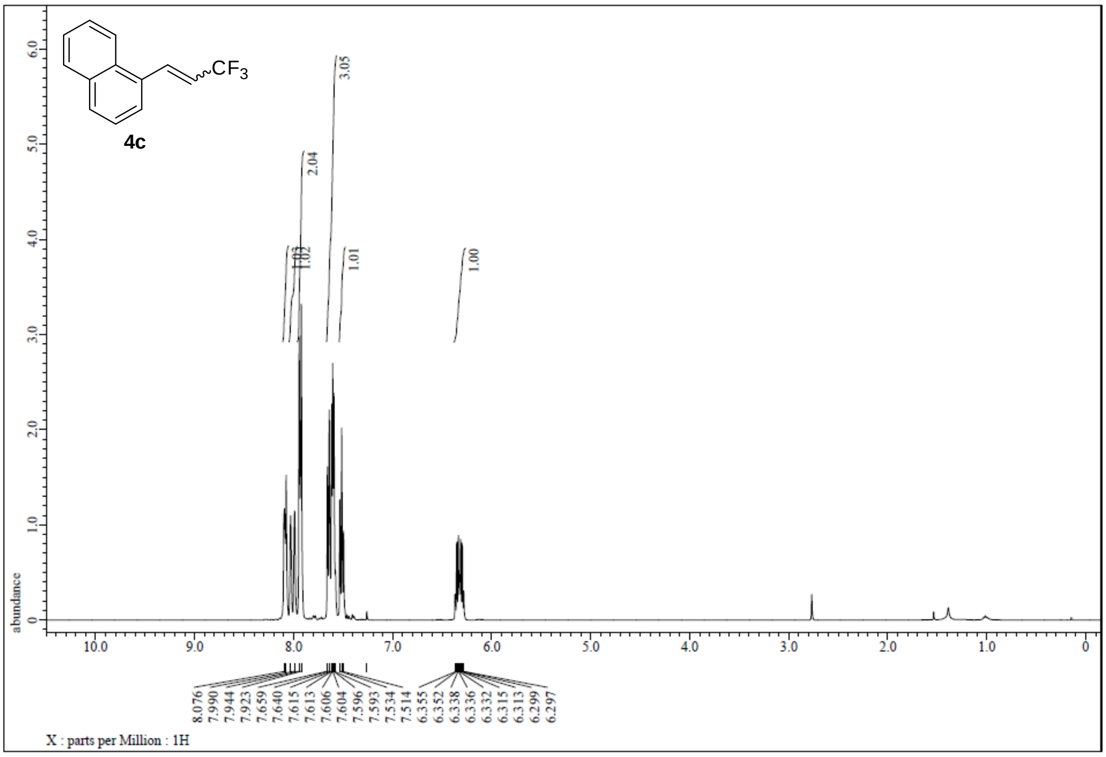

Figure S68. ^{1}H NMR spectrum of 4c, related to Figure 3.

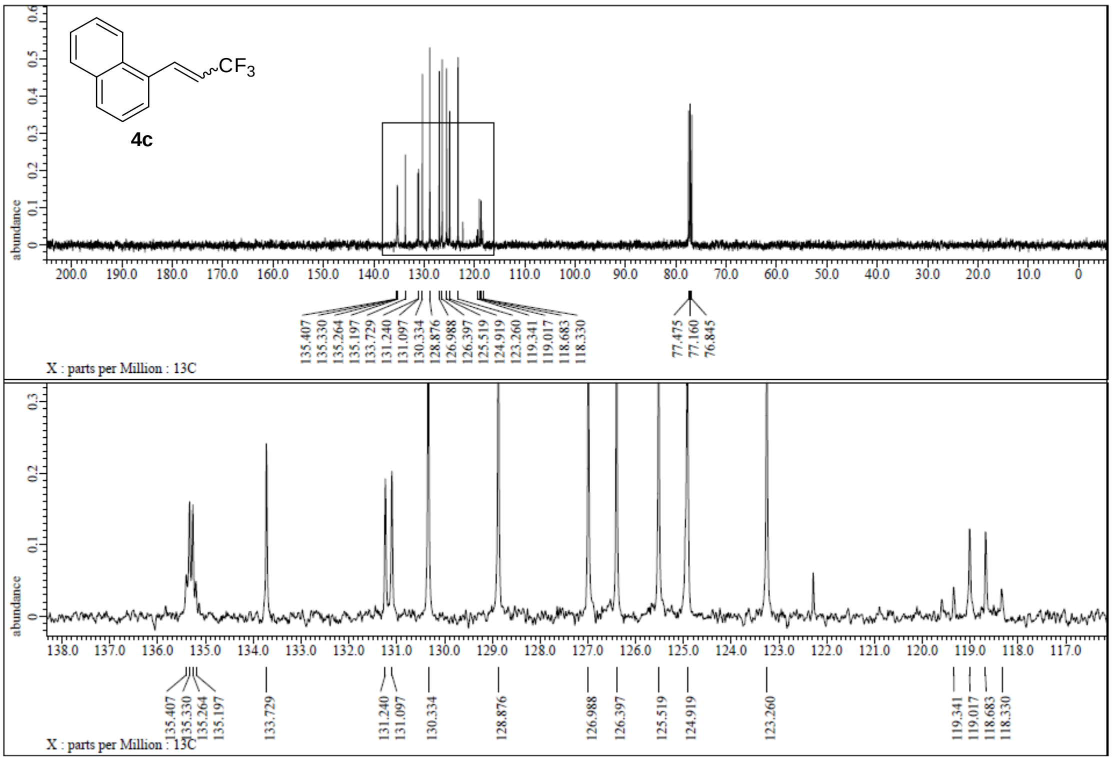

Figure S69. ^{13}C NMR spectrum of 4c, related to Figure 3.

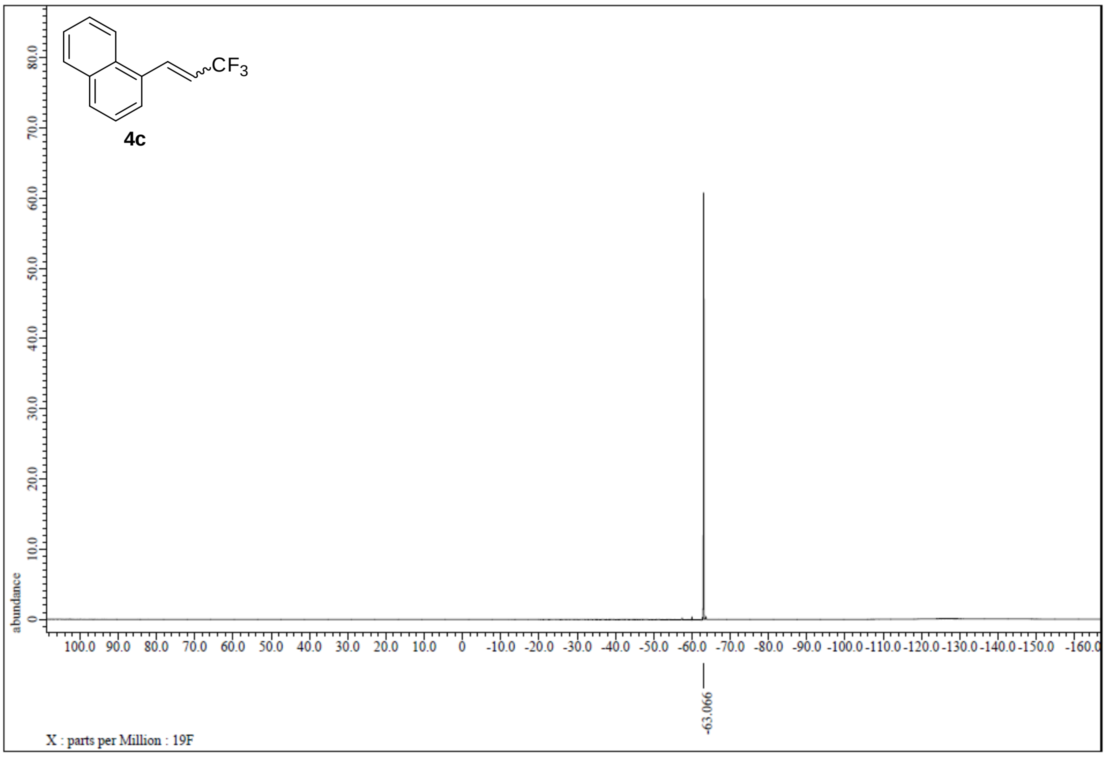

Figure S67. ^{19}F NMR spectrum of 4c, related to Figure 3.

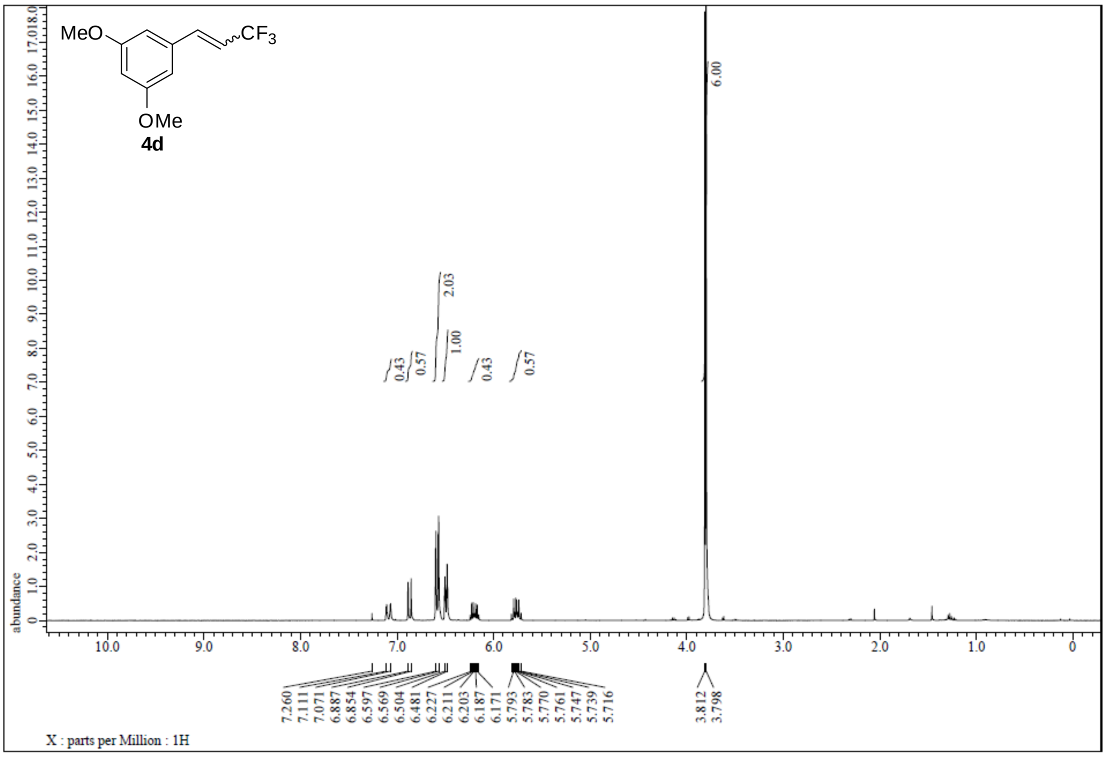

Figure S71. ^{1}H NMR spectrum of 4d, related to Figure 3.

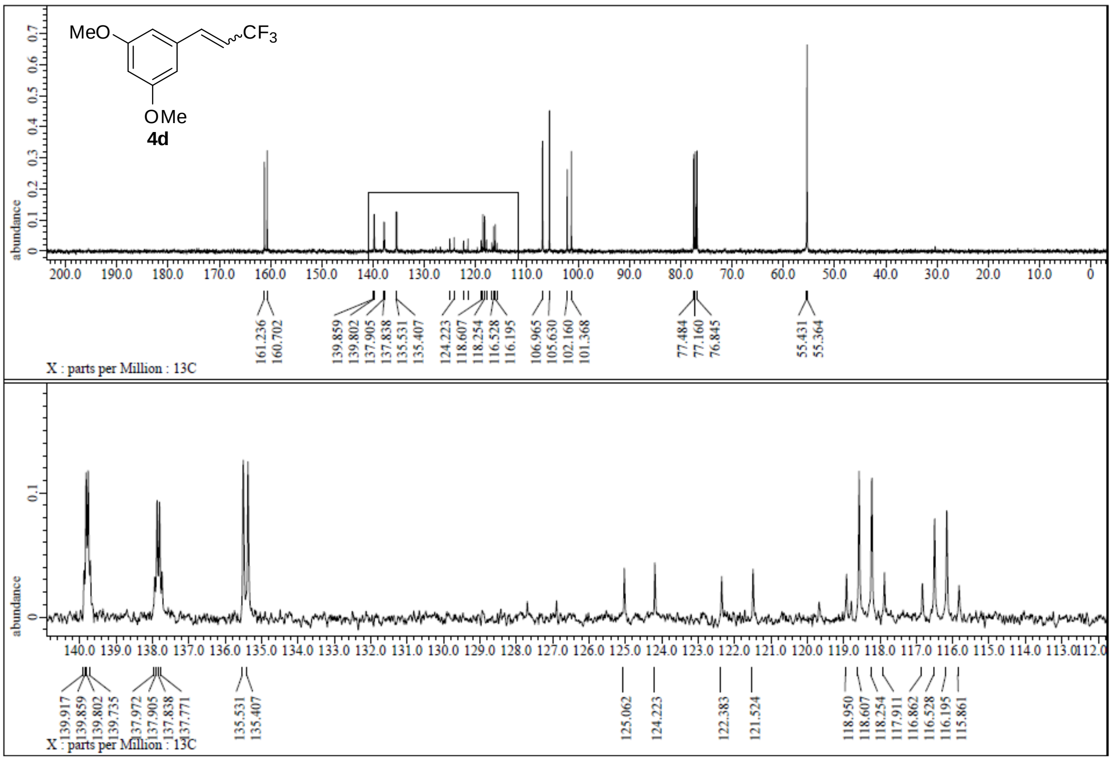

Figure S72. ^{13}C NMR spectrum of 4d, related to Figure 3.

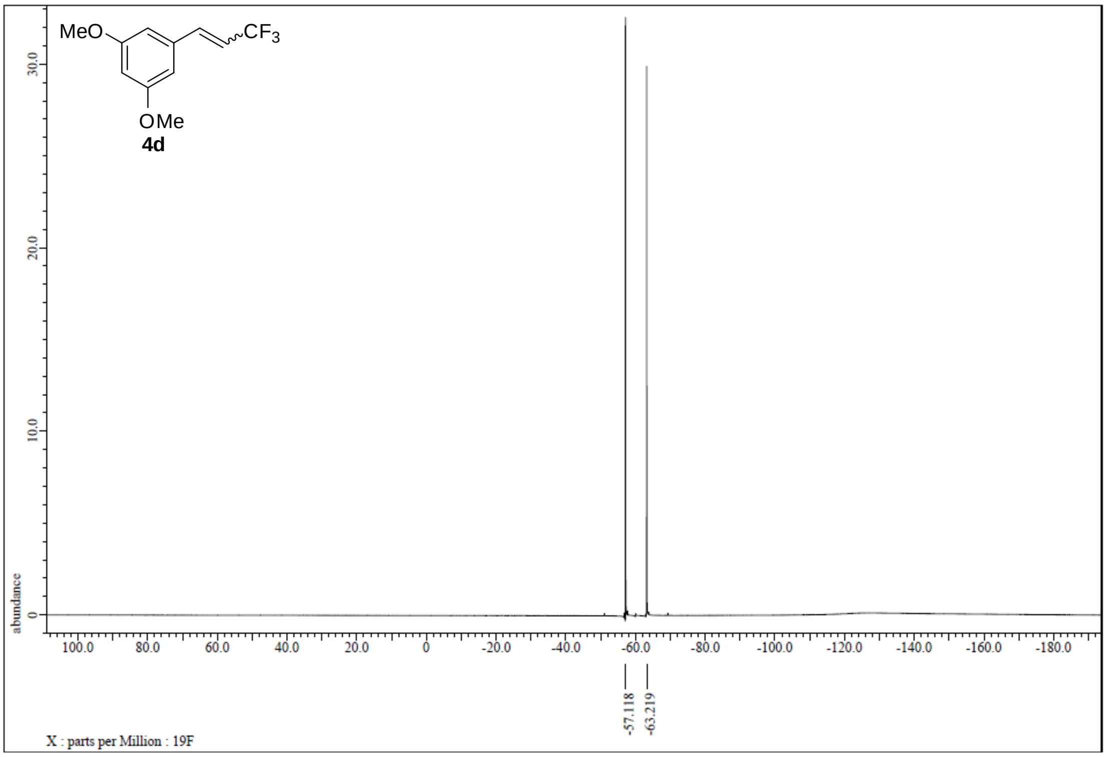

Figure S73. ^{19}F NMR spectrum of 4d, related to Figure 3.

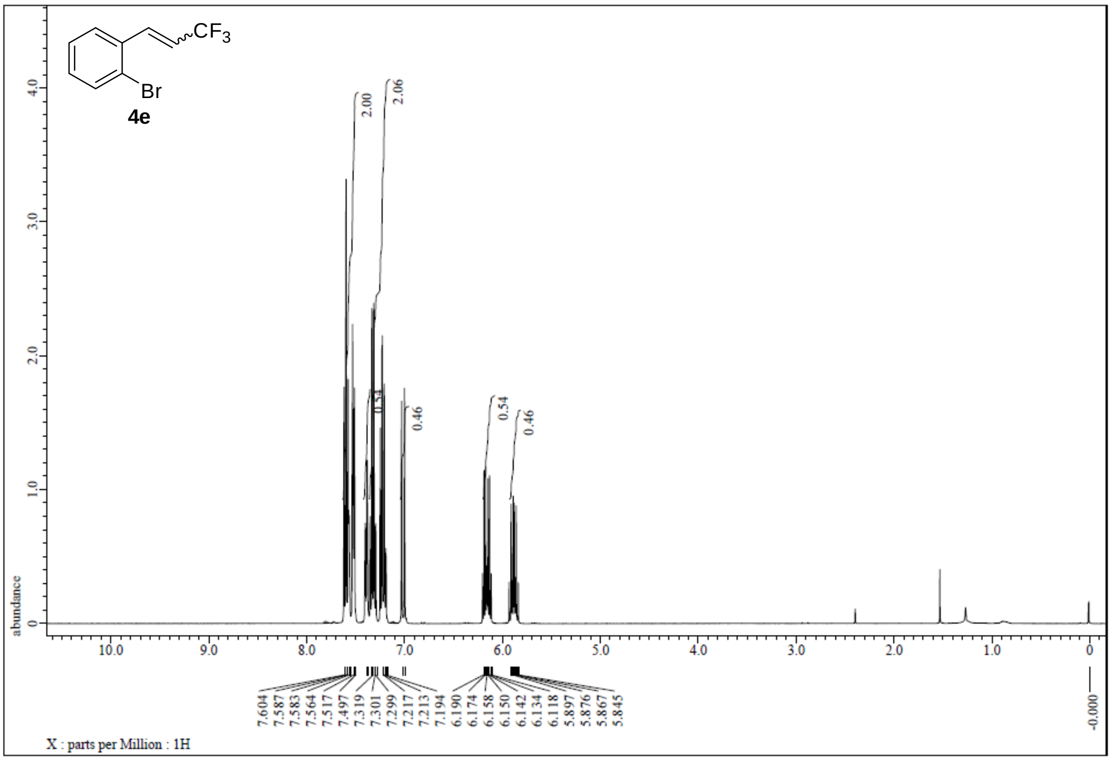

Figure S74. ^{1}H NMR spectrum of 4e, related to Figure 3.

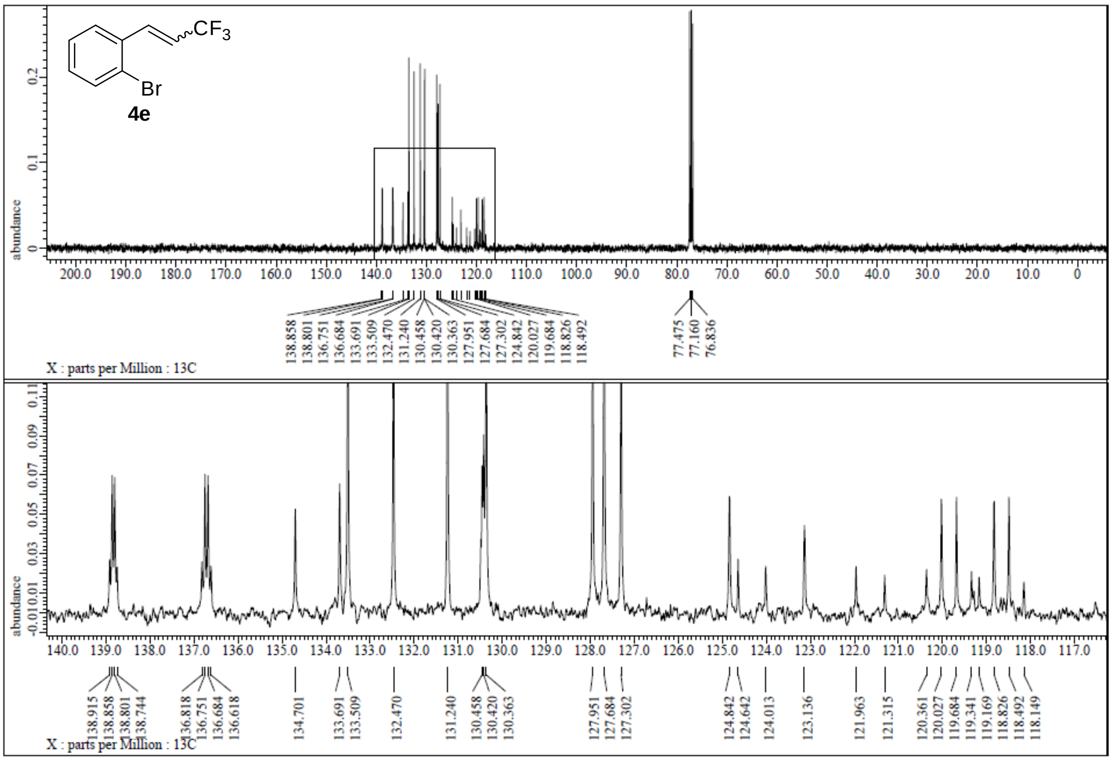

Figure S75. ^{13}C NMR spectrum of 4e, related to Figure 3.

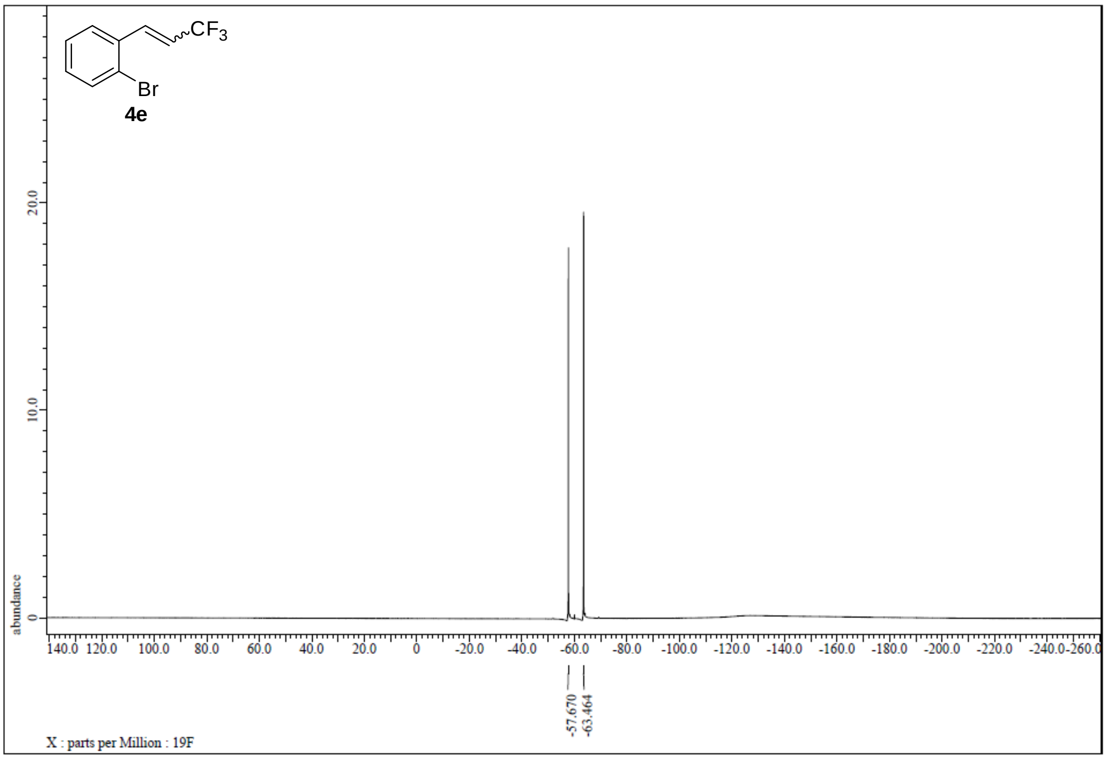

Figure S76. ^{19}F NMR spectrum of 4e, related to Figure 3.

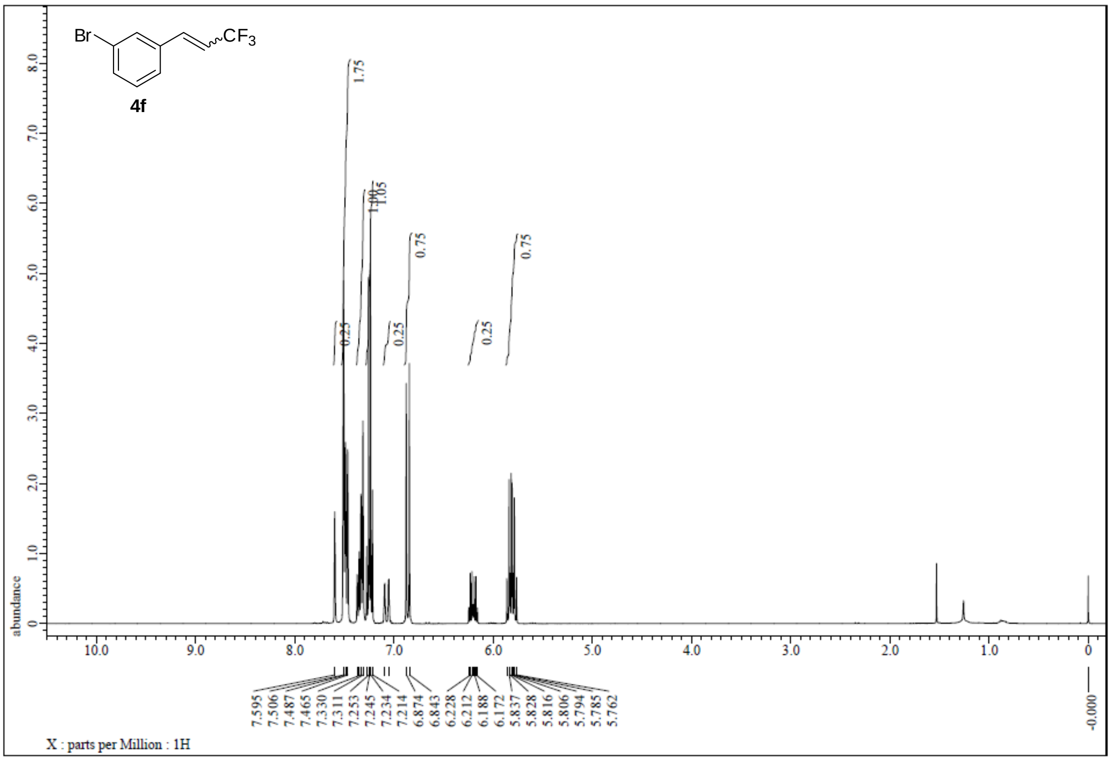

Figure S77. ^{1}H NMR spectrum of 4f, related to Figure 3.

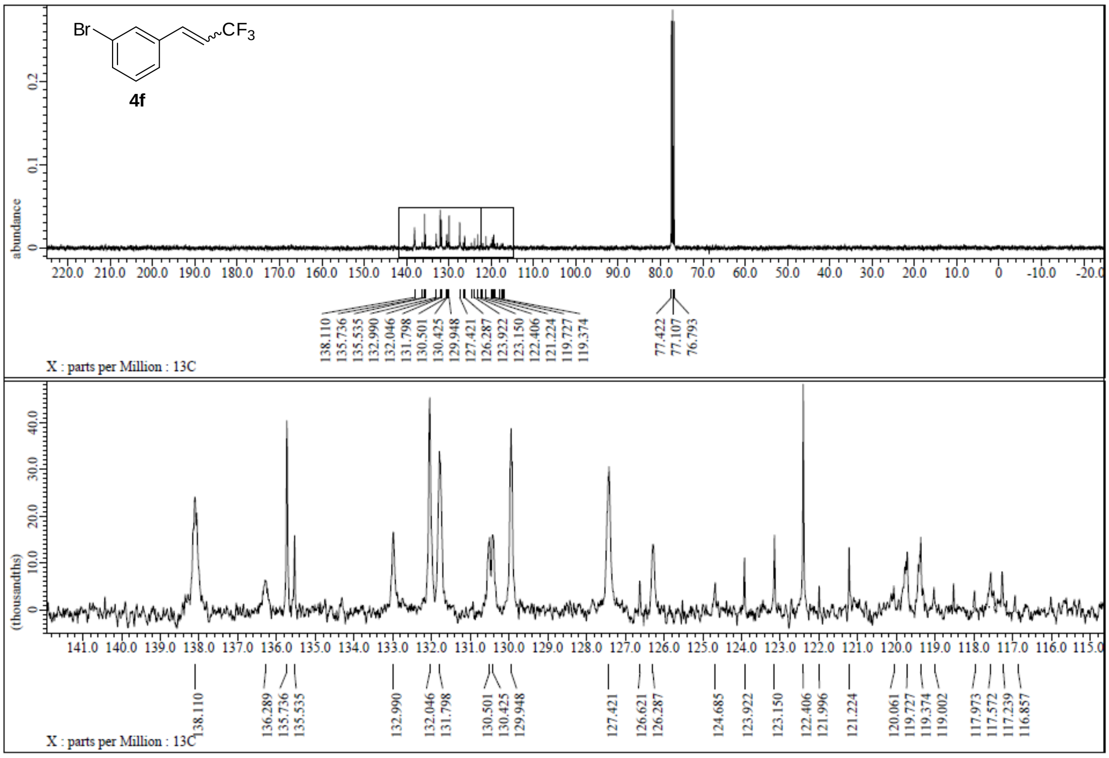

Figure S78. ^{13}C NMR spectrum of 4f, related to Figure 3.

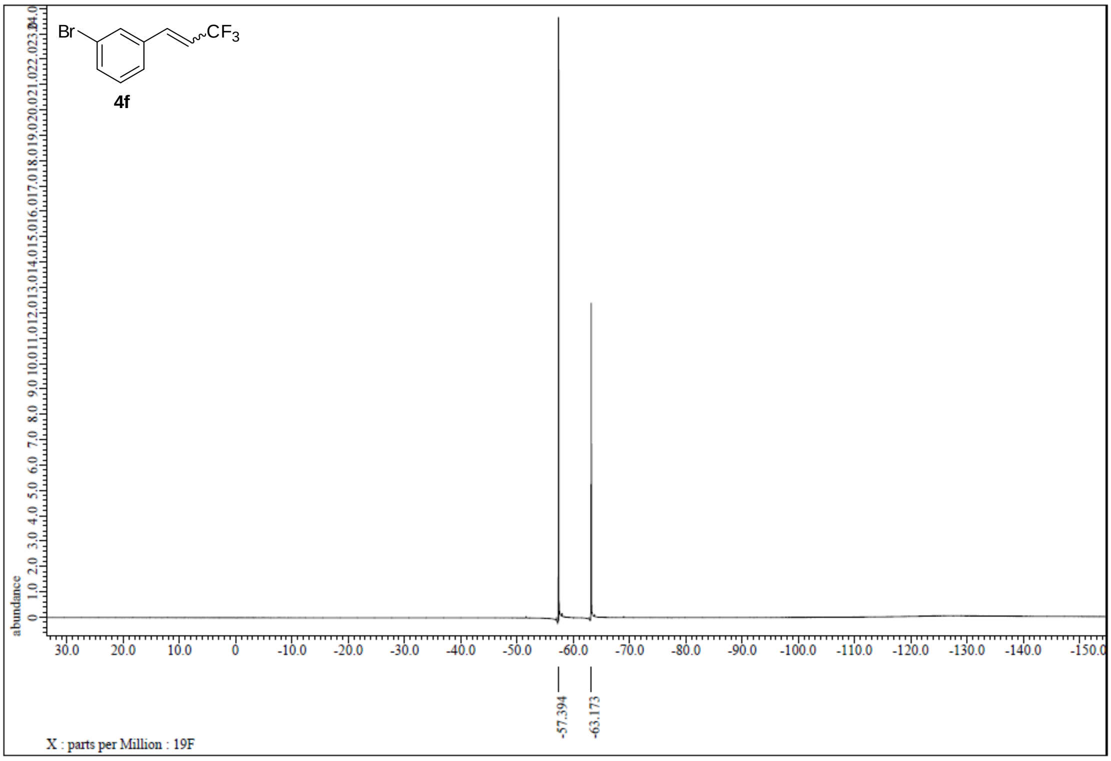

Figure S79. ^{19}F NMR spectrum of 4f, related to Figure 3.

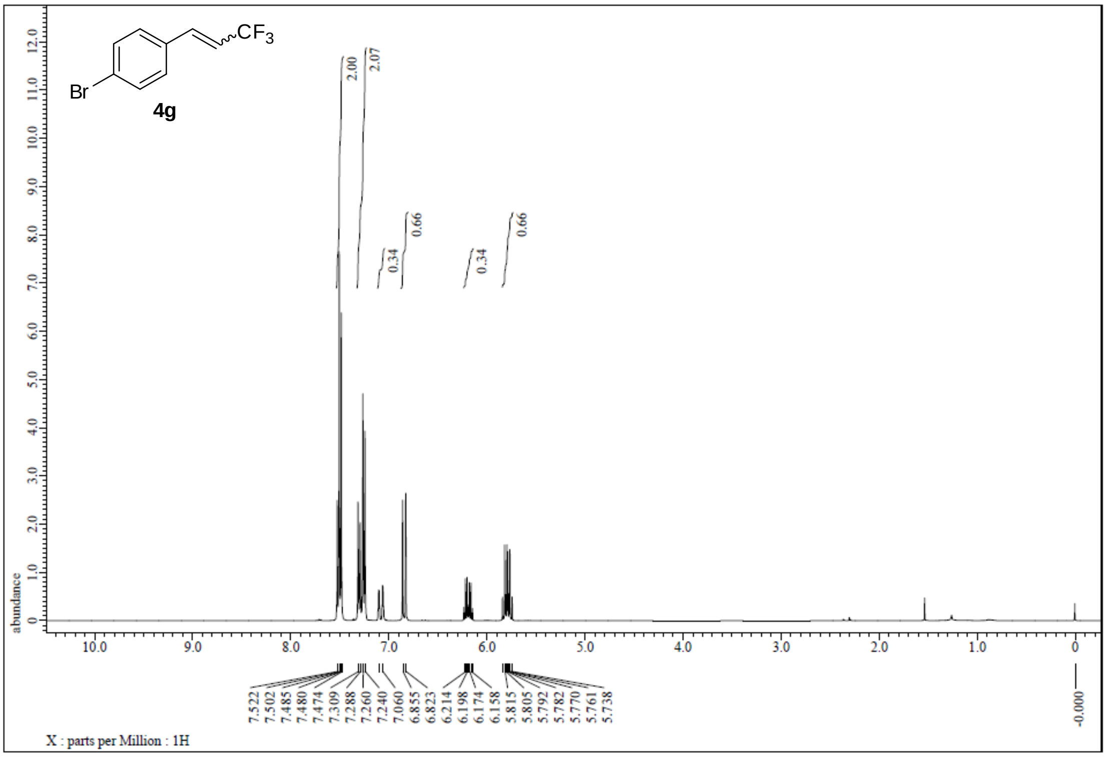

Figure S80. **^{1}H NMR spectrum of 4g, related to Figure 3**.

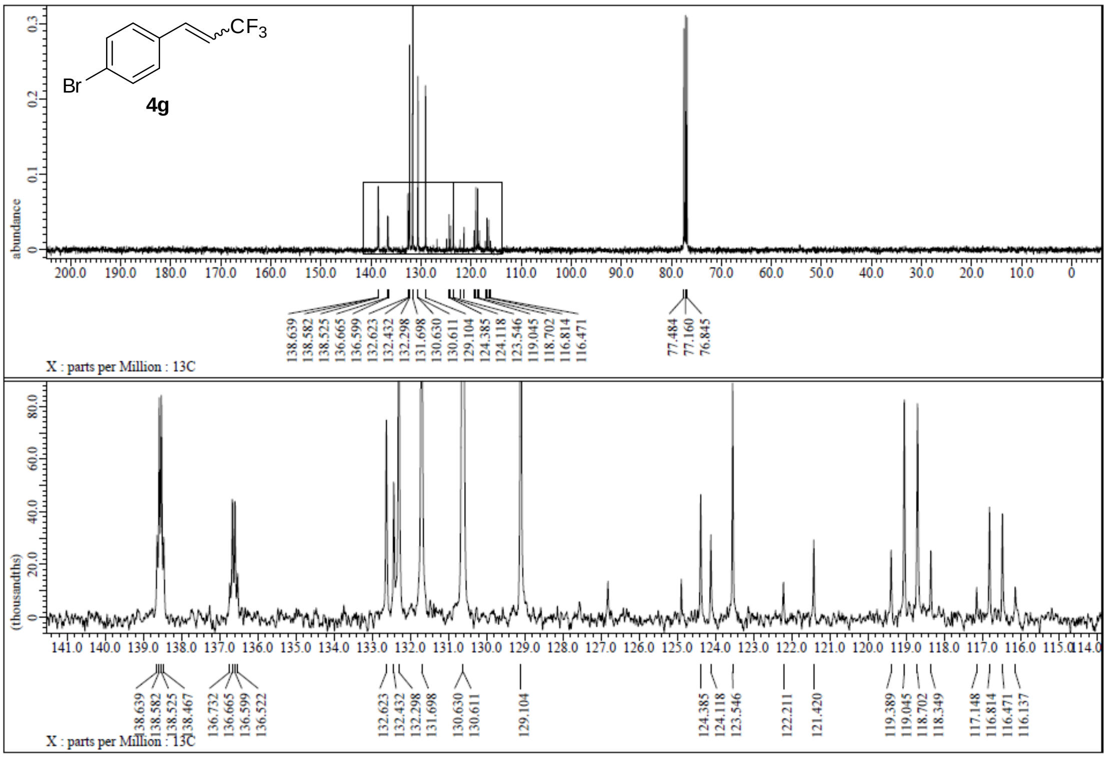

Figure S81. **^{13}C NMR spectrum of 4g, related to Figure 3**.

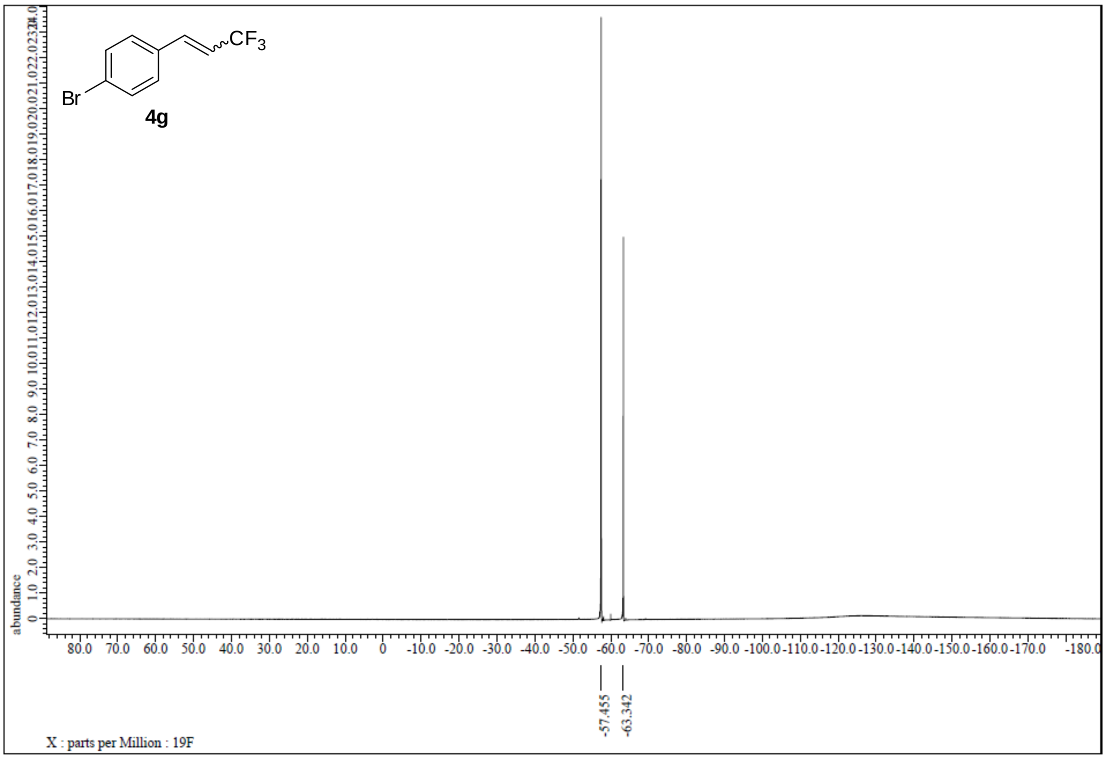

Figure S82. ^{19}F NMR spectrum of 4g, related to Figure 3.

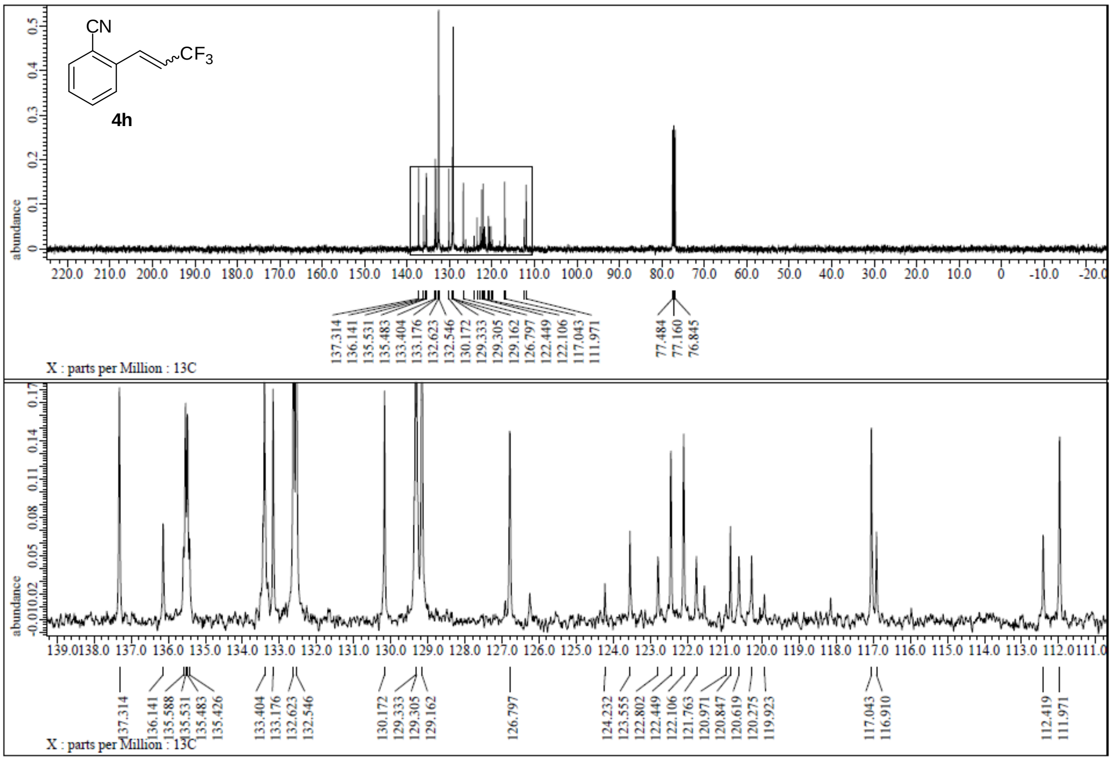

Figure S83. ^{1}H NMR spectrum of 4h, related to Figure 3.

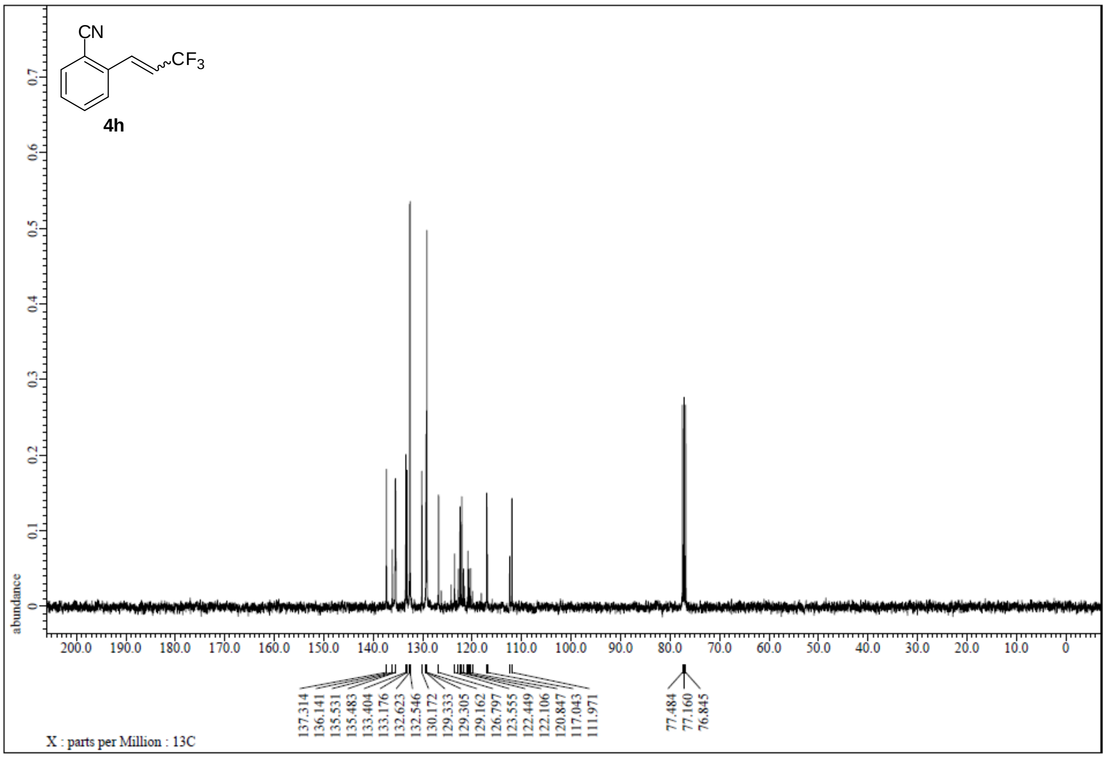

Figure S84. ^{13}C NMR spectrum of 4h, related to Figure 3.

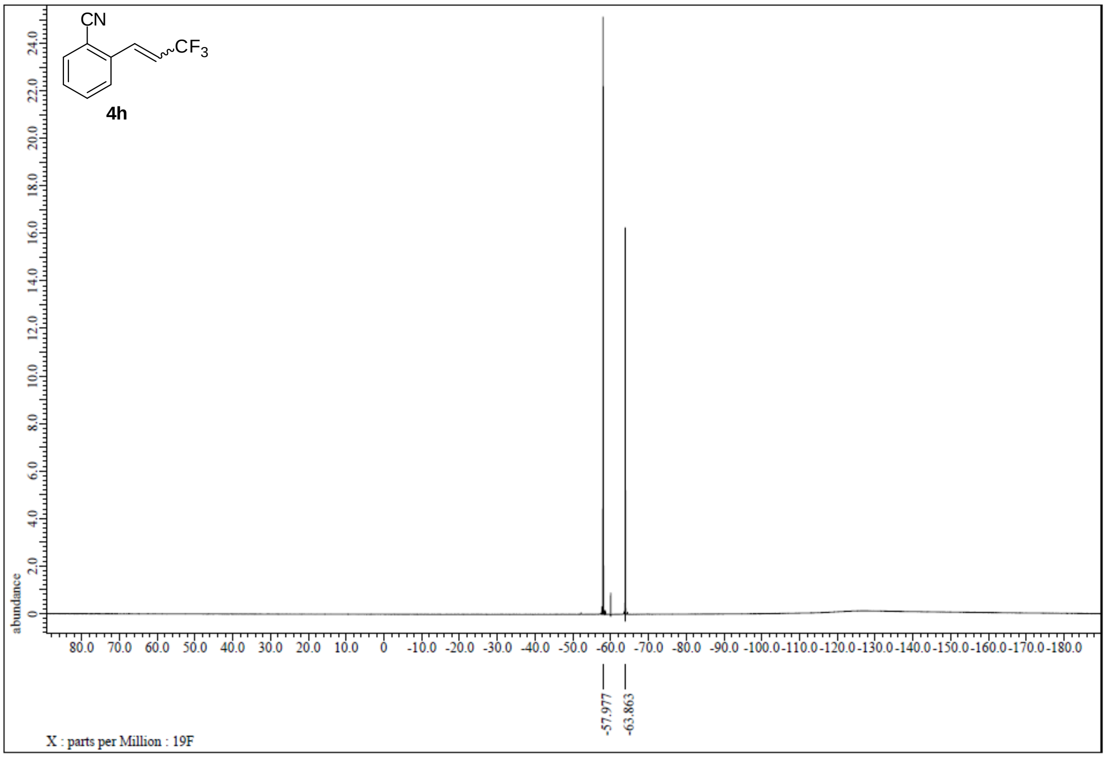

Figure S85. ^{19}F NMR spectrum of 4h, related to Figure 3.

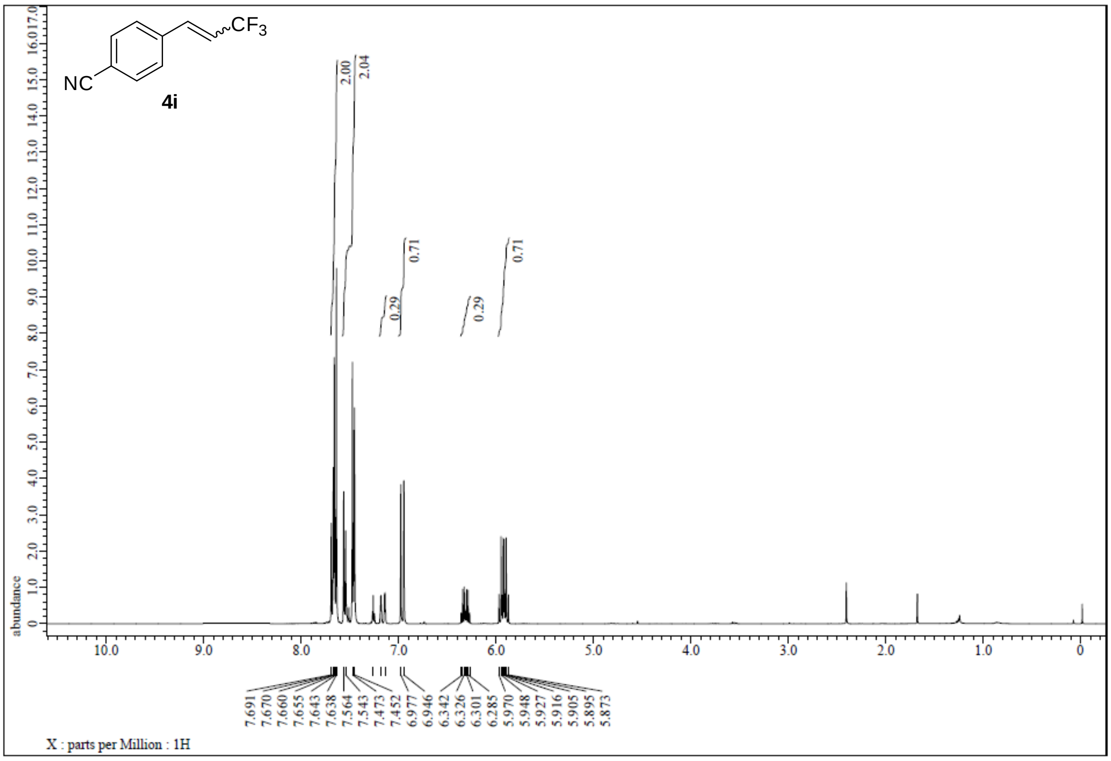

Figure S86. ^{1}H NMR spectrum of 4i, related to Figure 3.

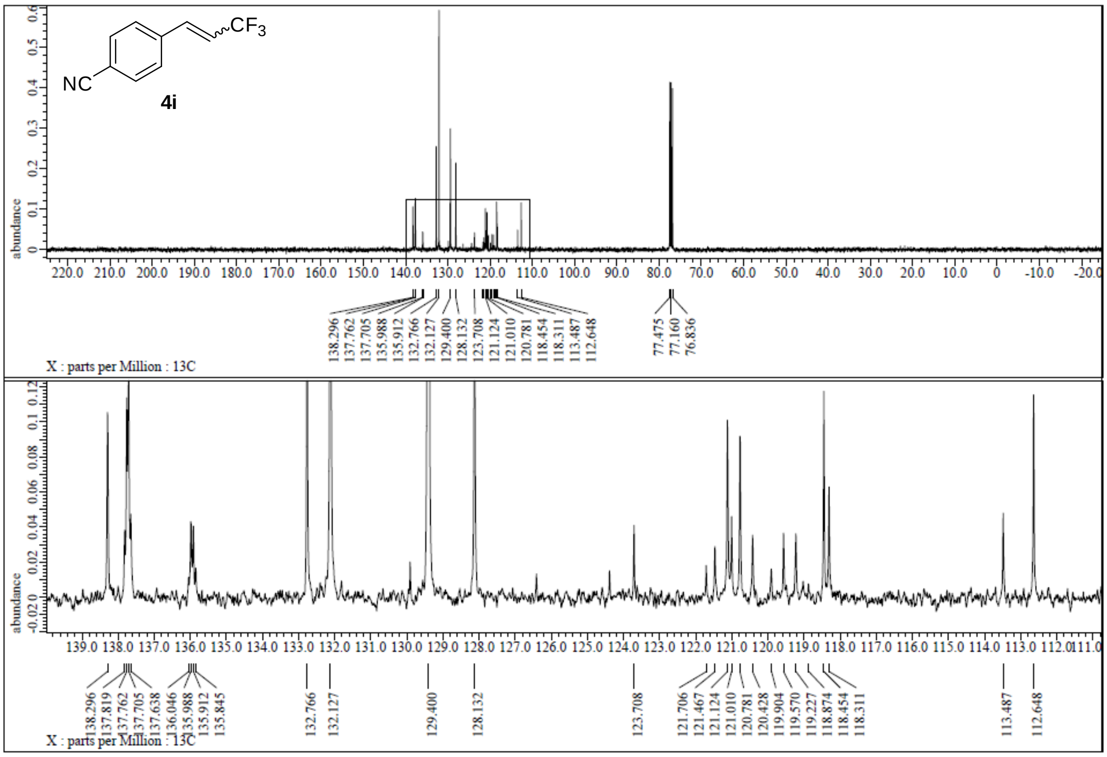

Figure S87. ^{13}C NMR spectrum of 4i, related to Figure 3.

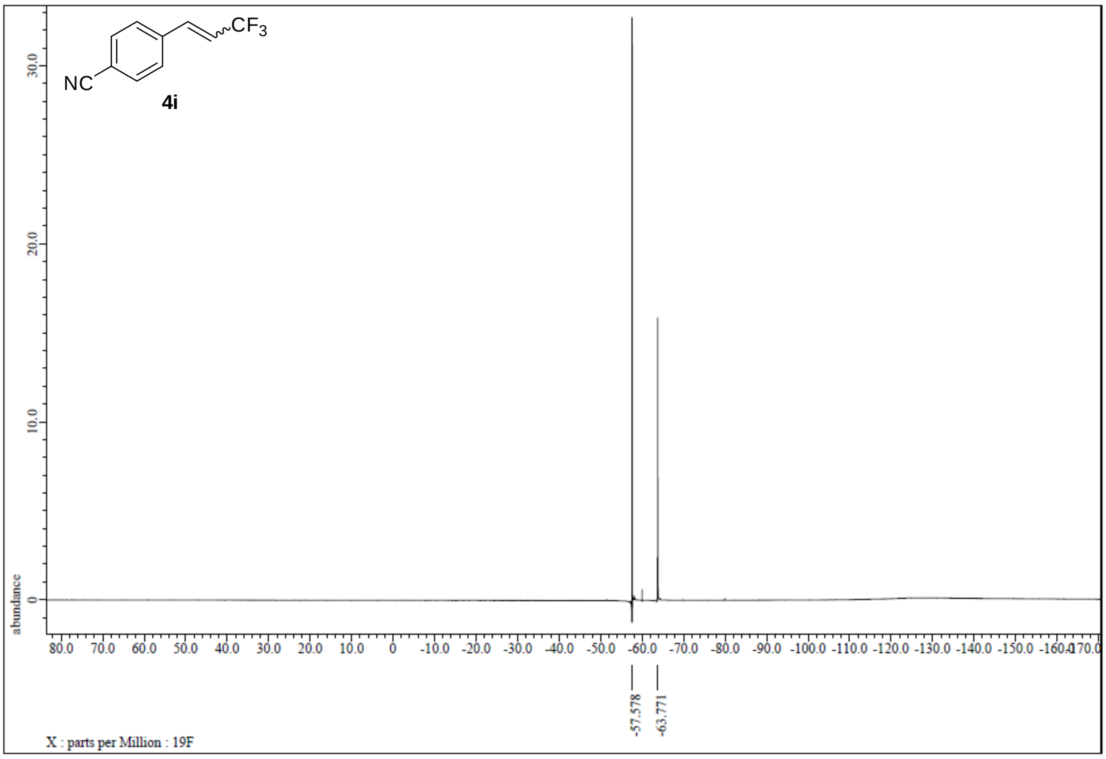

Figure S88. ^{19}F NMR spectrum of 4i, related to Figure 3.

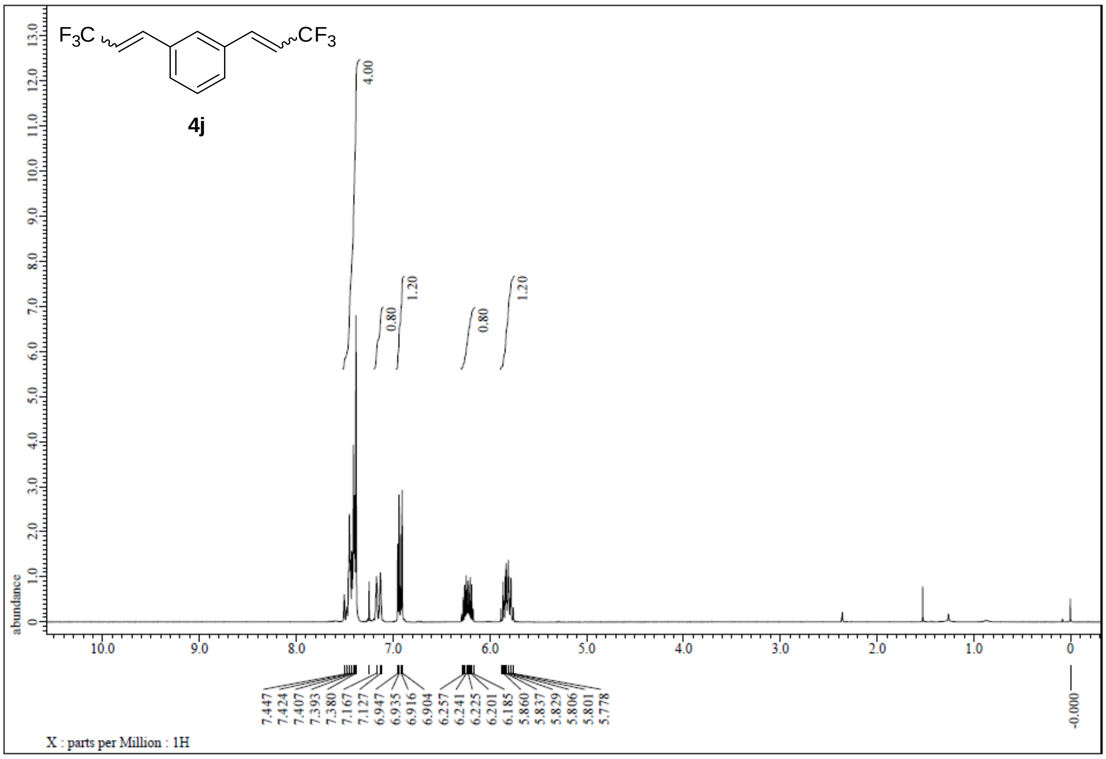

Figure S89. ^{1}H NMR spectrum of 4j, related to Figure 3.

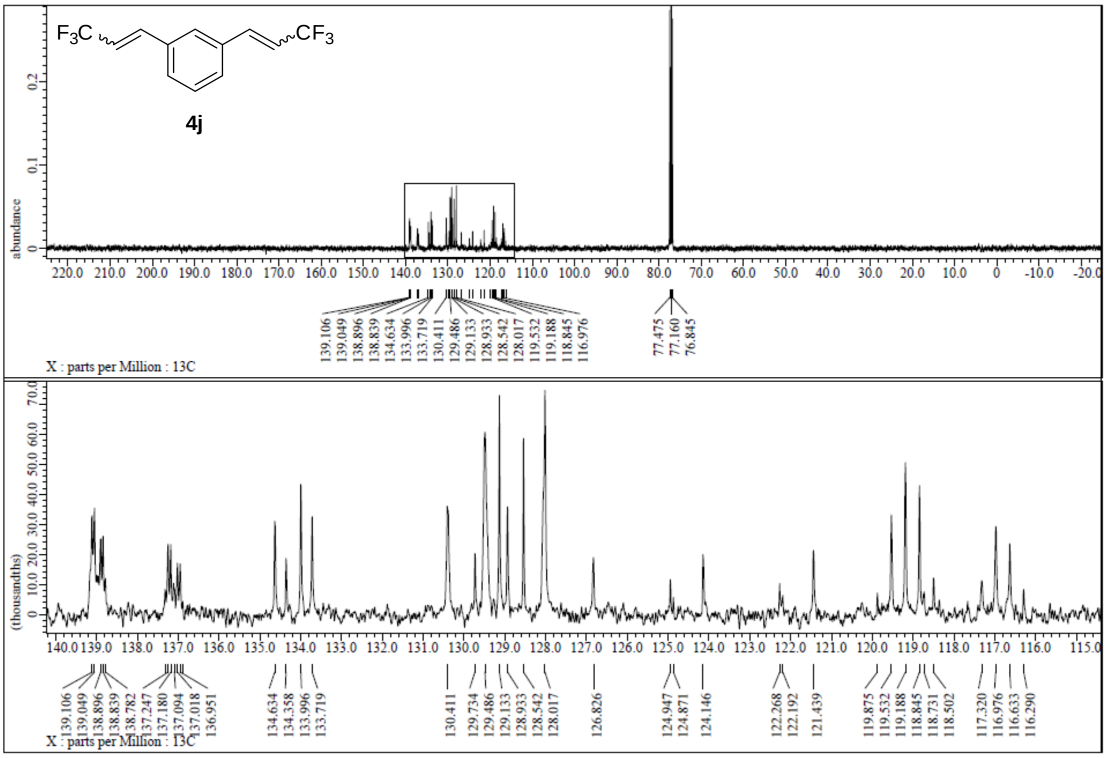

Figure S90. ^{13}C NMR spectrum of 4j, related to Figure 3.

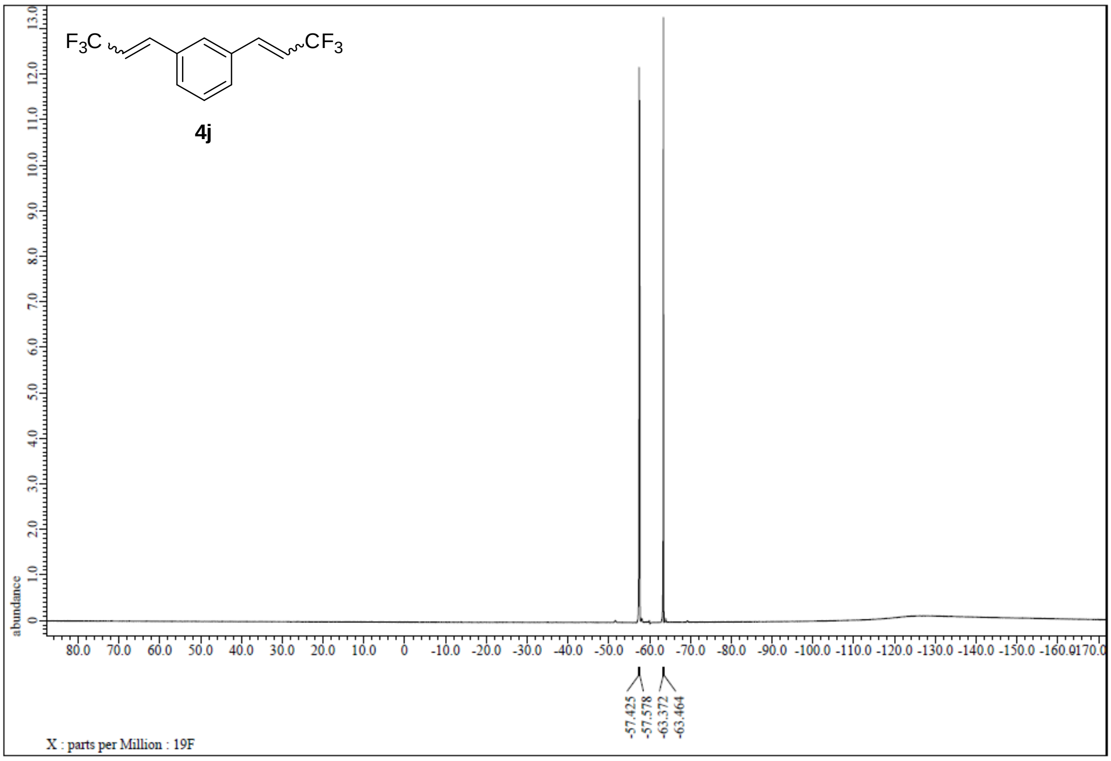

Figure S91. ^{19}F NMR spectrum of 4j, related to Figure 3.

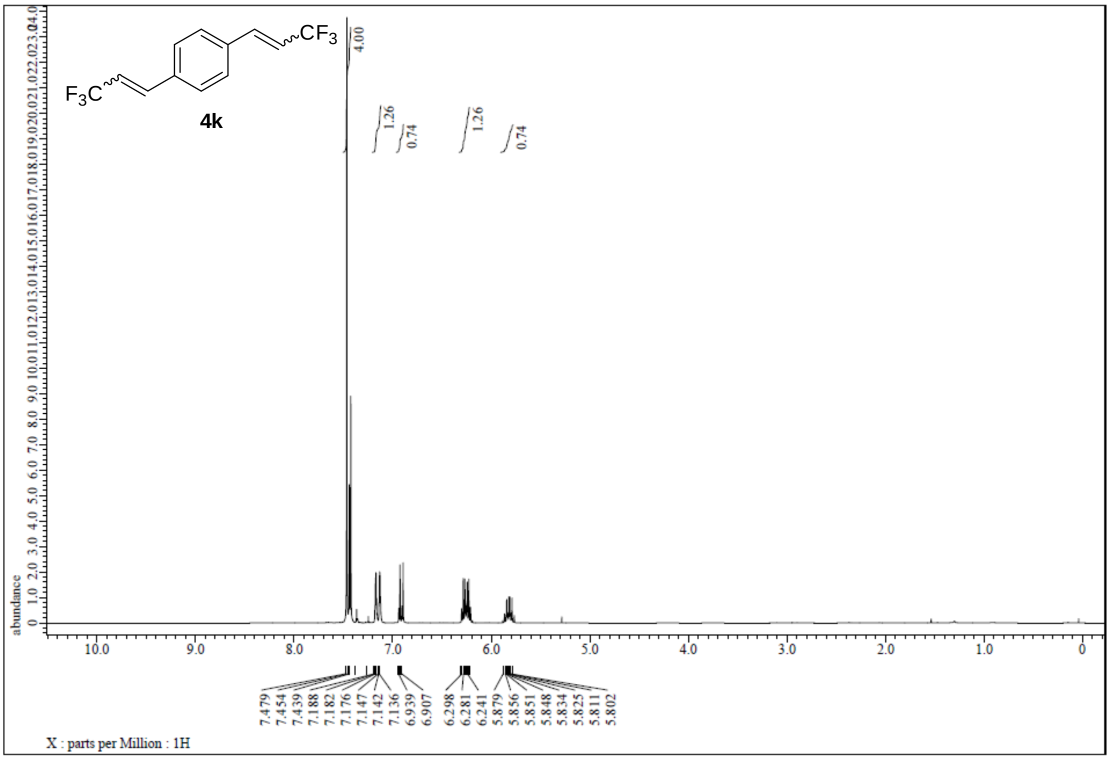

Figure S92. ^{1}H NMR spectrum of 4k, related to Figure 3.

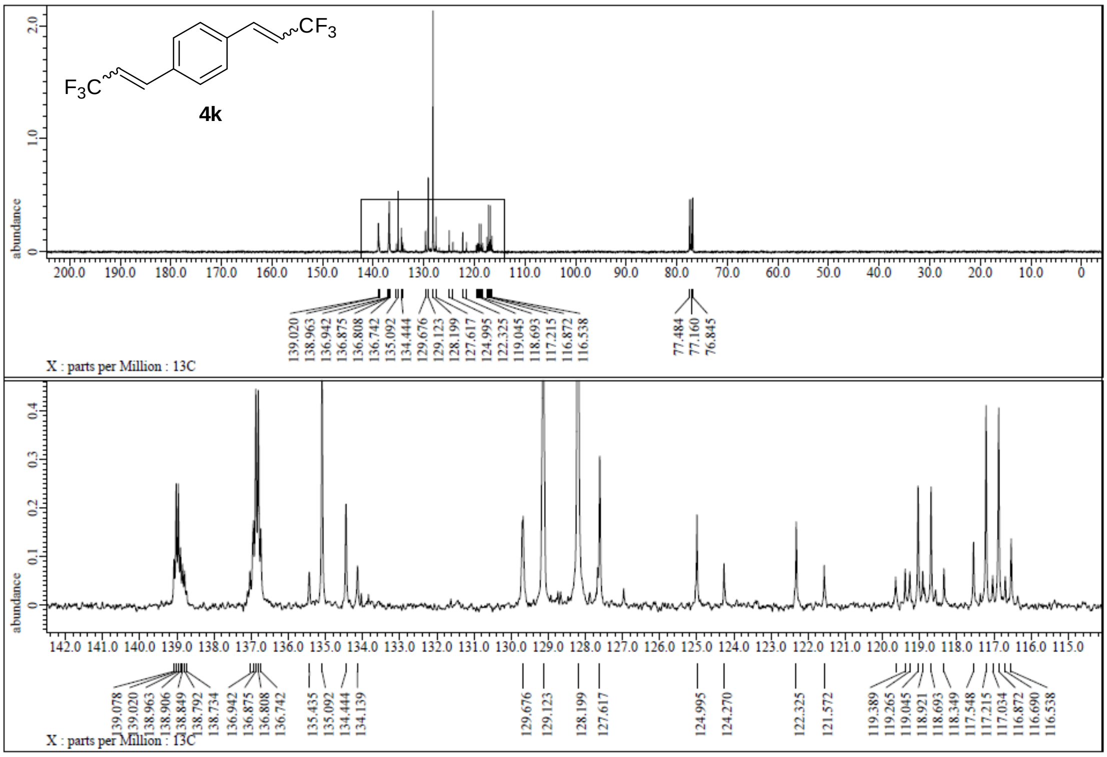

Figure S93. ^{13}C NMR spectrum of 4k, related to Figure 3.

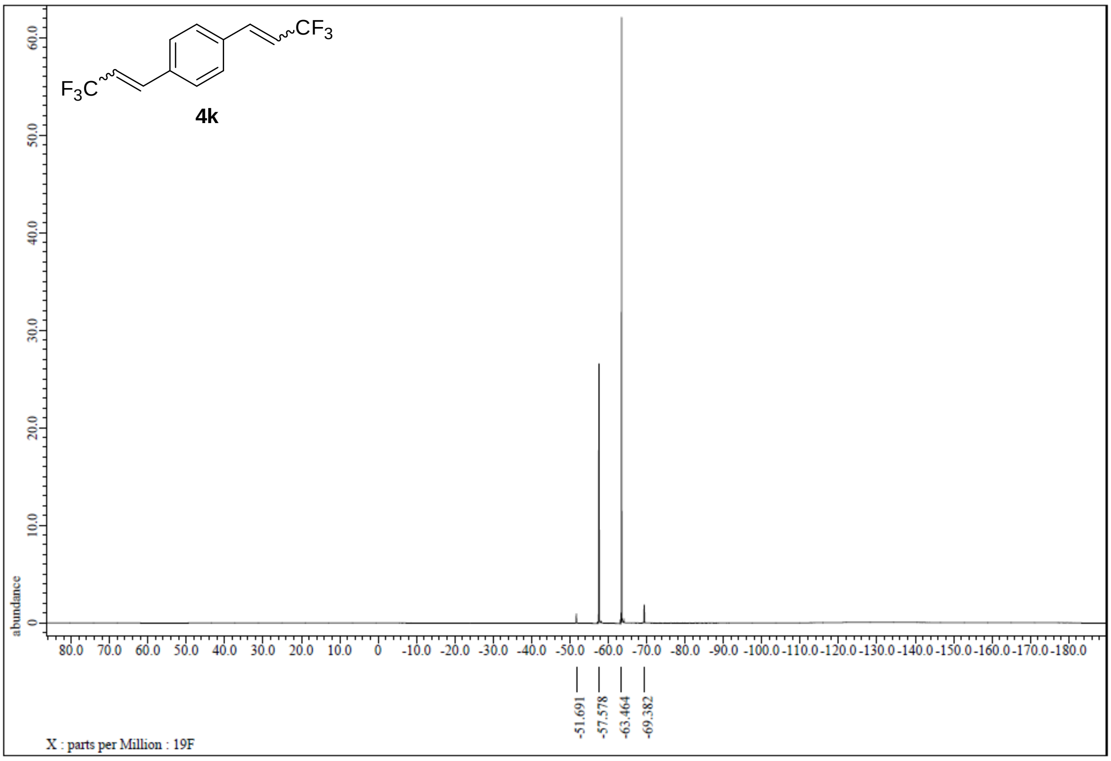

Figure S94. ^{19}F NMR spectrum of 4k, related to Figure 3.

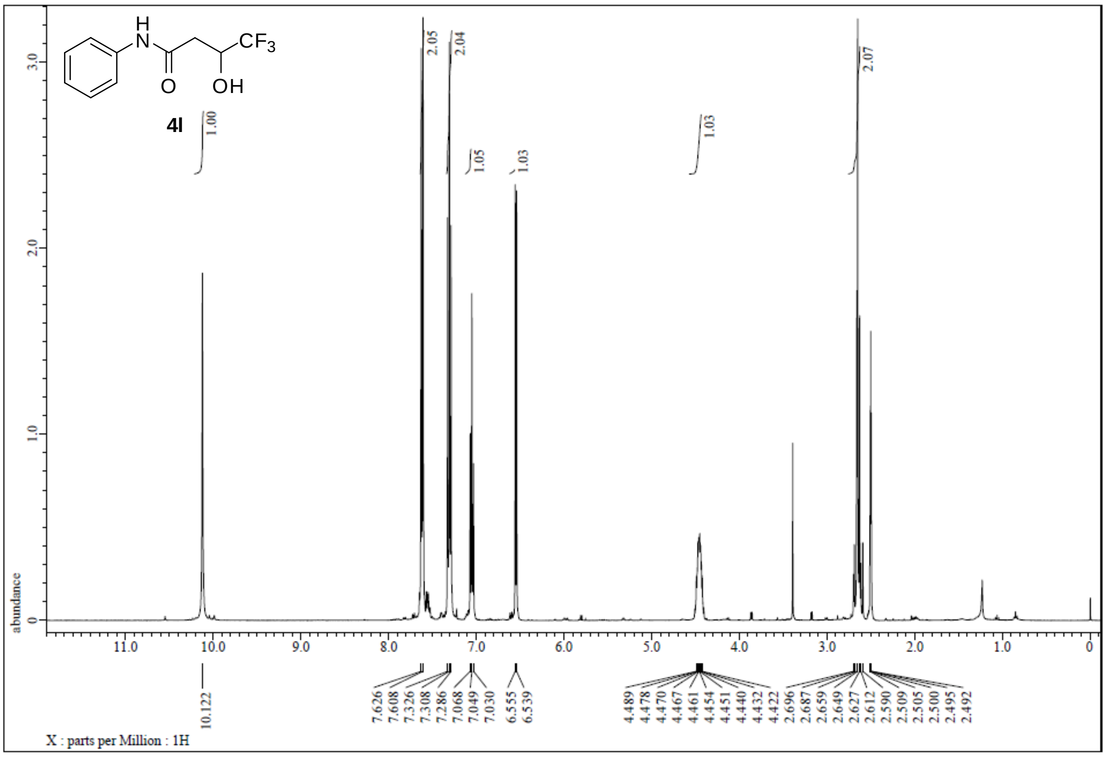

Figure S95. ^{1}H NMR spectrum of 4l, related to Figure 3.

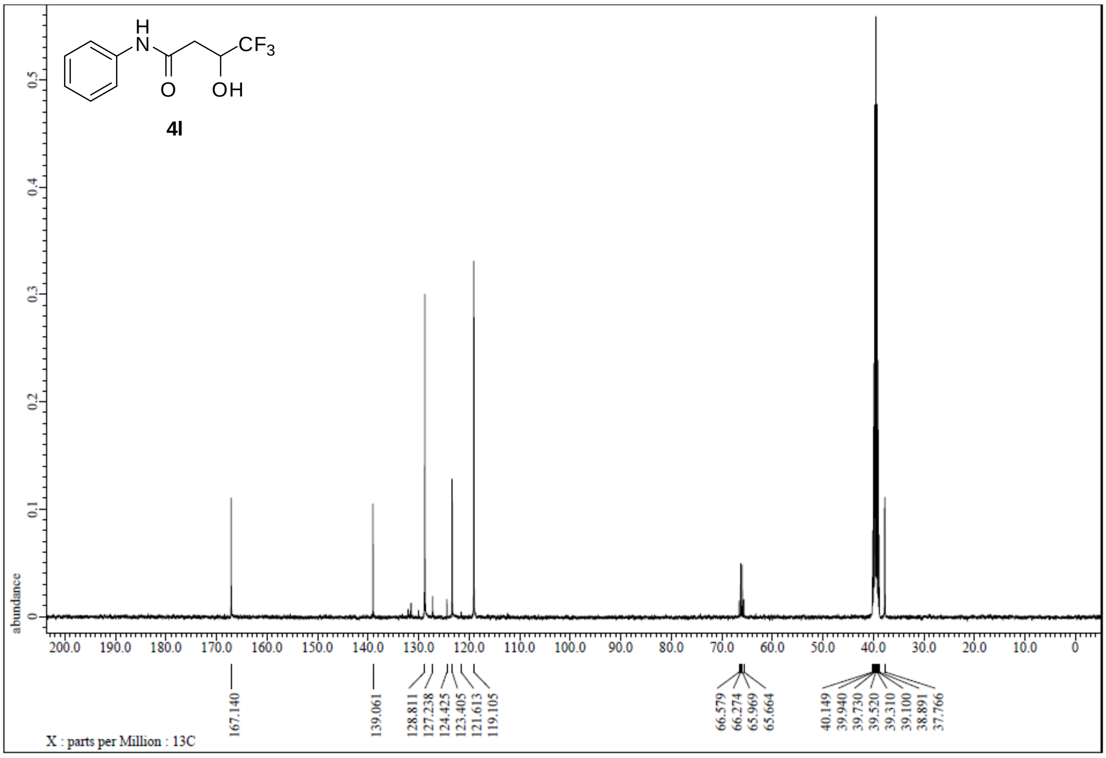

Figure S96. ^{13}C NMR spectrum of 4l, related to Figure 3.

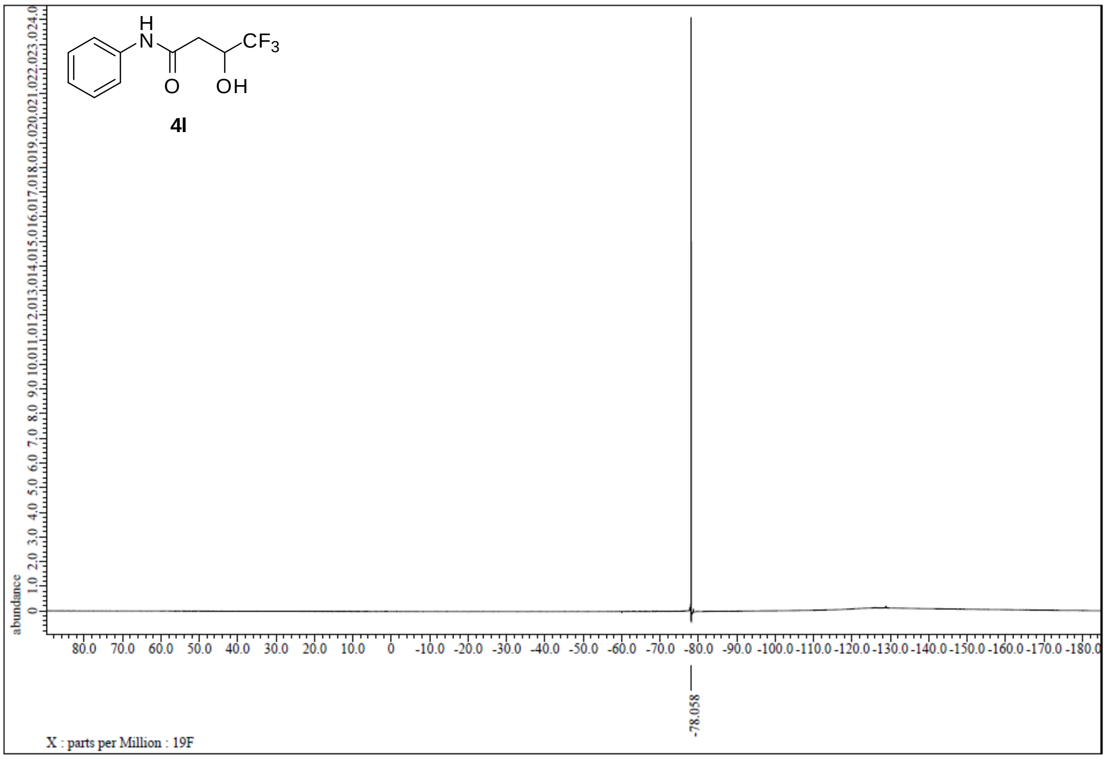

Figure S97. ^{19}F NMR spectrum of 4l, related to Figure 3.

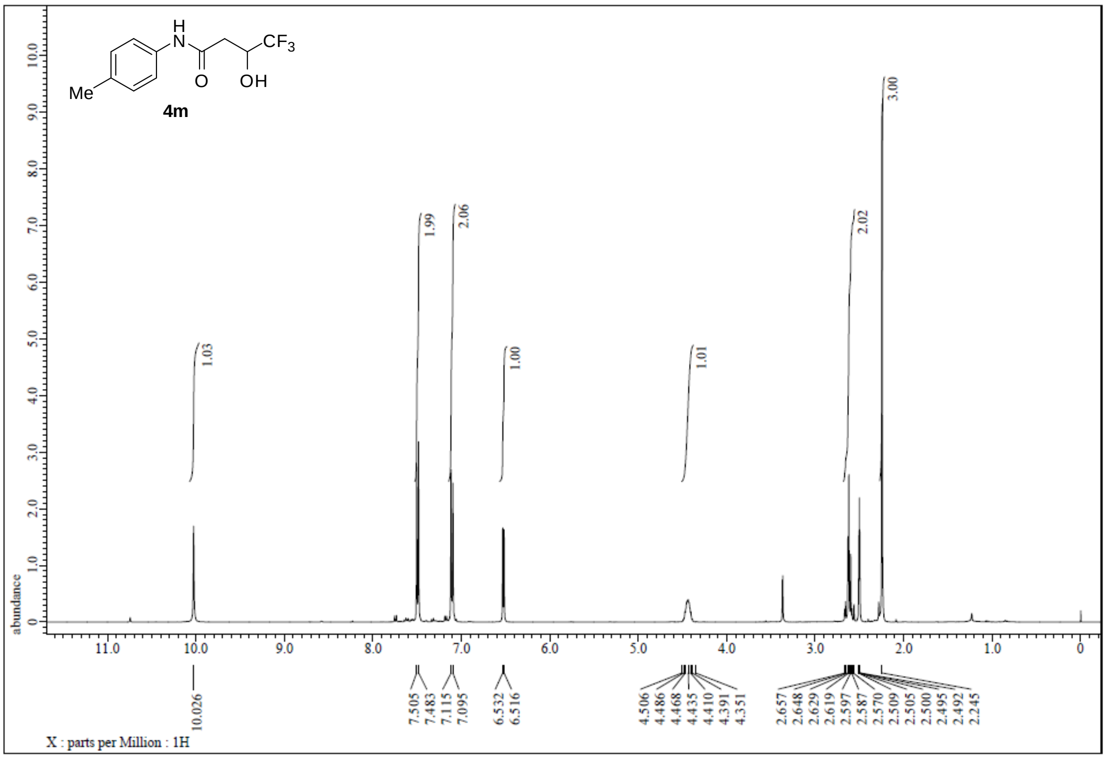

Figure S98. ^{1}H NMR spectrum of 4m, related to Figure 3.

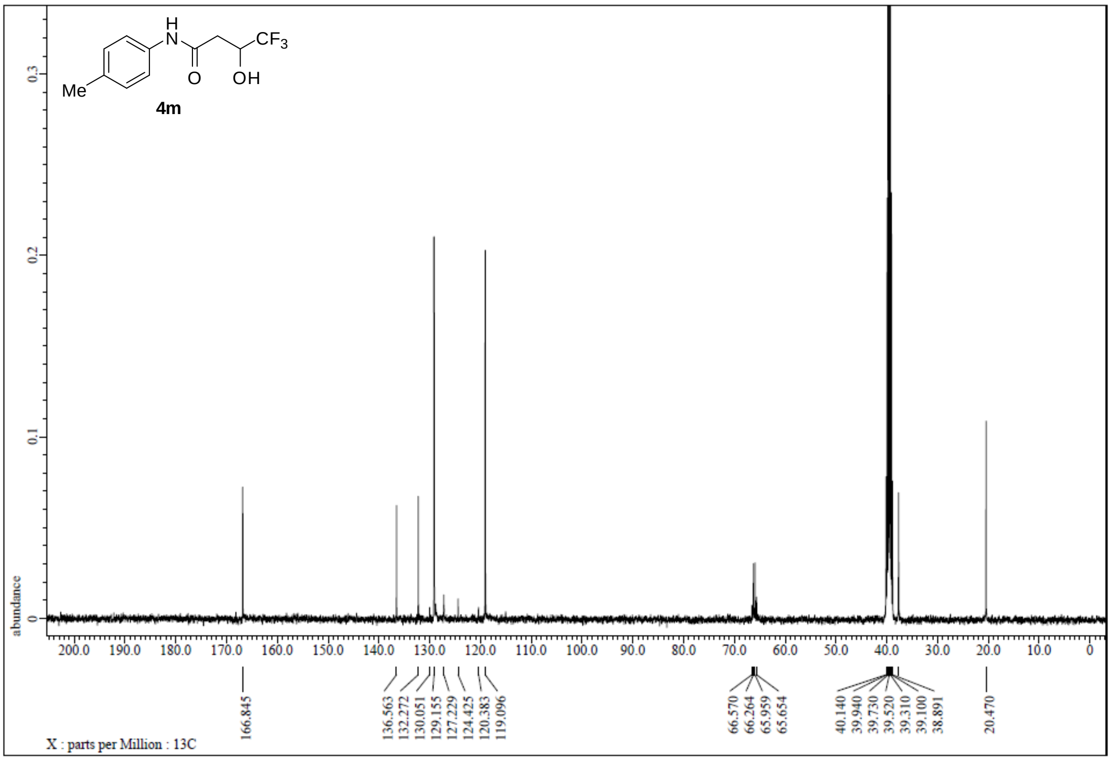

Figure S99. ^{13}C NMR spectrum of 4m, related to Figure 3.

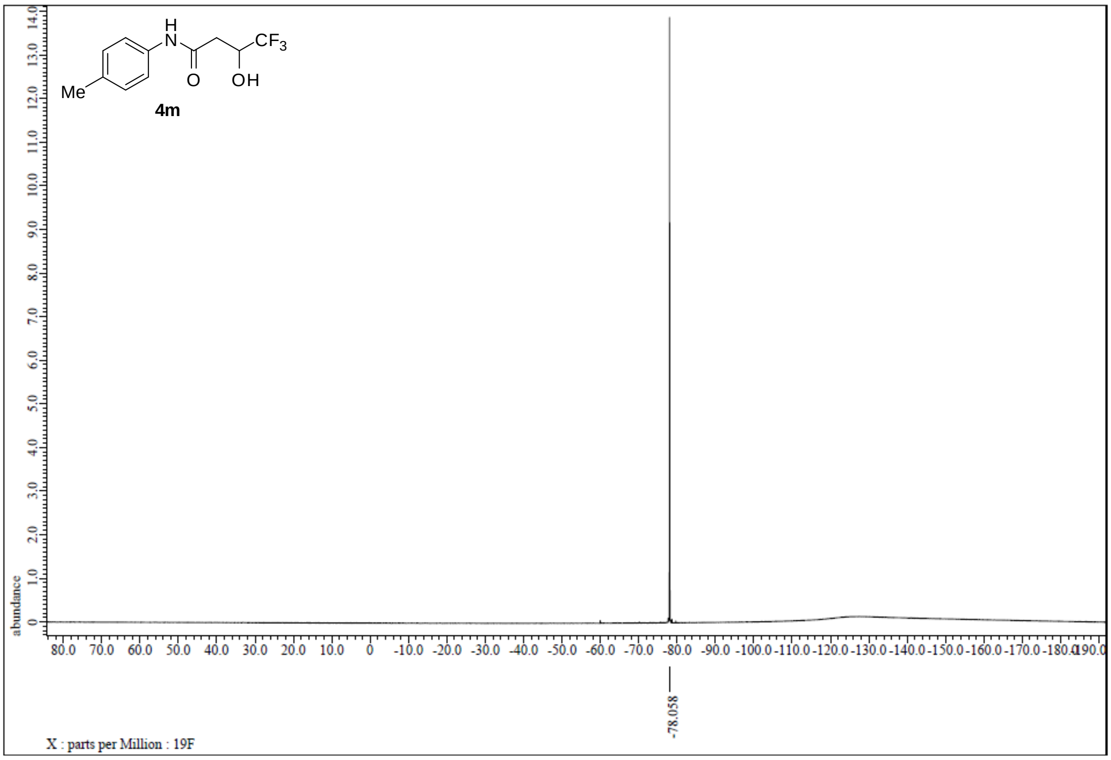

Figure S100. ^{19}F NMR spectrum of 4m, related to Figure 3.

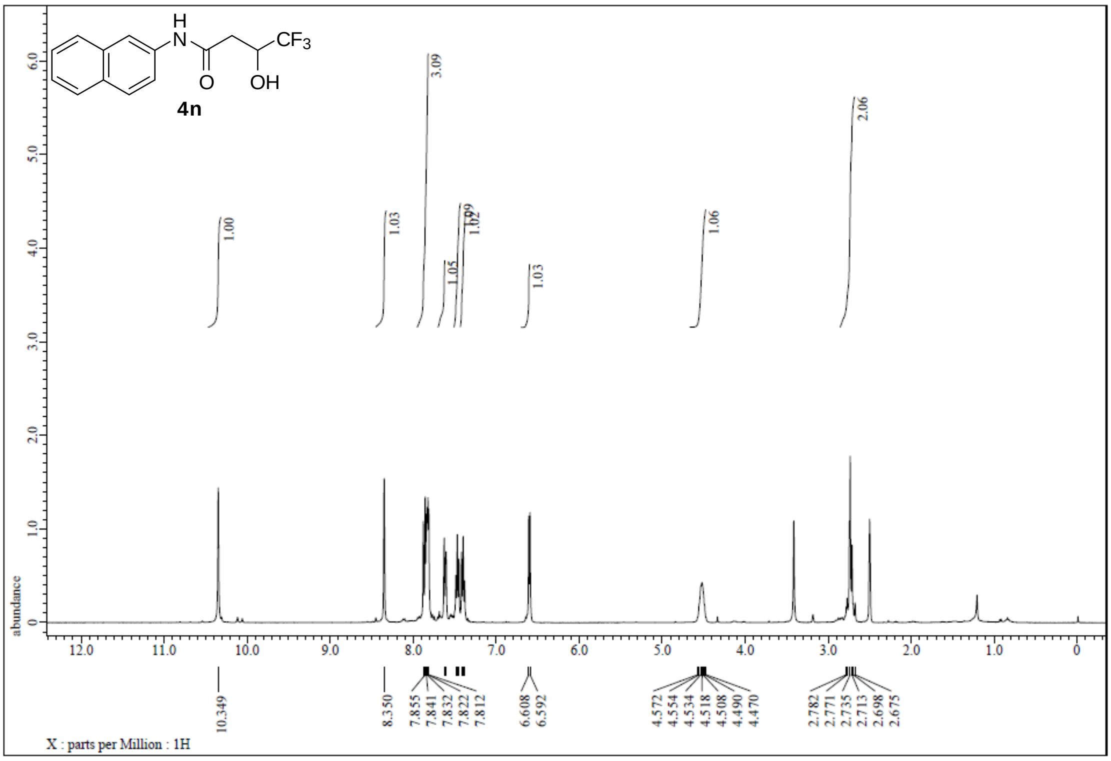

Figure S101. ^{1}H NMR spectrum of 4n, related to Figure 3.

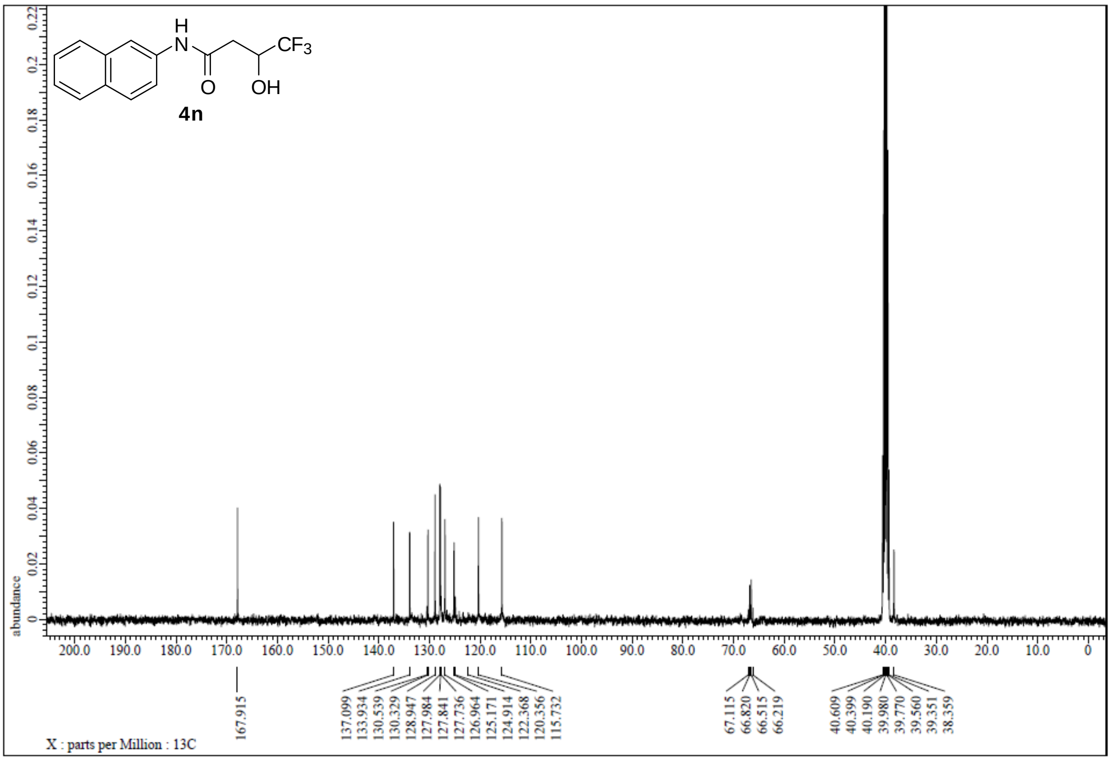

Figure S102. ^{13}C NMR spectrum of 4n, related to Figure 3.

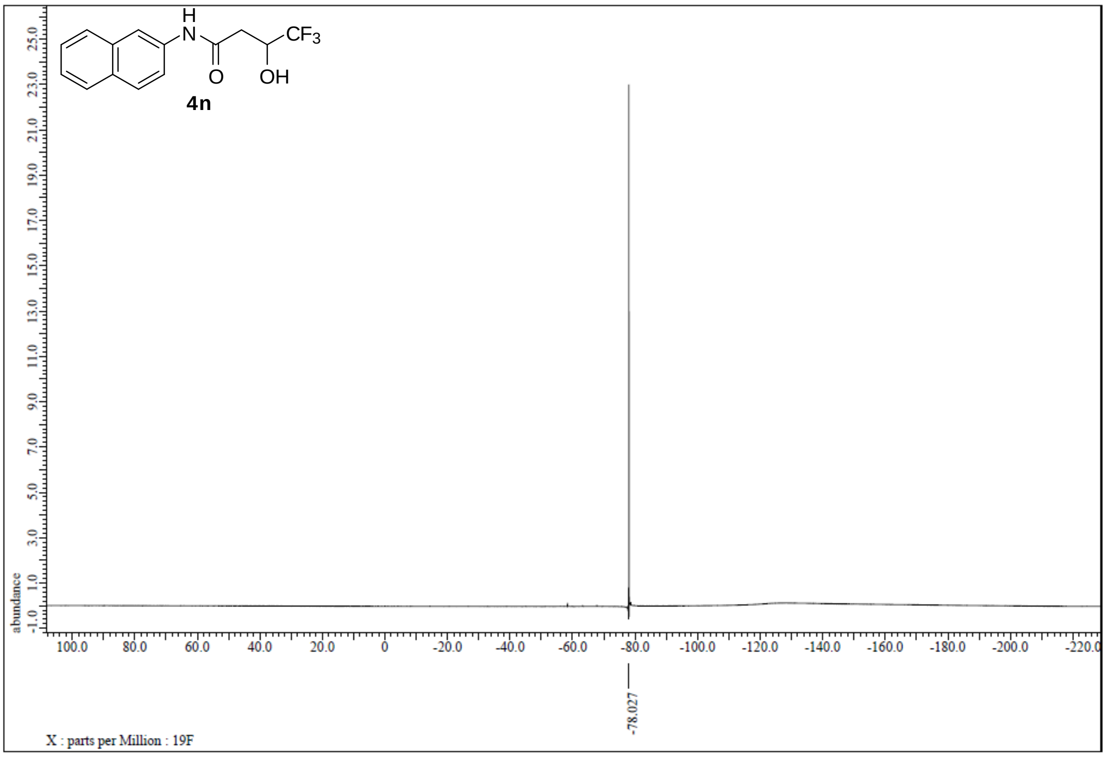

Figure S103. ^{19}F NMR spectrum of 4n, related to Figure 3.

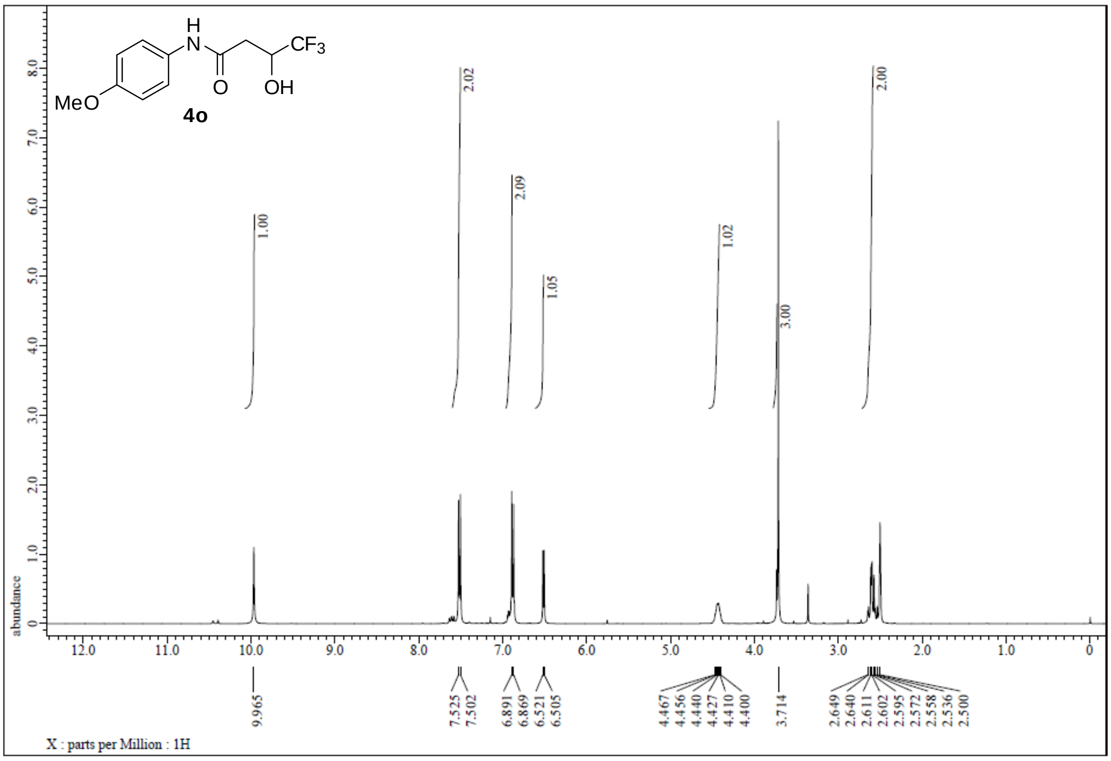

Figure S104. **^{1}H NMR spectrum of 4o, related to Figure 3**.

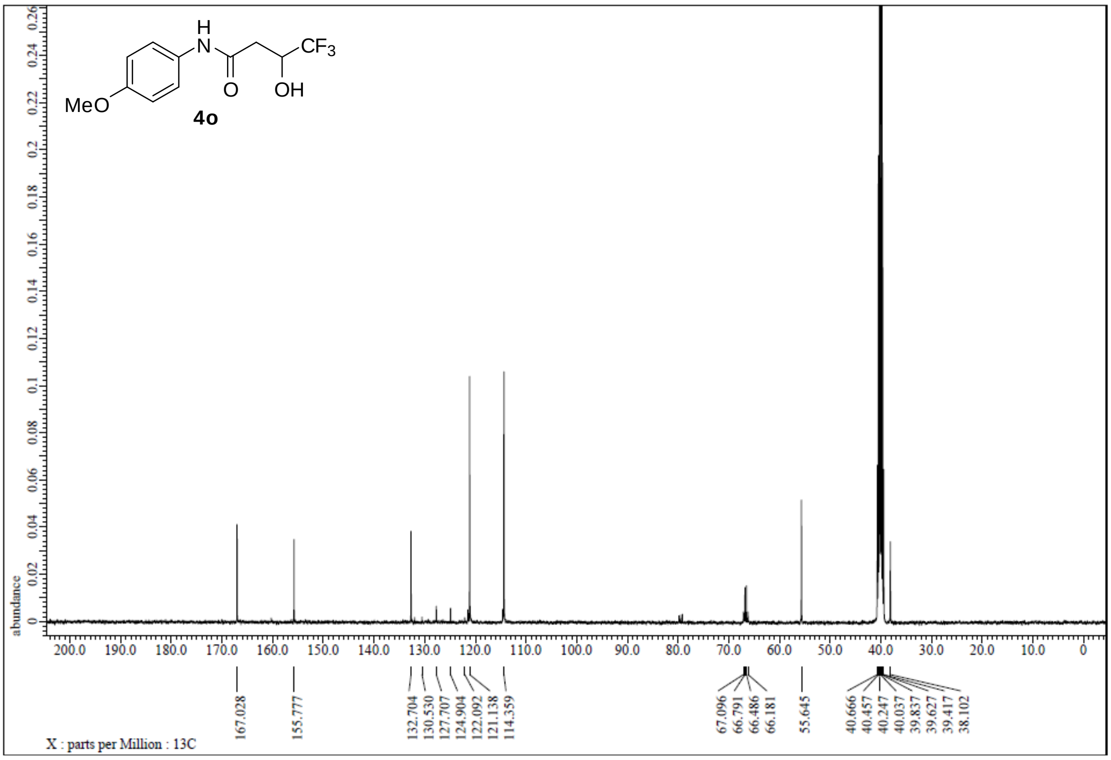

Figure S105. **^{13}C NMR spectrum of 4o, related to Figure 3**.

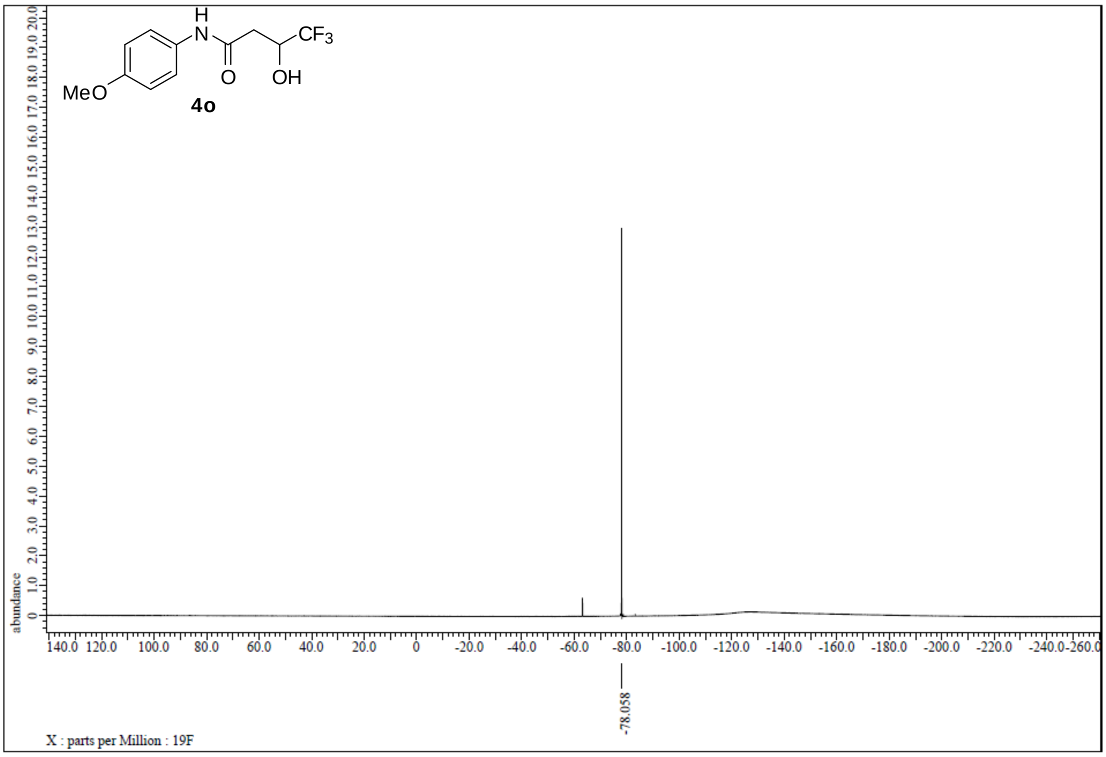

Figure S106. ^{19}F NMR spectrum of 4o, related to Figure 3.

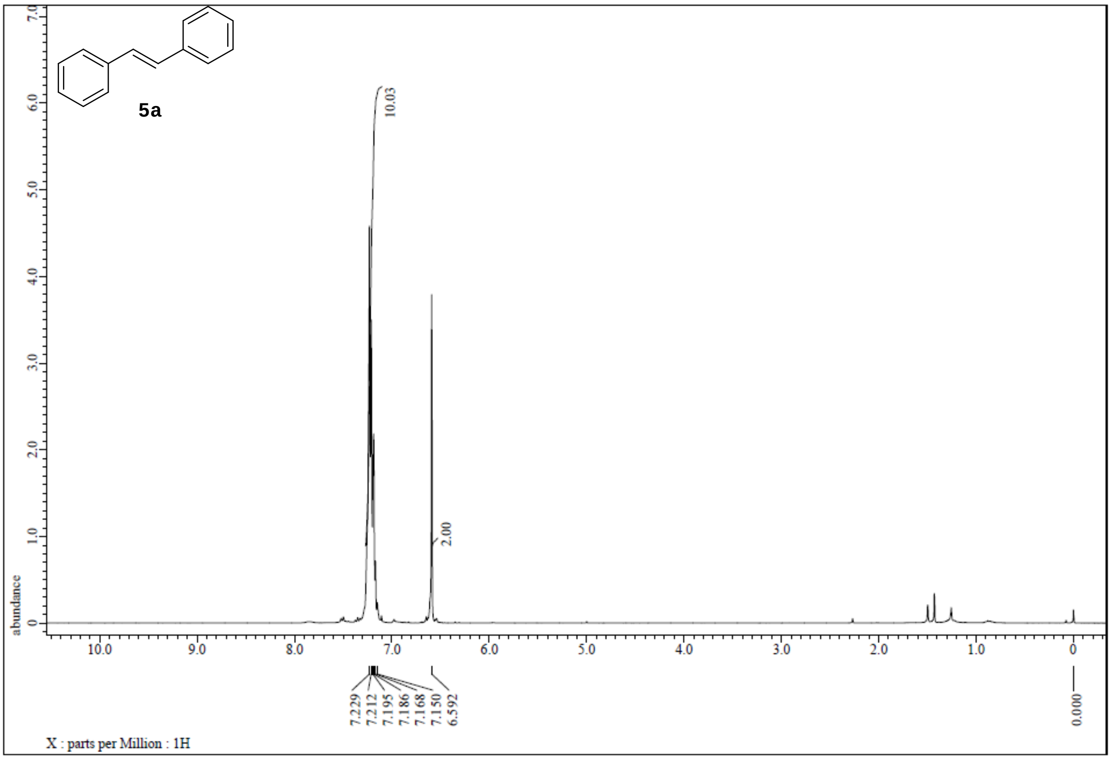

Figure S107. ^{1}H **NMR spectrum of 5a, related to Figure 4**.

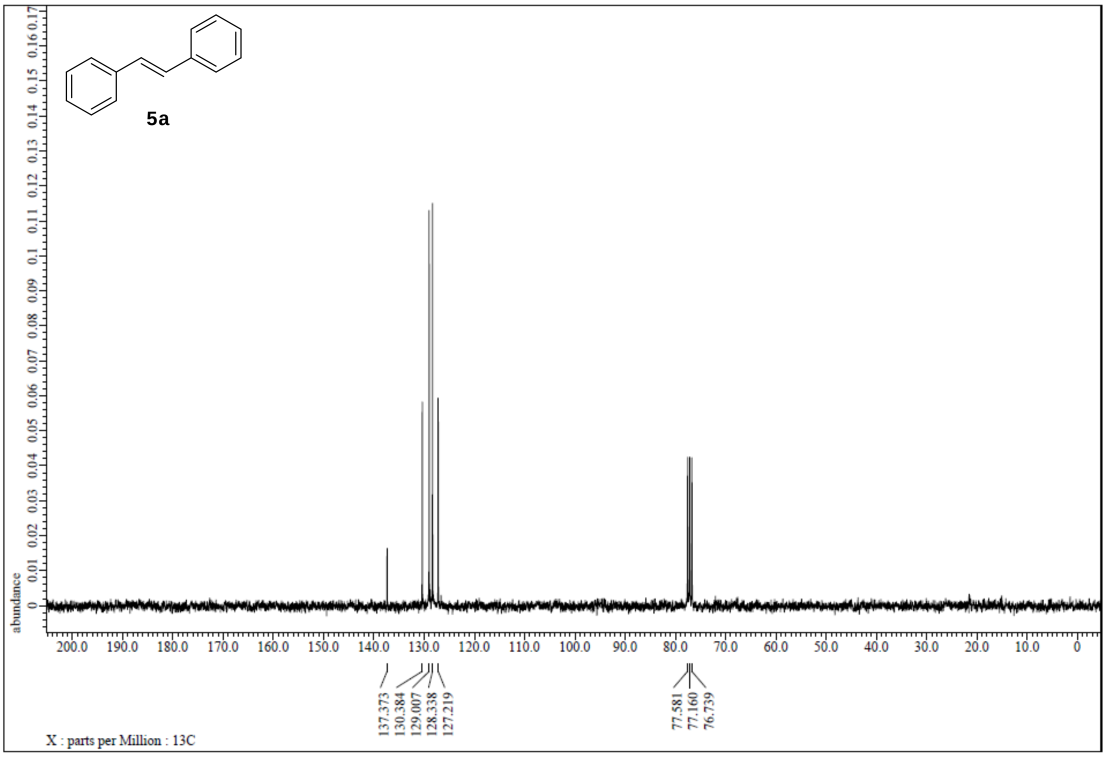

Figure S108. ^{13}C **NMR spectrum of 5a, related to Figure 4**.

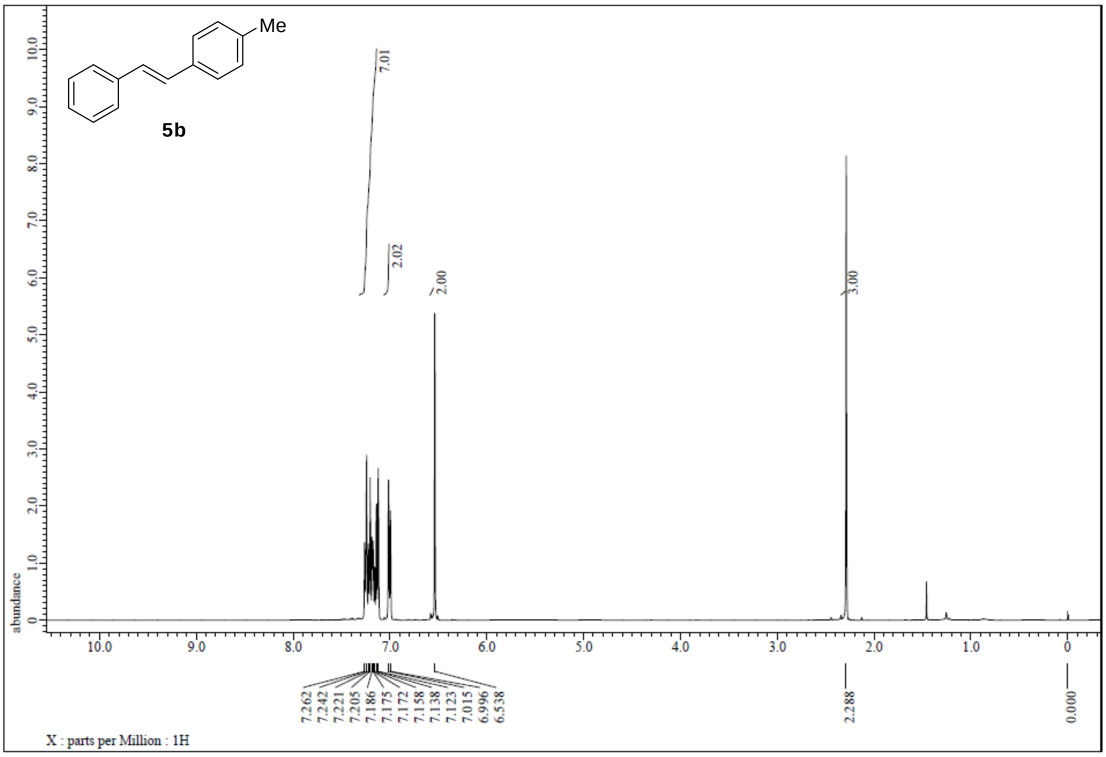

Figure S109. ^{1}H NMR spectrum of 5b, related to Figure 4.

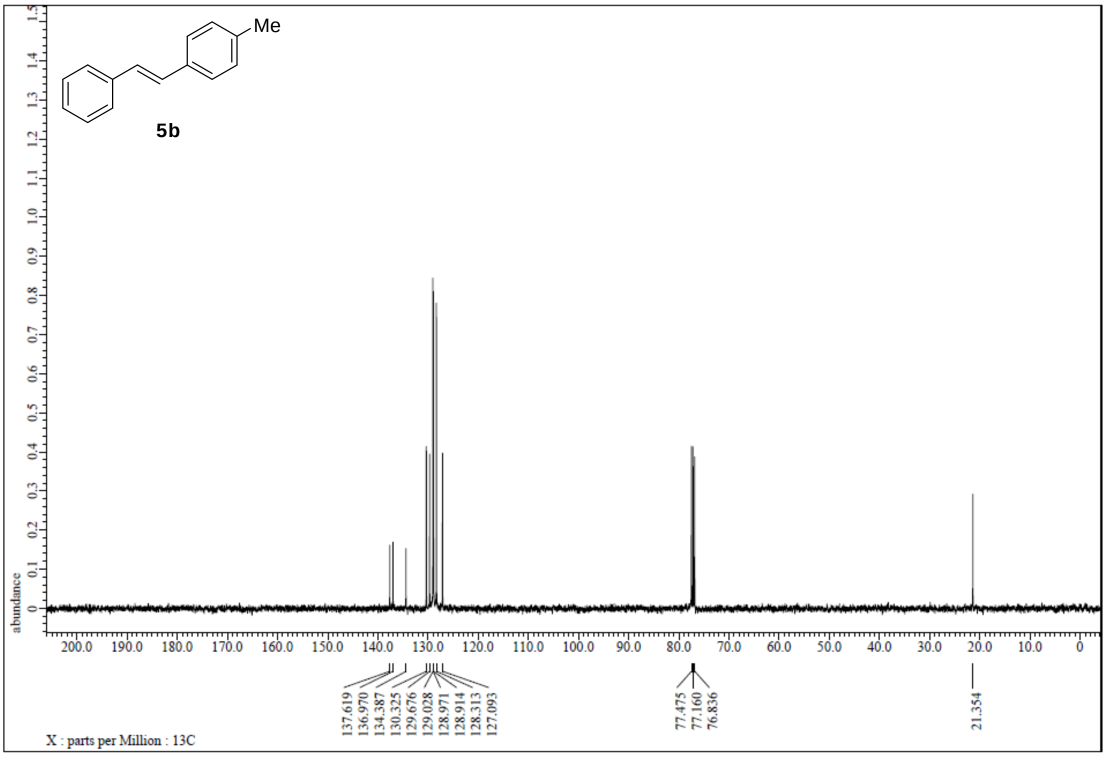

Figure S110. ^{13}C NMR spectrum of 5b, related to Figure 4.

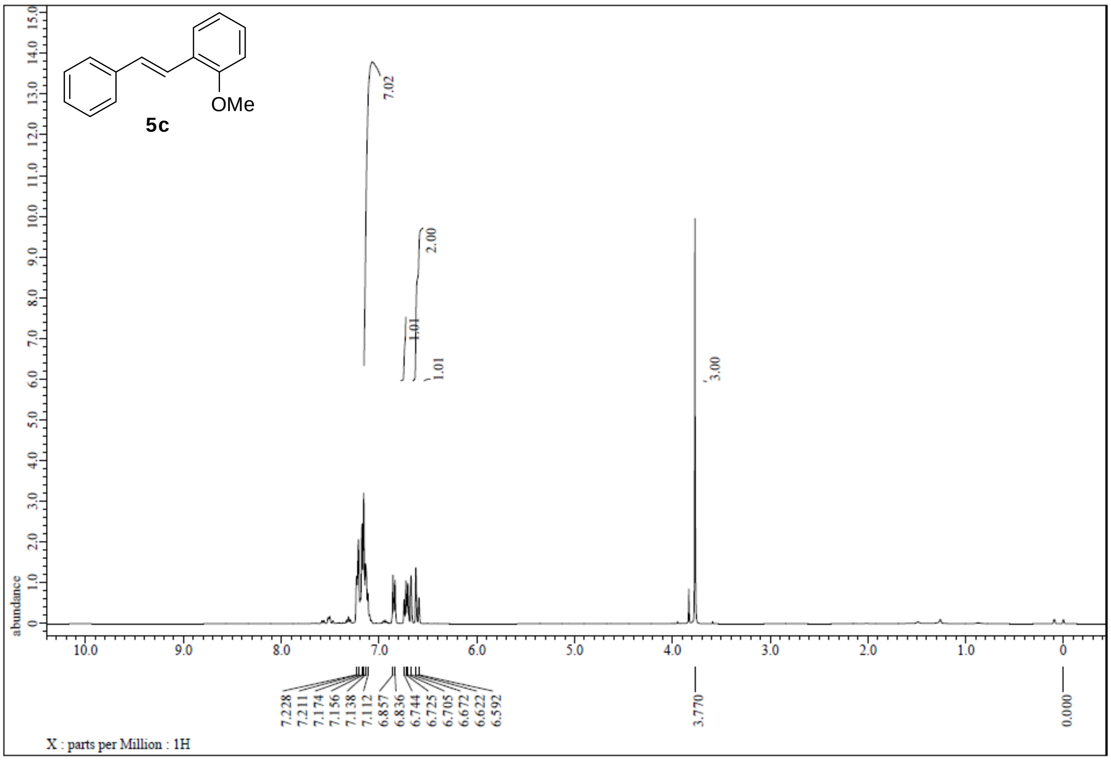

Figure S111. **^{1}H NMR spectrum of 5c, related to Figure 4**.

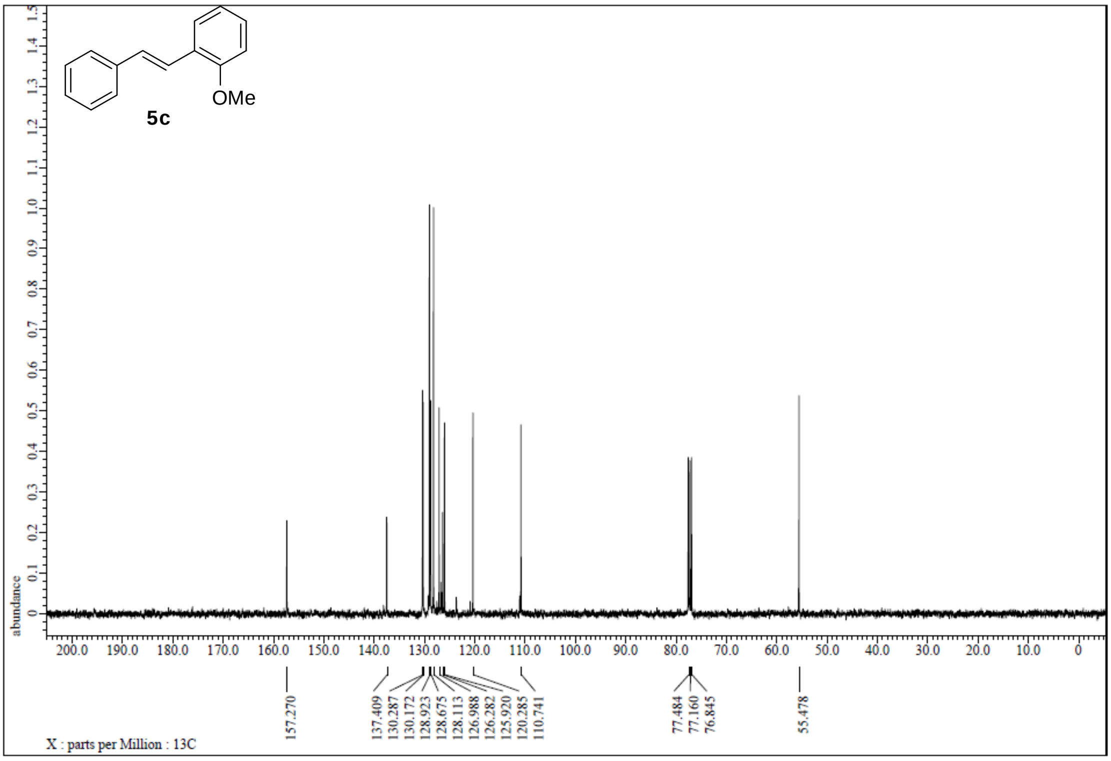

Figure S112. **^{13}C NMR spectrum of 5c, related to Figure 4**.

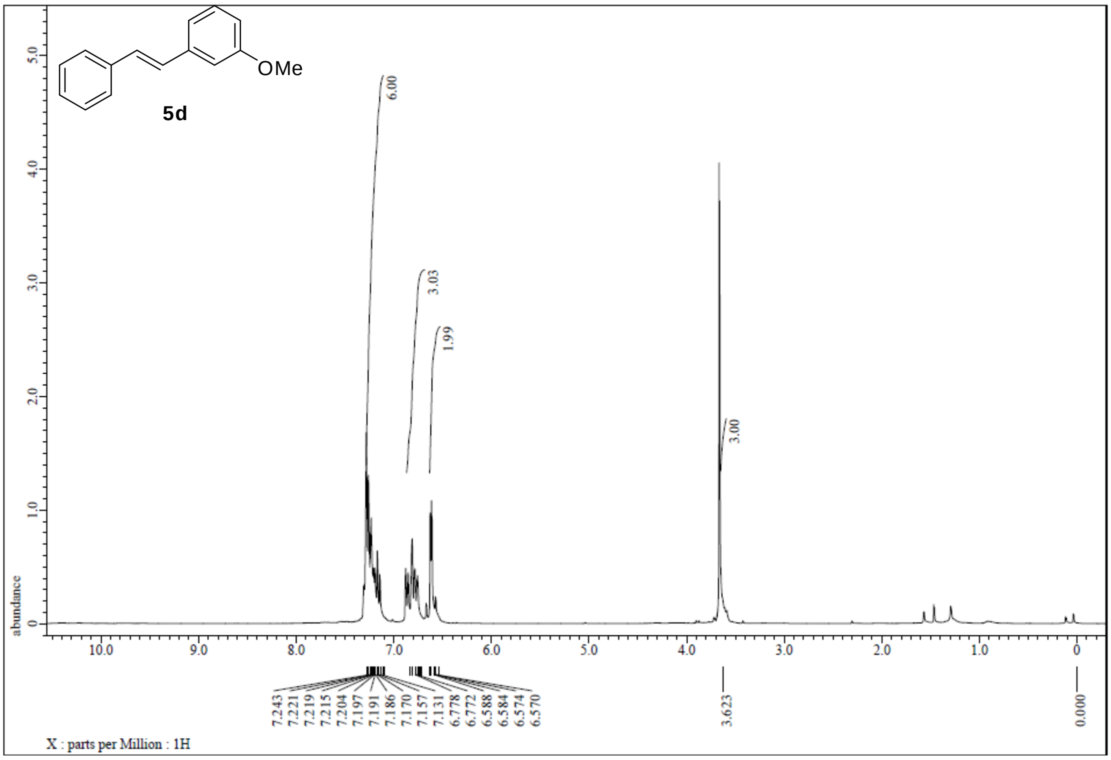

Figure S113. **^{1}H NMR spectrum of 5d, related to Figure 4**.

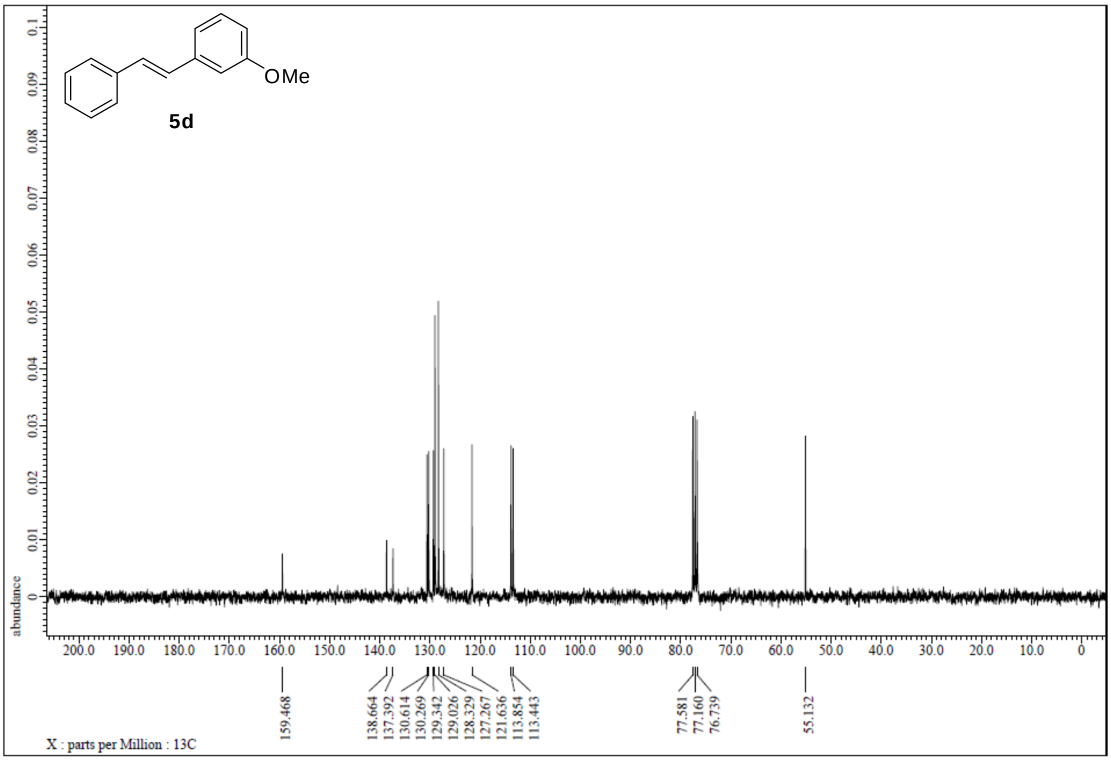

Figure S114. **^{13}C NMR spectrum of 5d, related to Figure 4**.

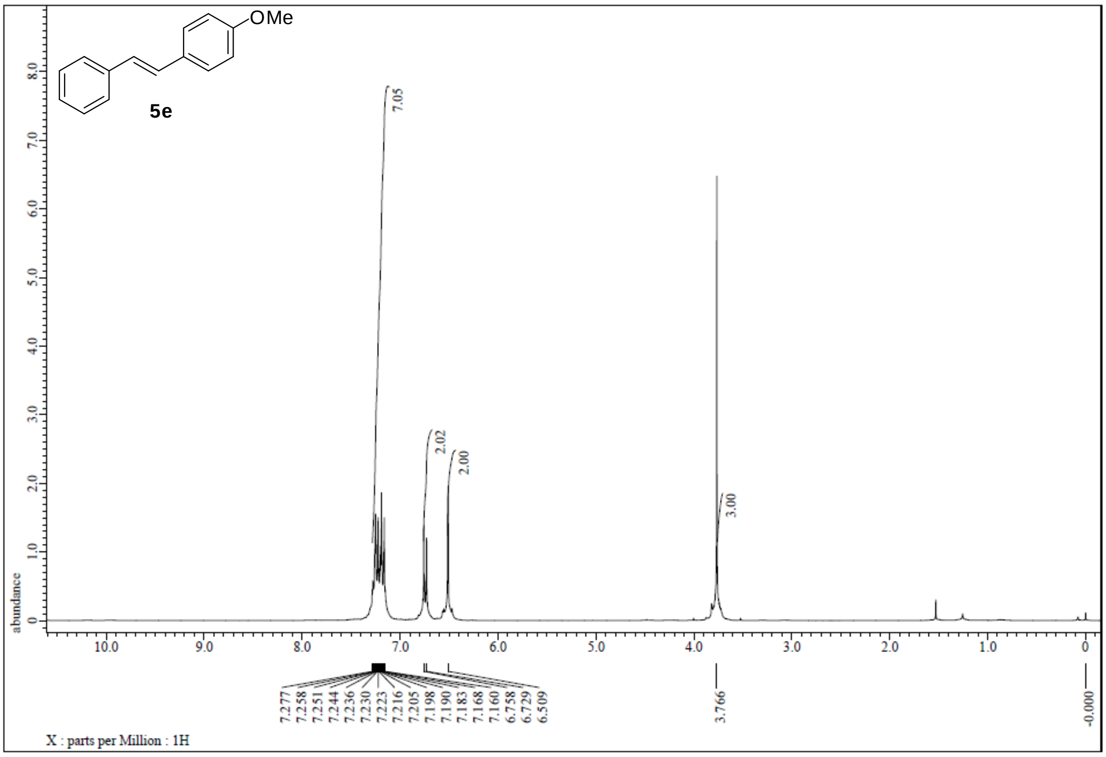

Figure S115. ^{1}H NMR spectrum of 5e, related to Figure 4.

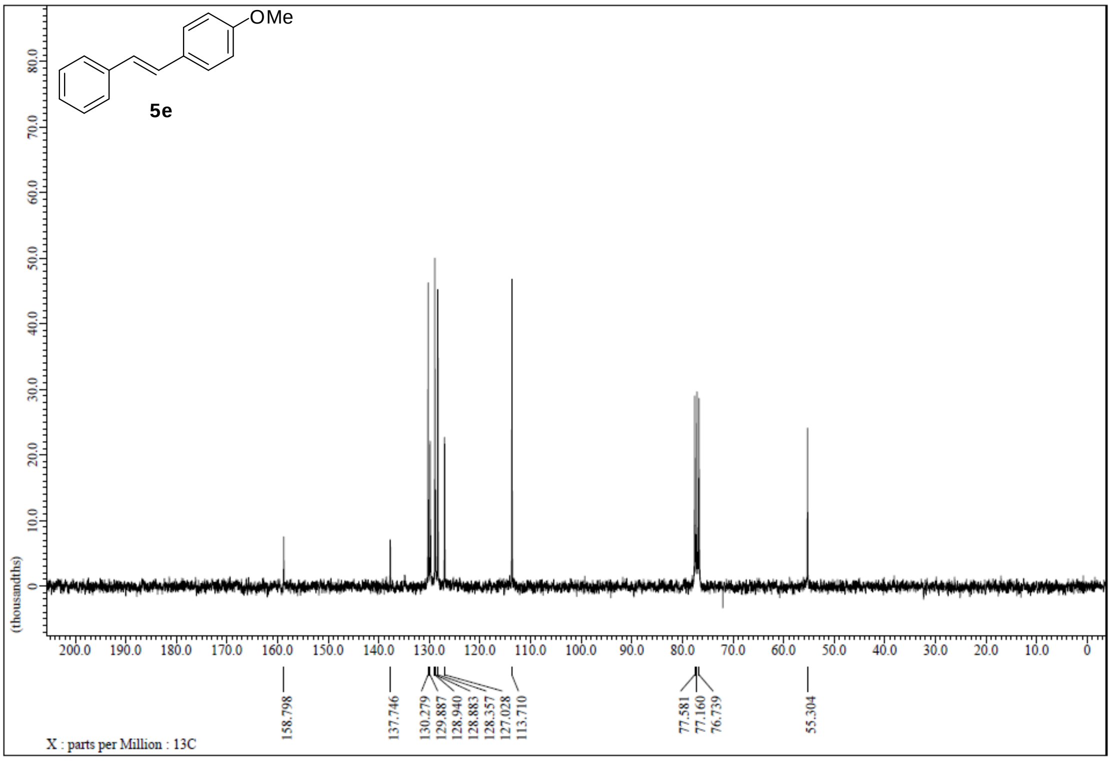

Figure S116. ^{13}C NMR spectrum of 5e, related to Figure 4.

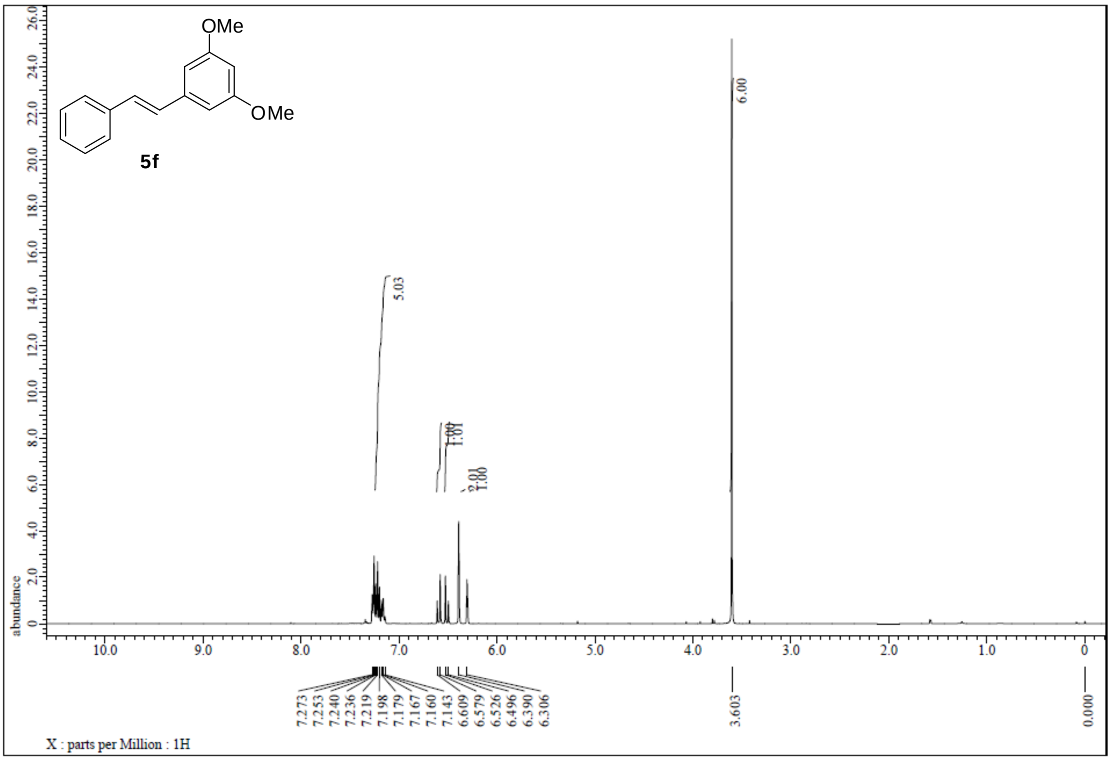

Figure S117. ^{1}H NMR spectrum of 5f, related to Figure 4.

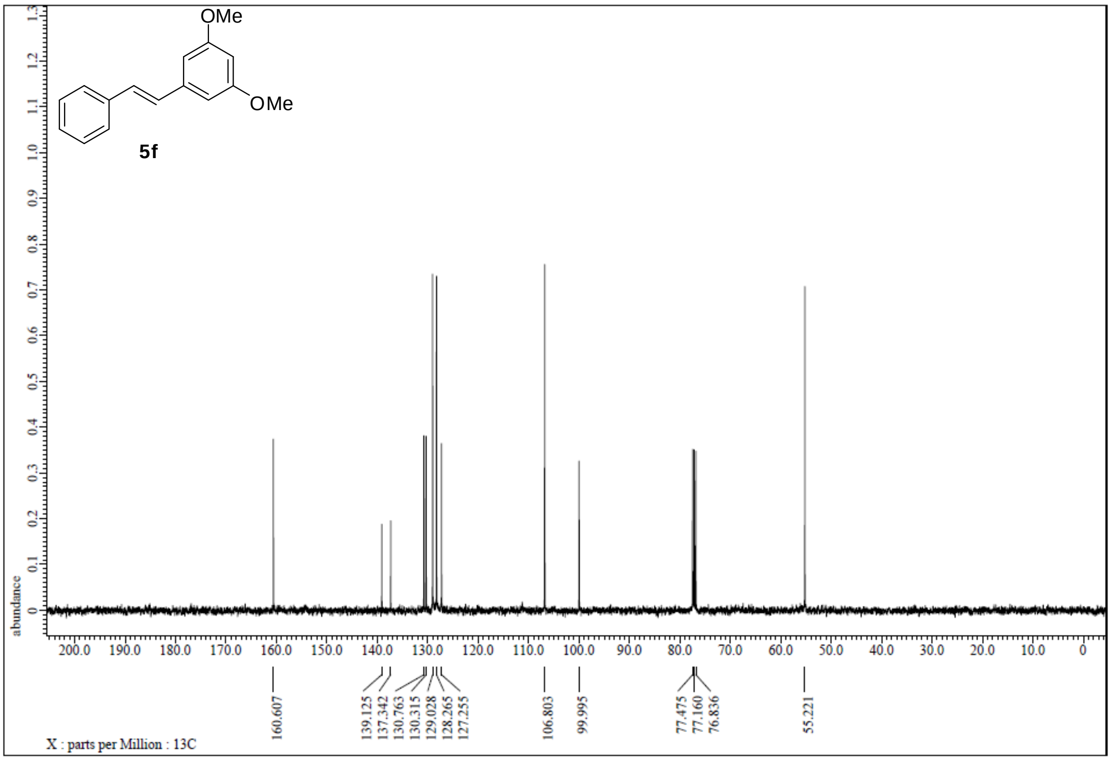

Figure S118. ^{13}C NMR spectrum of 5f, related to Figure 4.

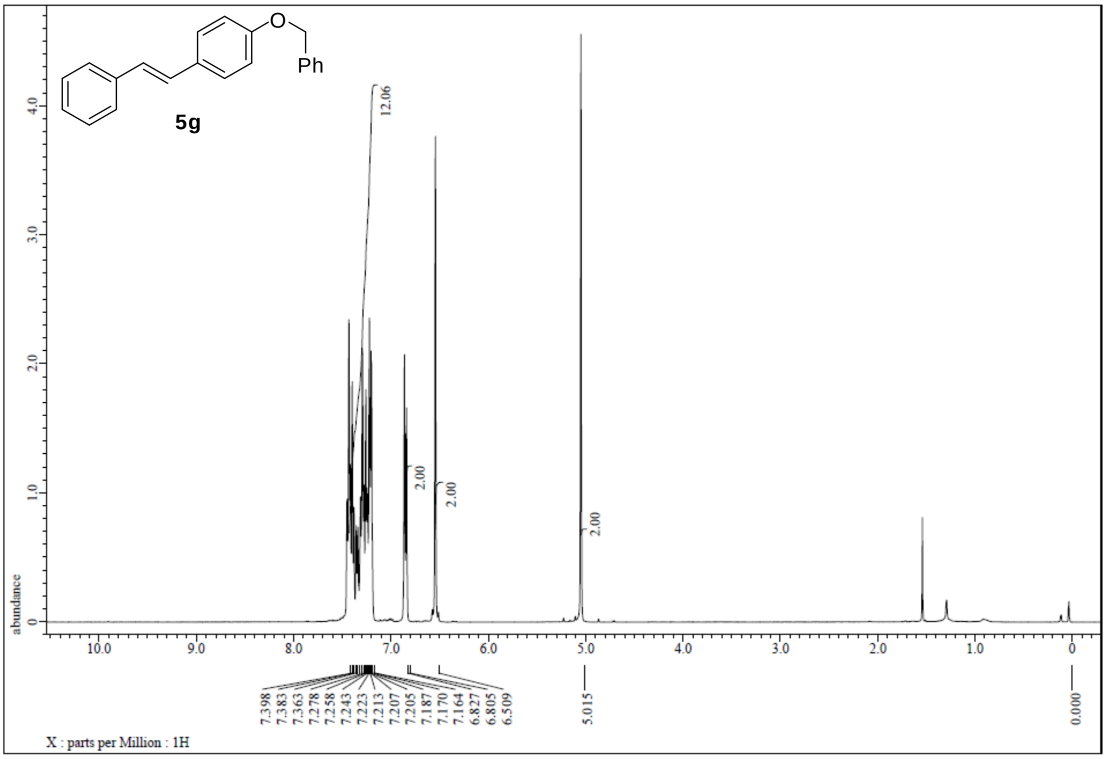

Figure S119. ^{1}H NMR spectrum of 5g, related to Figure 4.

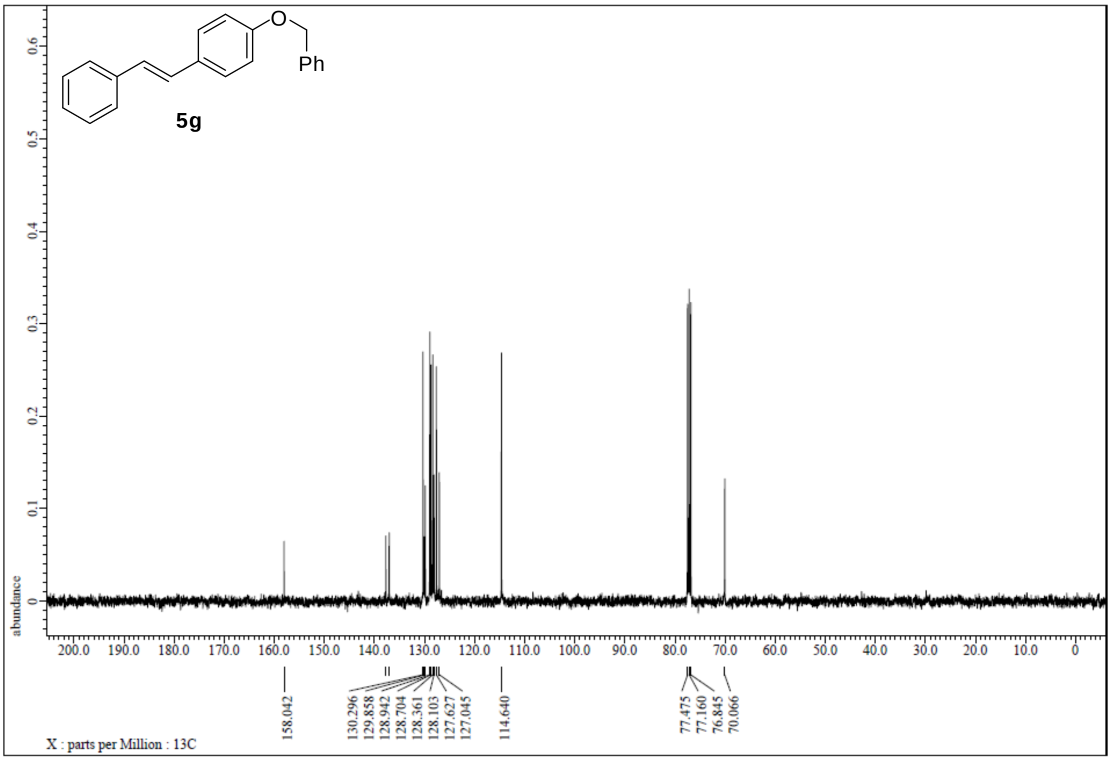

Figure S120. ^{13}C NMR spectrum of 5g, related to Figure 4.

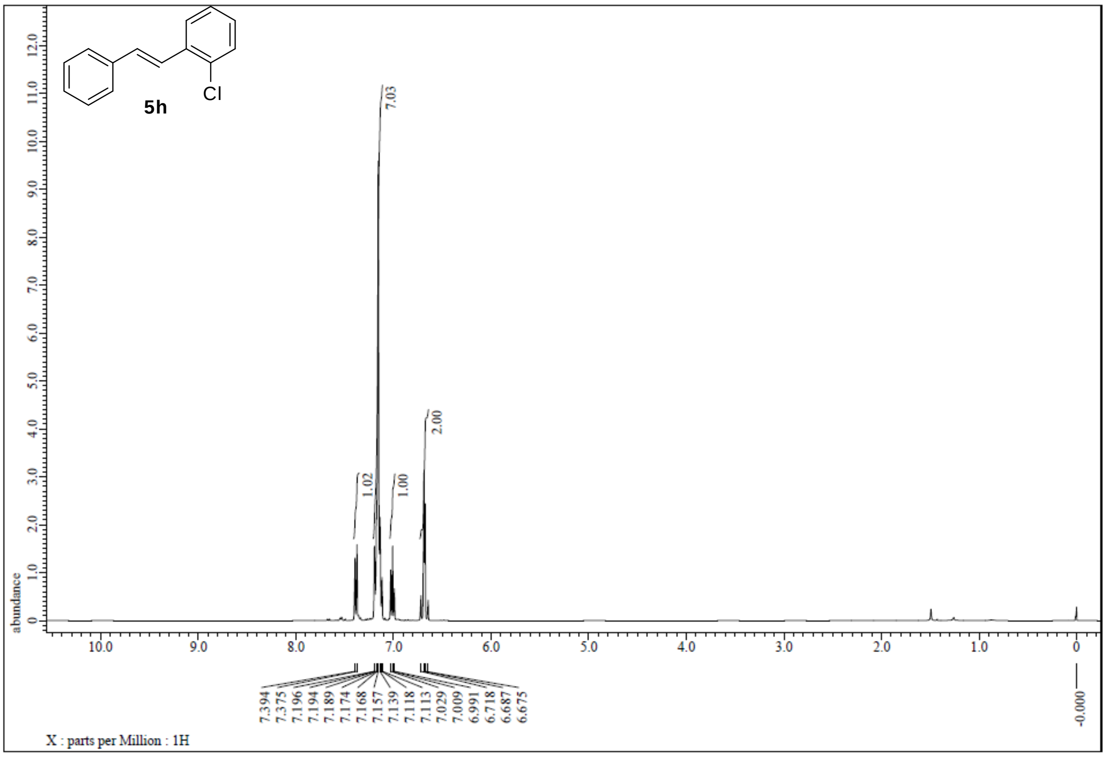

Figure S121. ^{1}H NMR spectrum of 5h, related to Figure 4.

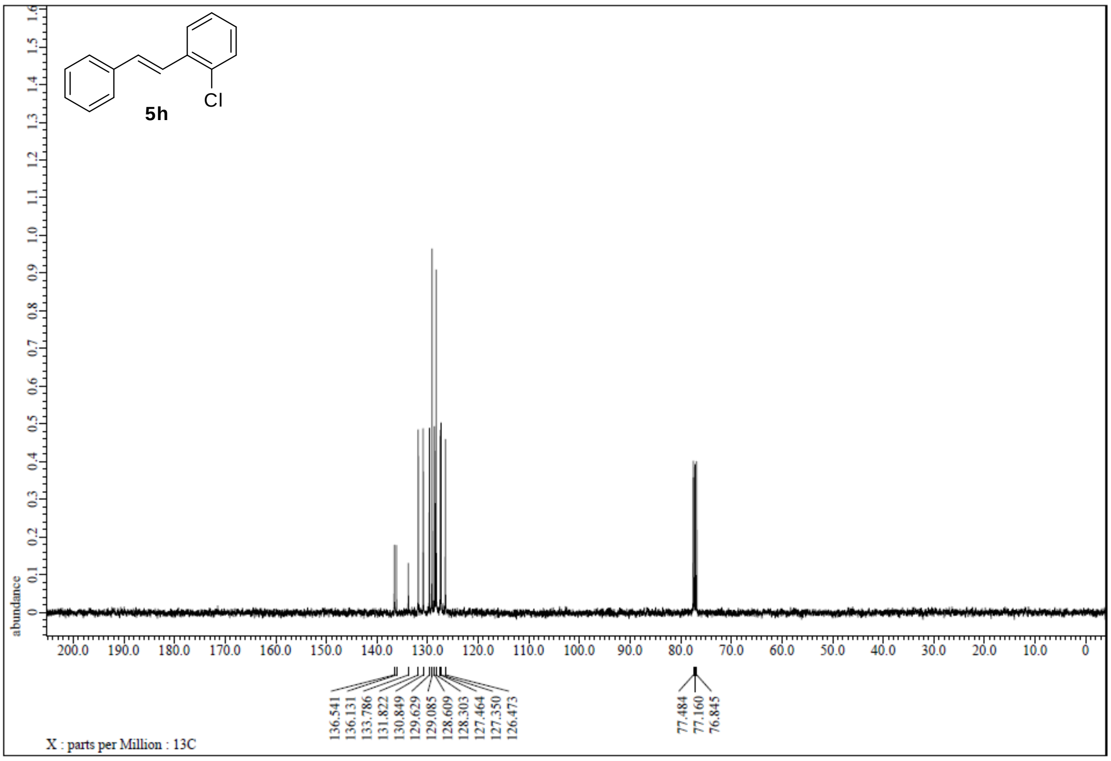

Figure S122. ^{13}C NMR spectrum of 5h, related to Figure 4.

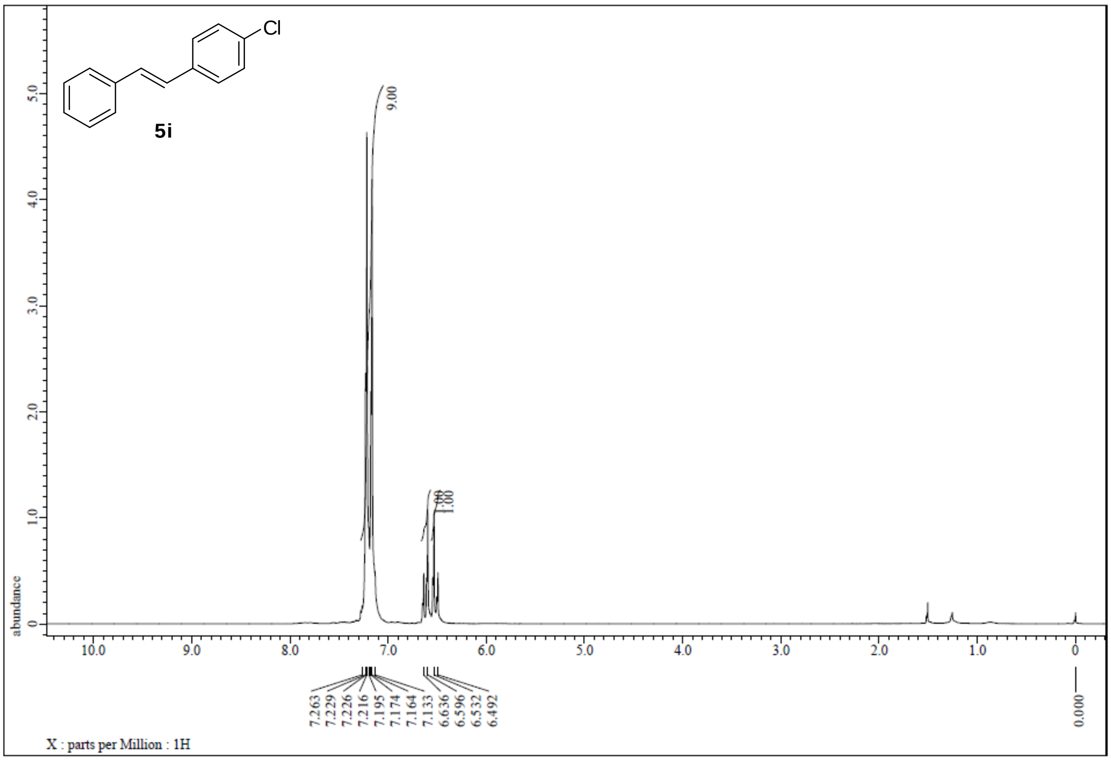

Figure S123. ^{1}H NMR spectrum of 5i, related to Figure 4.

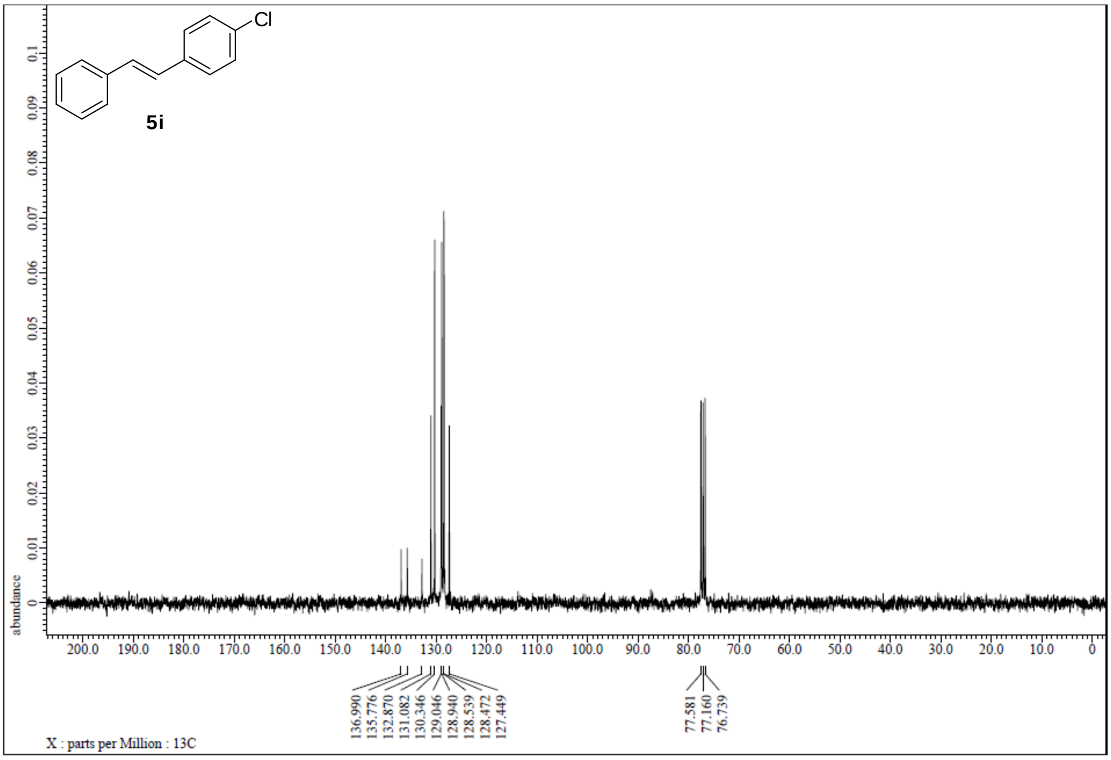

Figure S124. ^{13}C NMR spectrum of 5i, related to Figure 4.

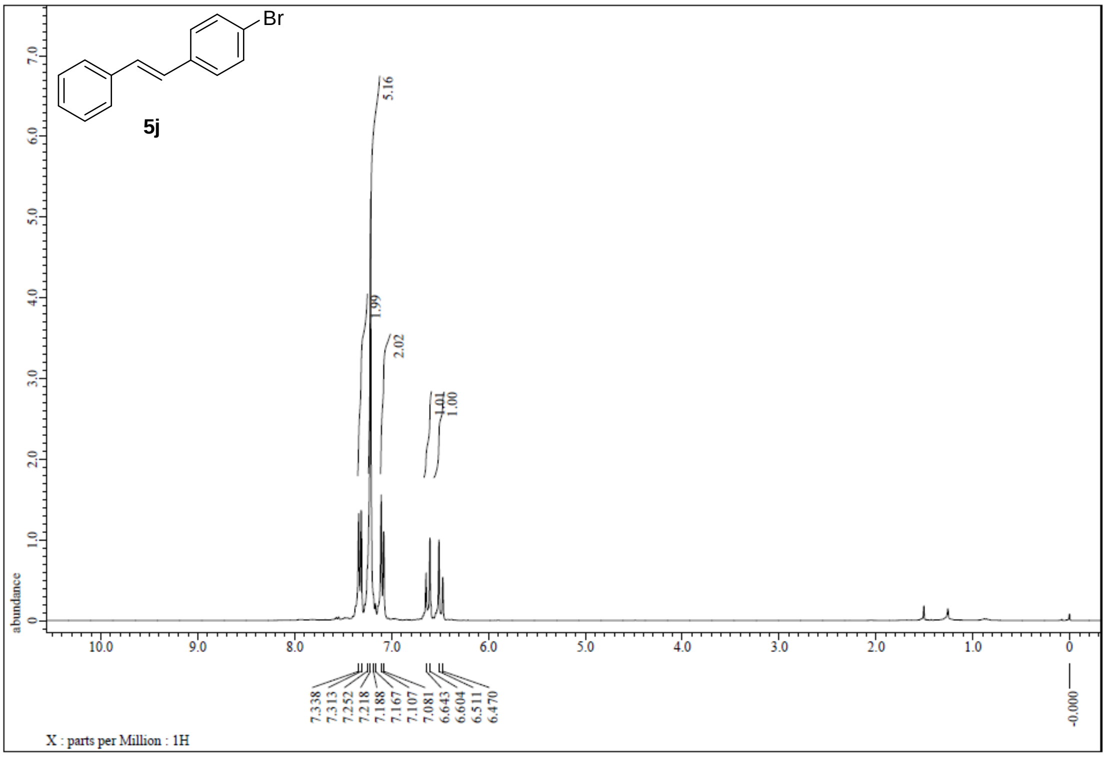

Figure S125. ^{1}H NMR spectrum of 5j, related to Figure 4.

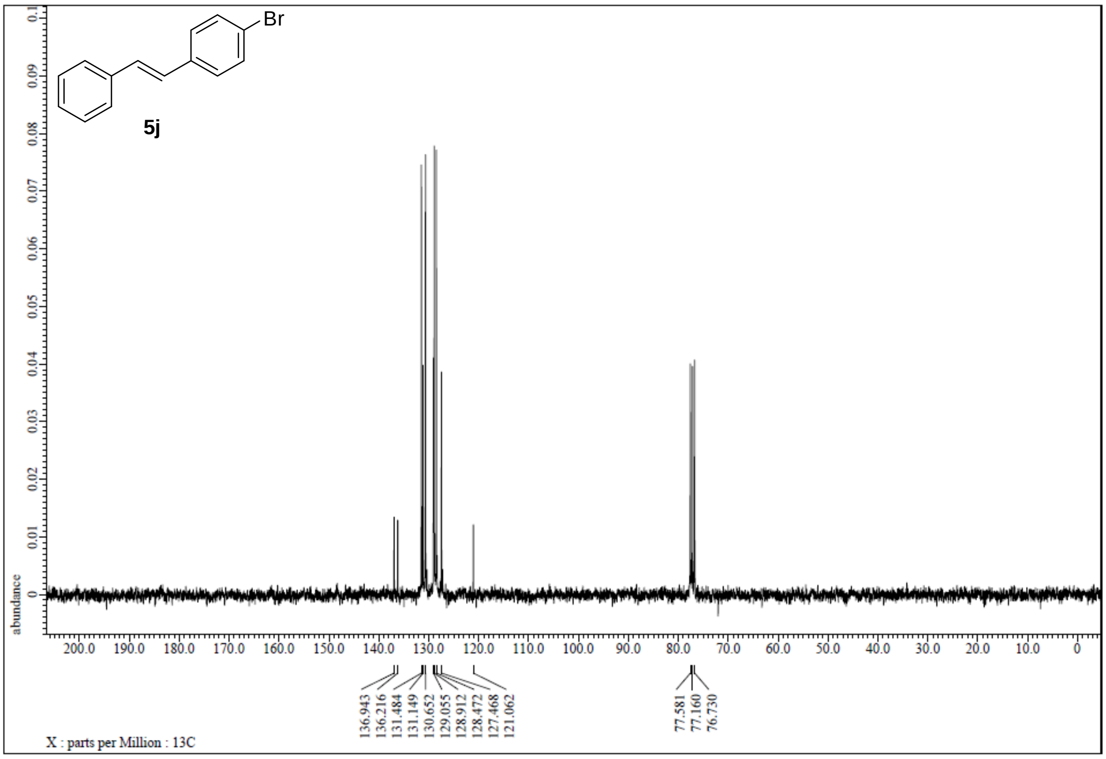

Figure S126. ^{13}C NMR spectrum of 5j, related to Figure 4.

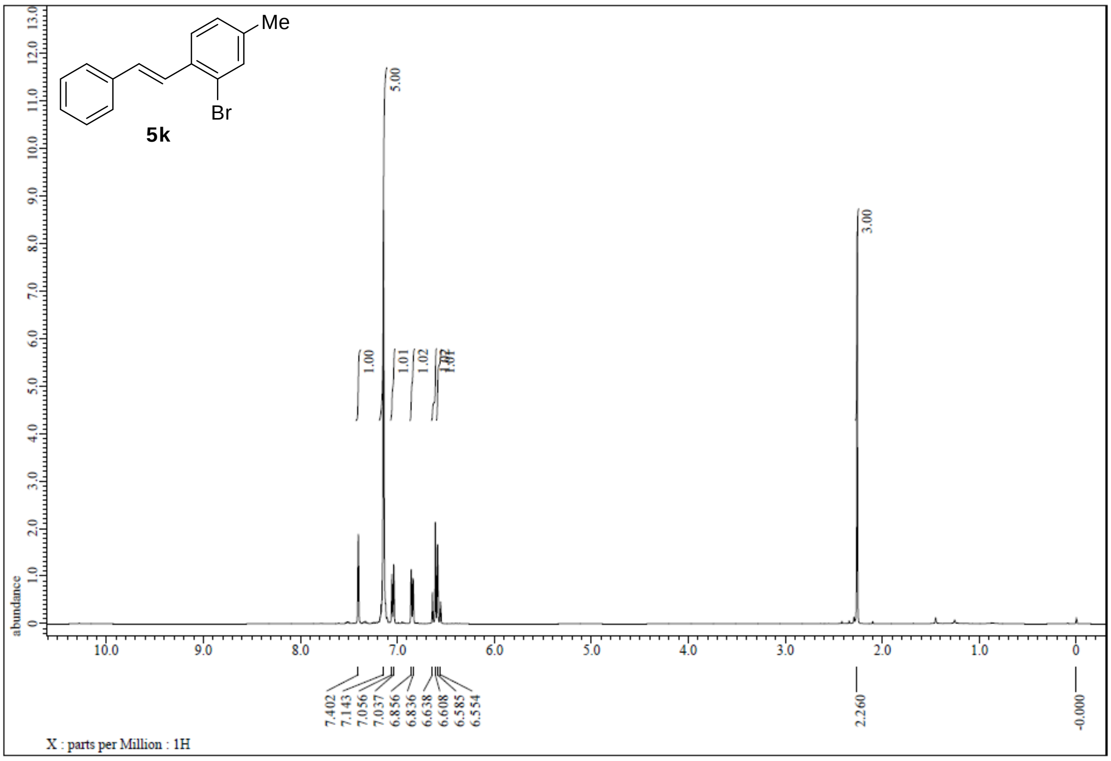

Figure S127. ^{1}**H NMR spectrum of 5k, related to Figure 4**.

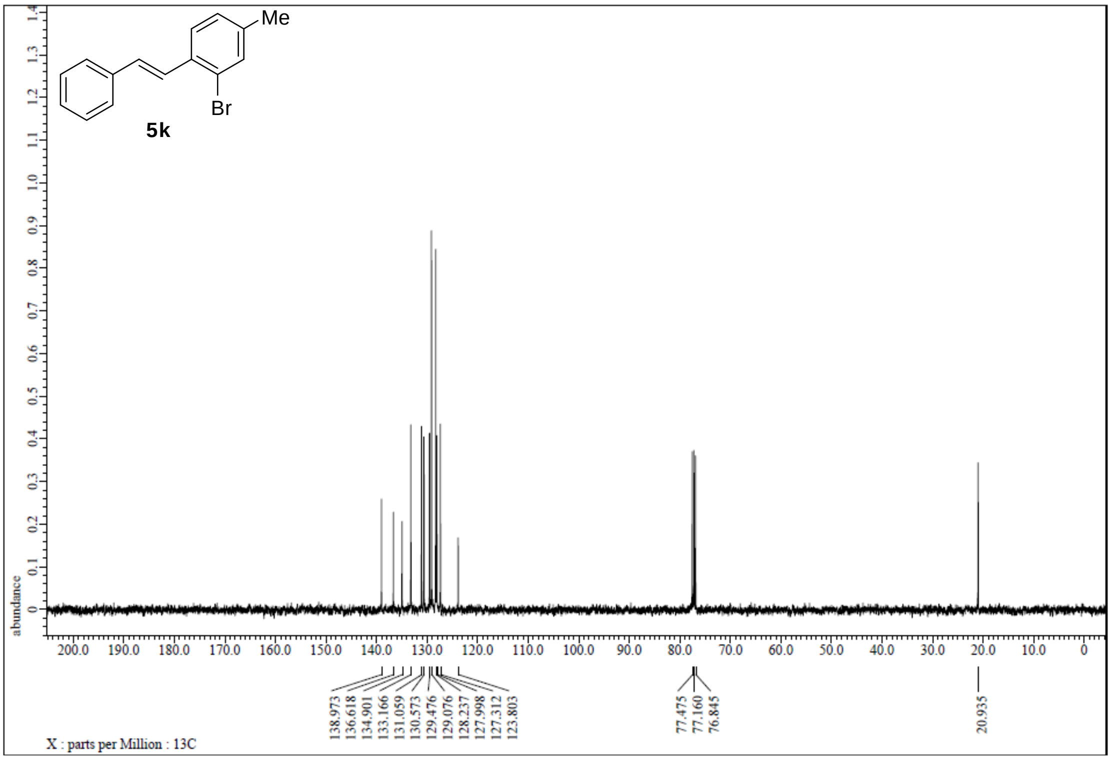

Figure S128. ^{13}**C NMR spectrum of 5k, related to Figure 4**.

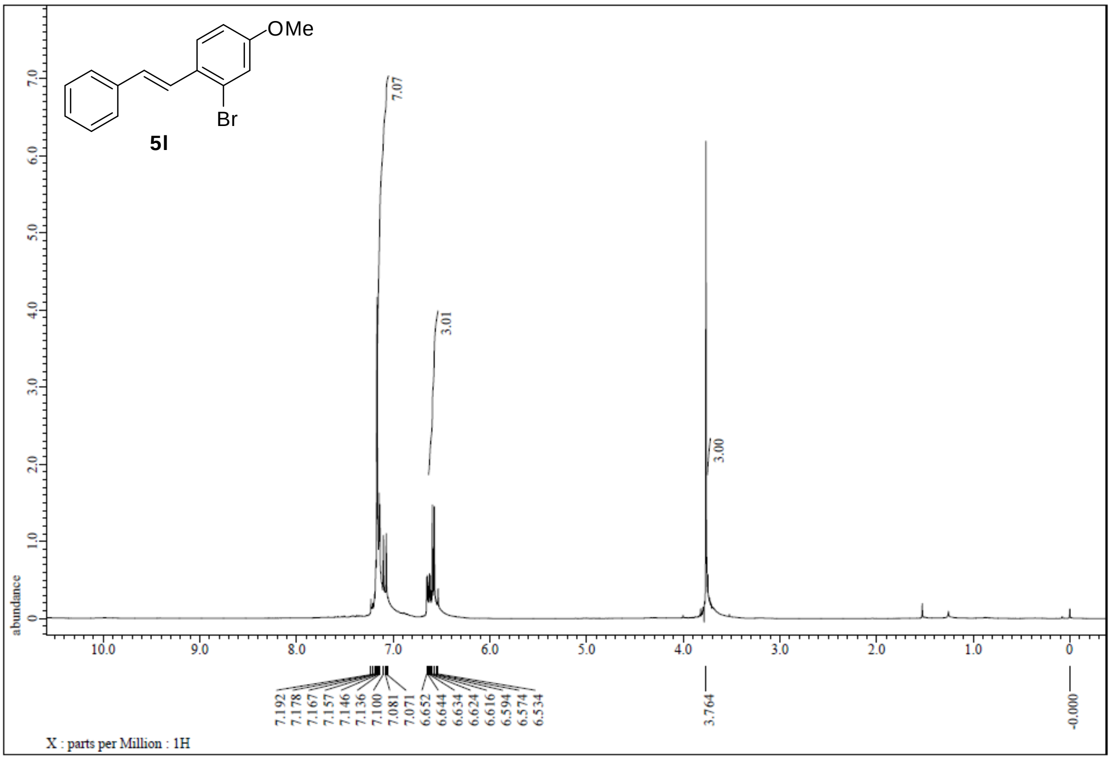

Figure S129. **^{1}H NMR spectrum of 5l, related to Figure 4**.

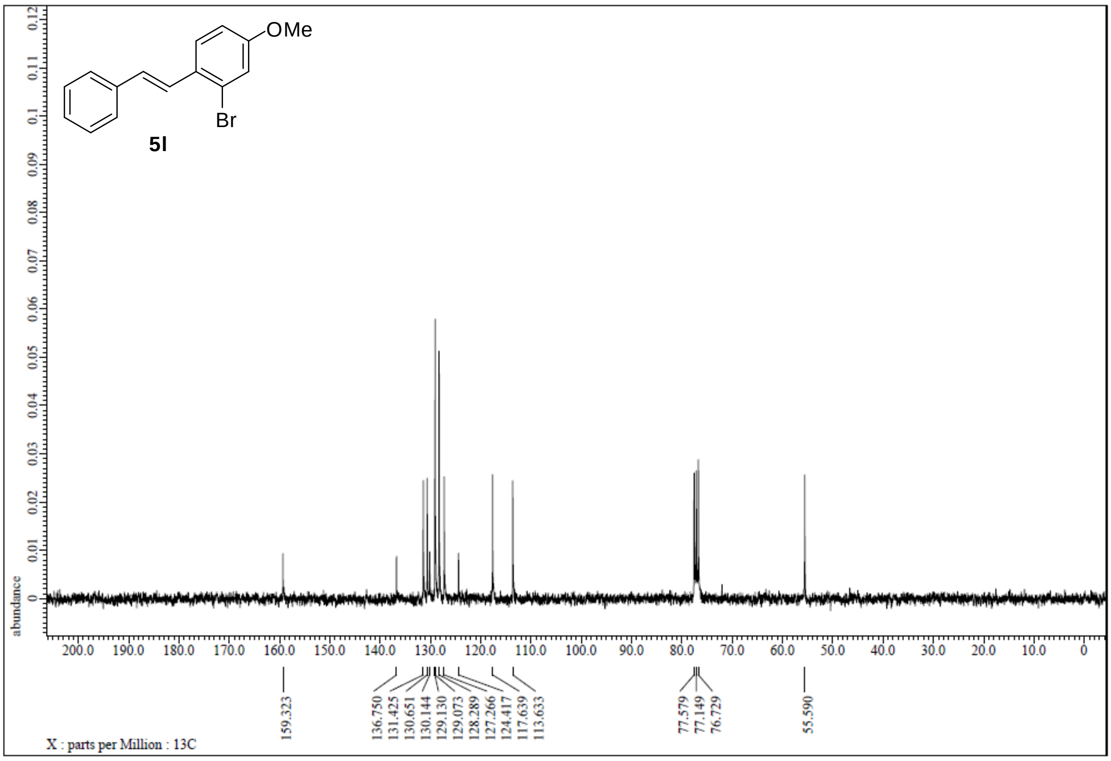

Figure S130. **^{13}C NMR spectrum of 5l, related to Figure 4**.

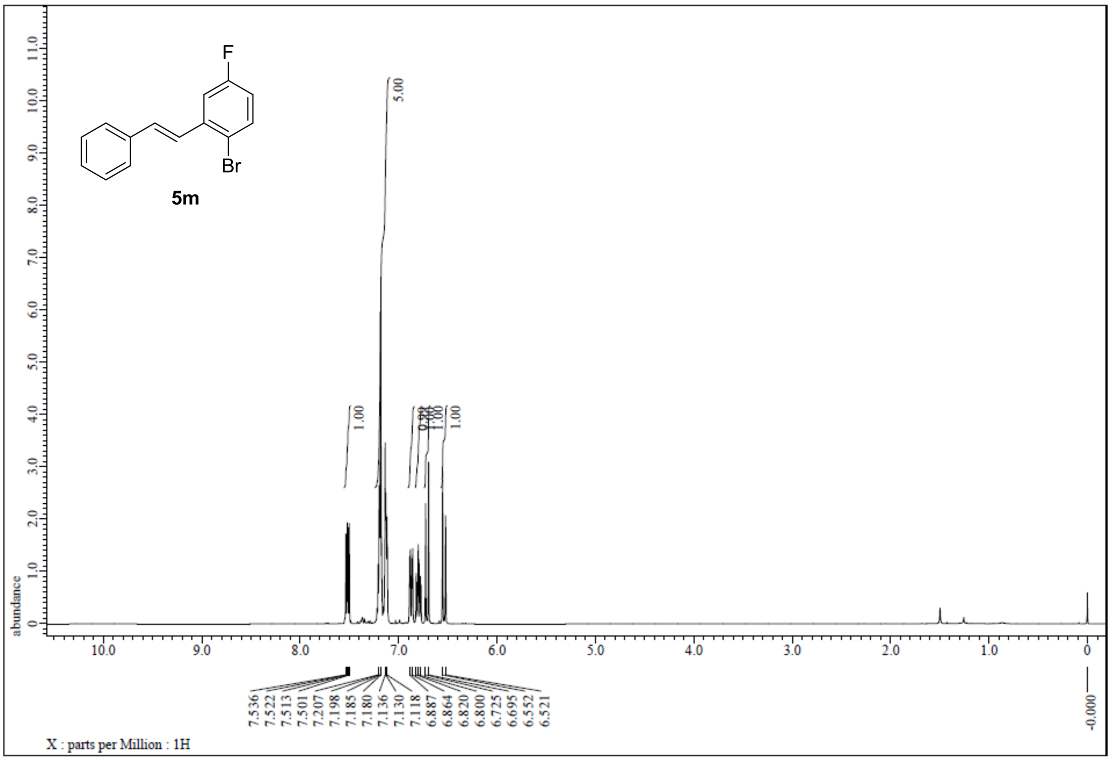

Figure S131. ^{1}H NMR spectrum of 5m, related to Figure 4.

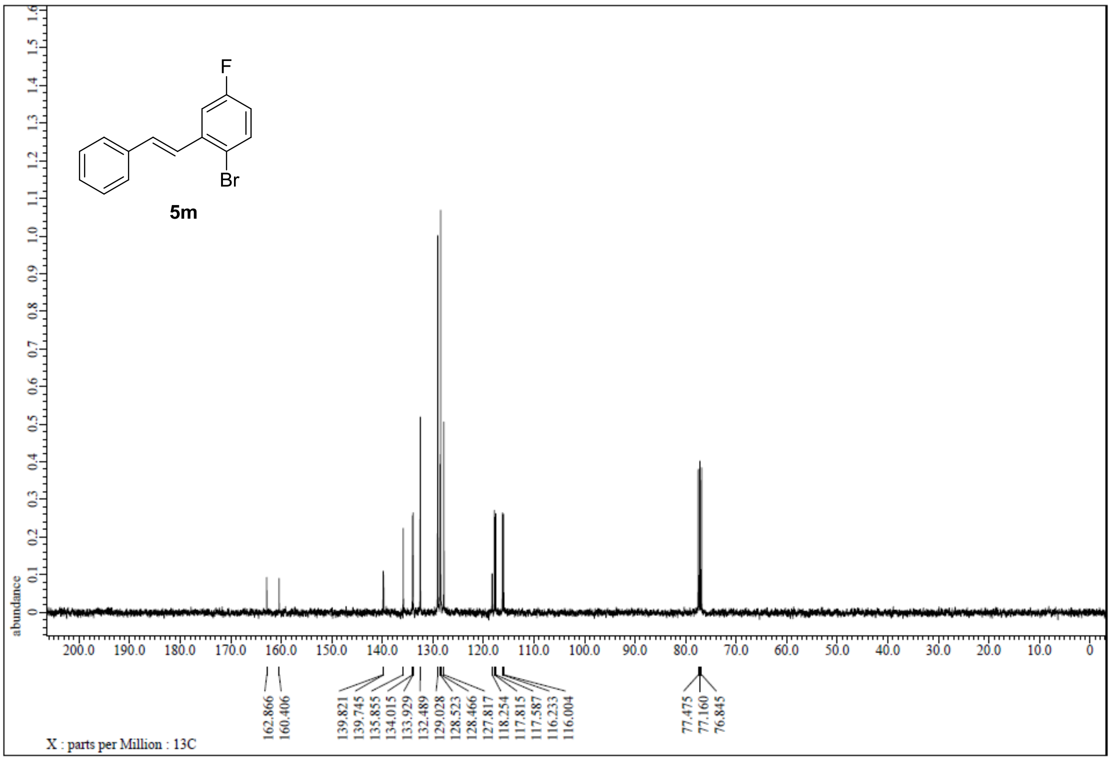

Figure S132. ^{13}C NMR spectrum of 5m, related to Figure 4.

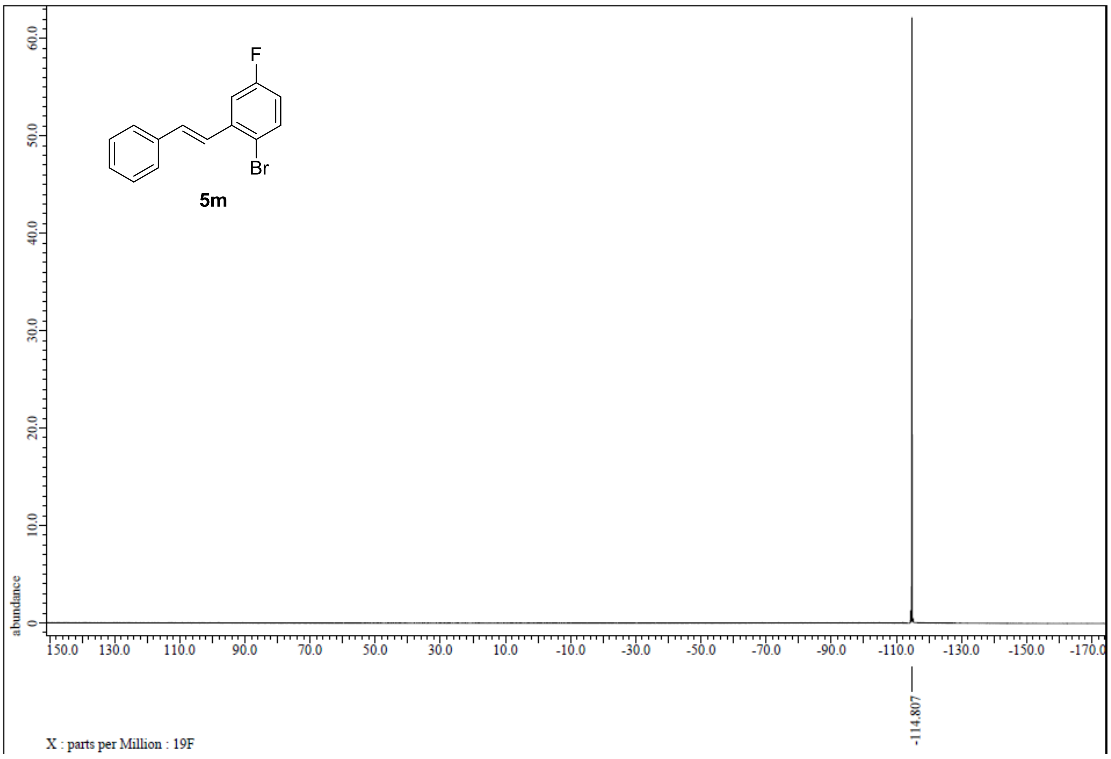

Figure S133. ^{19}C NMR spectrum of 5m, related to Figure 4.

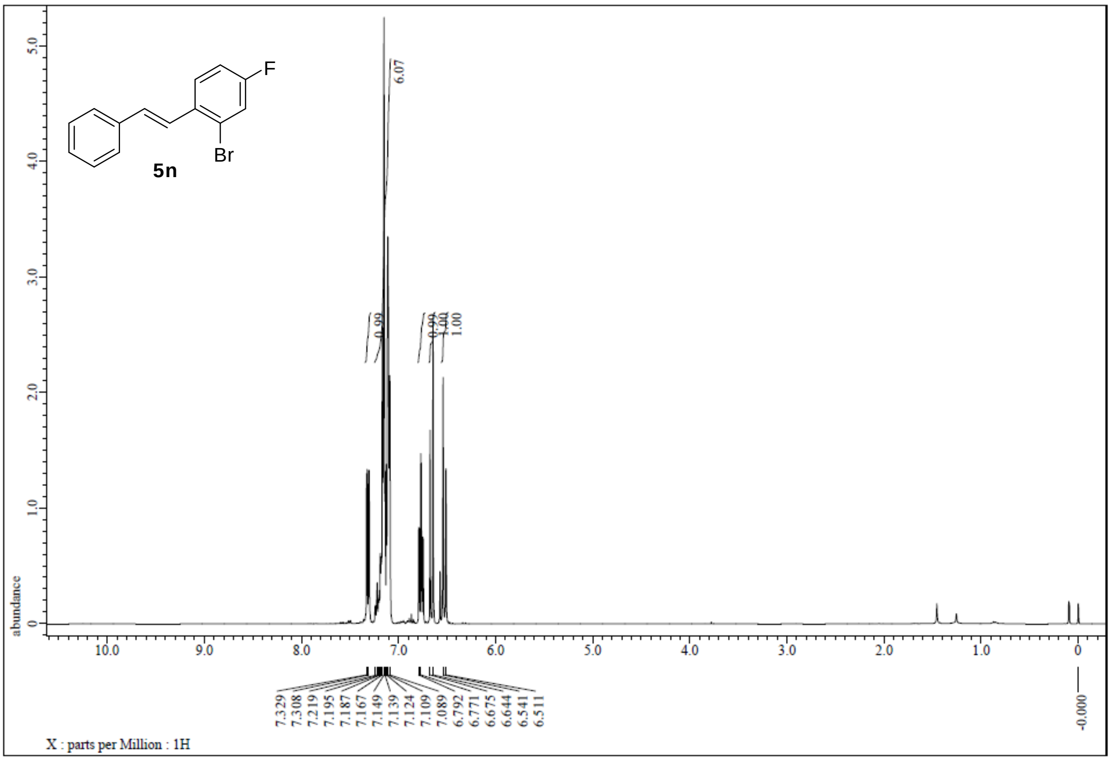

Figure S134. ¹H NMR spectrum of 5n, related to Figure 4.

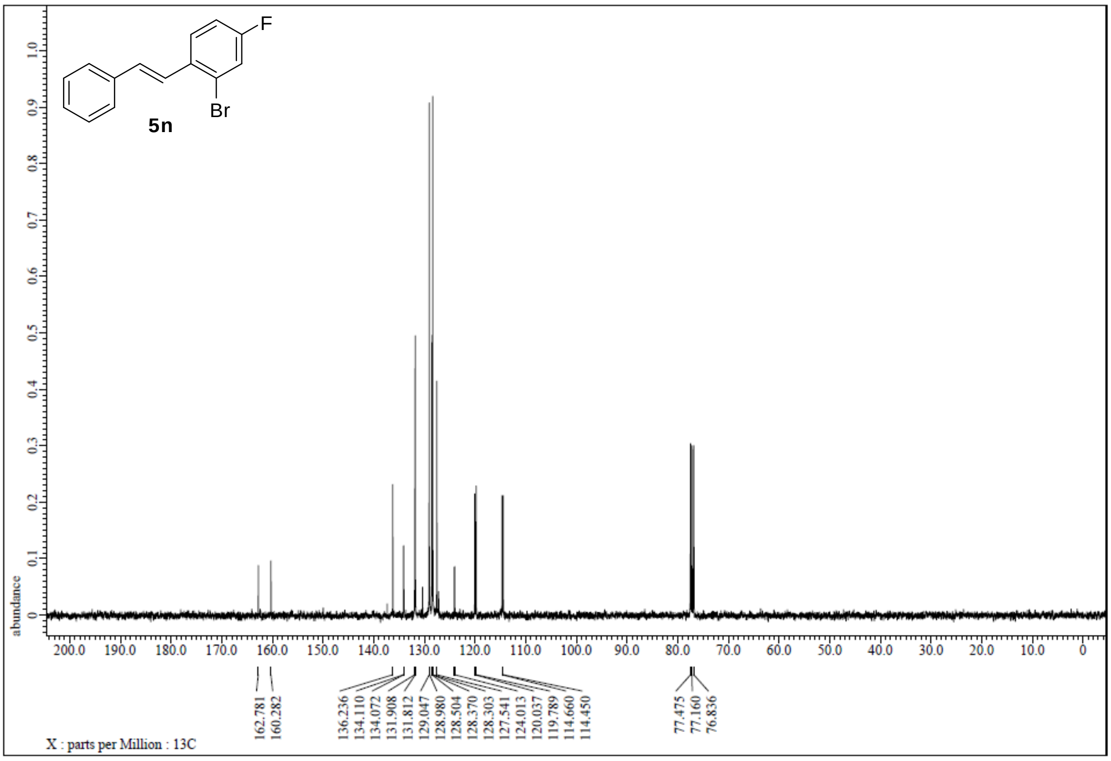

Figure S135. ¹³C NMR spectrum of 5n, related to Figure 4.

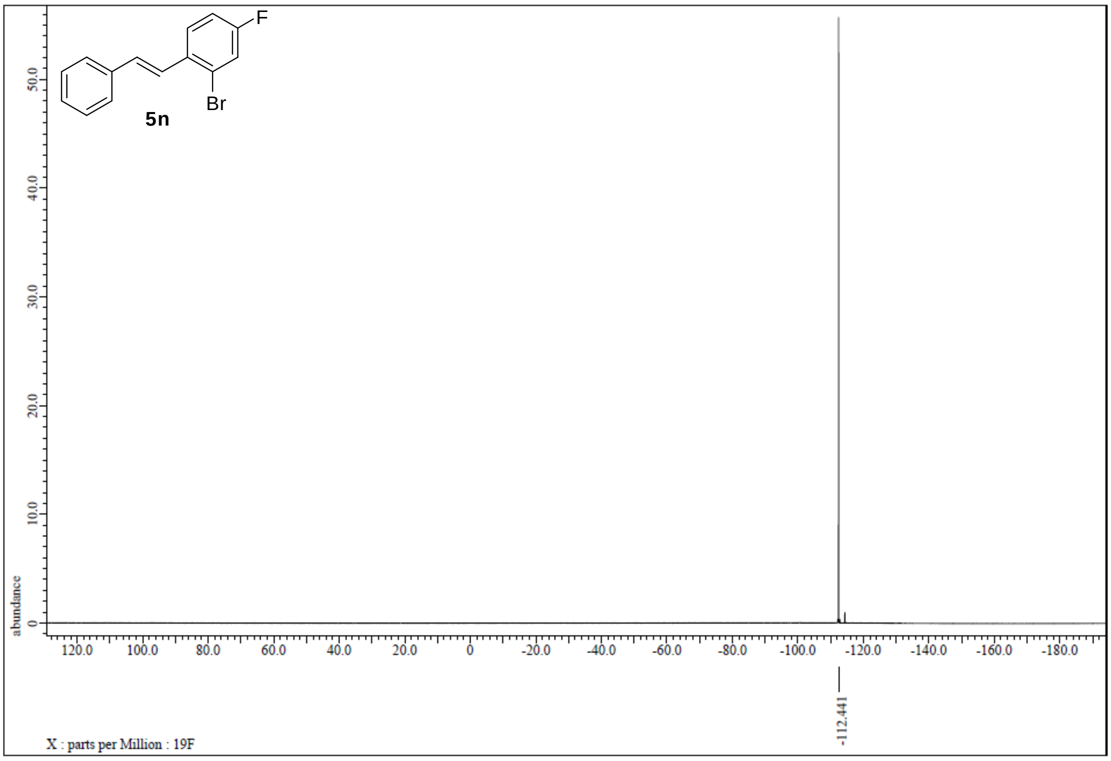

Figure S136. ^{19}C NMR spectrum of 5n, related to Figure 4.

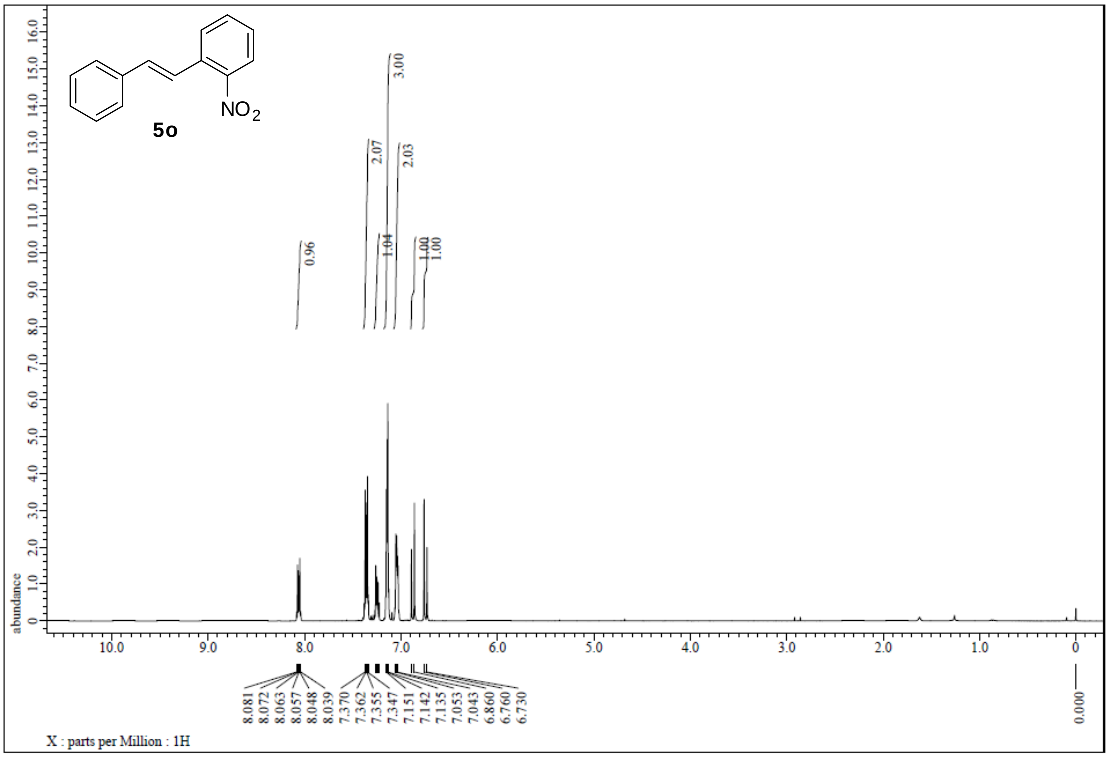

Figure S137. **^{1}H NMR spectrum of 5o, related to Figure 4**.

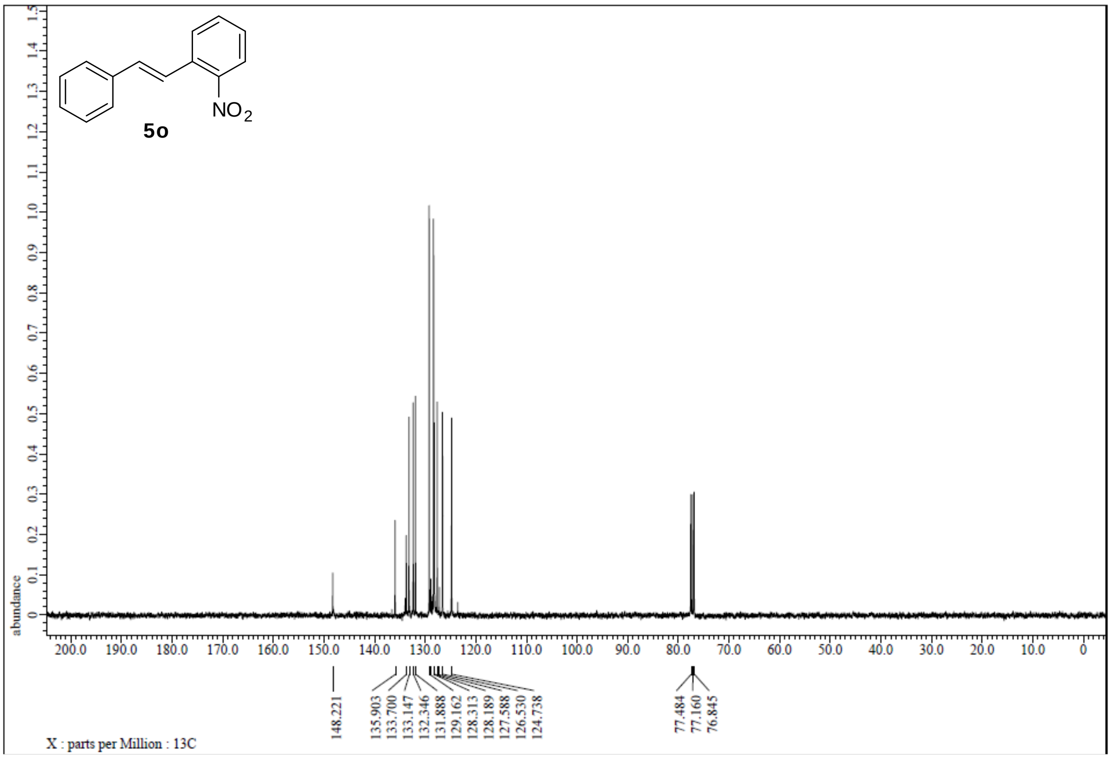

Figure S138. **^{13}C NMR spectrum of 5o, related to Figure 4**.

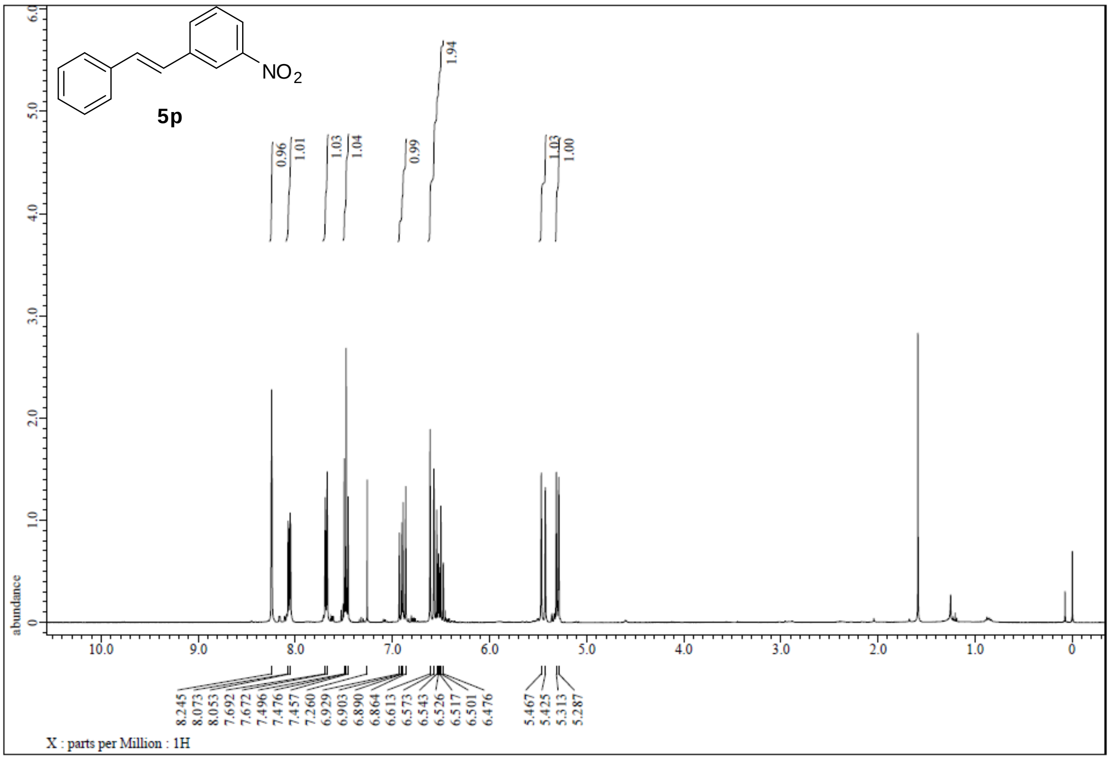

Figure S139. ¹H NMR spectrum of 5p, related to Figure 4.

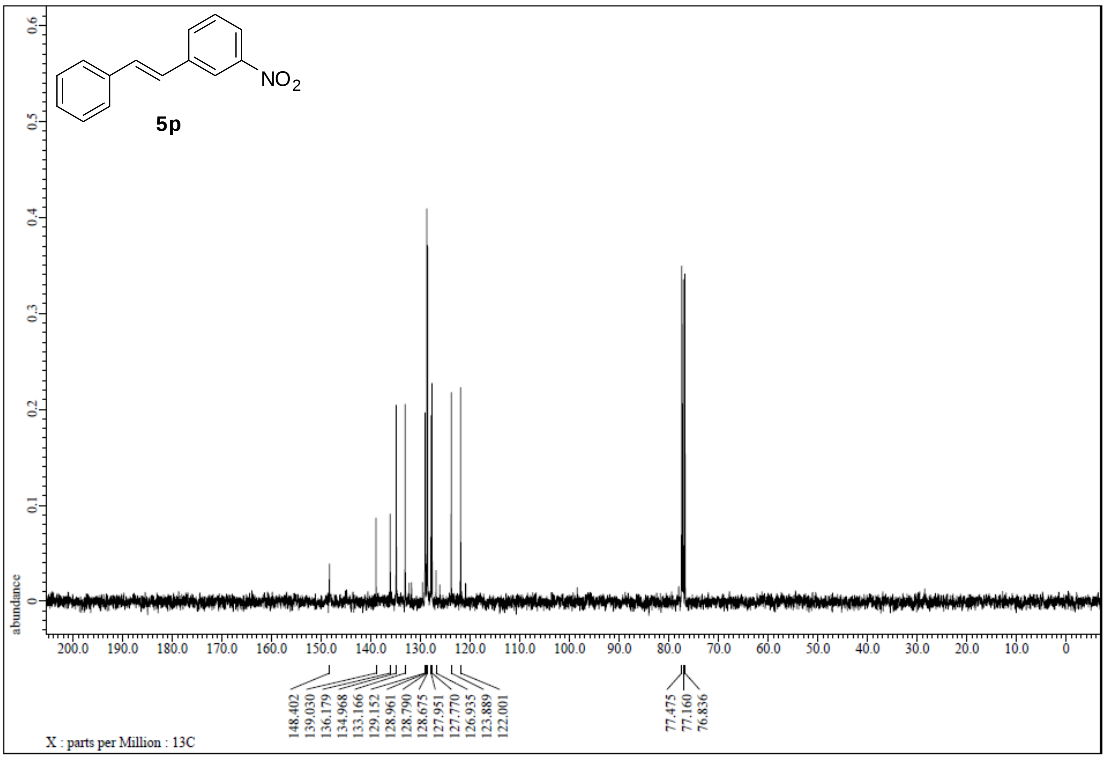

Figure S140. ¹³C NMR spectrum of 5p, related to Figure 4.

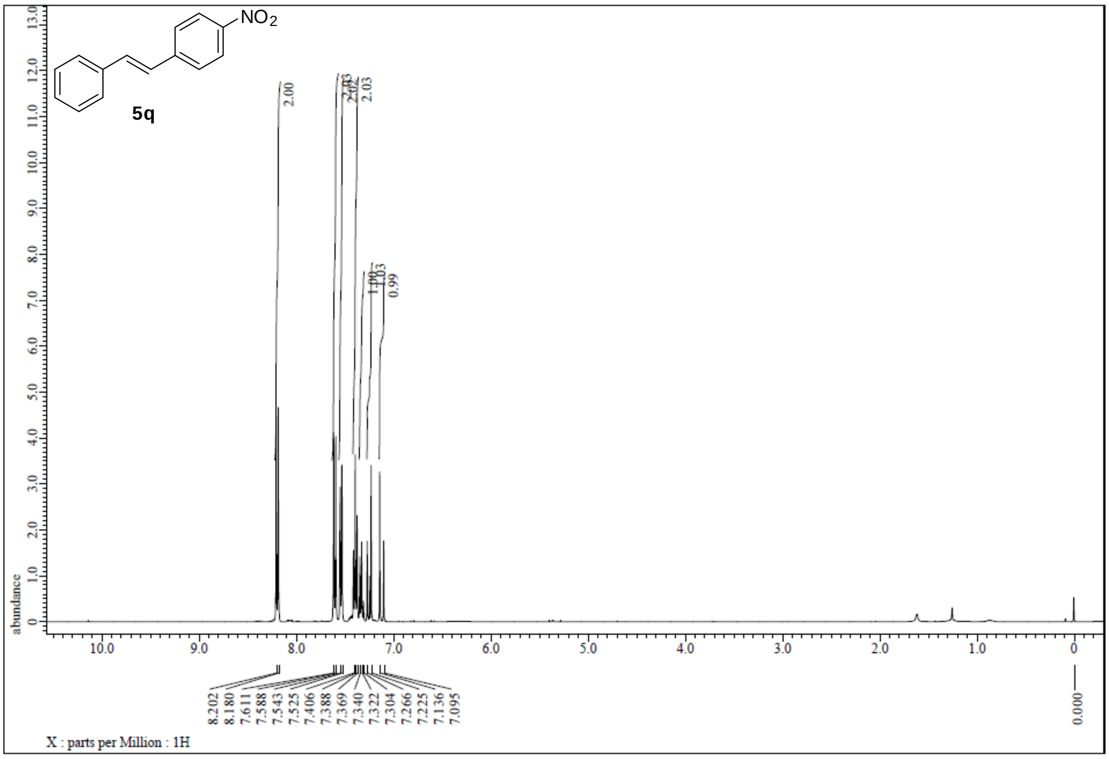

Figure S141. ^{1}H NMR spectrum of 5q, related to Figure 4.

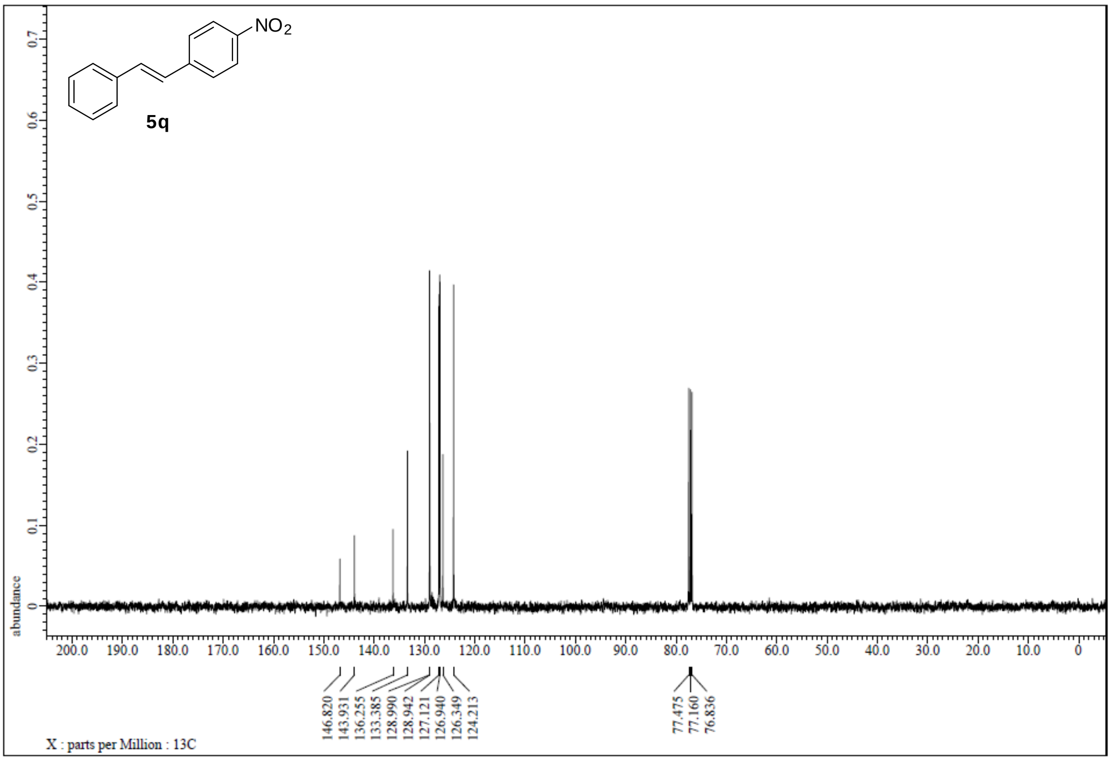

Figure S142. ^{13}C NMR spectrum of 5q, related to Figure 4.

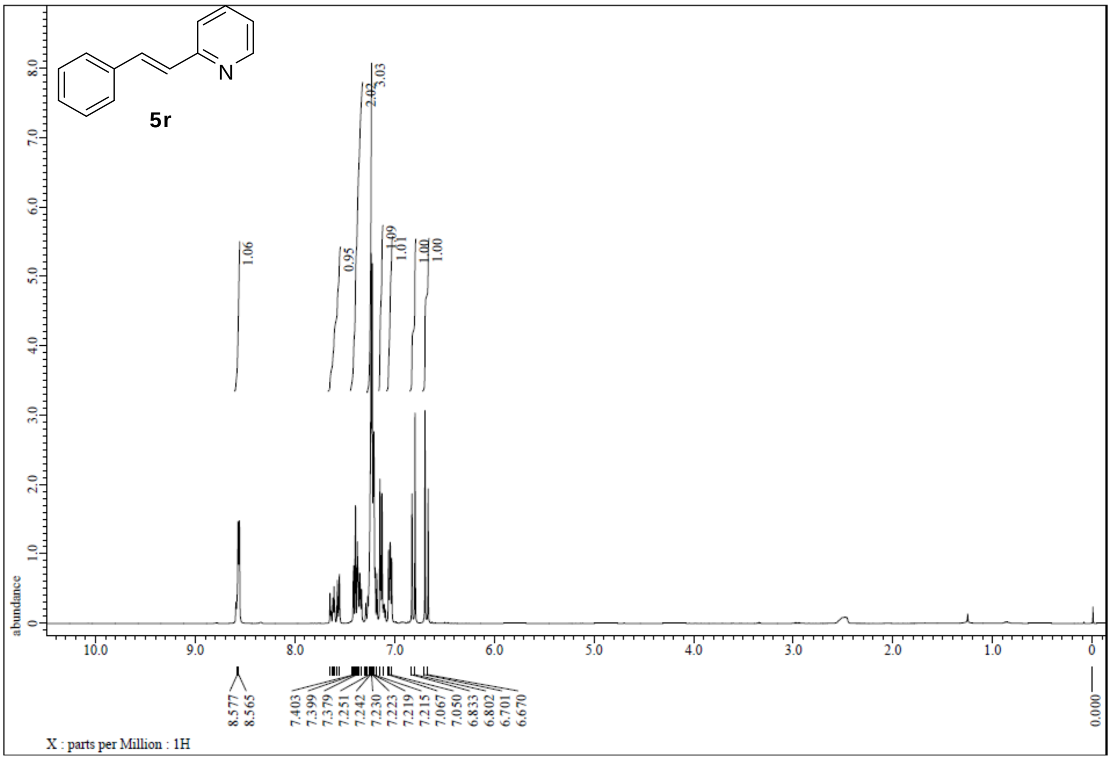

Figure S143. **^{1}H NMR spectrum of 5r, related to Figure 4**.

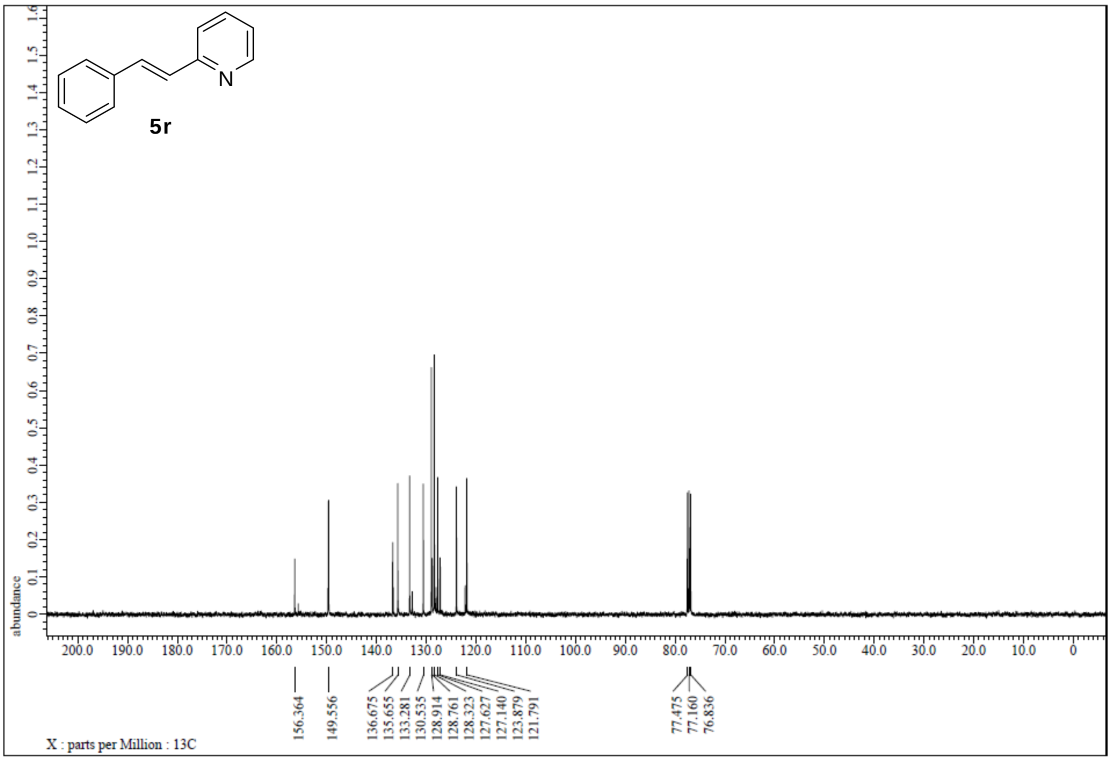

Figure S144. **^{13}C NMR spectrum of 5r, related to Figure 4**.

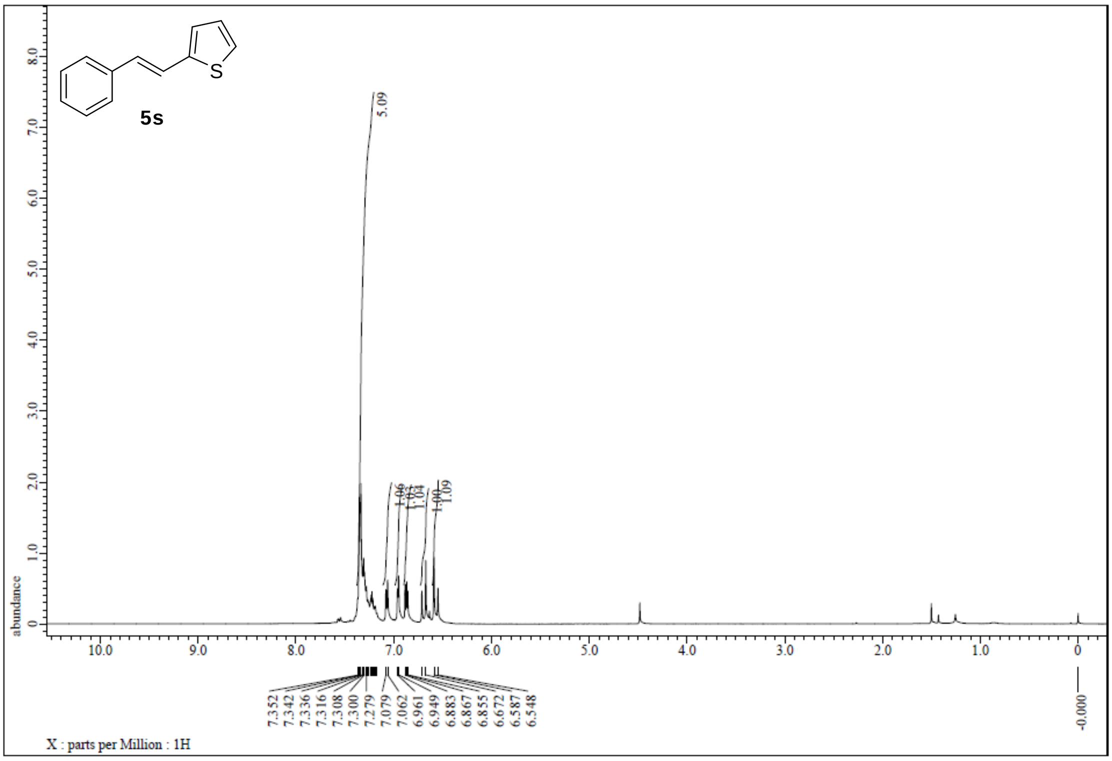

Figure S145. ^{1}H NMR spectrum of 5s, related to Figure 4.

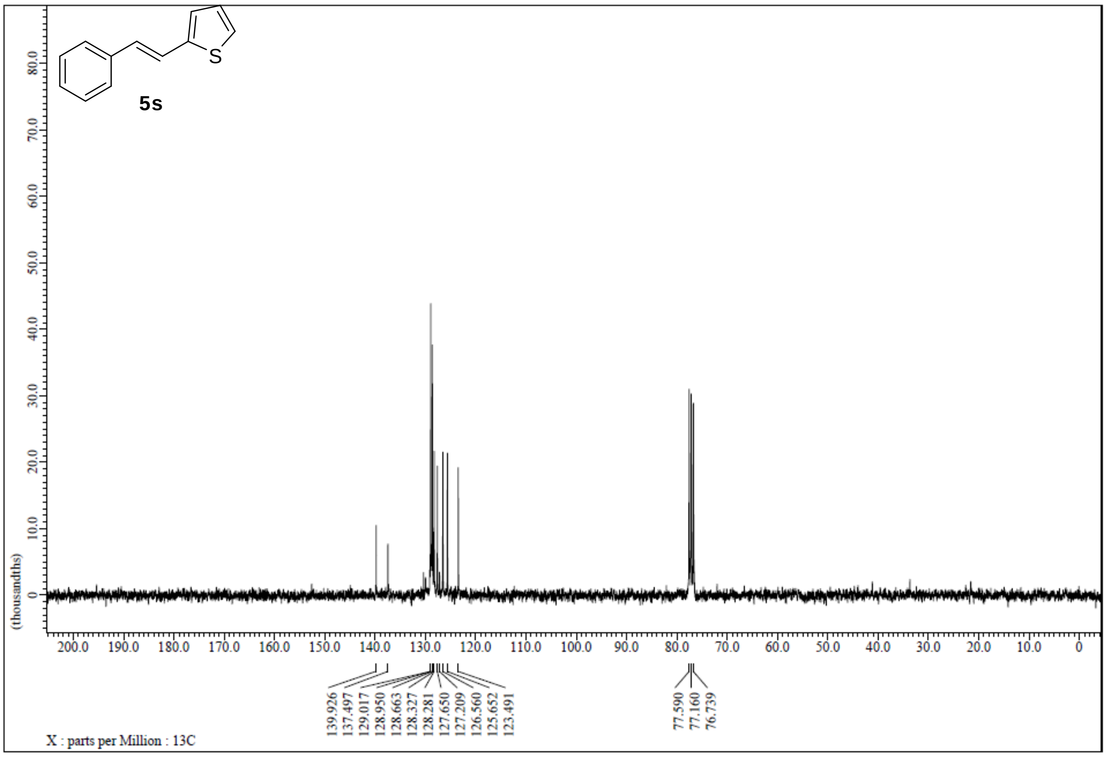

Figure S146. ^{13}C NMR spectrum of 5s, related to Figure 4.

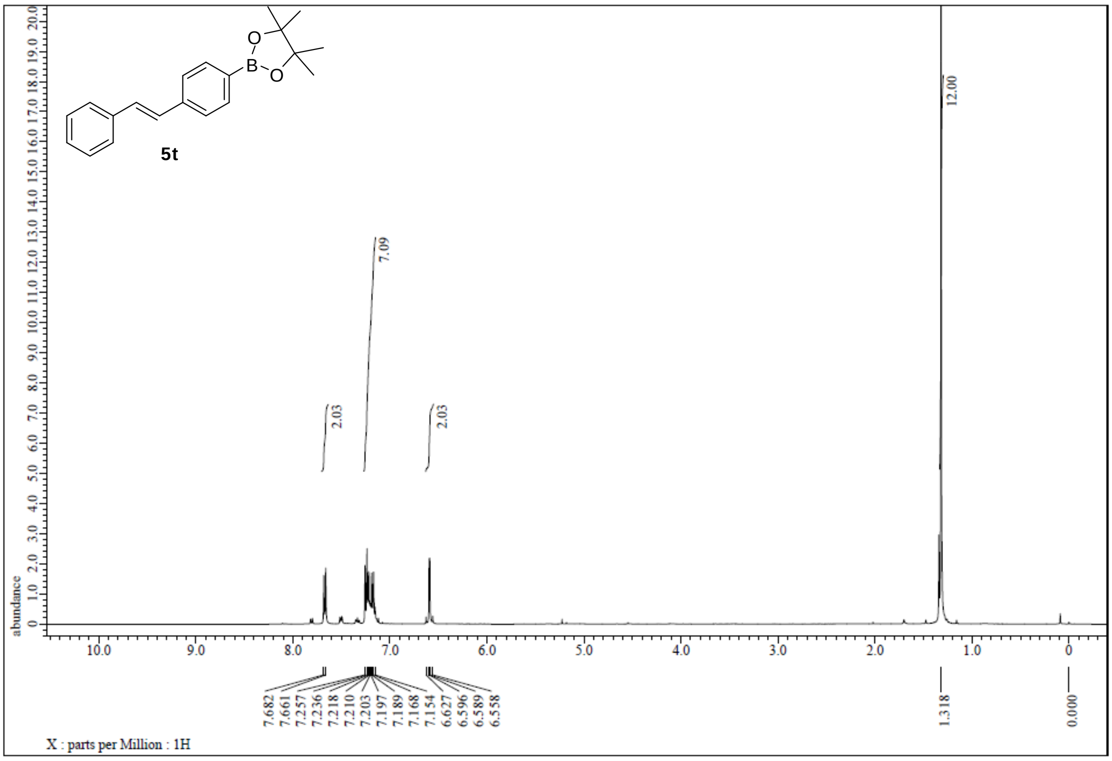

Figure S147. ^{1}H NMR spectrum of 5t, related to Figure 4.

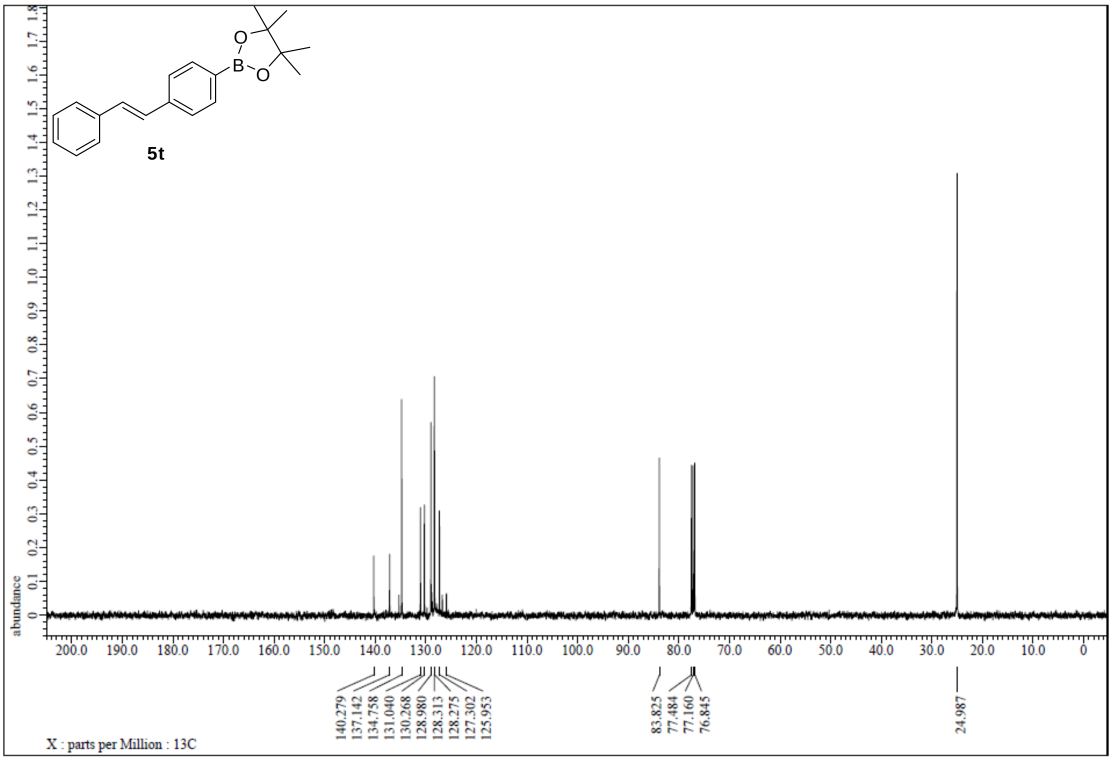

Figure S148. ^{13}C NMR spectrum of 5t, related to Figure 4.

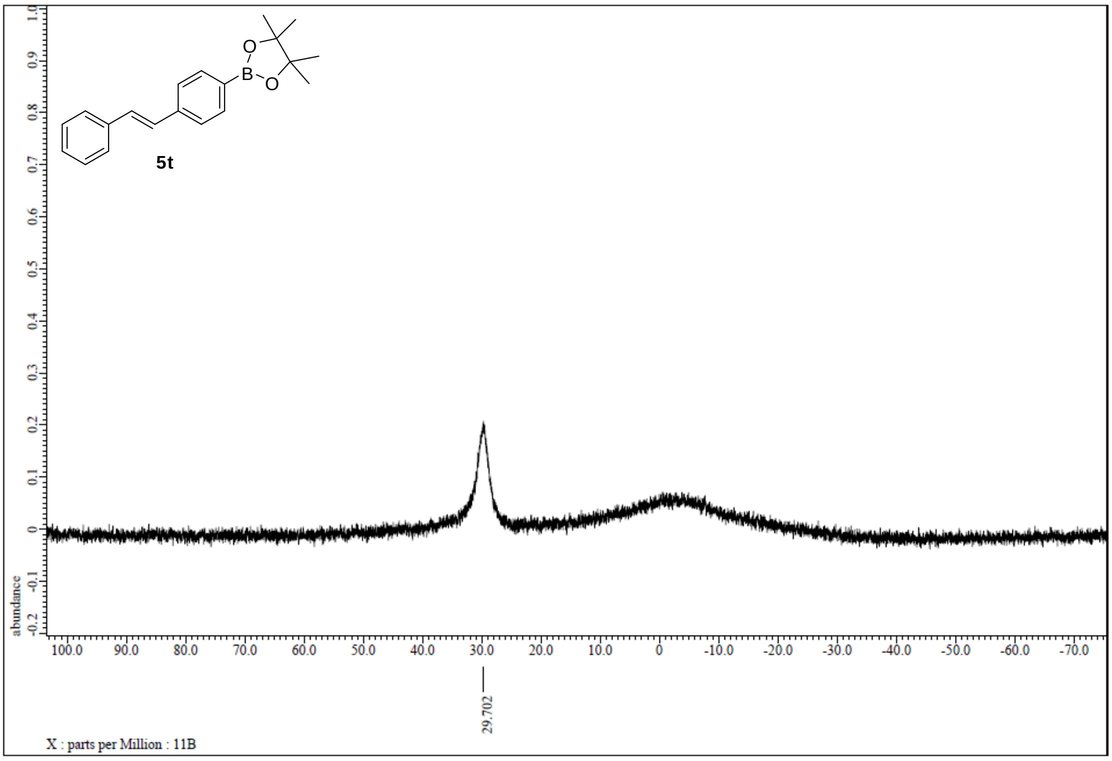

Figure S149. ^{11}B NMR spectrum of 5t, related to Figure 4.

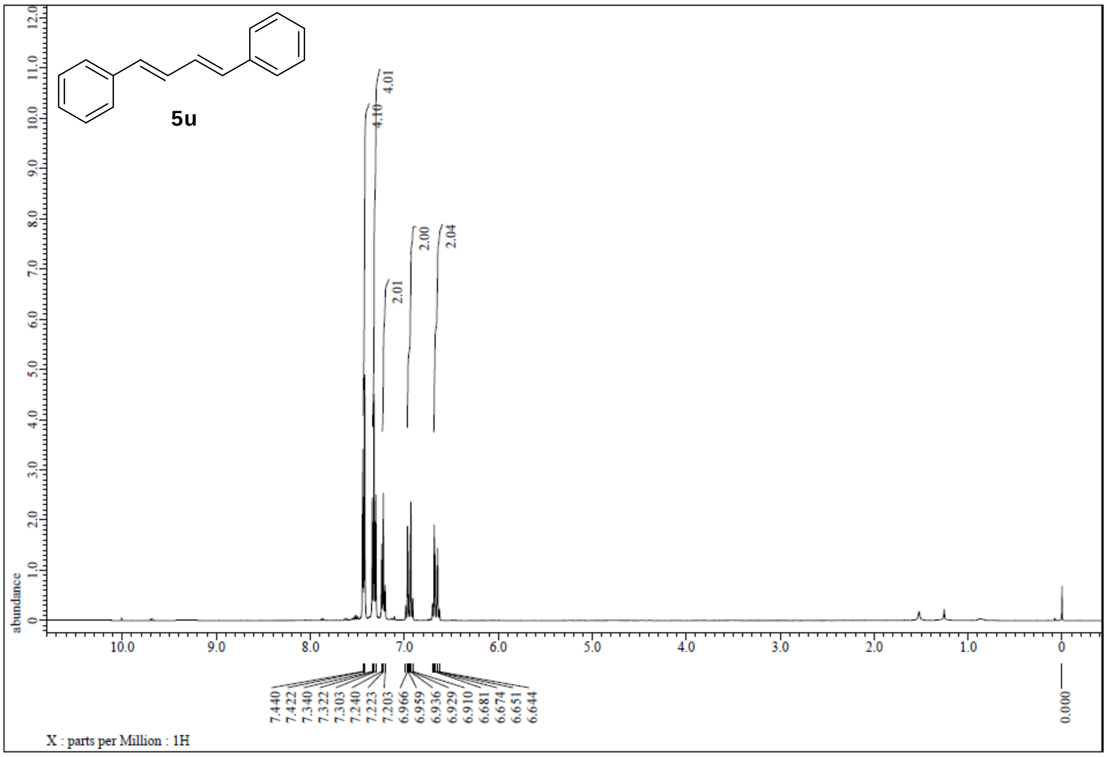

Figure S150. **^{1}H NMR spectrum of 5u, related to Figure 4**.

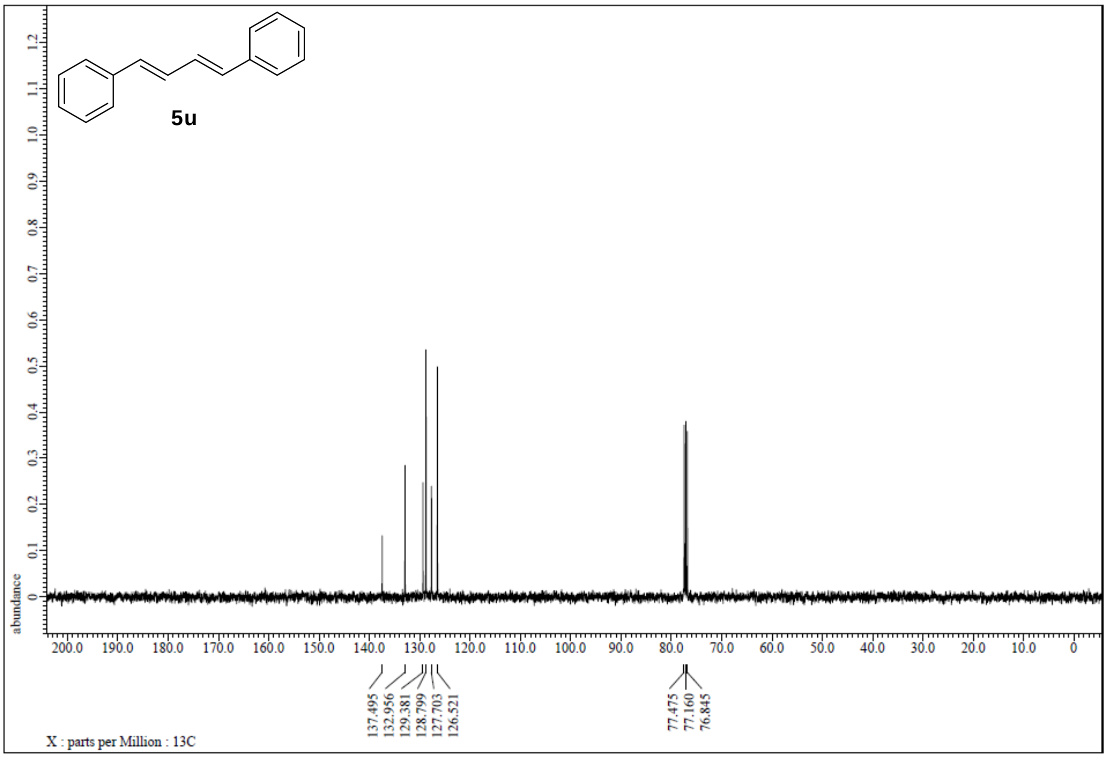

Figure S151. **^{13}C NMR spectrum of 5u, related to Figure 4**.

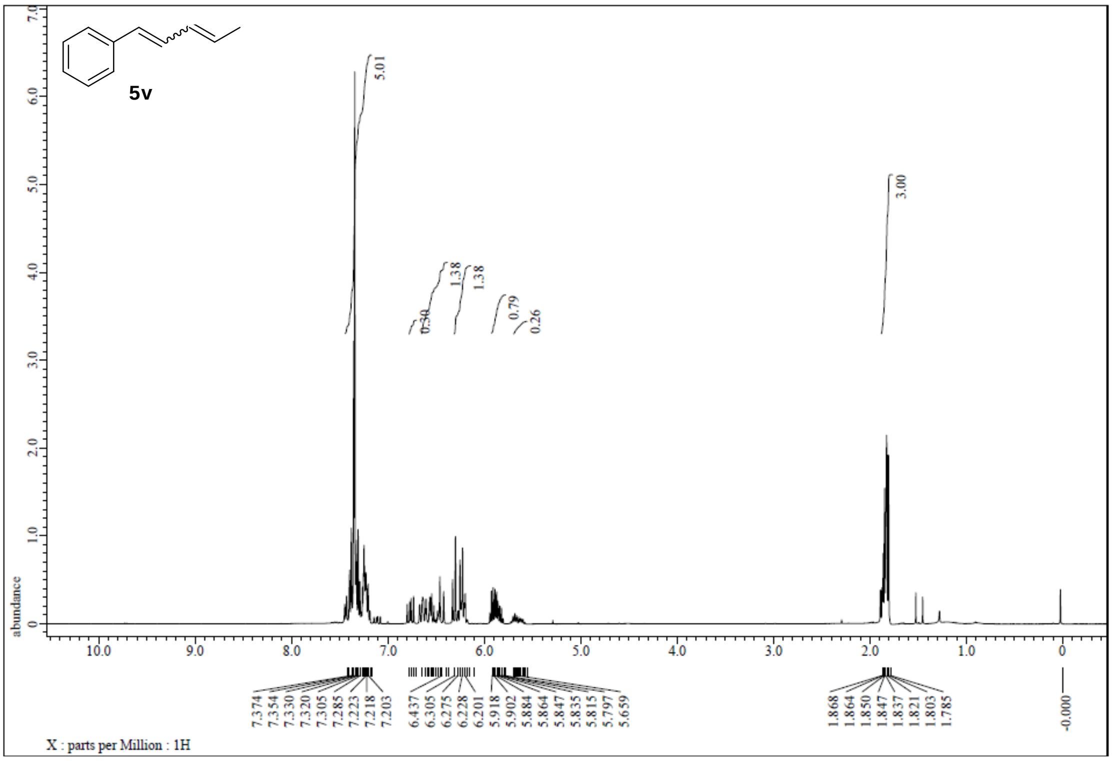

Figure S152. **^{1}H NMR spectrum of 5v, related to Figure 4**.

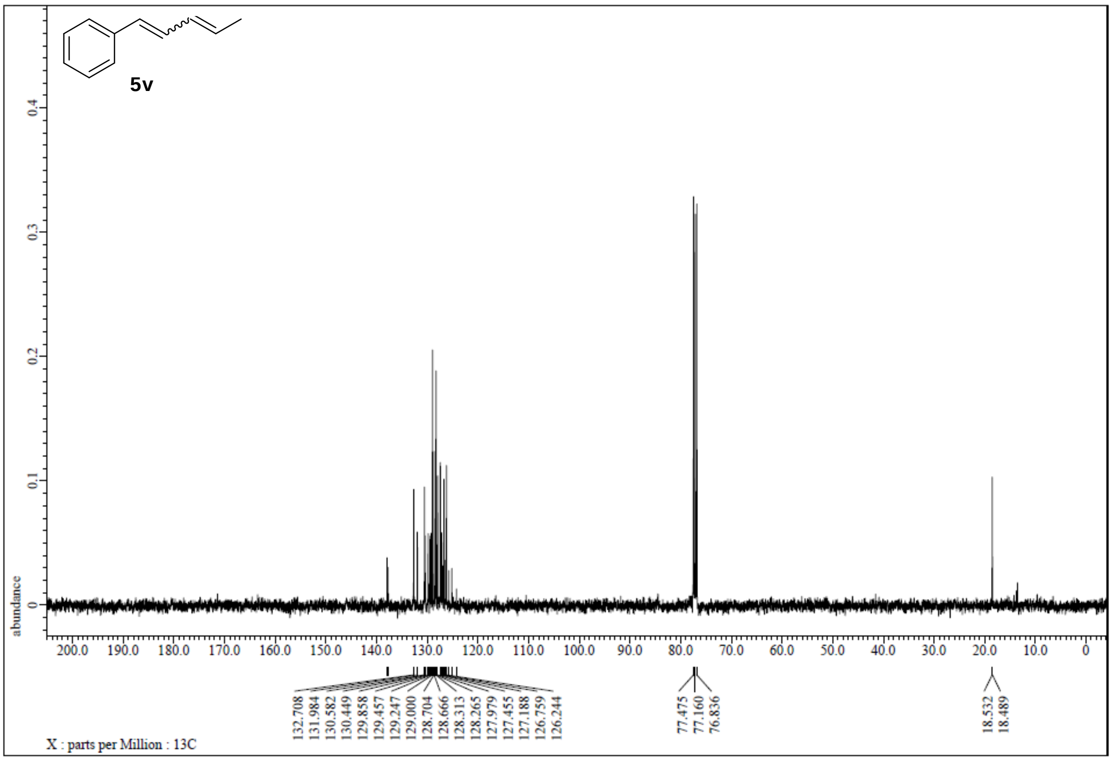

Figure S153. **^{13}C NMR spectrum of 5v, related to Figure 4**.

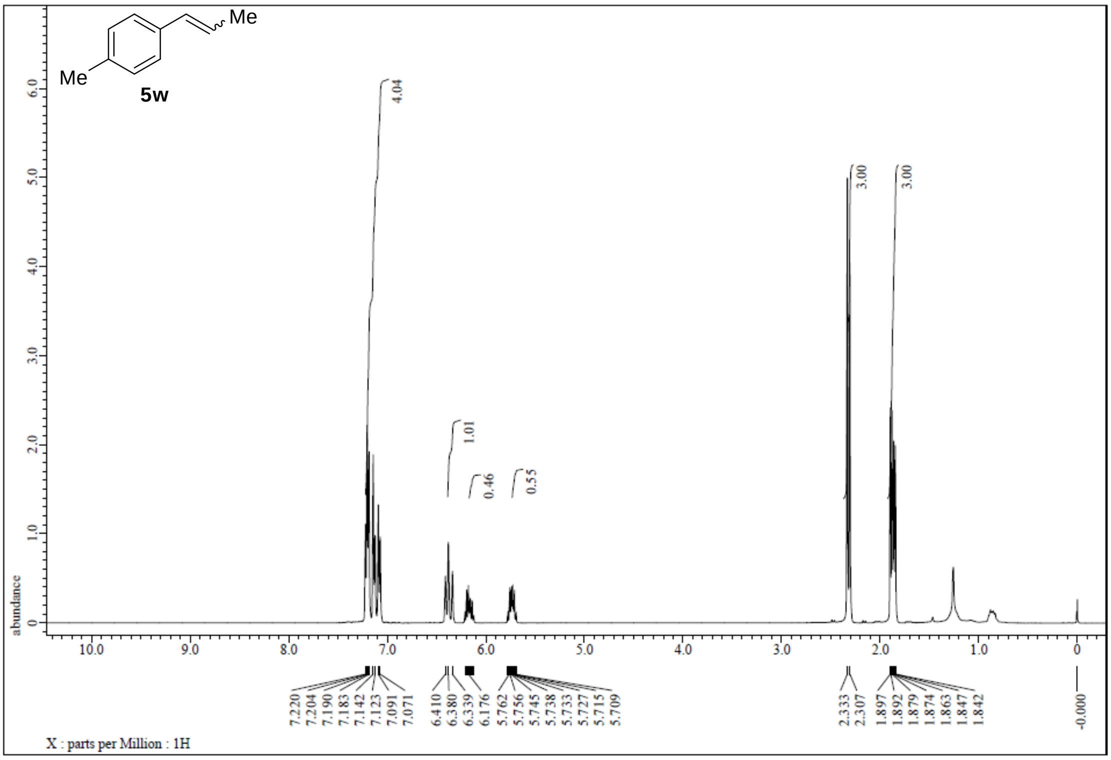

Figure S154. **^{1}H NMR spectrum of 5w, related to Figure 4**.

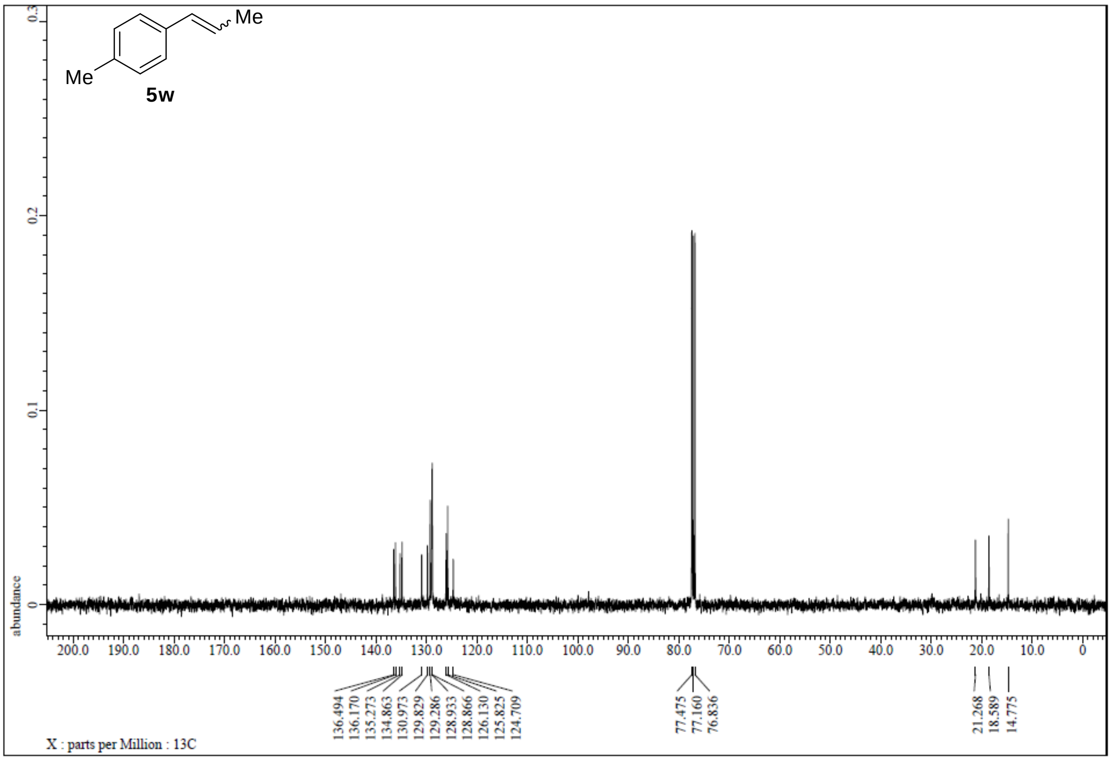

Figure S155. **^{13}C NMR spectrum of 5w, related to Figure 4**.

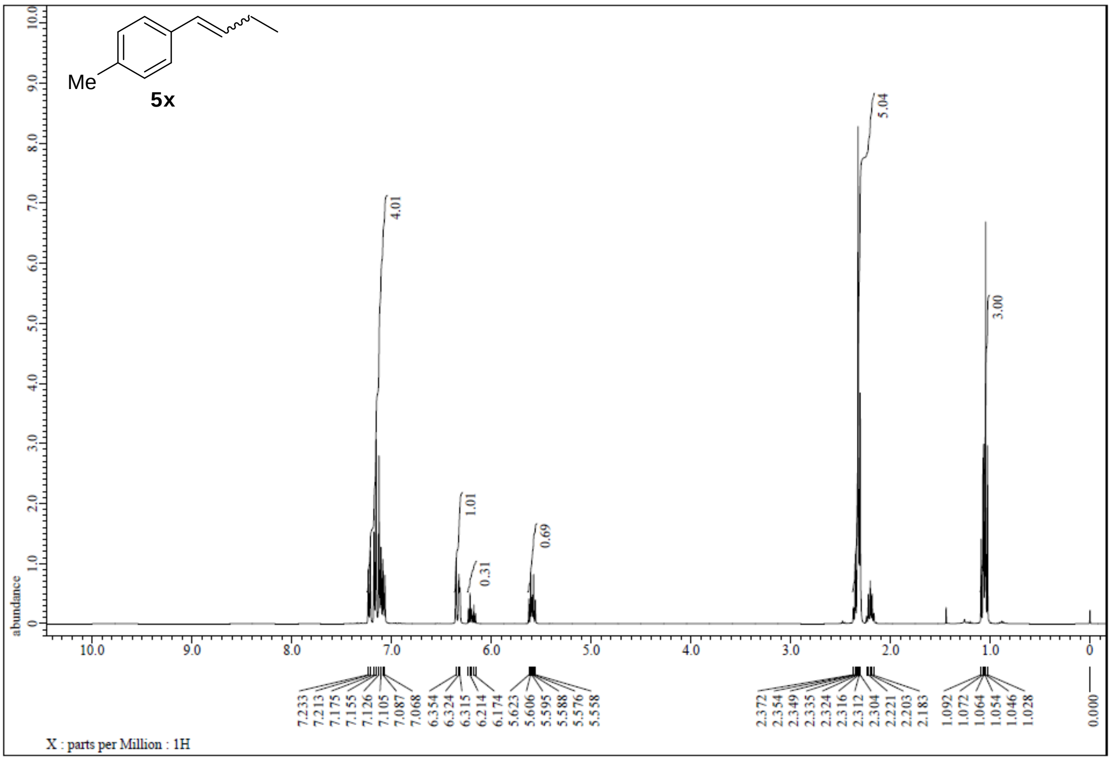

Figure S156. **^{1}H NMR spectrum of 5x, related to Figure 4**.

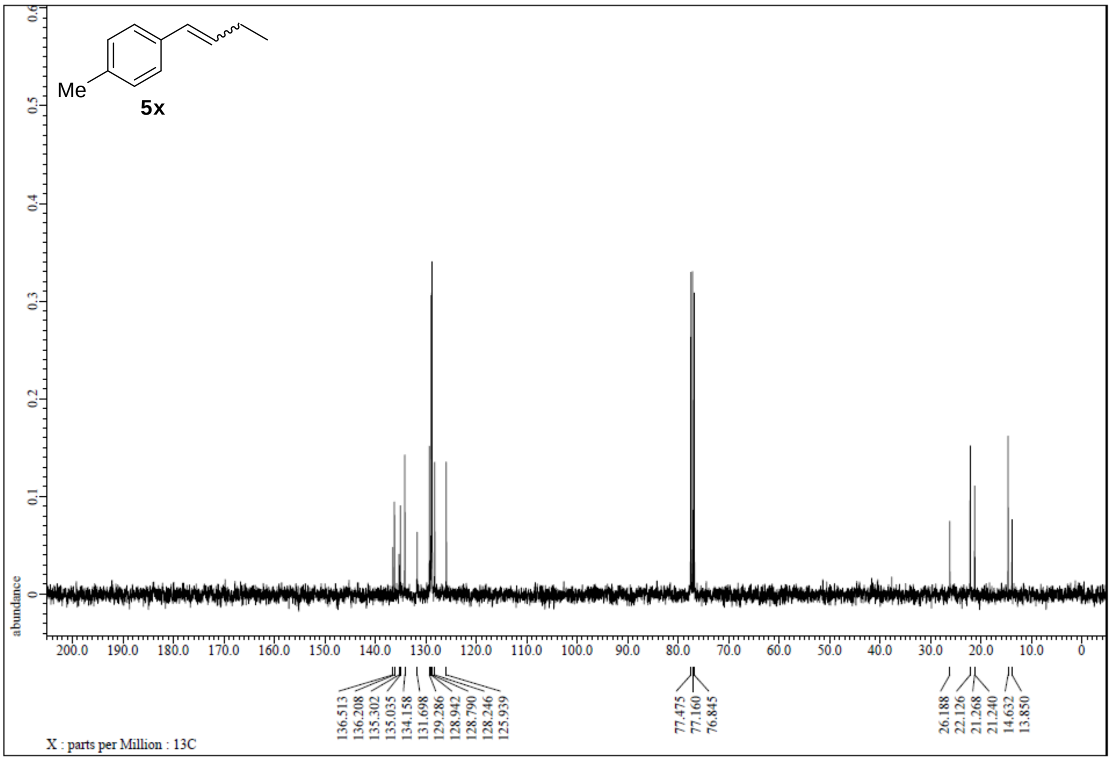

Figure S157. **^{13}C NMR spectrum of 5x, related to Figure 4**.

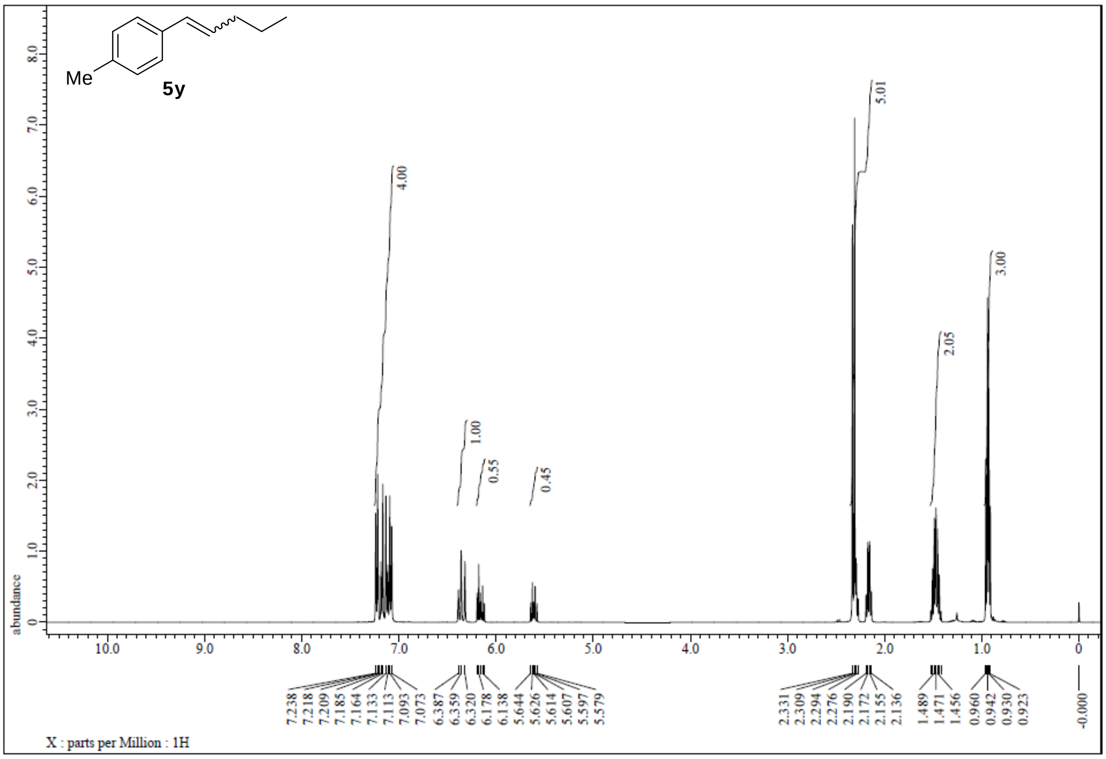

Figure S158. ^{1}H NMR spectrum of 5y, related to Figure 4.

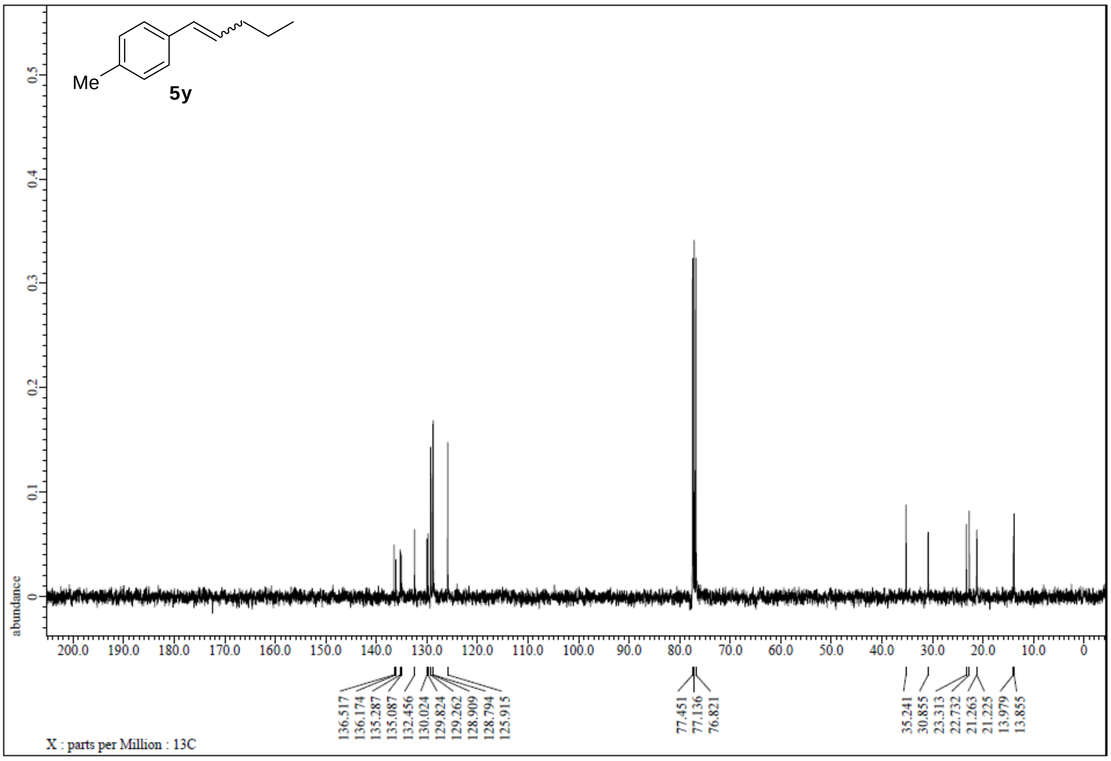

Figure S159. ^{13}C NMR spectrum of 5y, related to Figure 4.

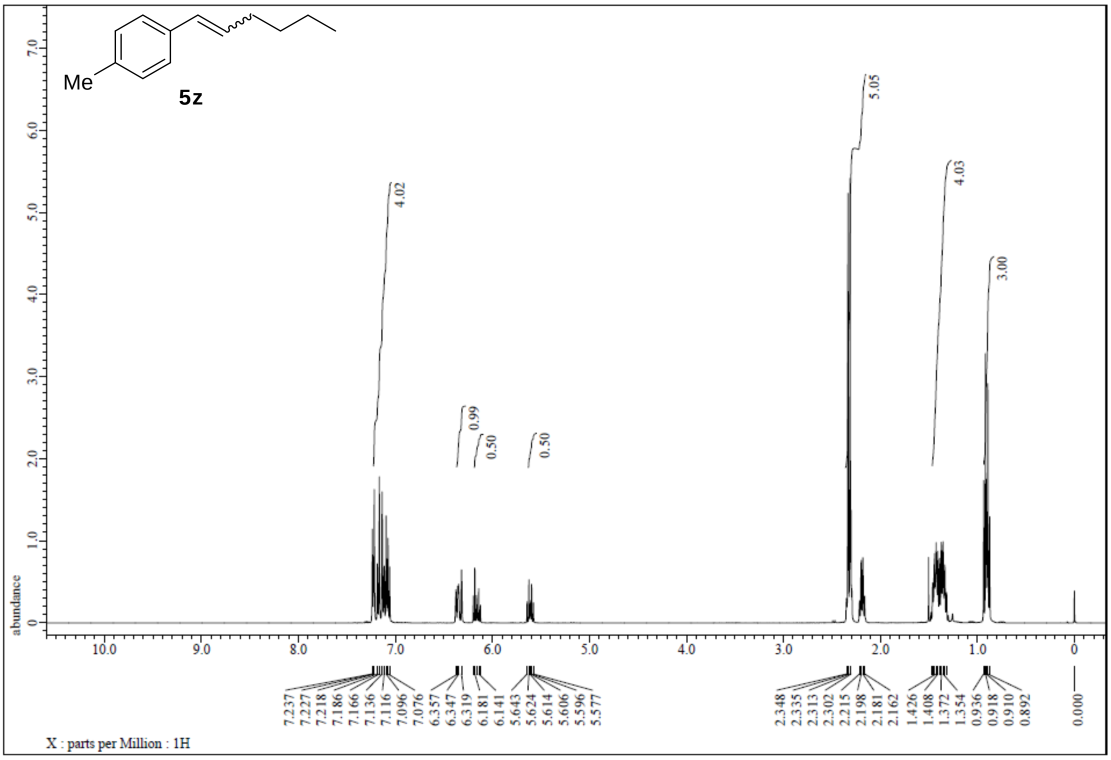

Figure S160. ¹H NMR spectrum of 5z, related to Figure 4.

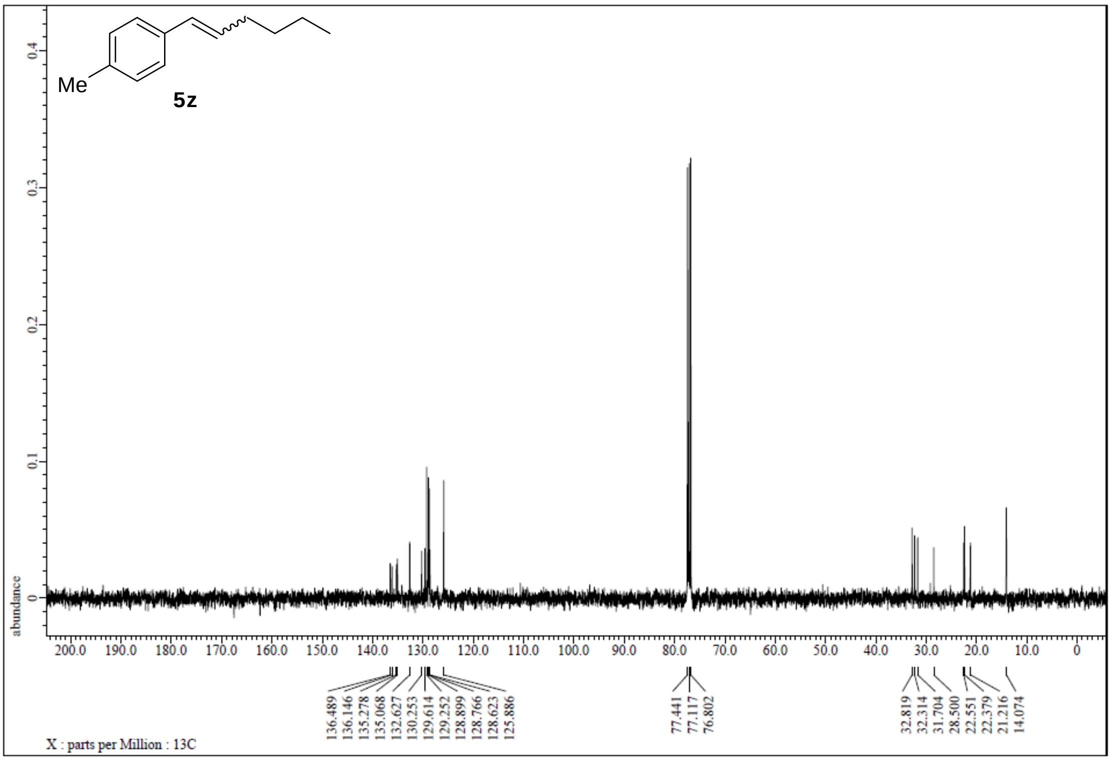

Figure S161. ¹³C NMR spectrum of 5z, related to Figure 4.

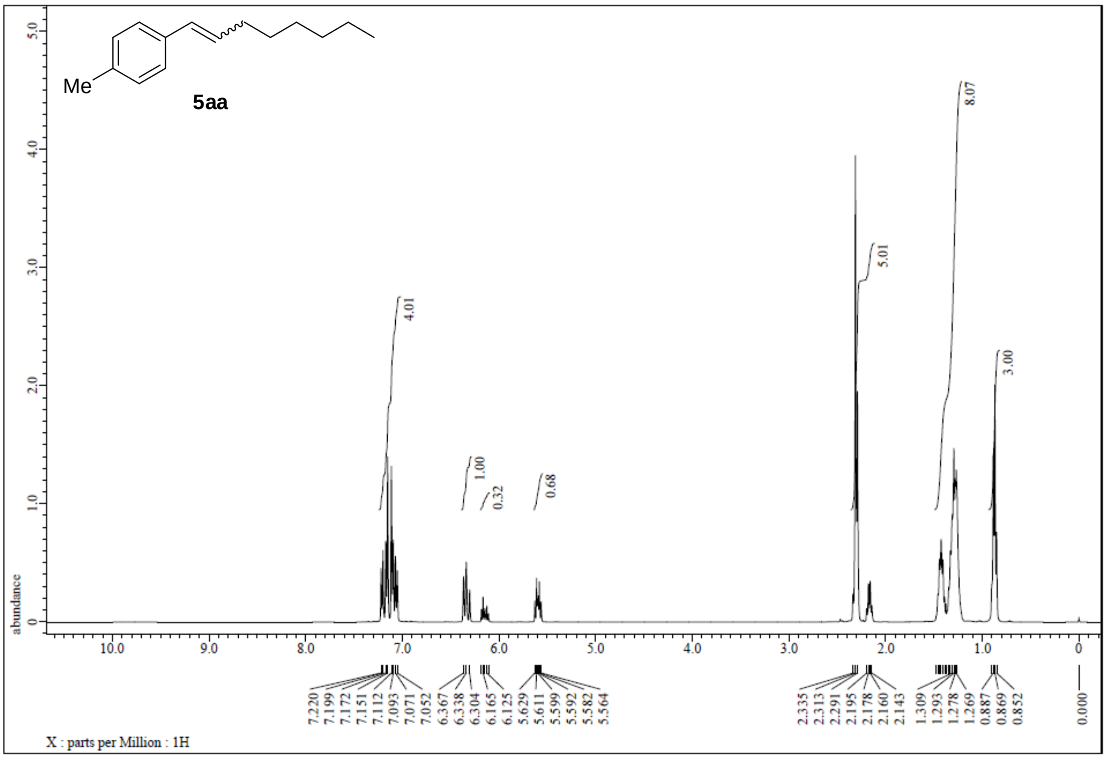

Figure S162. ^{1}H NMR spectrum of 5aa, related to Figure 4.

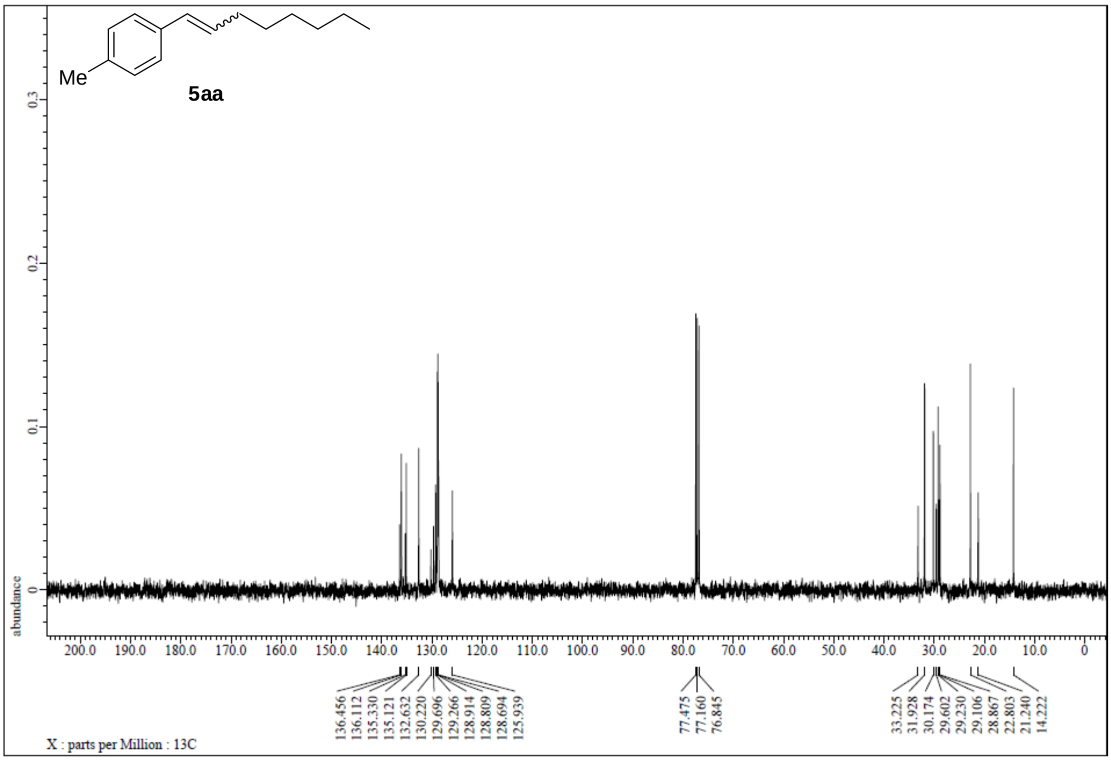

Figure S163. ^{13}C NMR spectrum of 5aa, related to Figure 4.

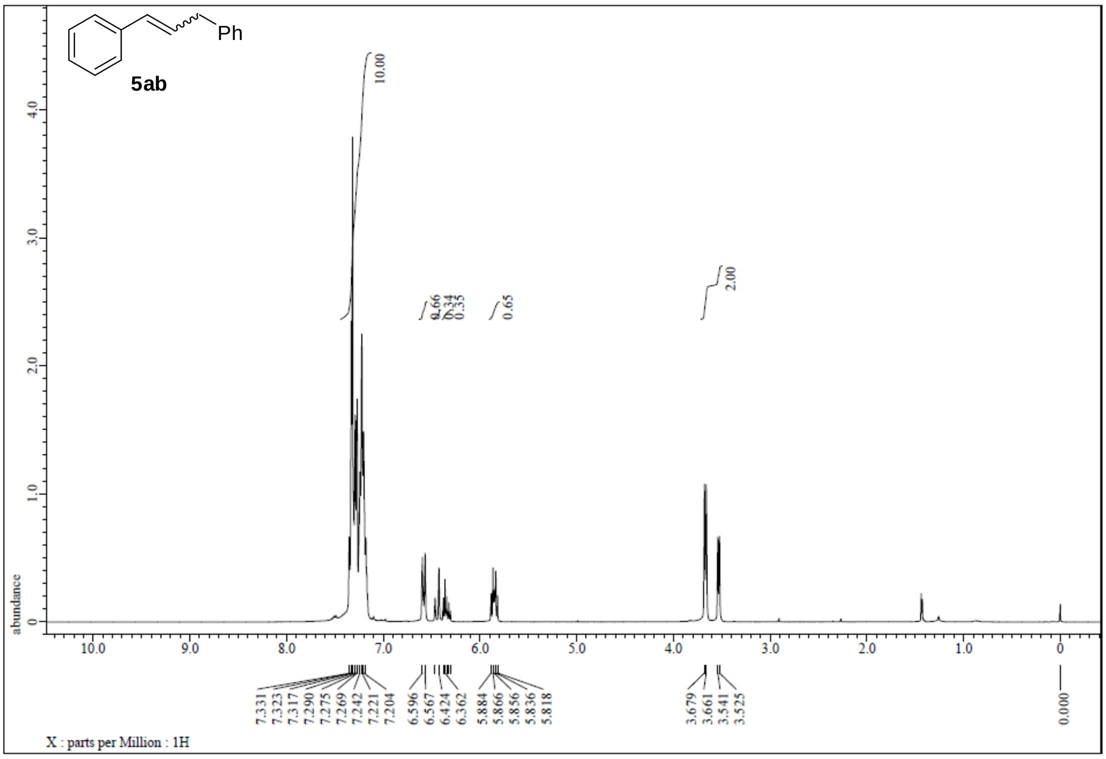

Figure S164. ^{1}H NMR spectrum of 5ab, related to Figure 4.

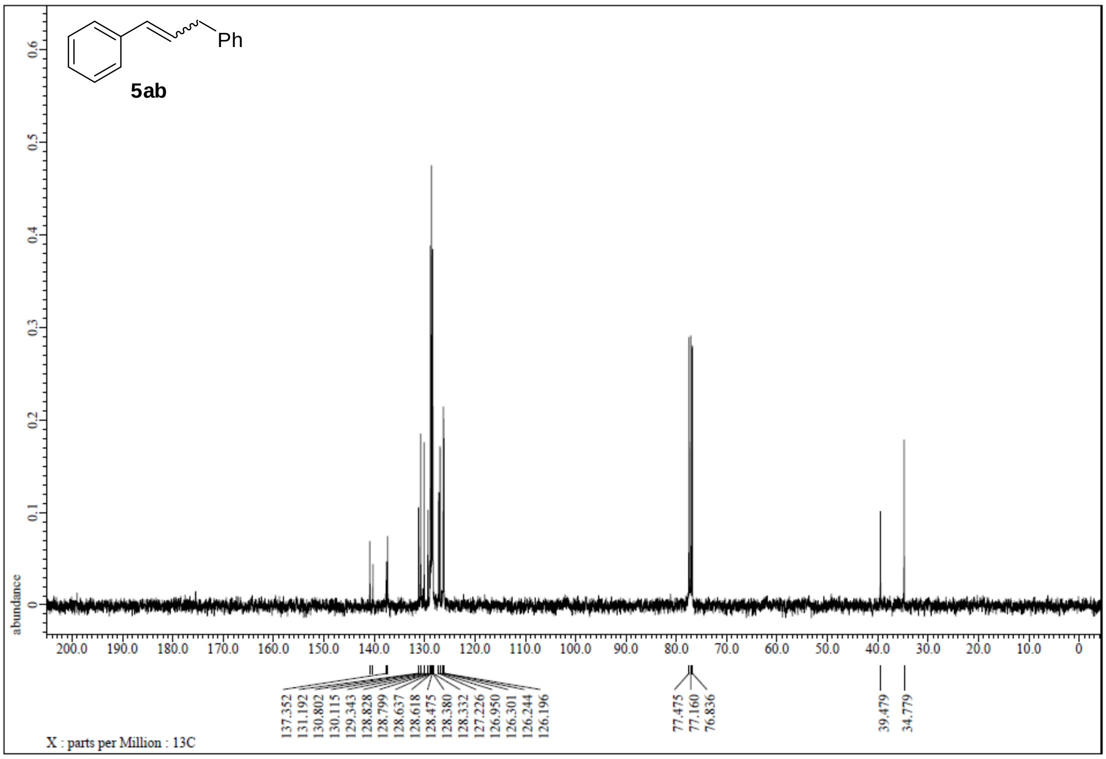

Figure S165. ^{13}C NMR spectrum of 5ab, related to Figure 4.

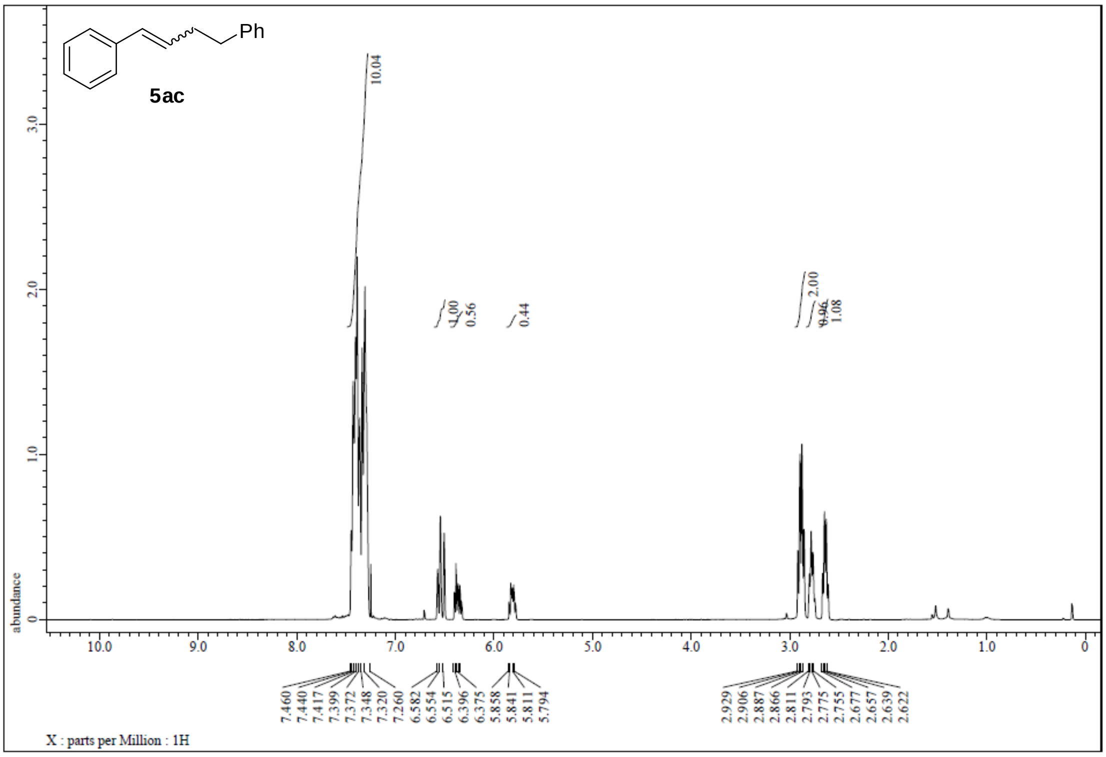

Figure S166. **^{1}H NMR spectrum of 5ac, related to Figure 4**.

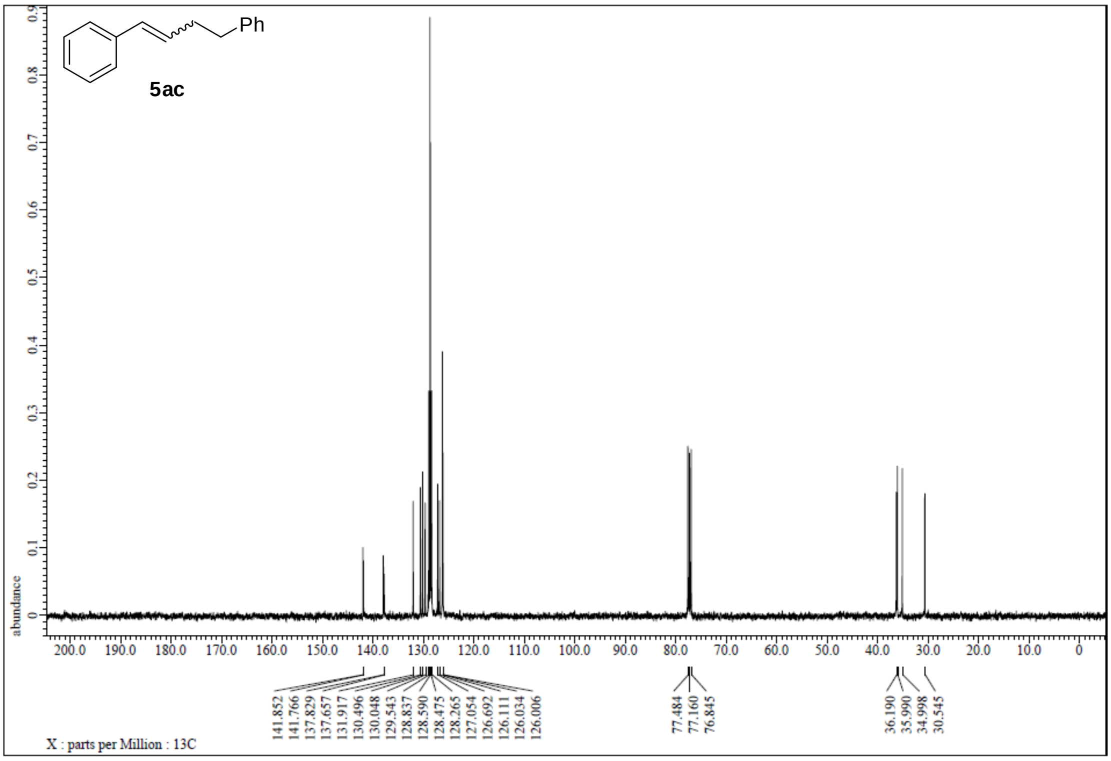

Figure S167. **^{13}C NMR spectrum of 5ac, related to Figure 4**.

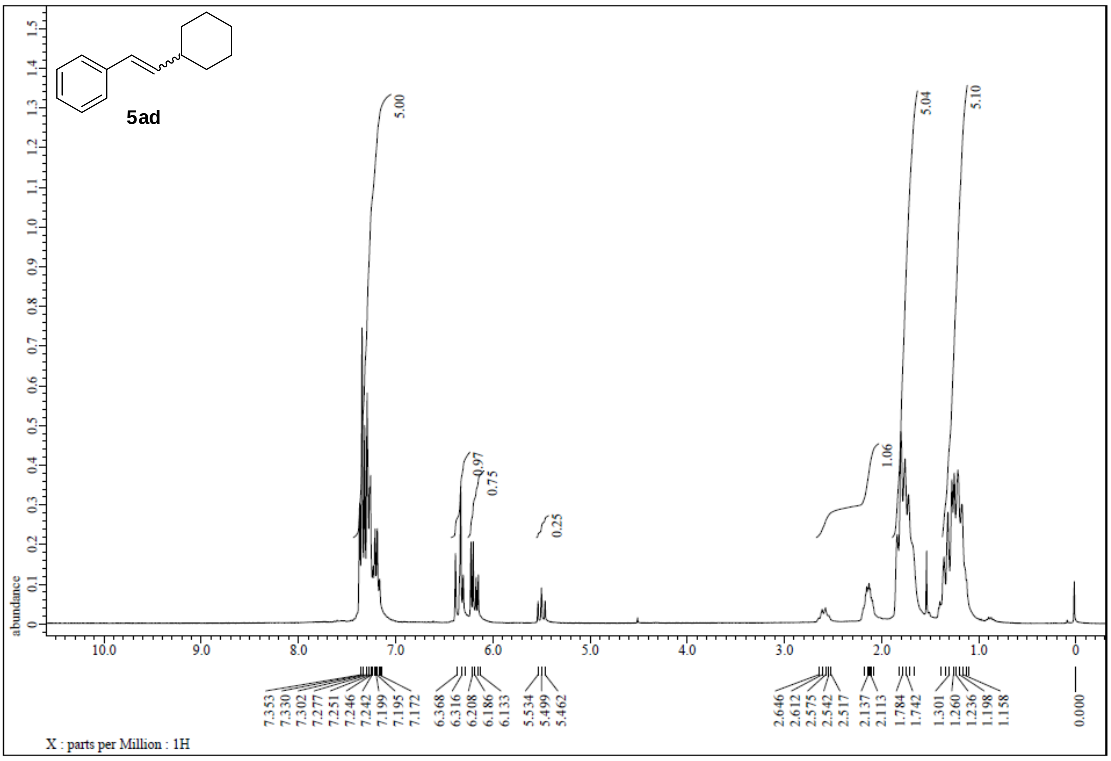

Figure S168. ^{1}H NMR spectrum of 5ad, related to Figure 4.

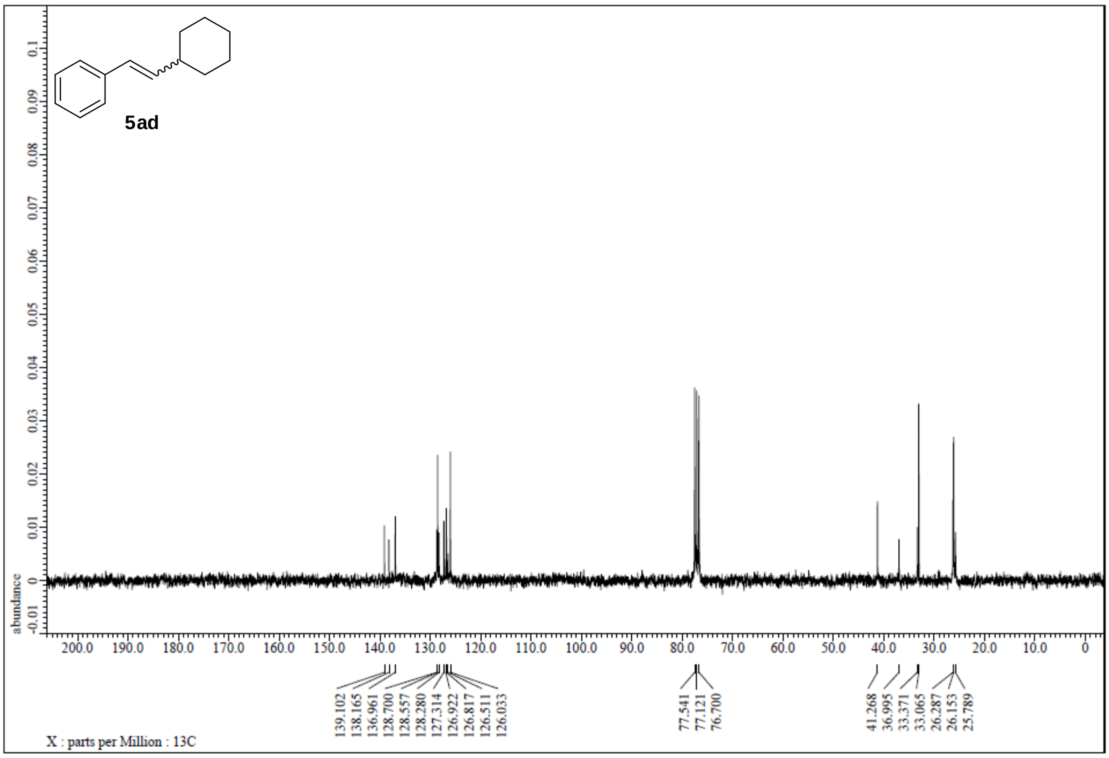

Figure S169. ^{13}C NMR spectrum of 5ad, related to Figure 4.

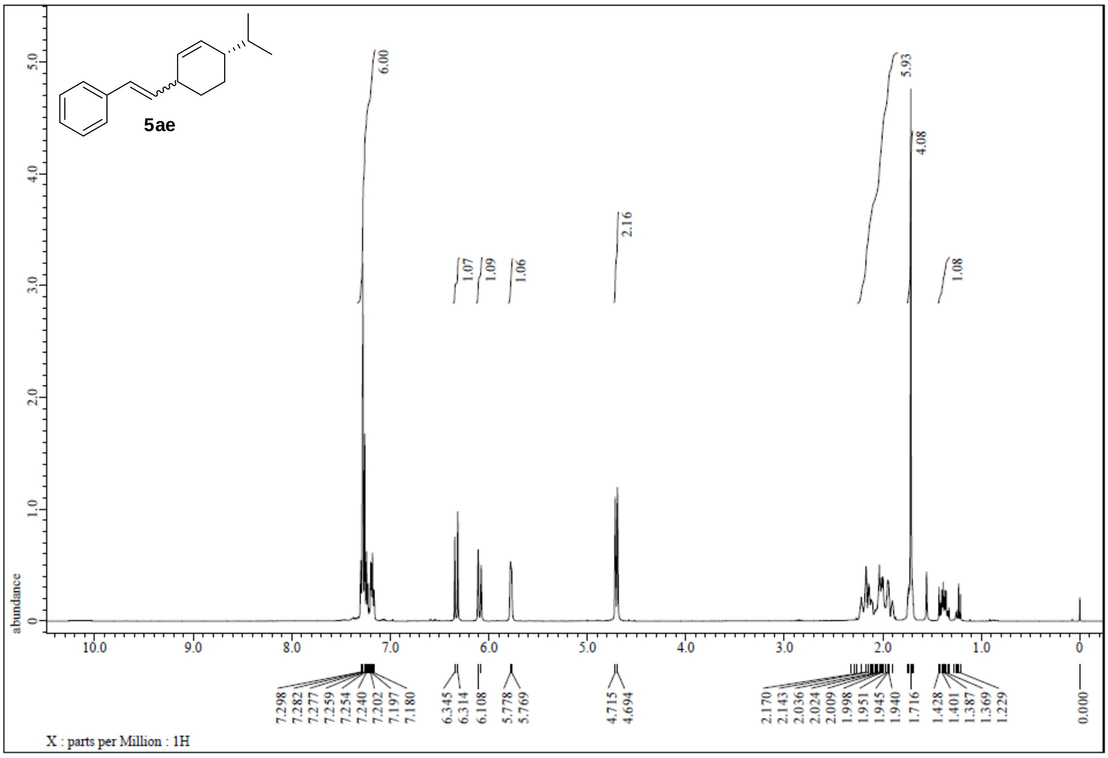

Figure S170. **^{1}H NMR spectrum of 5ae, related to Figure 4**.

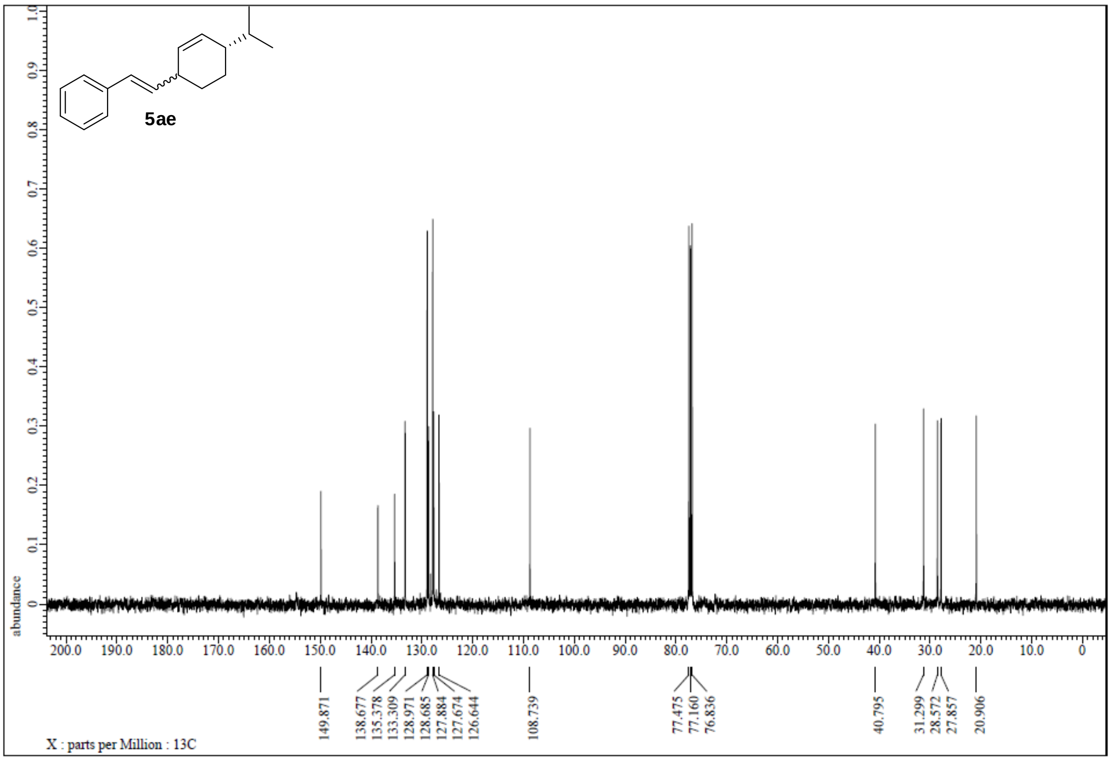

Figure S171. **^{13}C NMR spectrum of 5ae, related to Figure 4**.

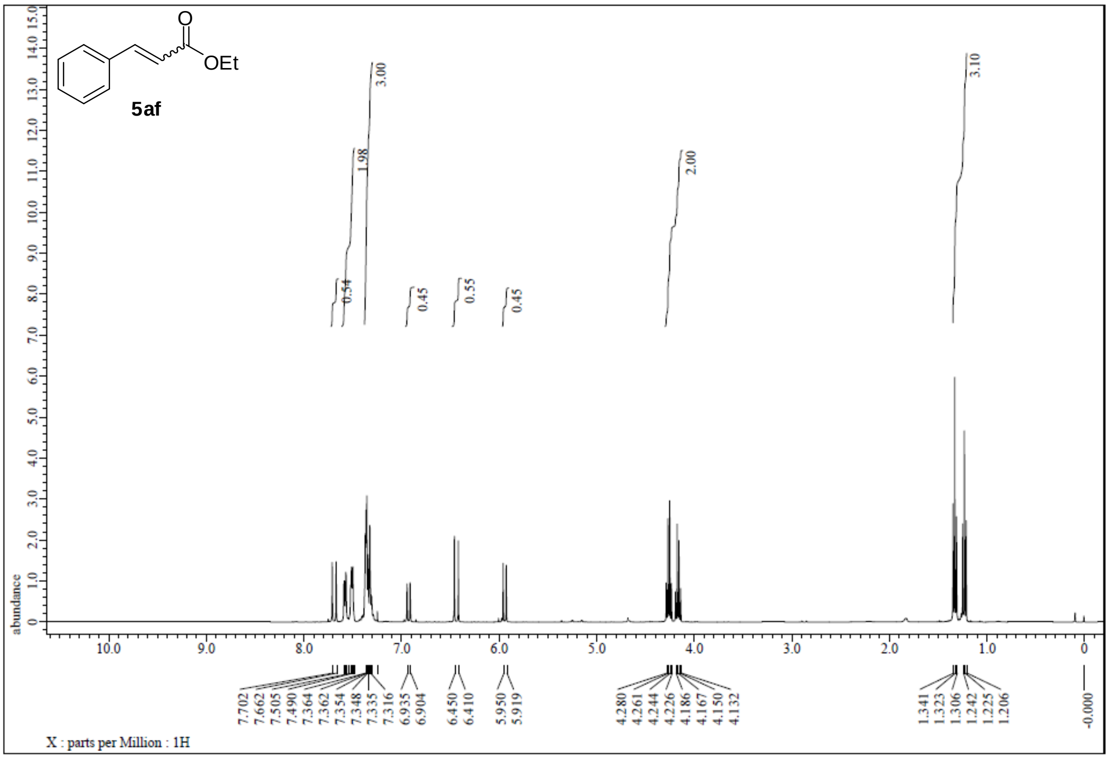

Figure S172. [1]**H NMR spectrum of 5af, related to Figure 4**.

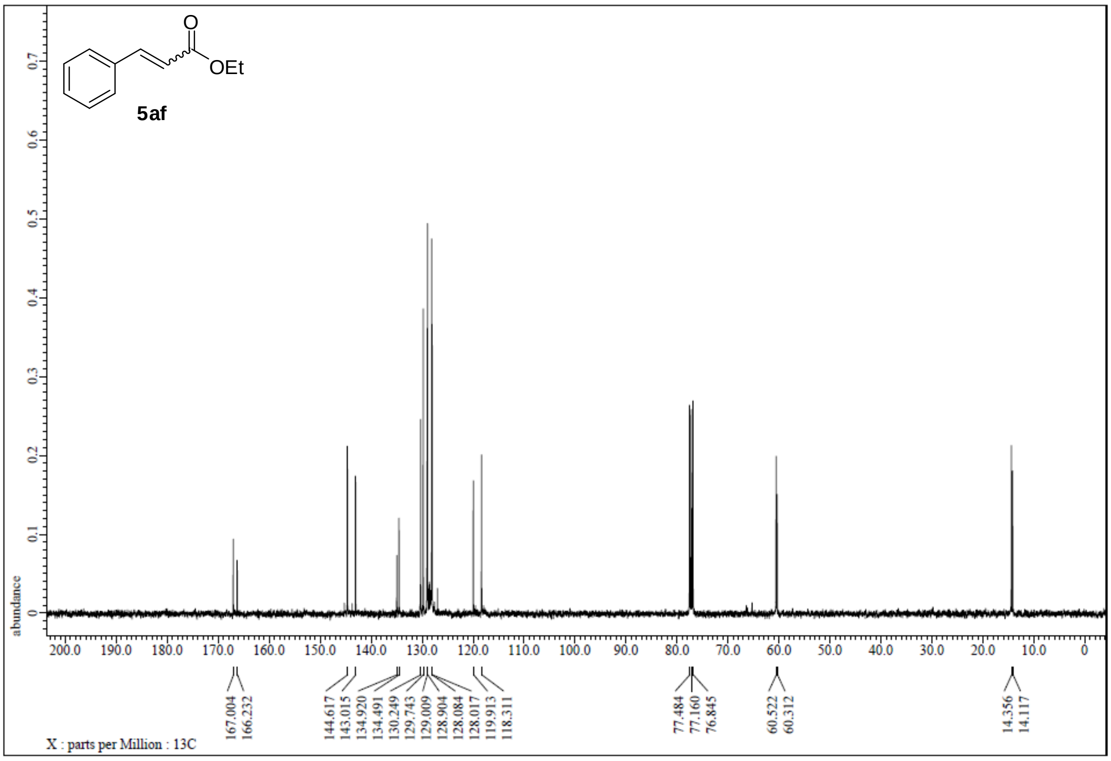

Figure S173. [13]**C NMR spectrum of 5af, related to Figure 4**.

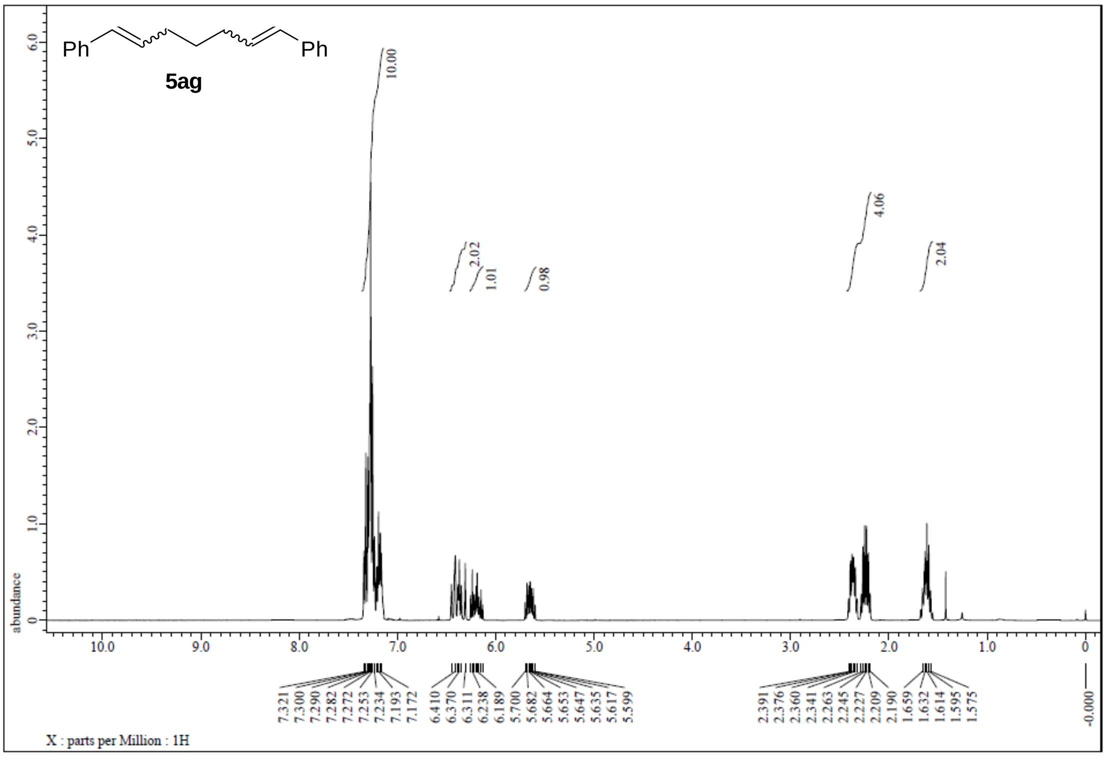

Figure S174. ^{1}H NMR spectrum of 5ag, related to Figure 4.

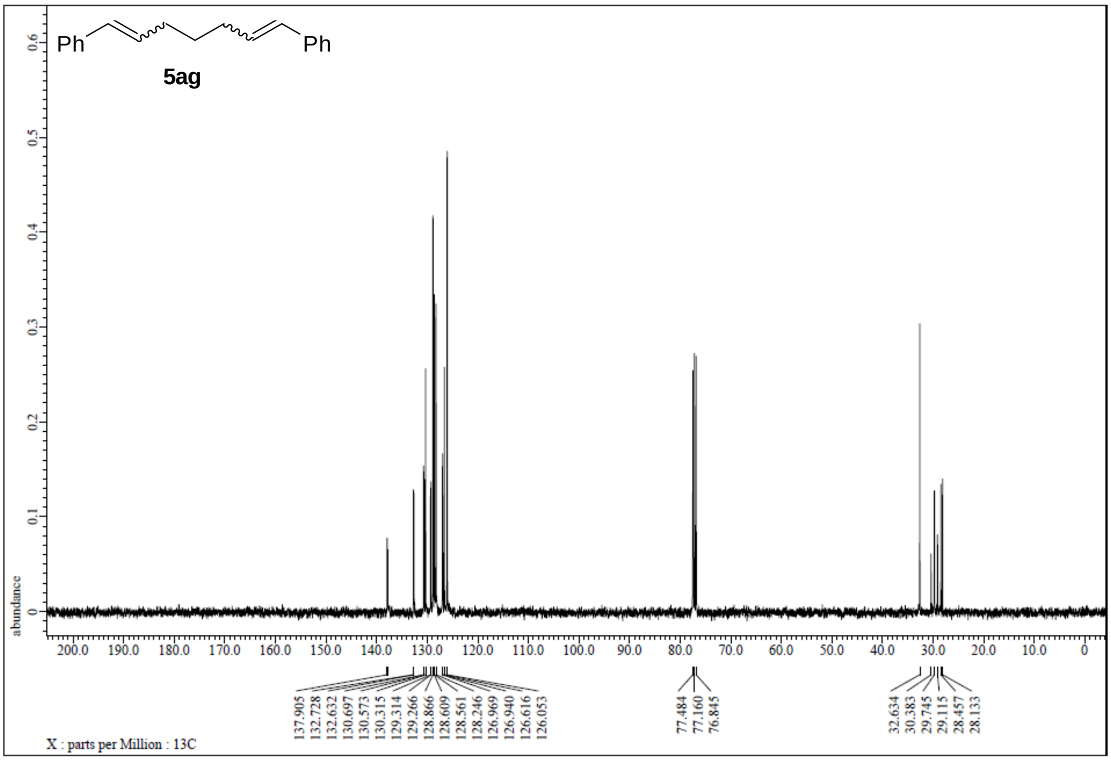

Figure S175. ^{13}C NMR spectrum of 5ag, related to Figure 4.

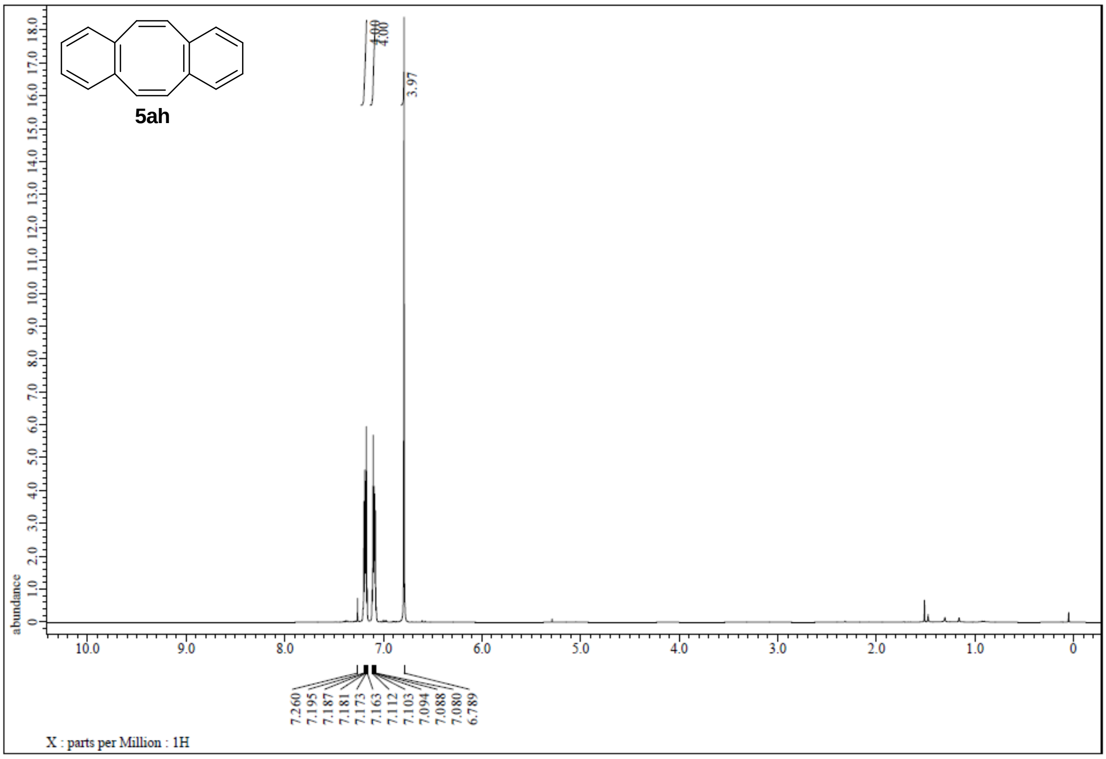

Figure S176. **^{1}H NMR spectrum of 5ah, related to Figure 4**.

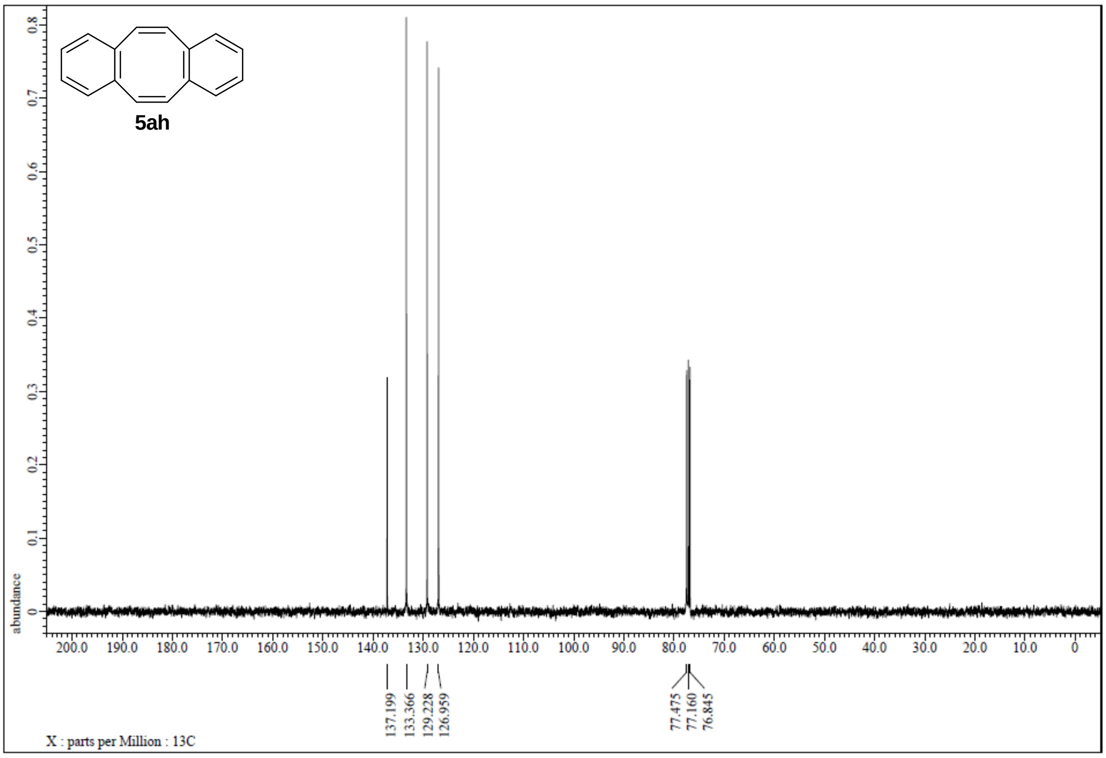

Figure S177. **^{13}C NMR spectrum of 5ah, related to Figure 4**.

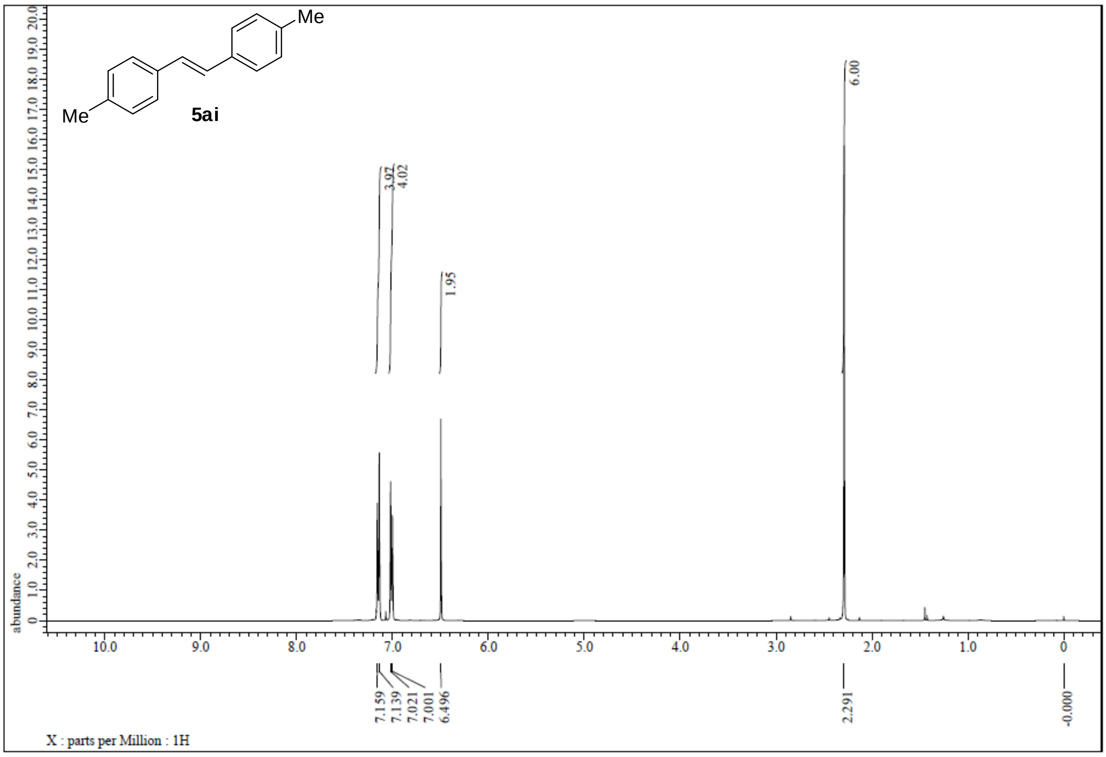

Figure S178. **^{1}H NMR spectrum of 5ai, related to Figure 5**.

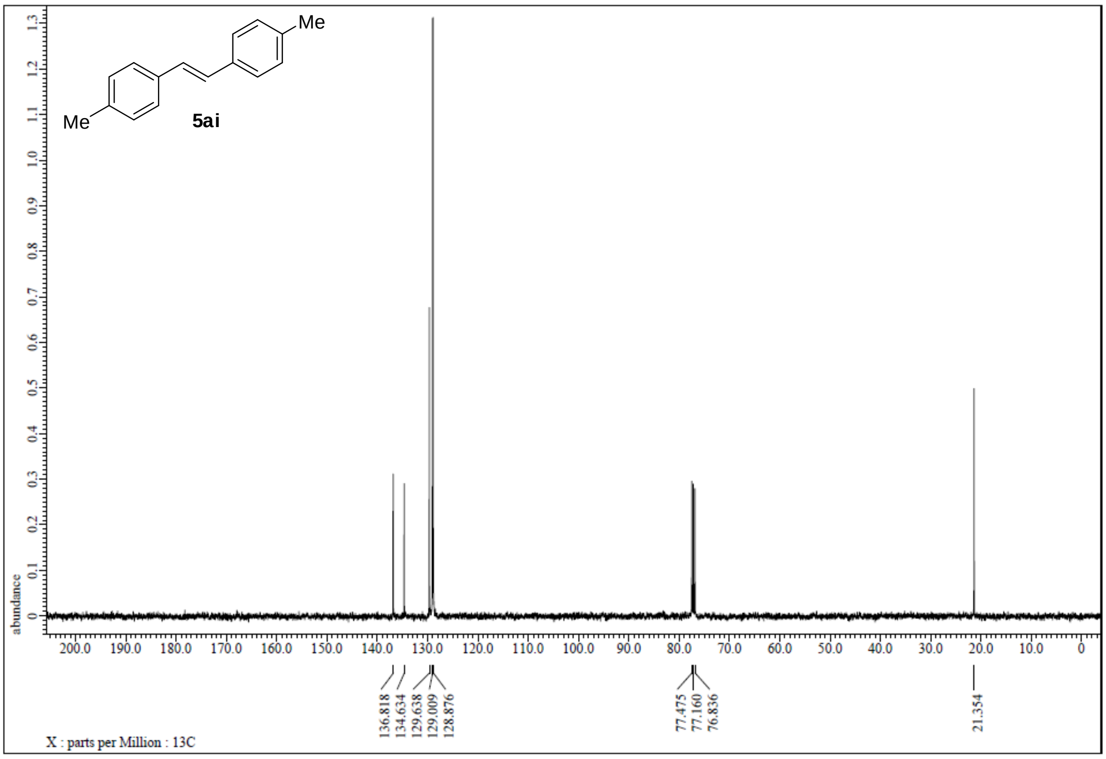

Figure S179. **^{13}C NMR spectrum of 5ai, related to Figure 5**.

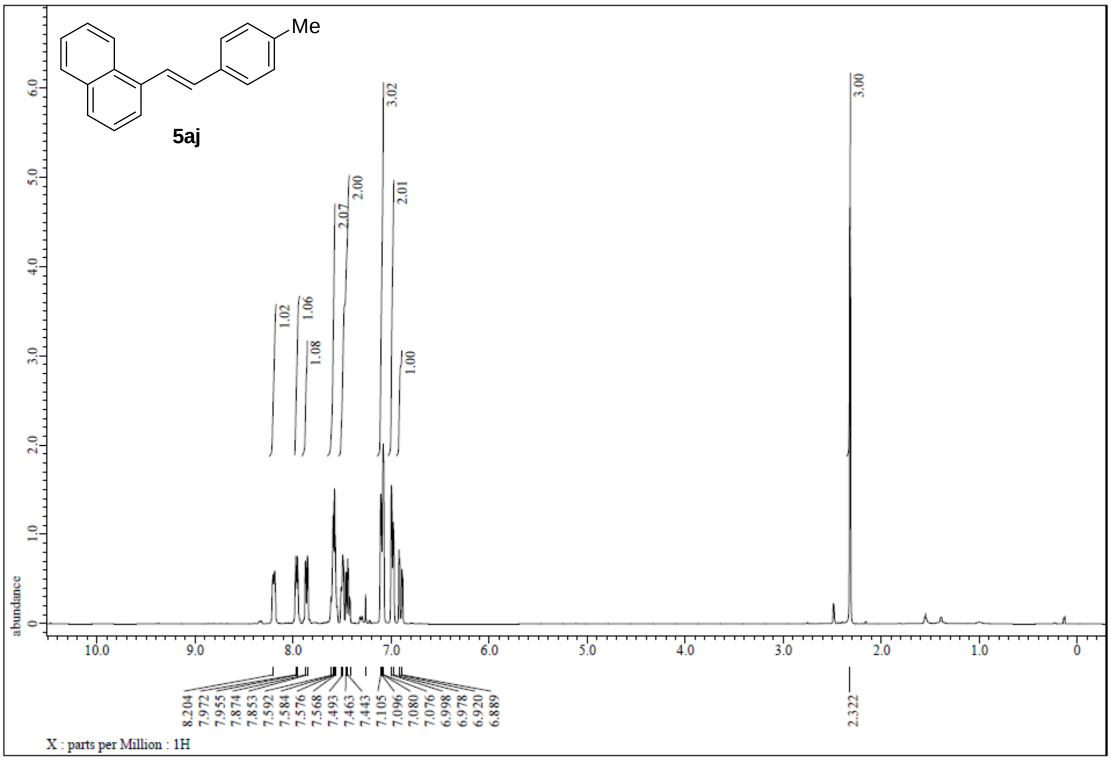

Figure S180. ^{1}**H NMR spectrum of 5aj, related to Figure 5**.

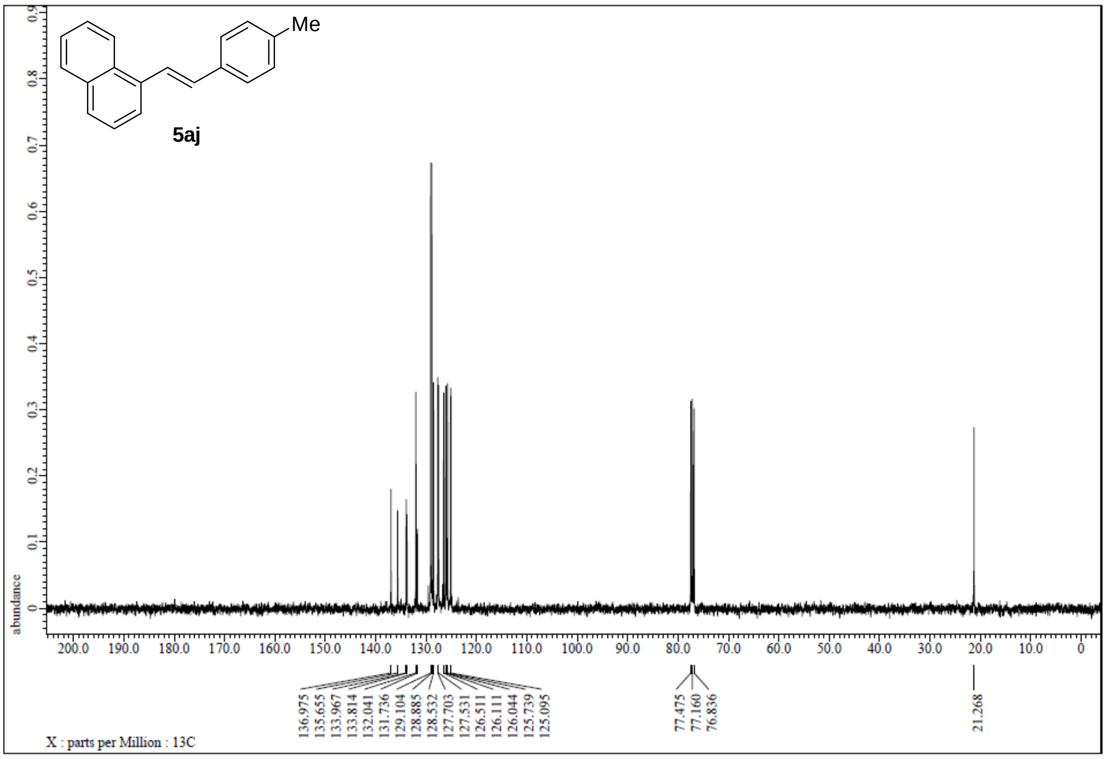

Figure S181. ^{13}**C NMR spectrum of 5aj, related to Figure 5**.

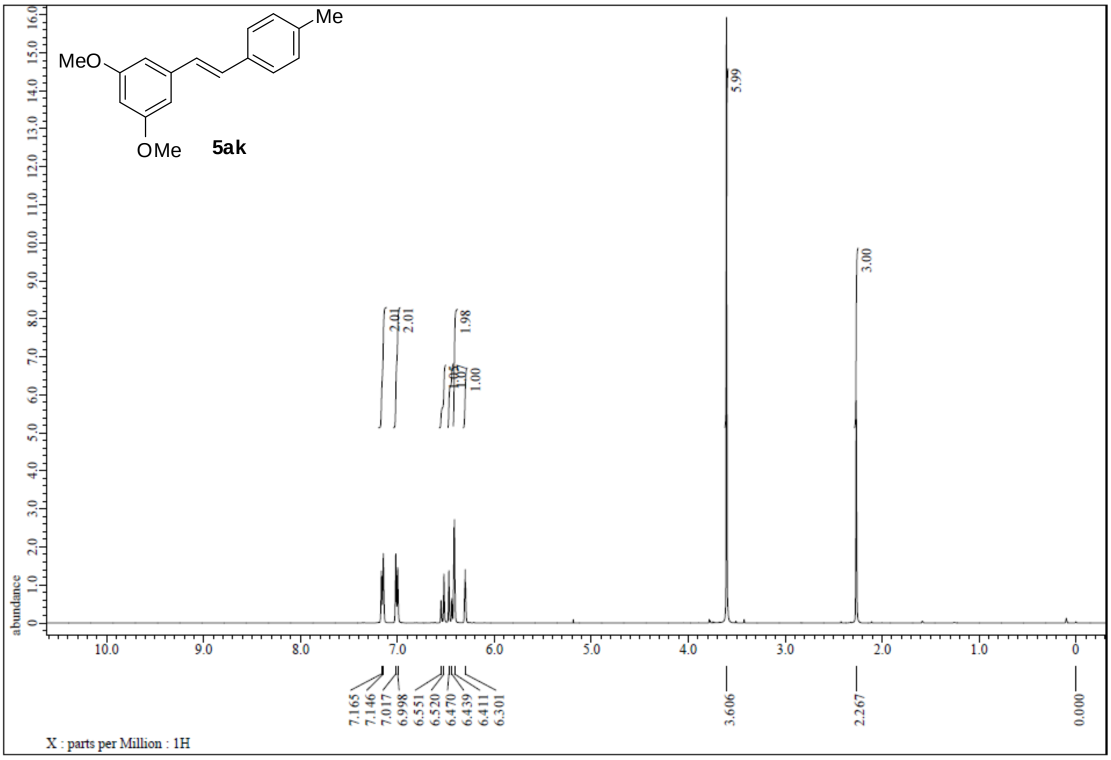

Figure S182. ^{1}**H NMR spectrum of 5ak, related to Figure 5**.

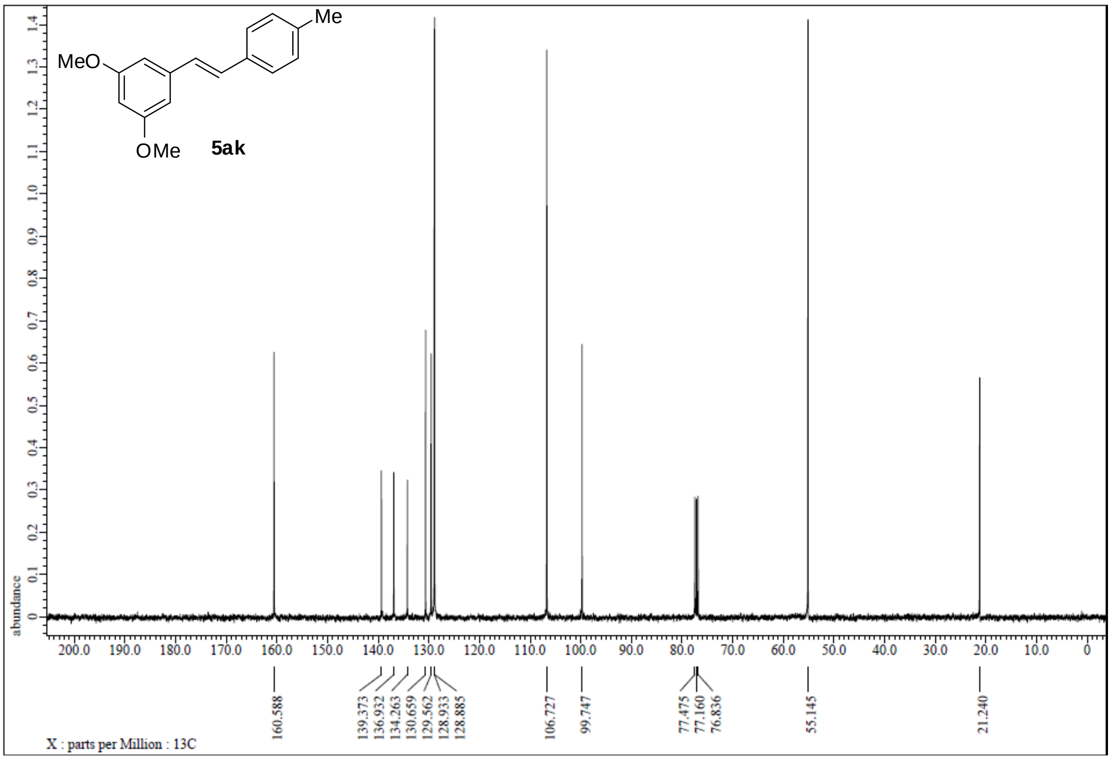

Figure S183. ^{13}**C NMR spectrum of 5ak, related to Figure 5**.

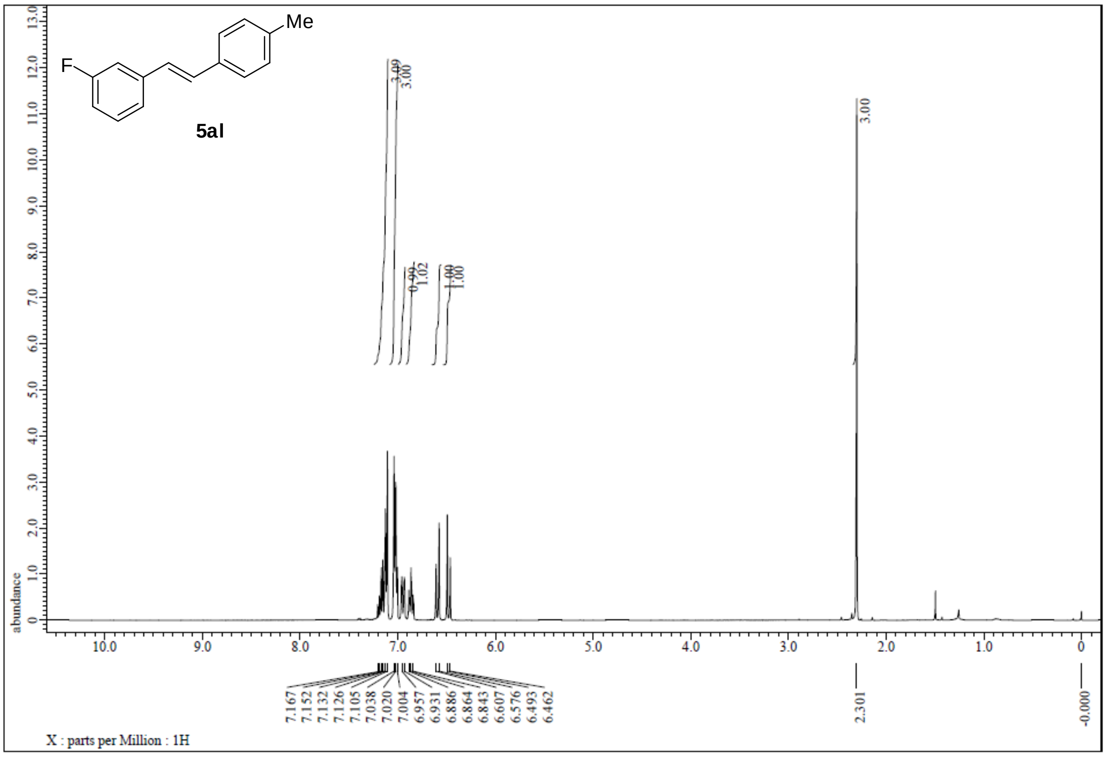

Figure S184. **^{1}H NMR spectrum of 5al, related to Figure 5**.

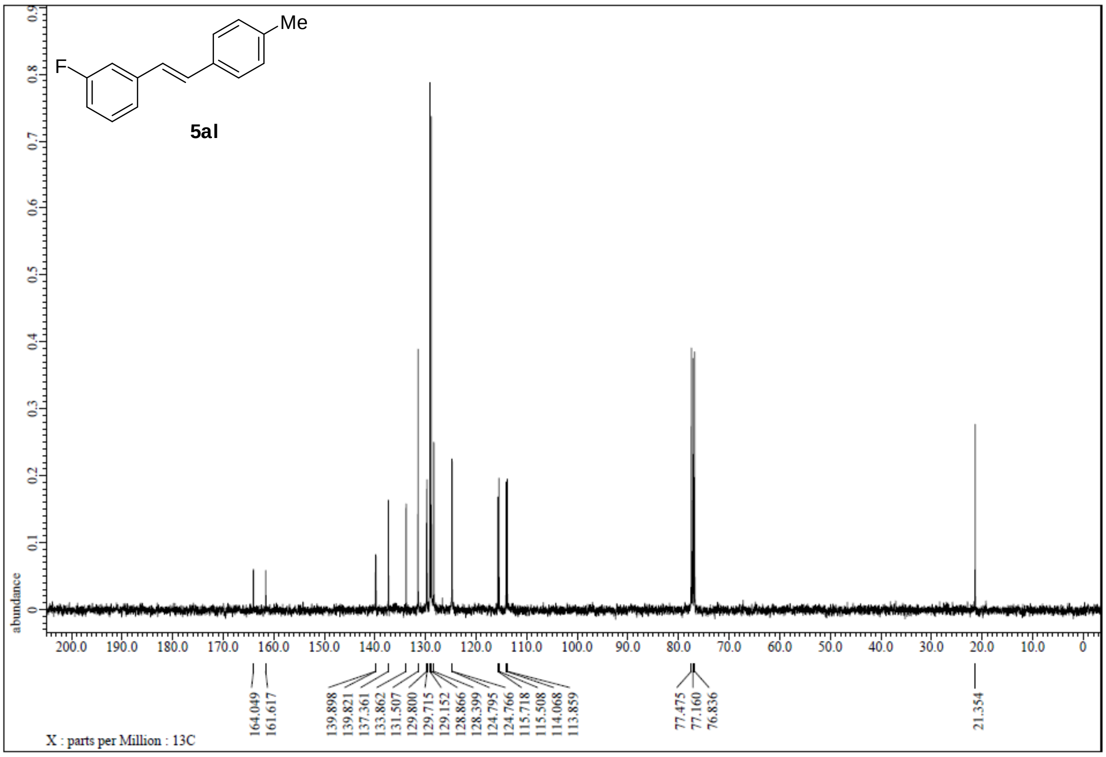

Figure S185. **^{13}C NMR spectrum of 5al, related to Figure 5**.

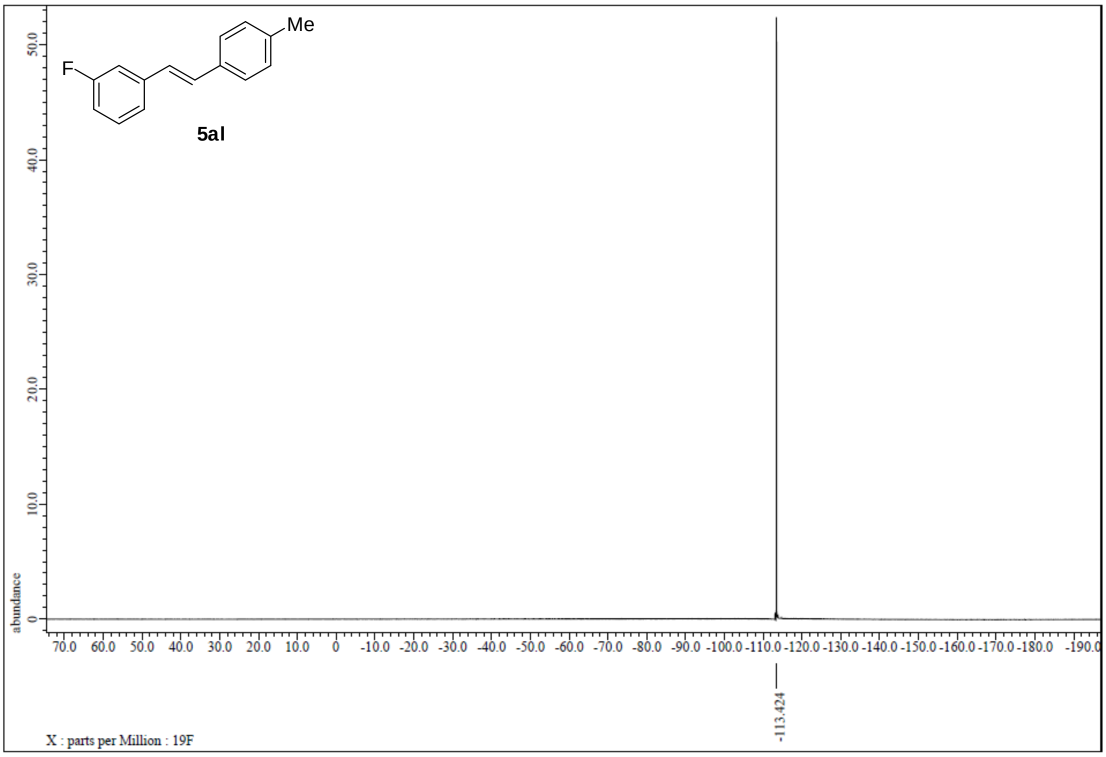

Figure S186. **^{19}F NMR spectrum of 5al, related to Figure 5**.

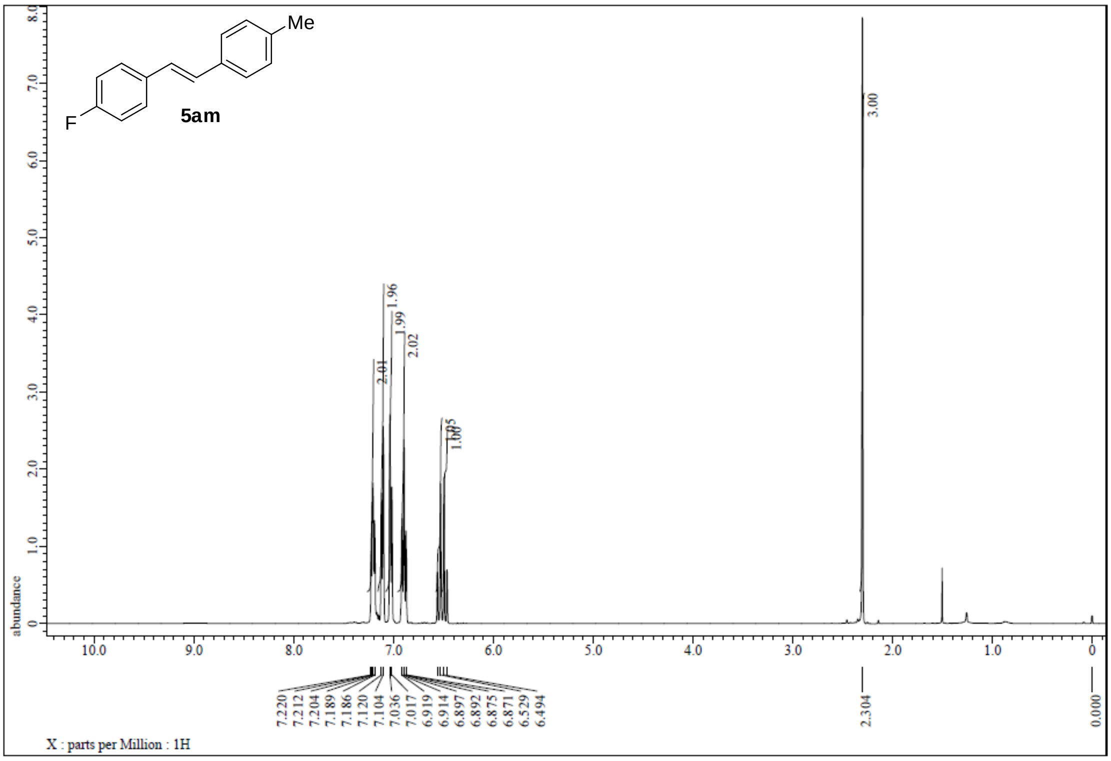

Figure S187. **^{1}H NMR spectrum of 5am, related to Figure 5**.

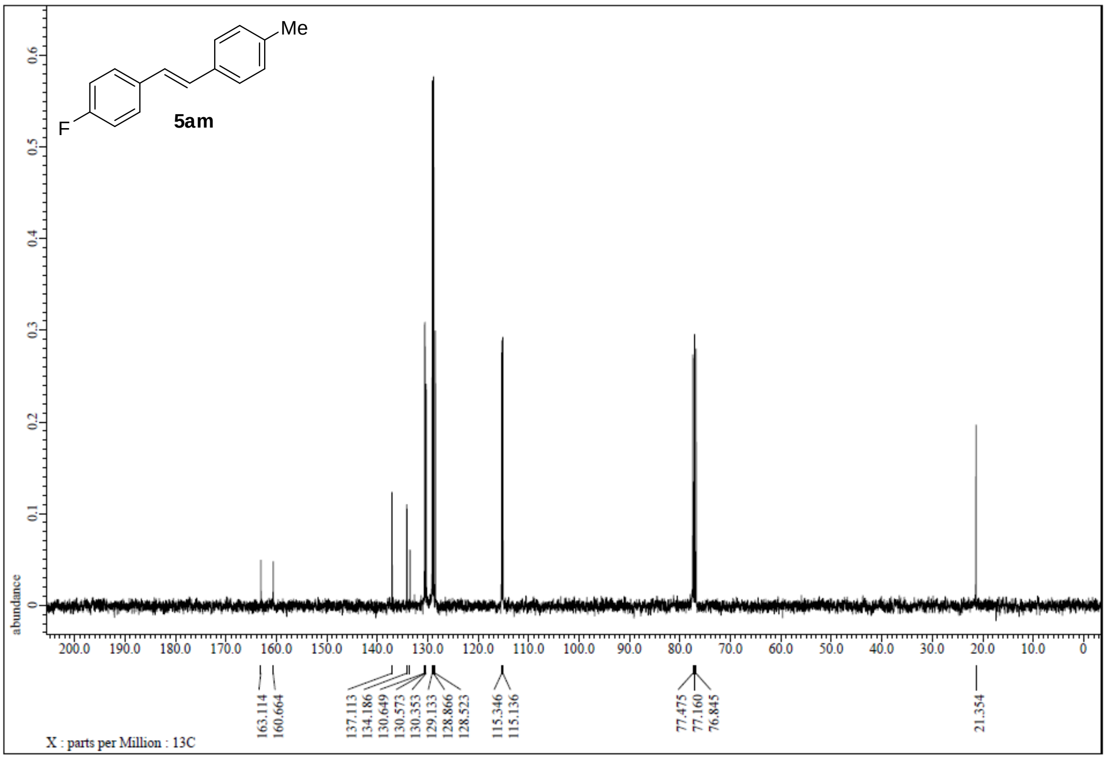

Figure S188. **^{13}C NMR spectrum of 5am, related to Figure 5**.

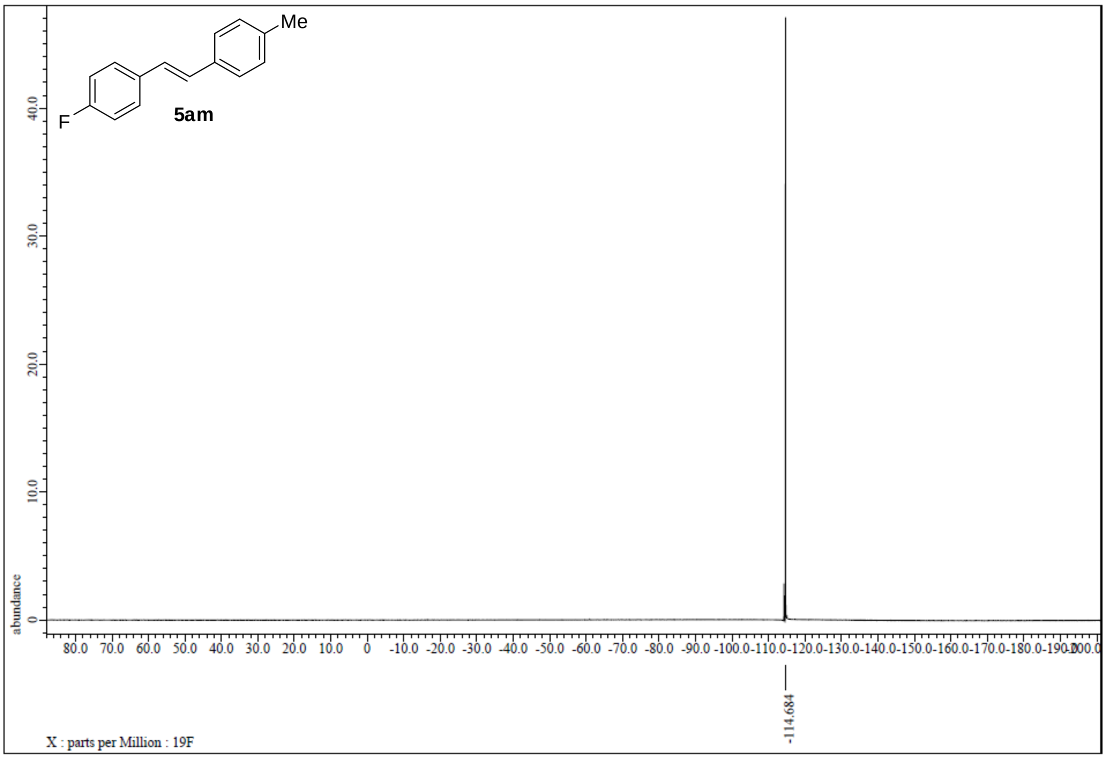

Figure S189. ^{19}F NMR spectrum of 5am, related to Figure 5.

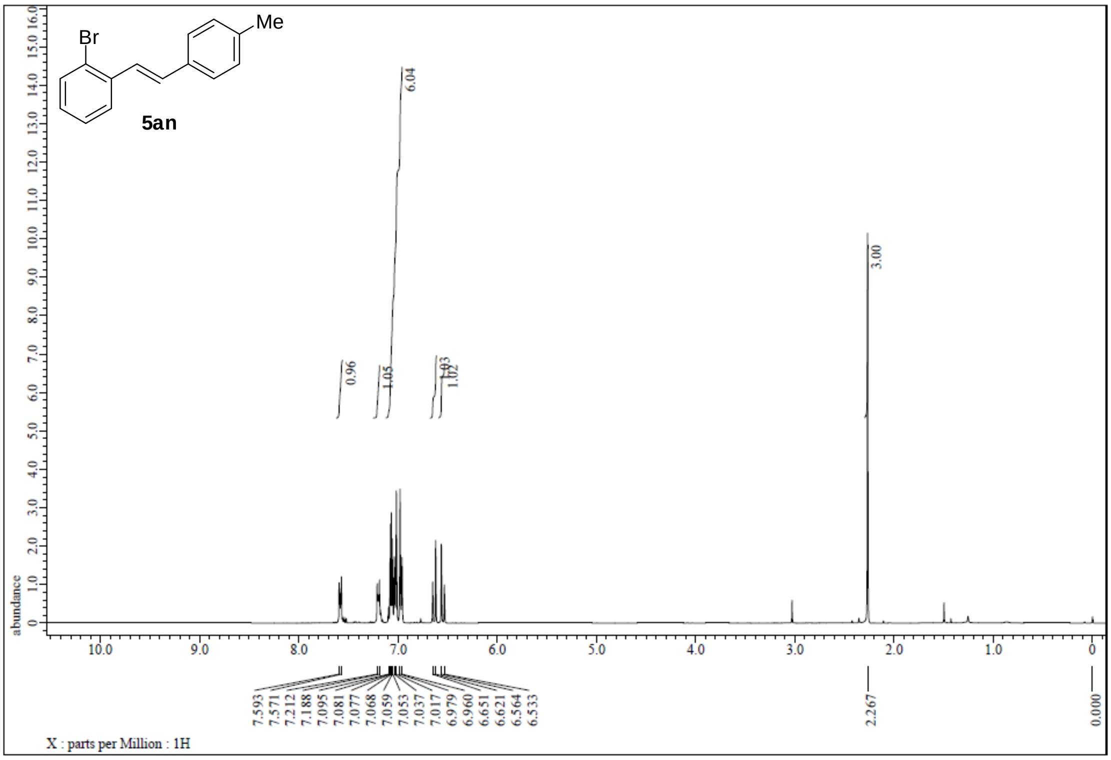

Figure S190. **^{1}H NMR spectrum of 5an, related to Figure 5**.

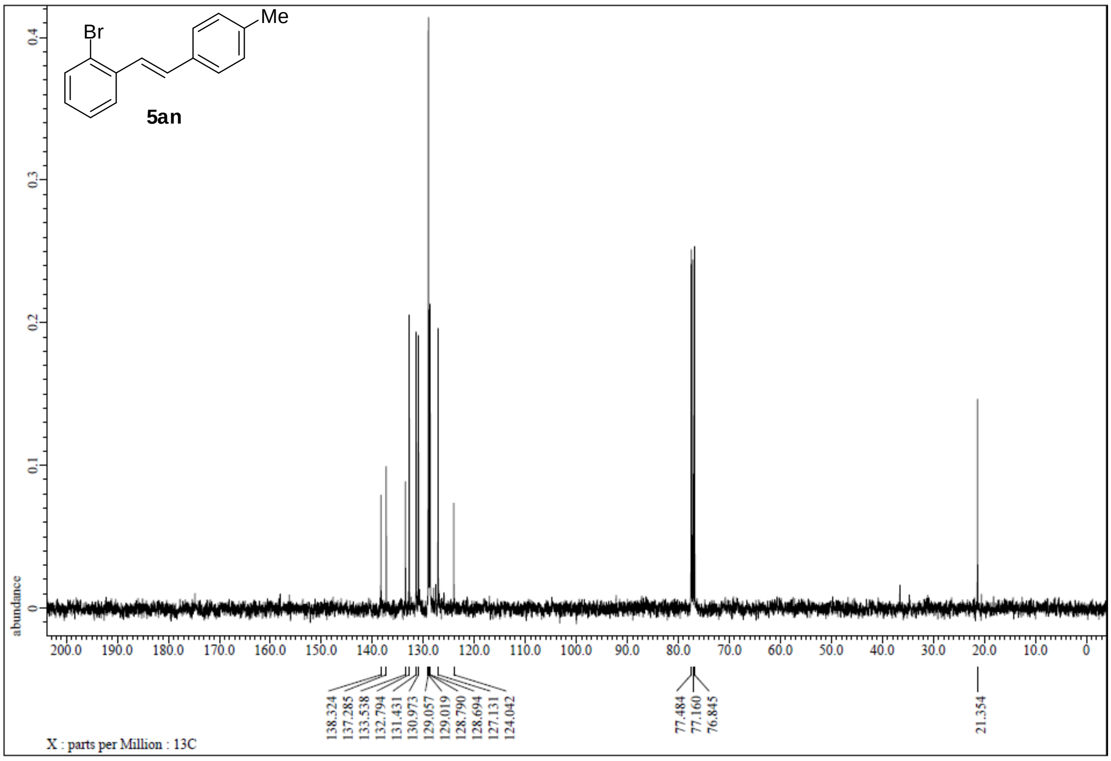

Figure S191. **^{13}C NMR spectrum of 5an, related to Figure 5**.

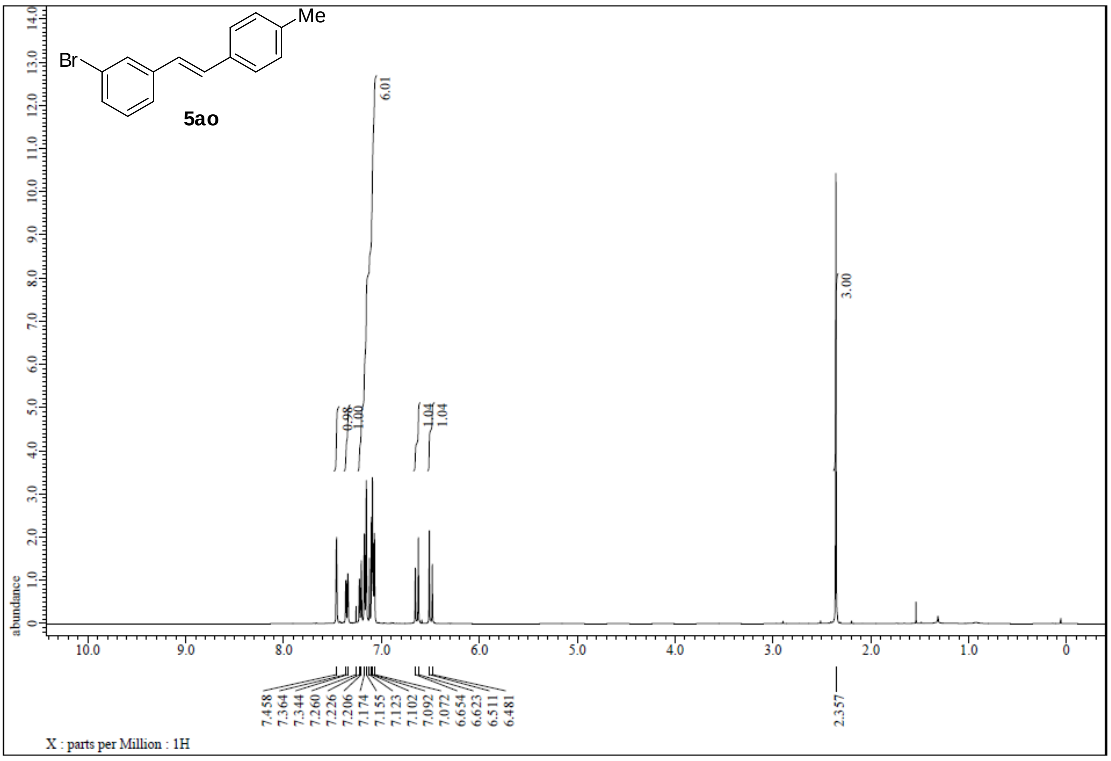

Figure S192. [1]**H NMR spectrum of 5ao, related to Figure 5**.

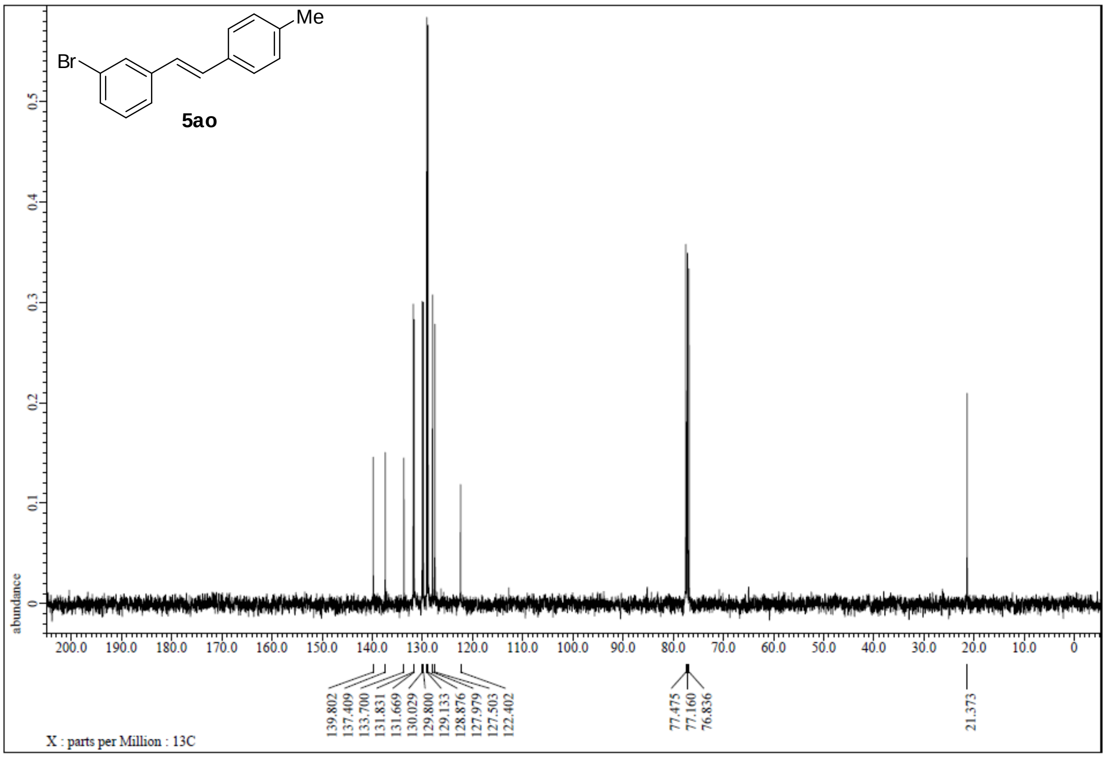

Figure S193. [13]**C NMR spectrum of 5ao, related to Figure 5**.

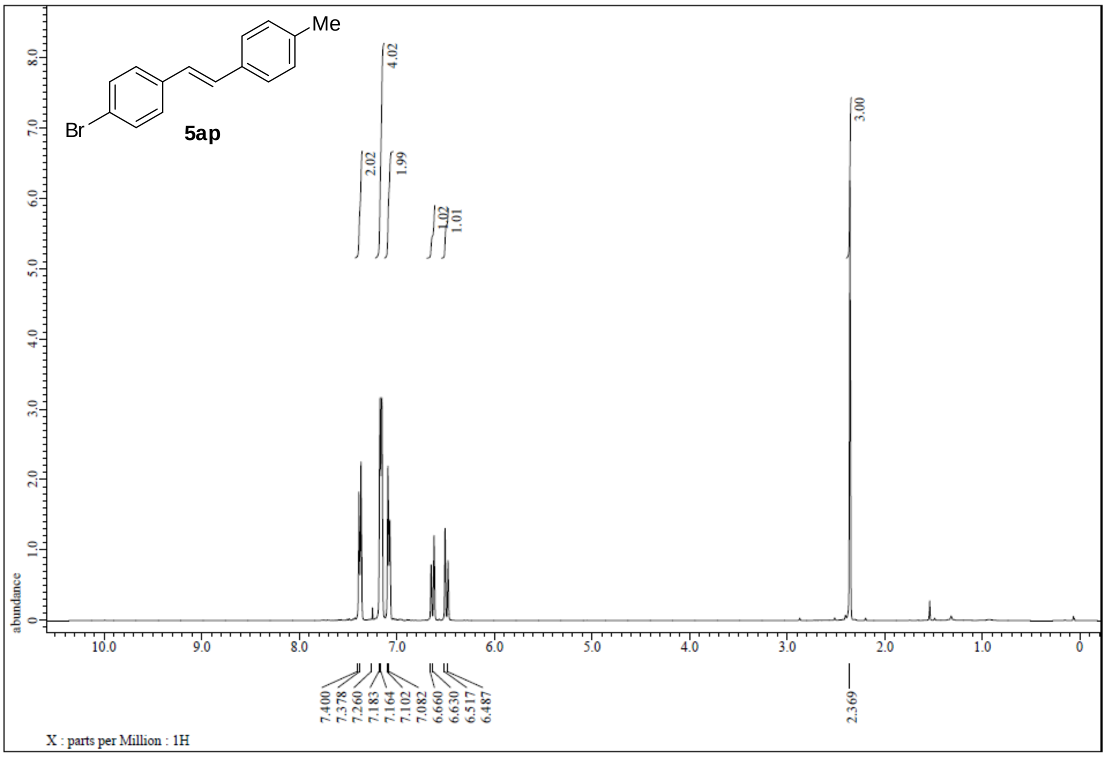

Figure S194. ^{1}**H NMR spectrum of 5ap, related to Figure 5**.

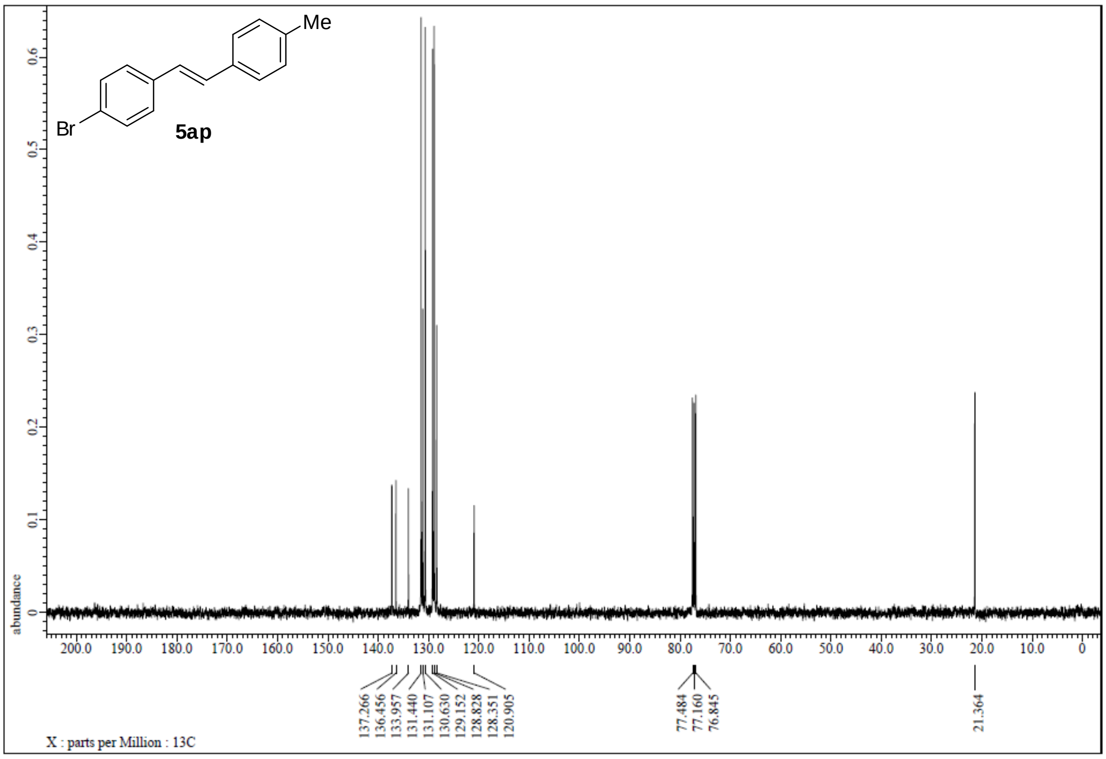

Figure S195. ^{13}**C NMR spectrum of 5ap, related to Figure 5**.

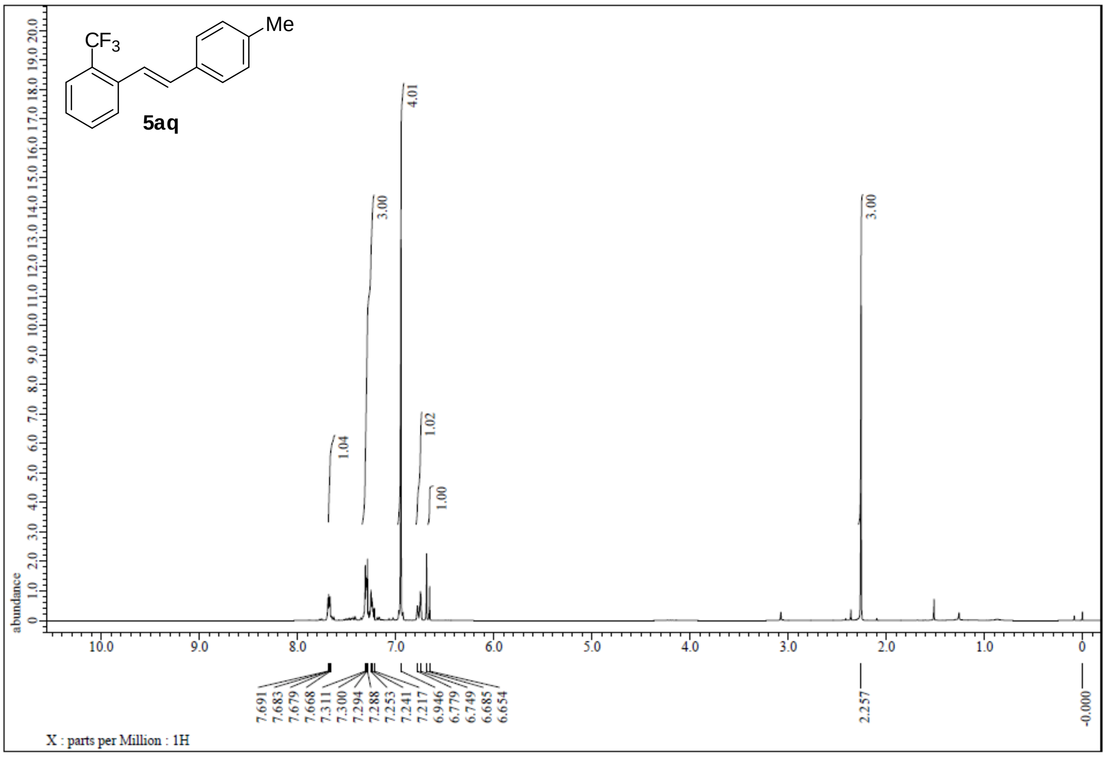

Figure S196. ^{1}H NMR spectrum of 5aq, related to Figure 5.

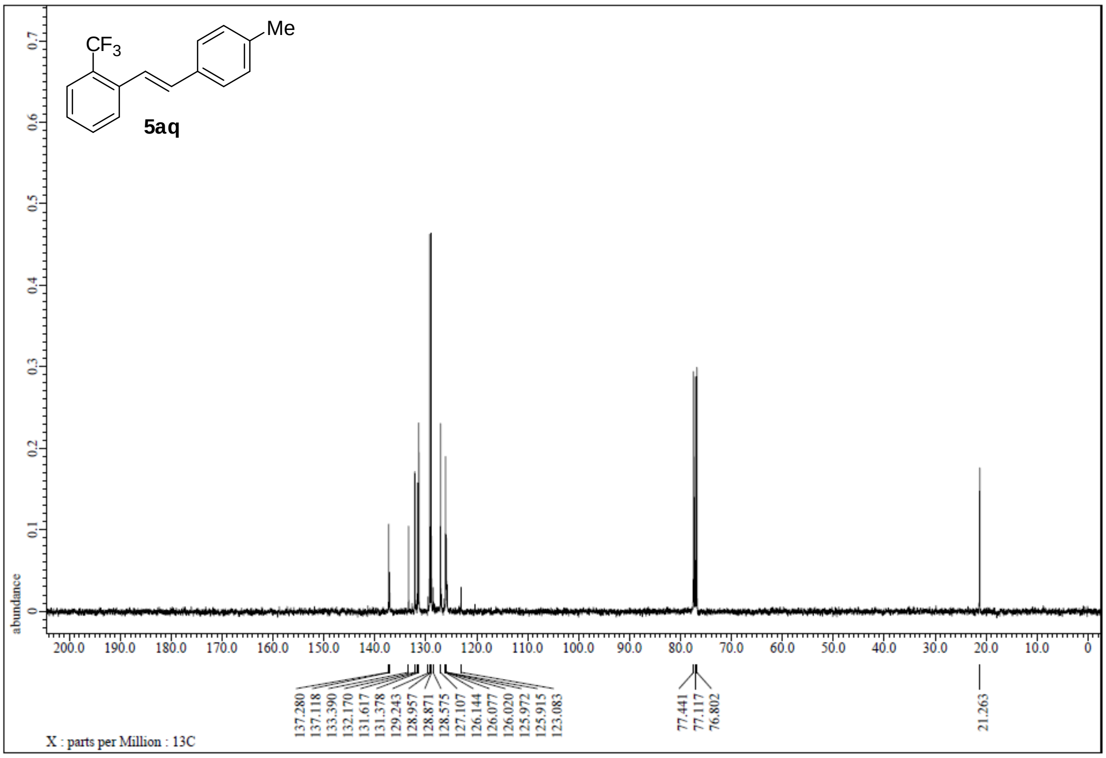

Figure S197. ^{13}C NMR spectrum of 5aq, related to Figure 5.

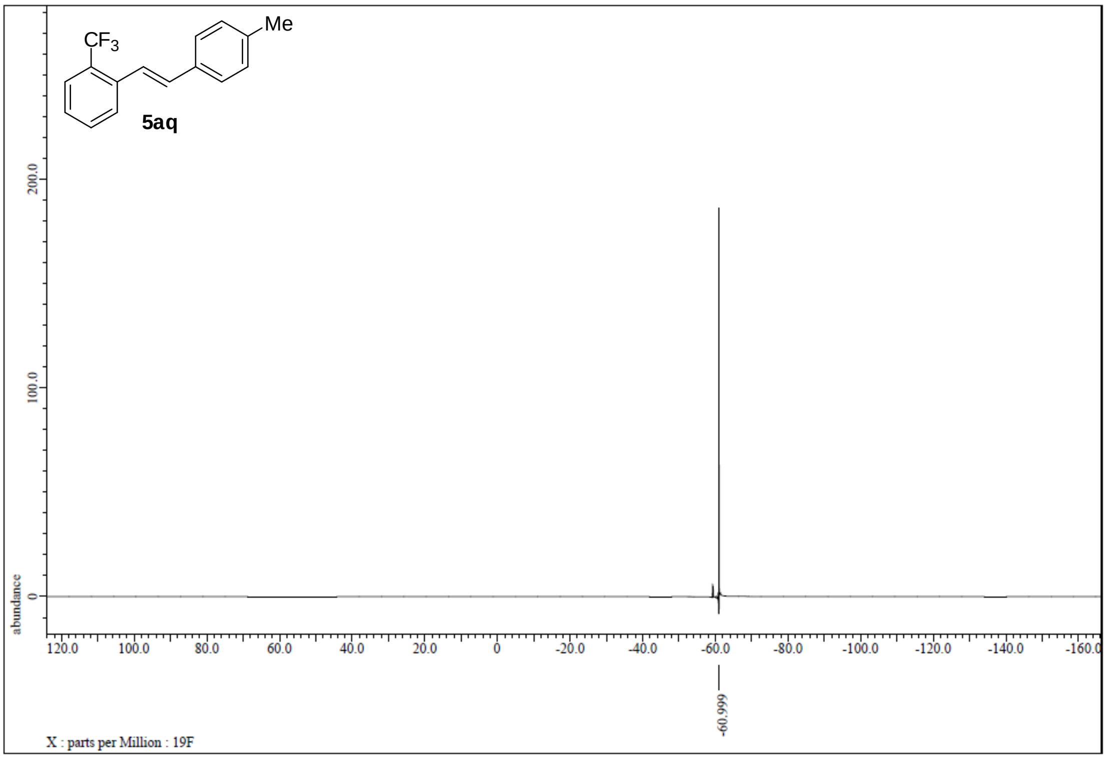

Figure S198. ^{19}F NMR spectrum of 5aq, related to Figure 5.

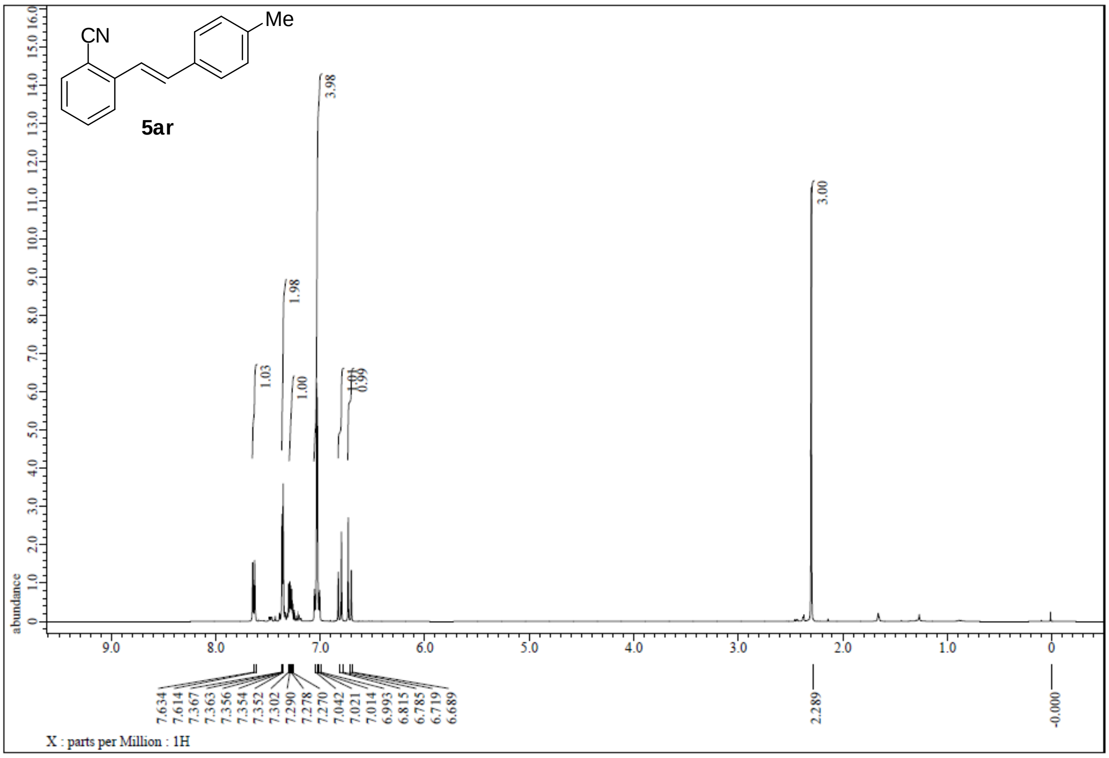

Figure S199. **^{1}H NMR spectrum of 5ar, related to Figure 5**.

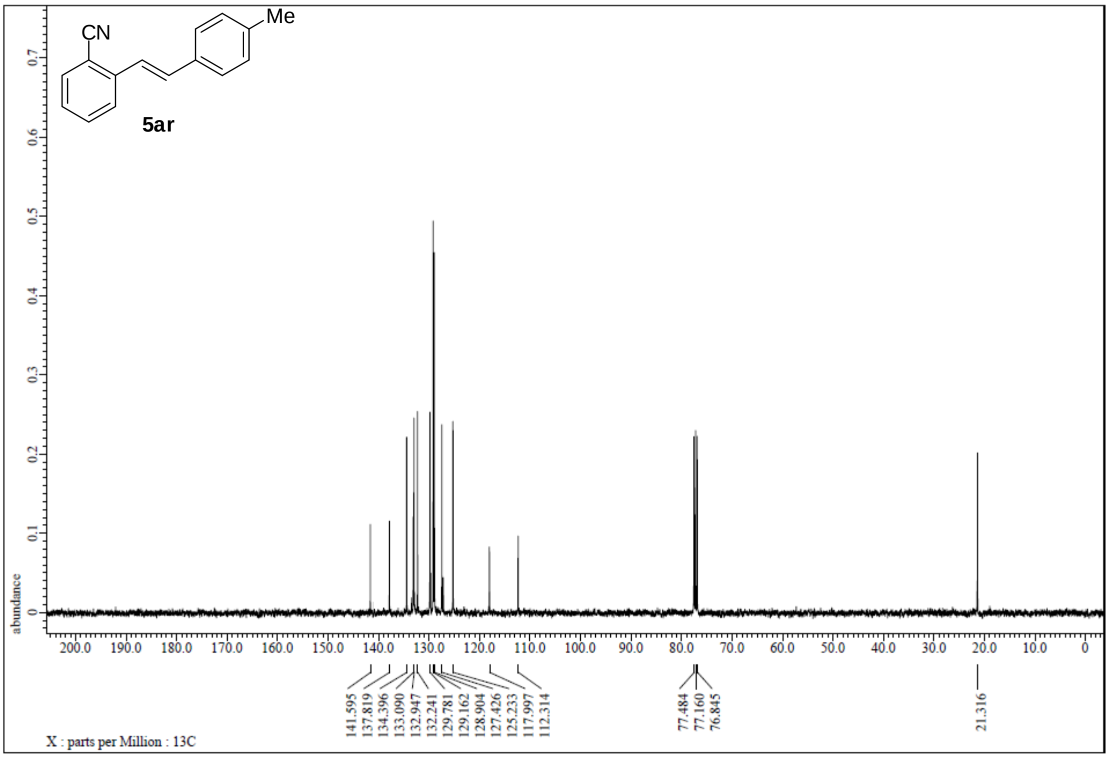

Figure S200. **^{13}C NMR spectrum of 5ar, related to Figure 5**.

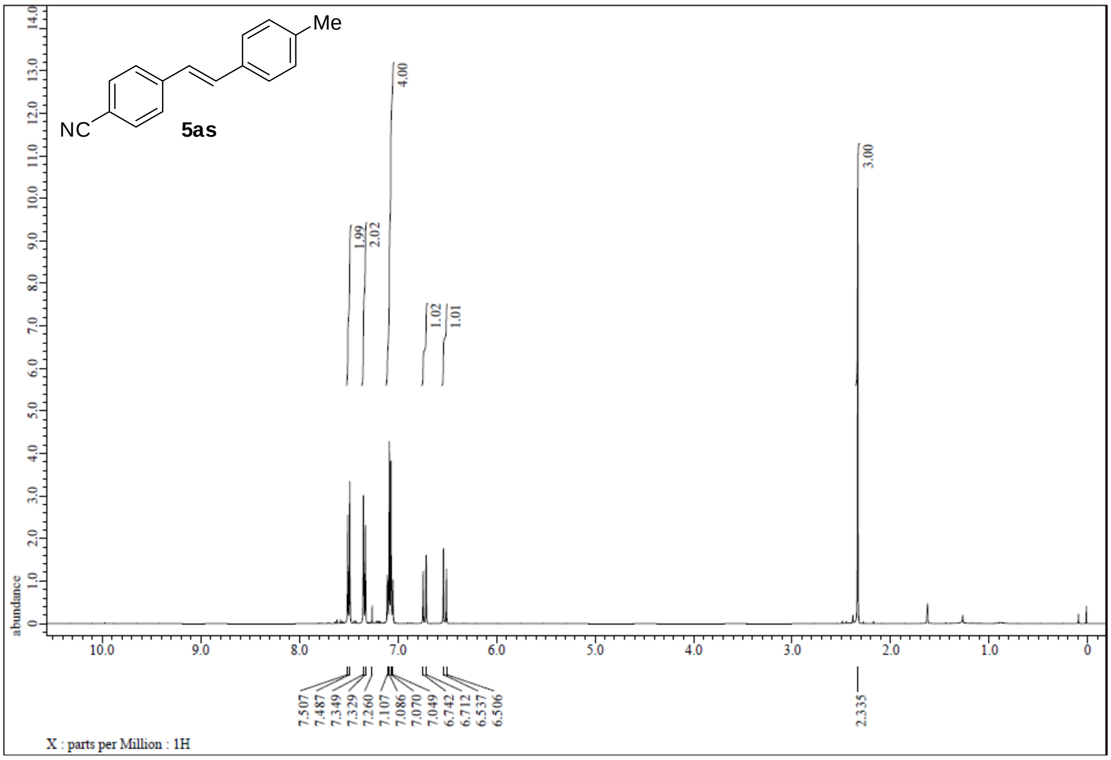

Figure S201. [1]**H NMR spectrum of 5as, related to Figure 5**.

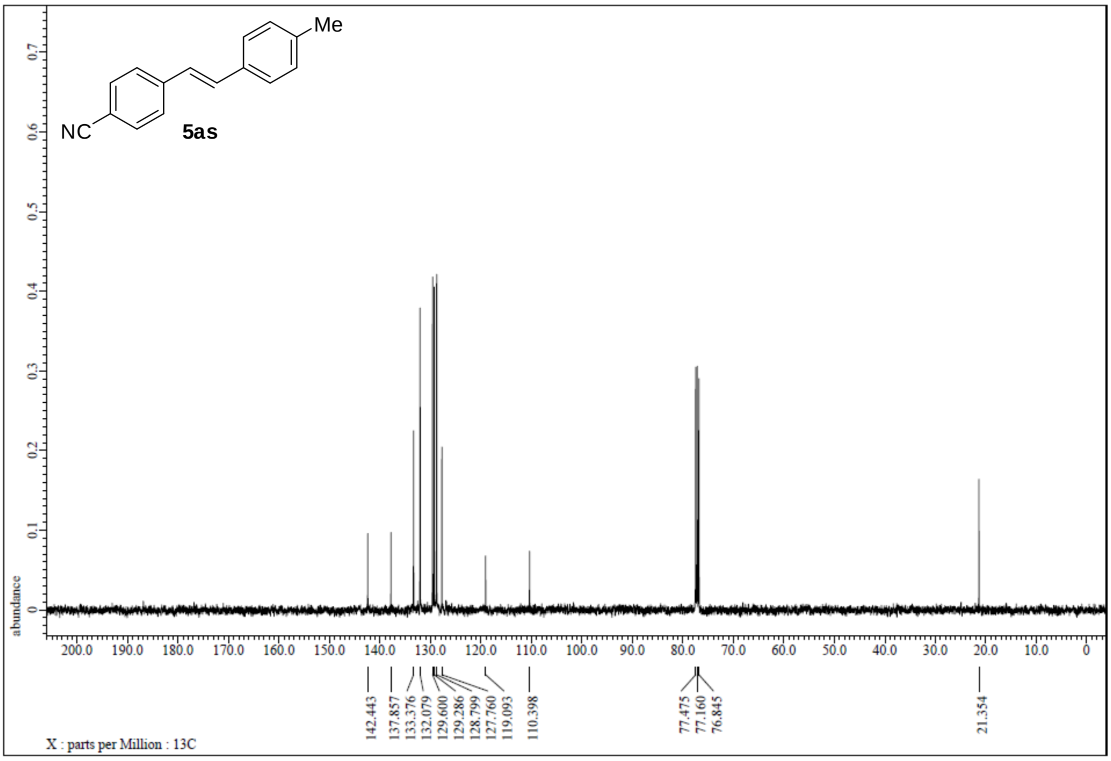

Figure S202. [13]**C NMR spectrum of 5as, related to Figure 5**.

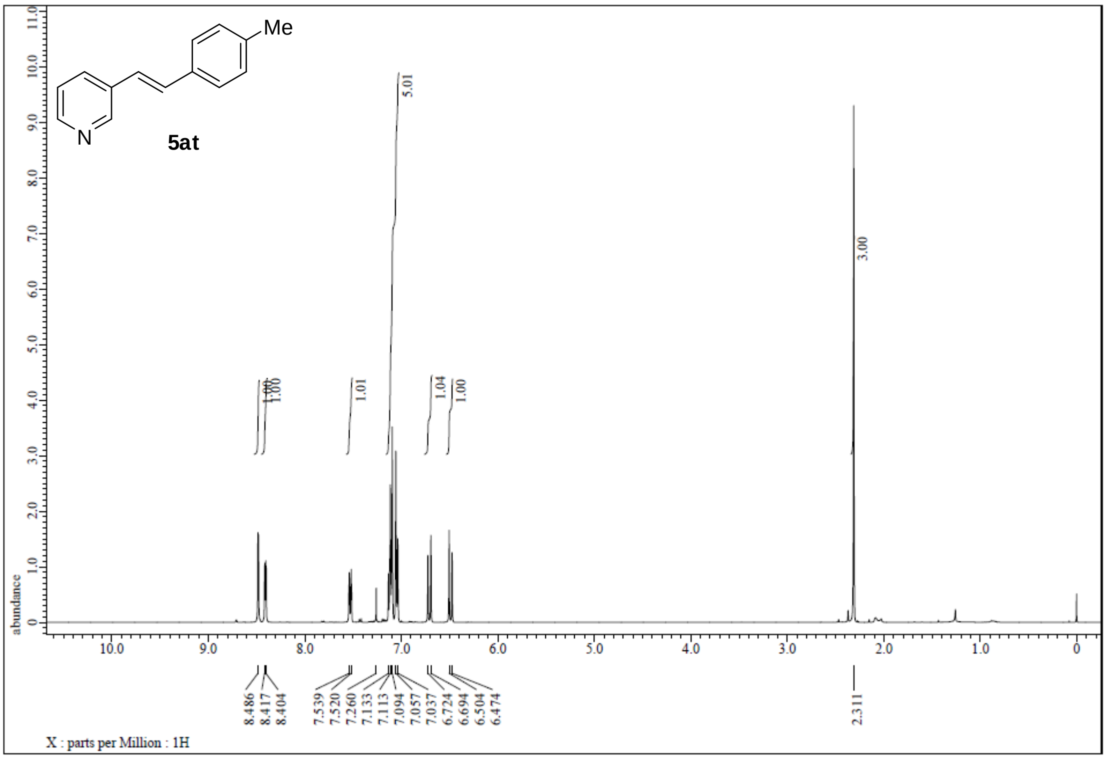

Figure S203. ^{1}**H NMR spectrum of 5at, related to Figure 5**.

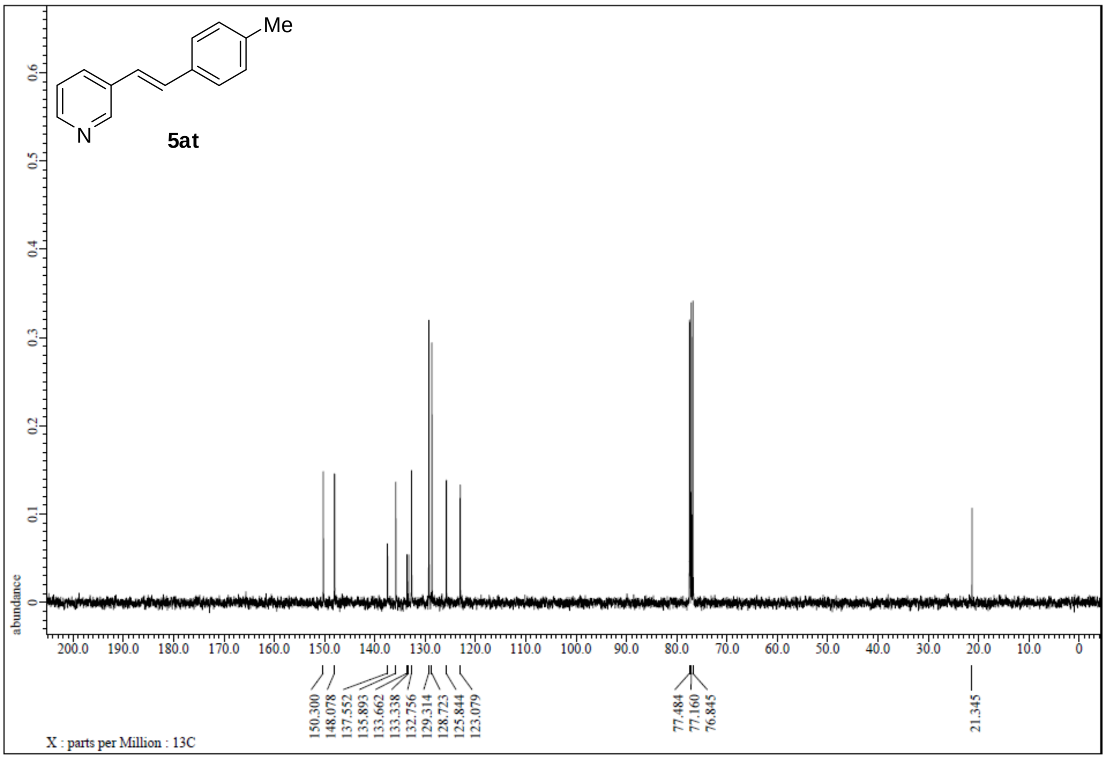

Figure S204. ^{13}**C NMR spectrum of 5at, related to Figure 5**.

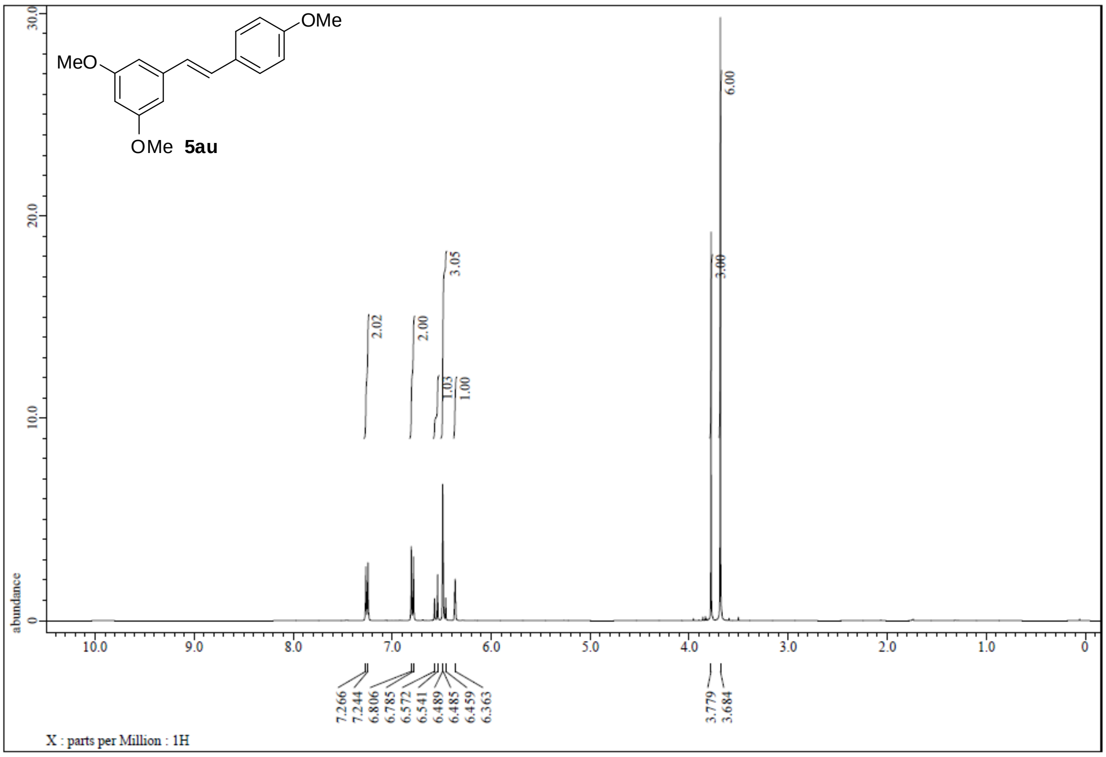

Figure S205. ^{1}H NMR spectrum of 5au, related to Figure 5.

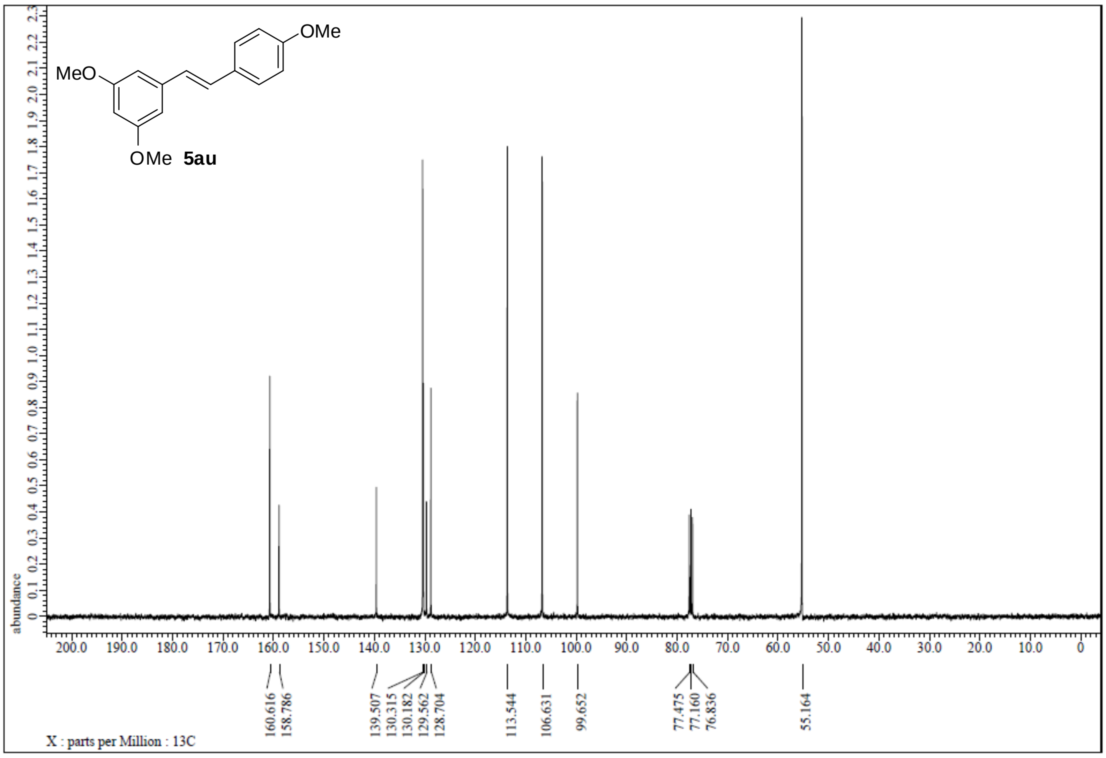

Figure S206. ^{13}C NMR spectrum of 5au, related to Figure 5.

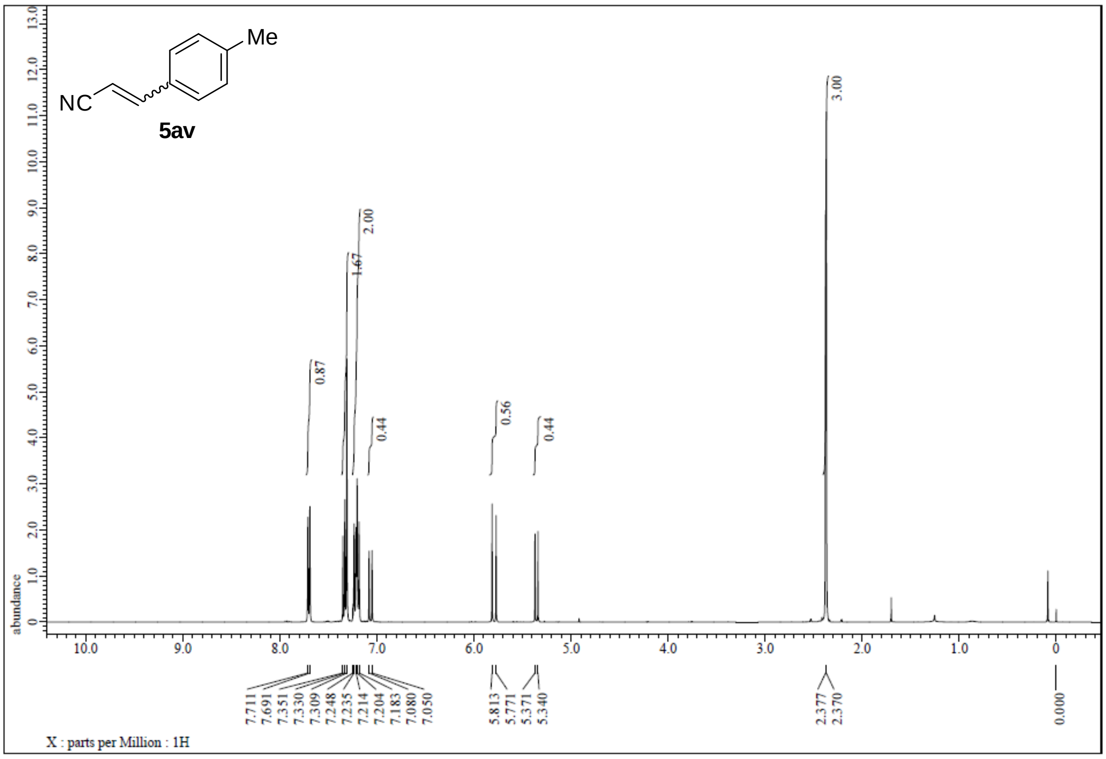

Figure S207. ^{1}**H NMR spectrum of 5av, related to Figure 5**.

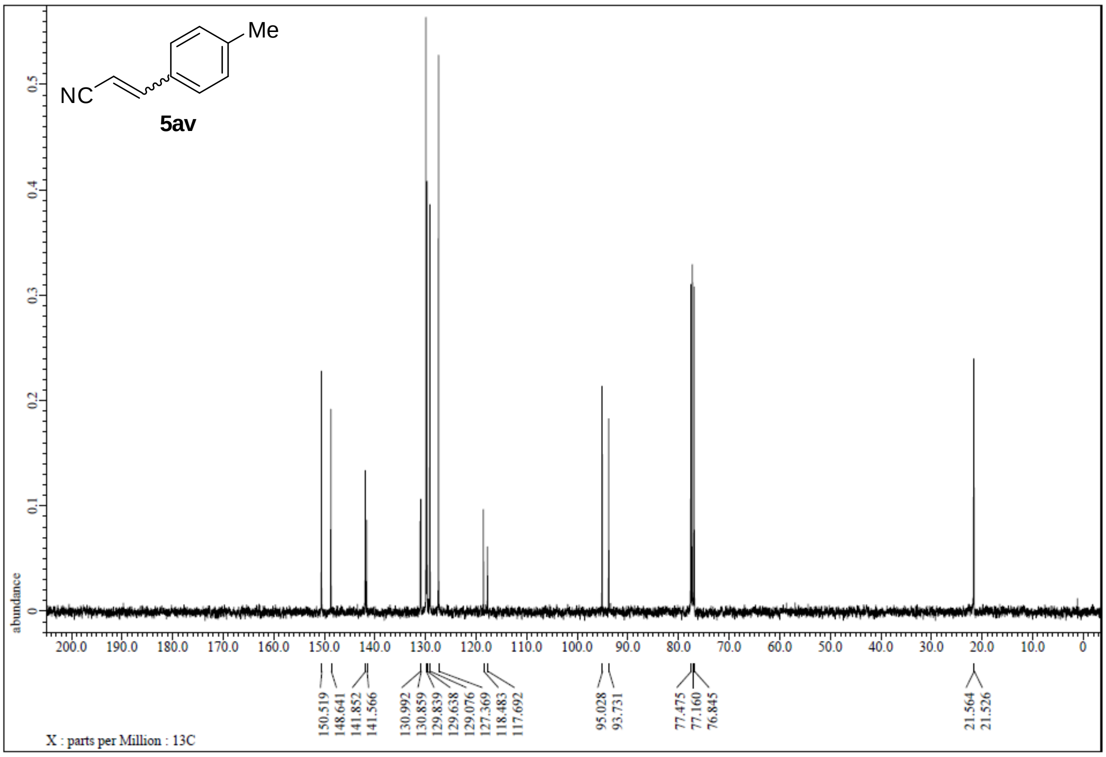

Figure S208. ^{13}**C NMR spectrum of 5av, related to Figure 5**.

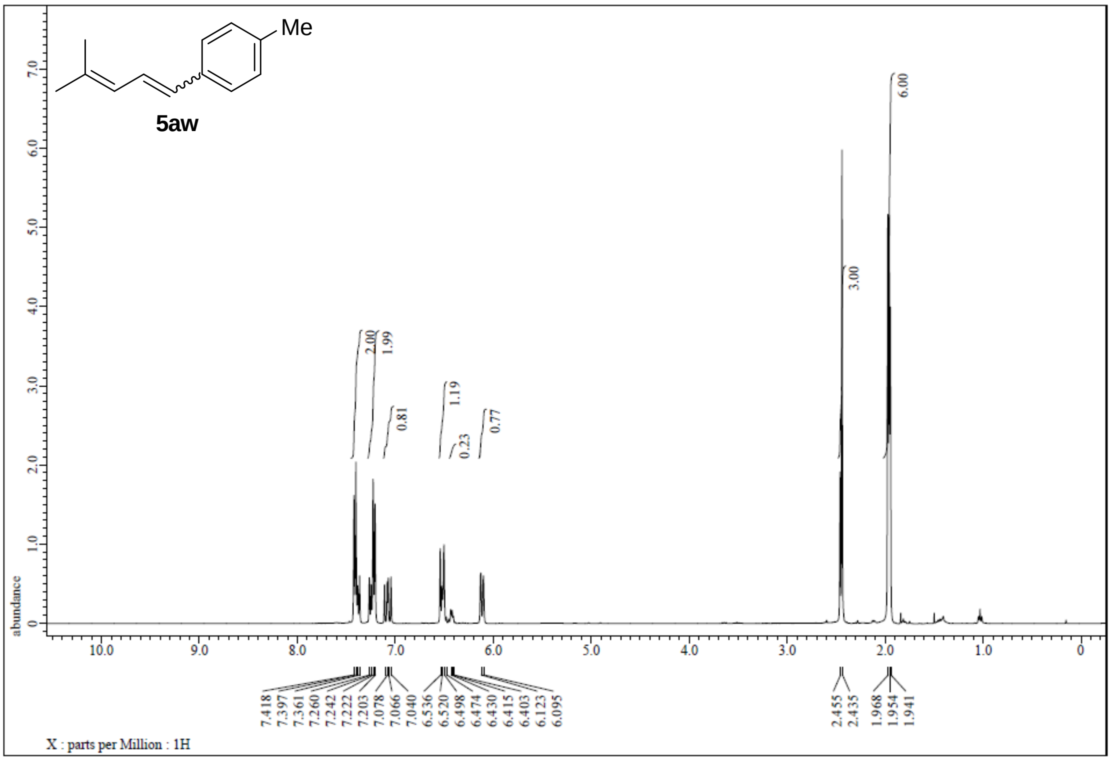

Figure S209. ^{1}H NMR spectrum of 5aw, related to Figure 5.

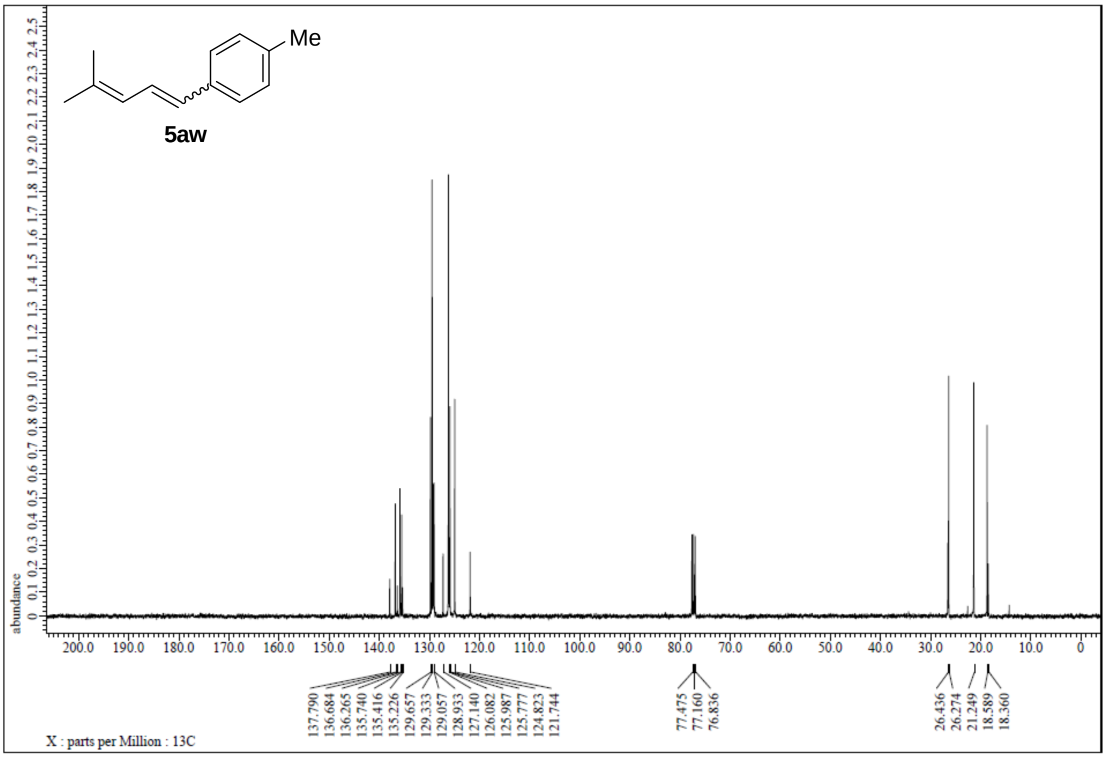

Figure S210. ^{13}C NMR spectrum of 5aw, related to Figure 5.

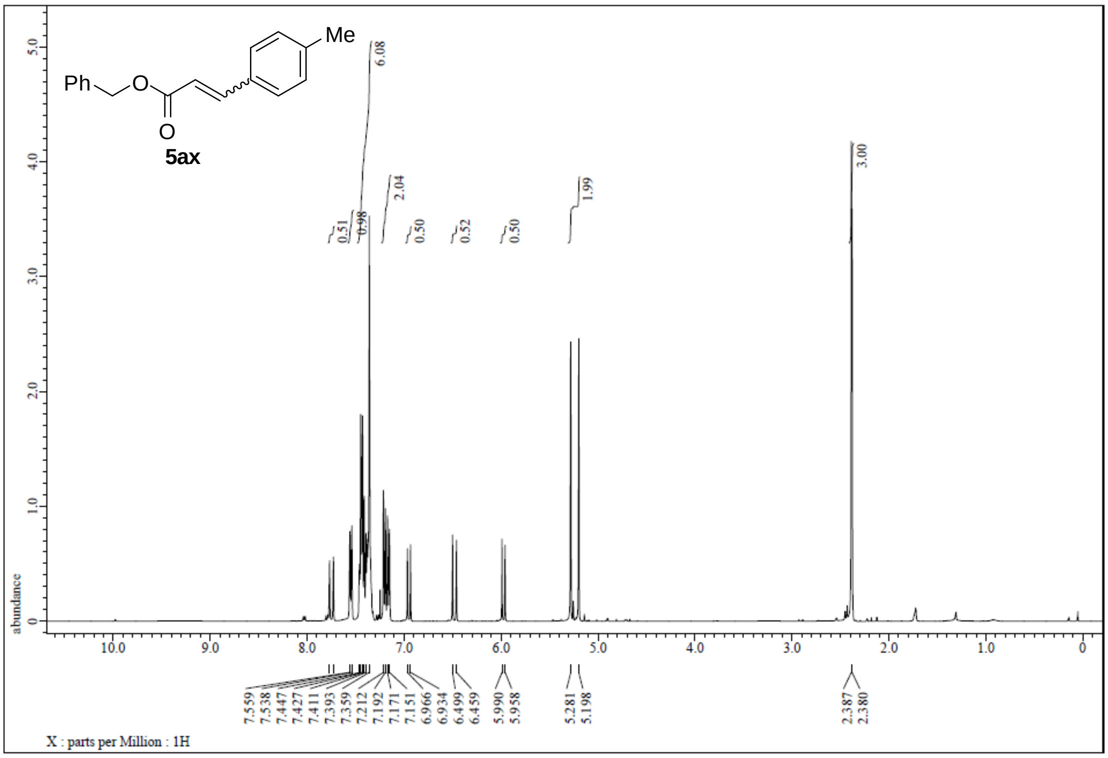

Figure S211. ¹H NMR spectrum of 5ax, related to Figure 5.

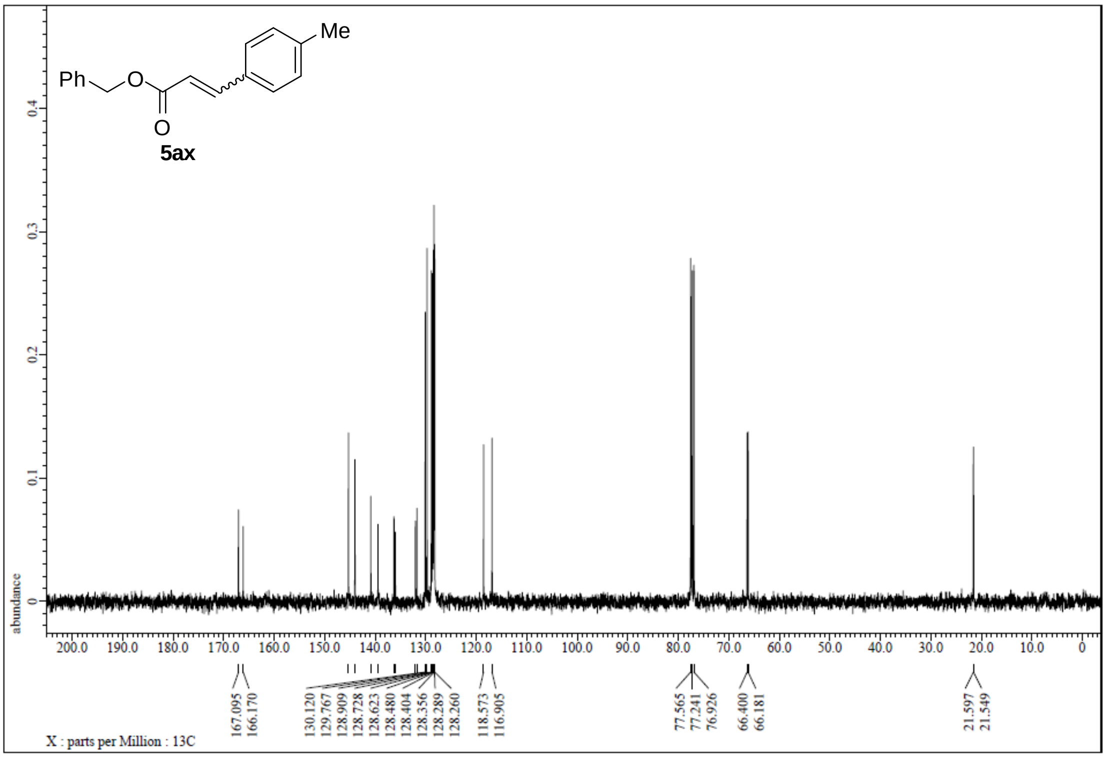

Figure S212. ¹³C NMR spectrum of 5ax, related to Figure 5.

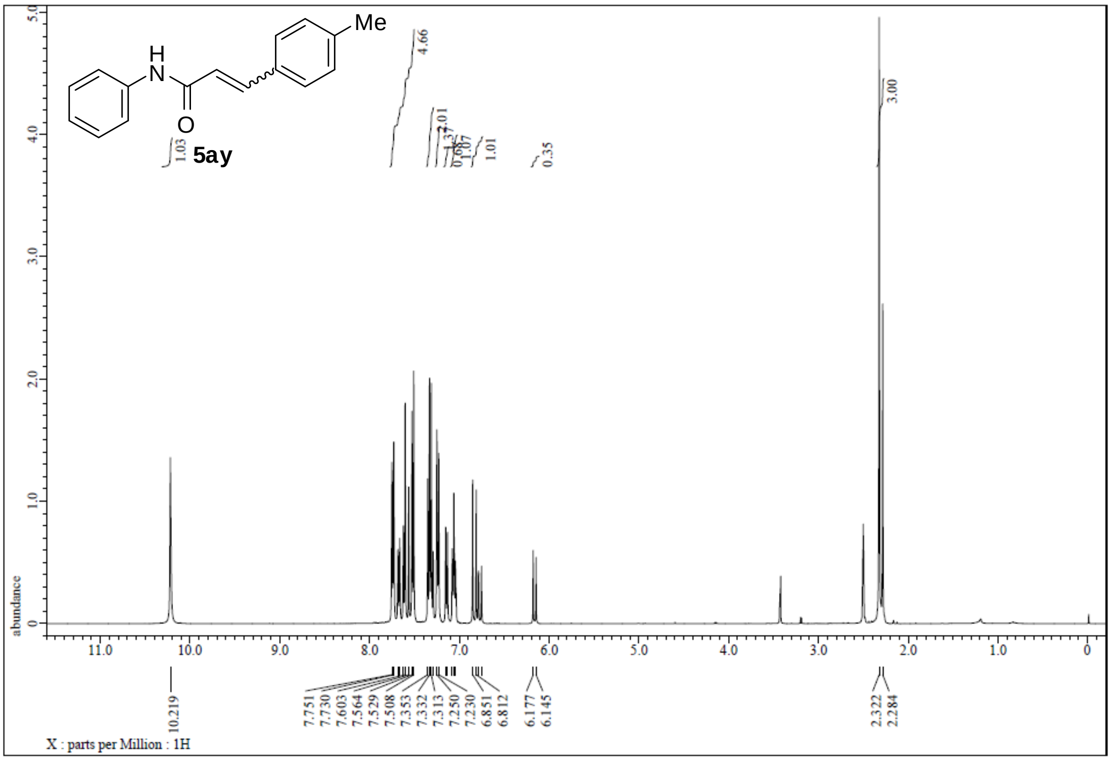

Figure S213. ^{1}H NMR spectrum of 5ay, related to Figure 5.

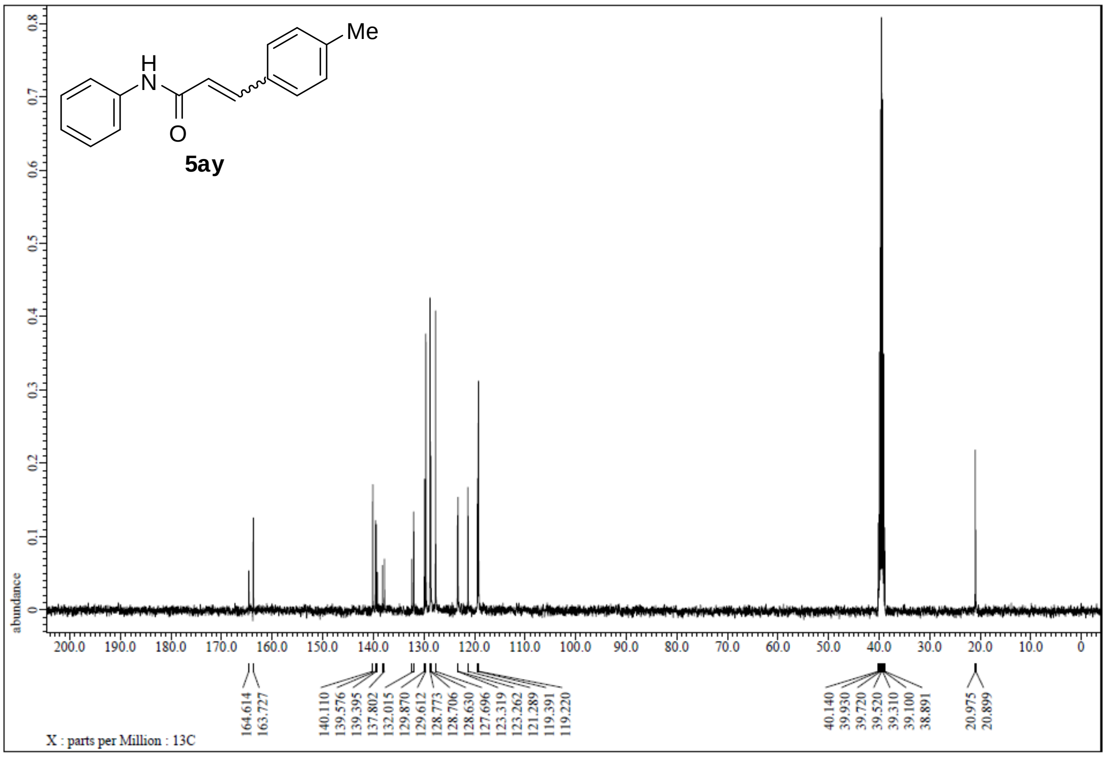

Figure S214. ^{13}C NMR spectrum of 5ay, related to Figure 5.

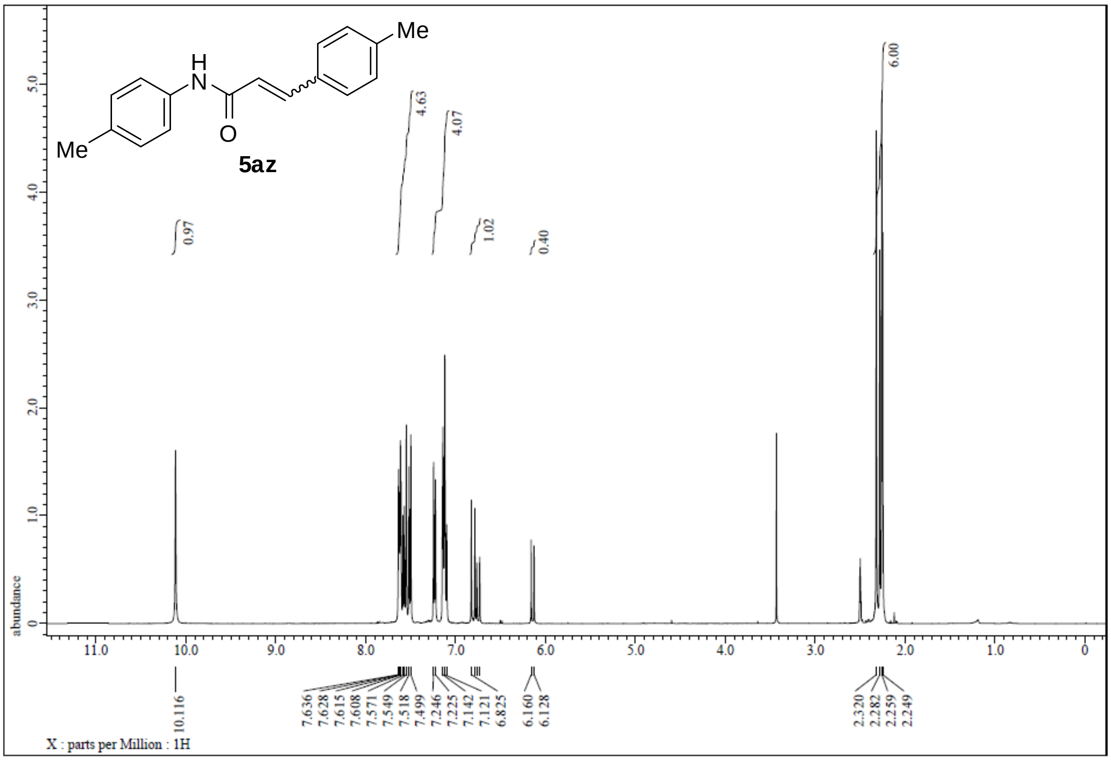

Figure S215. **^{1}H NMR spectrum of 5az, related to Figure 5**.

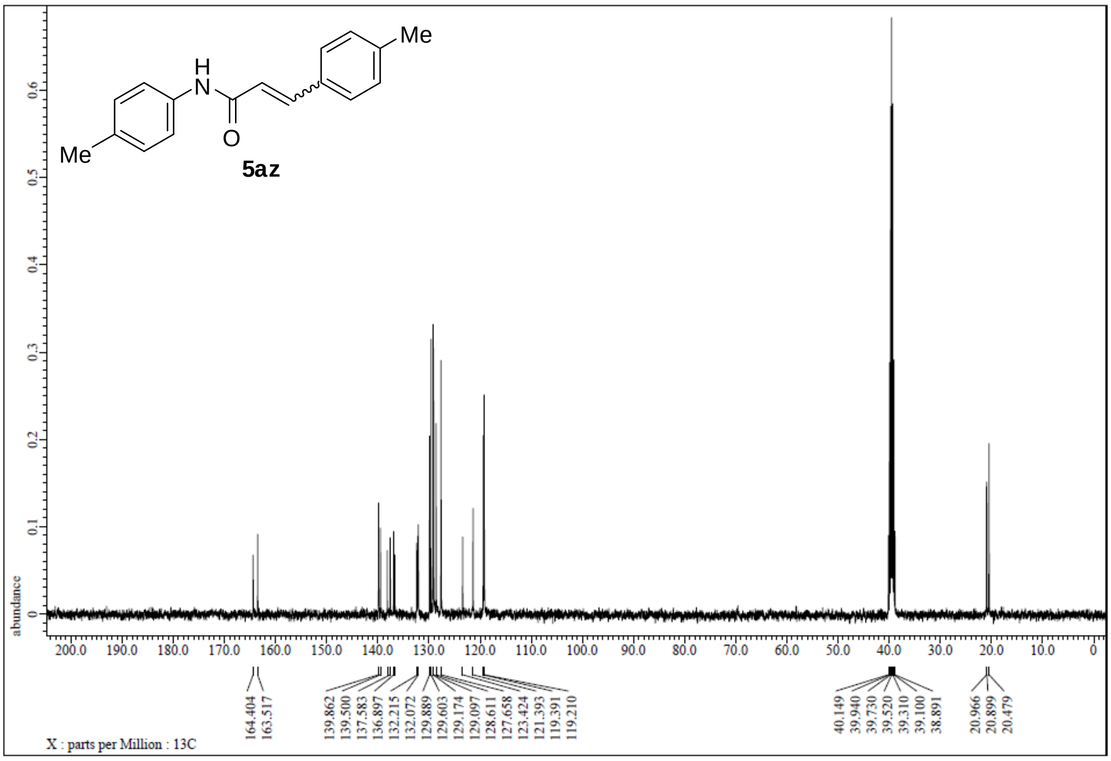

Figure S216. **^{13}C NMR spectrum of 5az, related to Figure 5**.

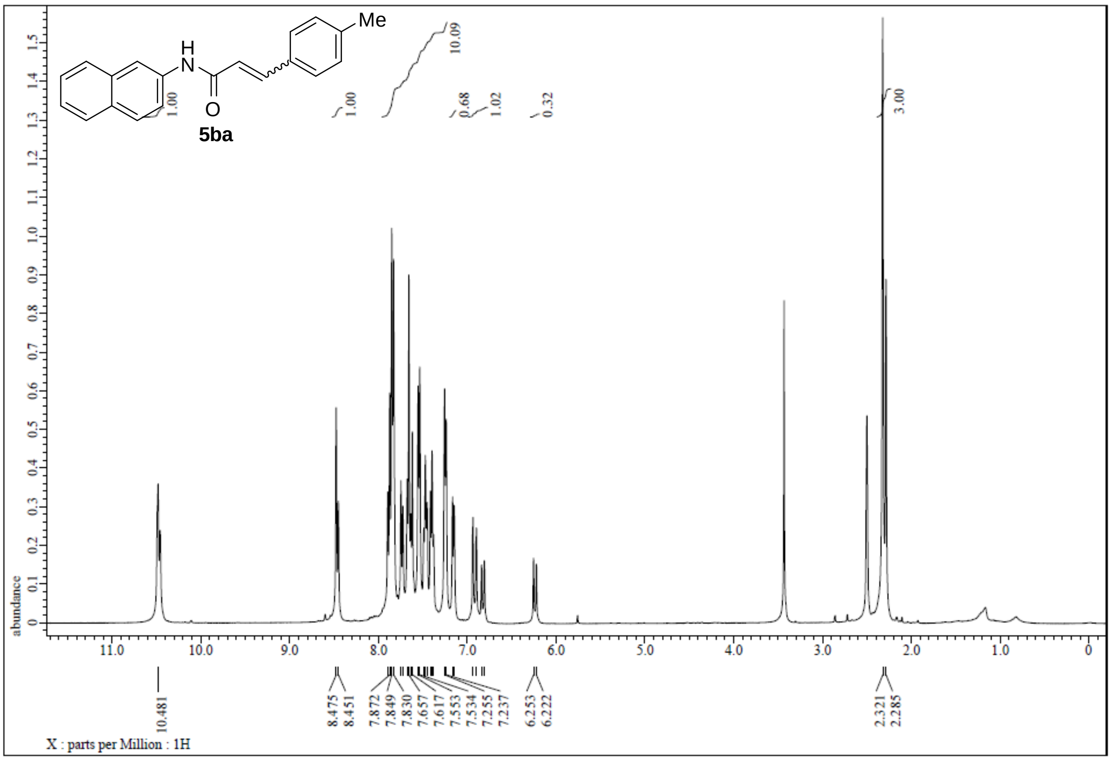

Figure S217. ^{1}H NMR spectrum of 5ba, related to Figure 5.

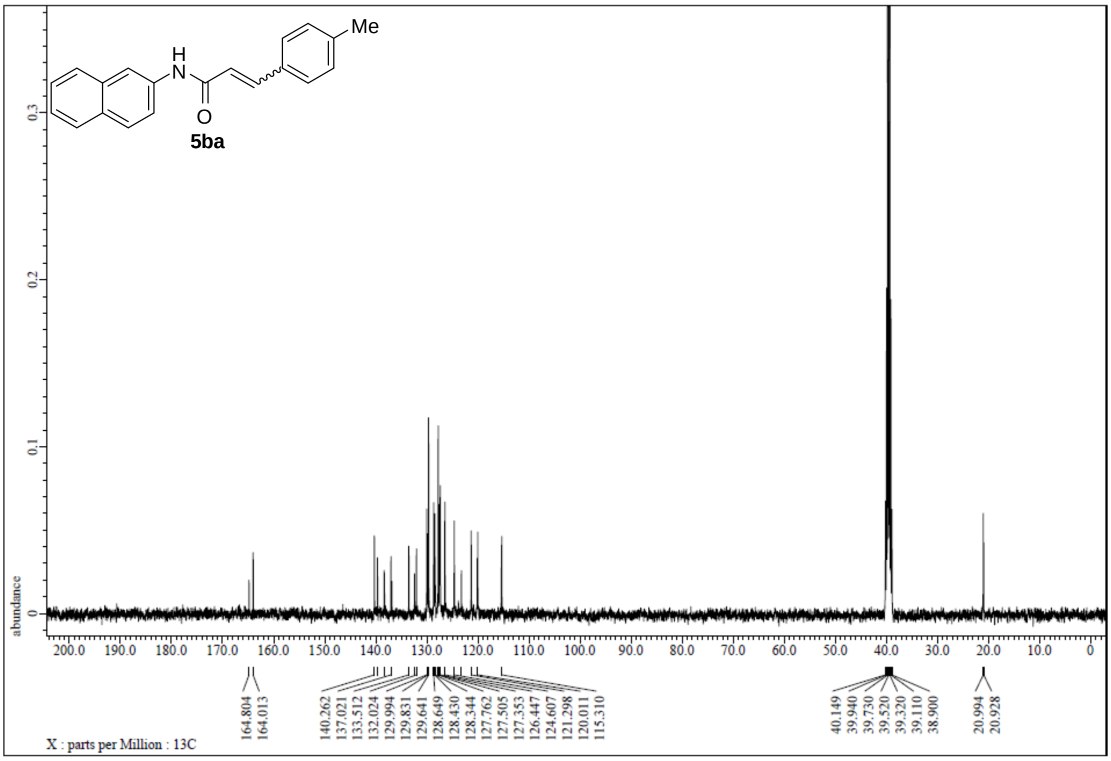

Figure S218. ^{13}C NMR spectrum of 5ba, related to Figure 5.

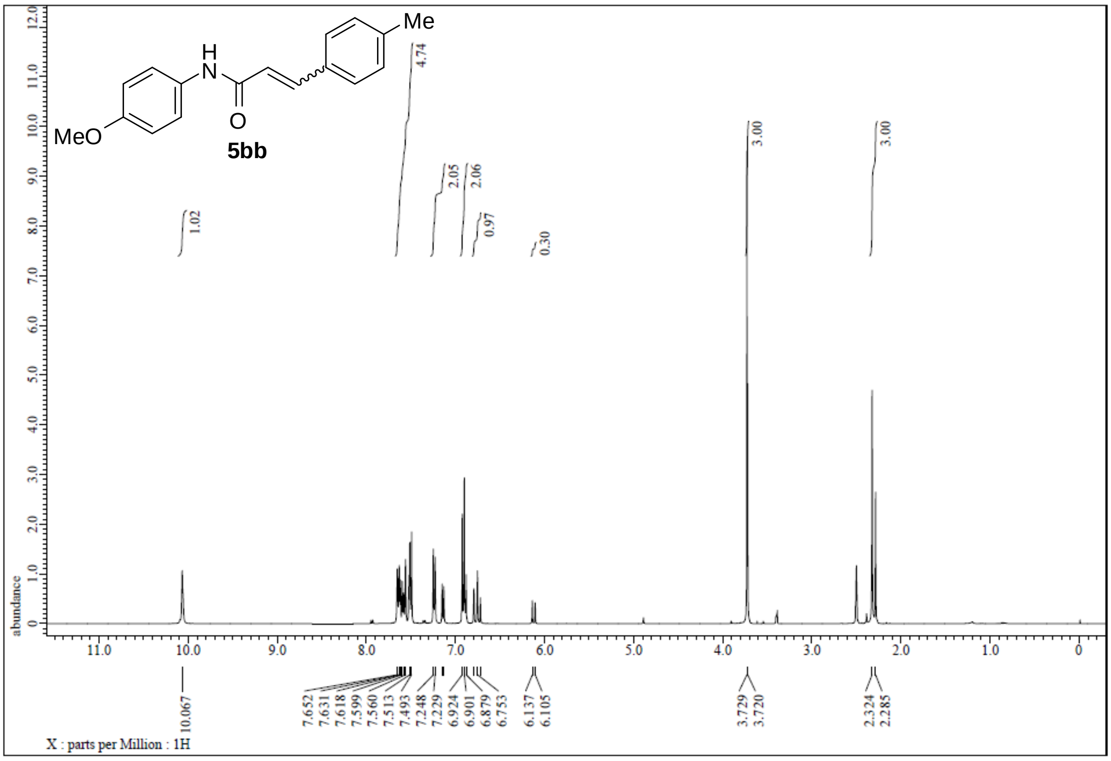

Figure S219. ^{1}**H NMR spectrum of 5bb, related to Figure 5**.

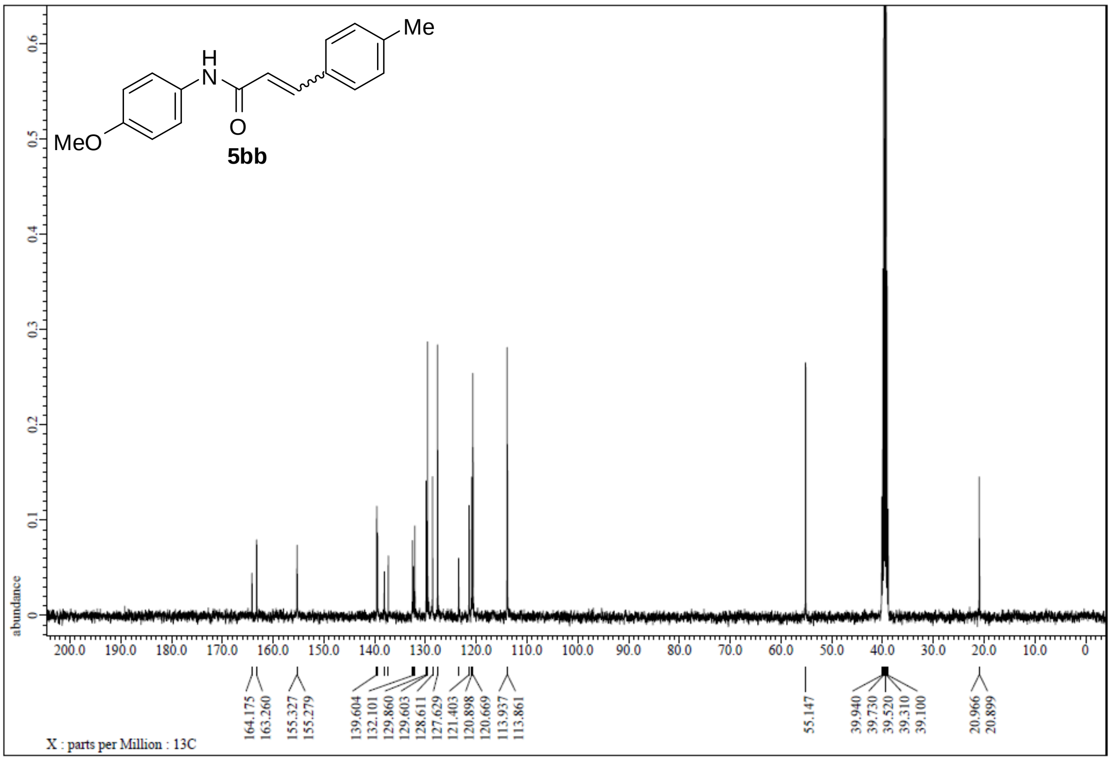

Figure S220. ^{13}**C NMR spectrum of 5bb, related to Figure 5**.

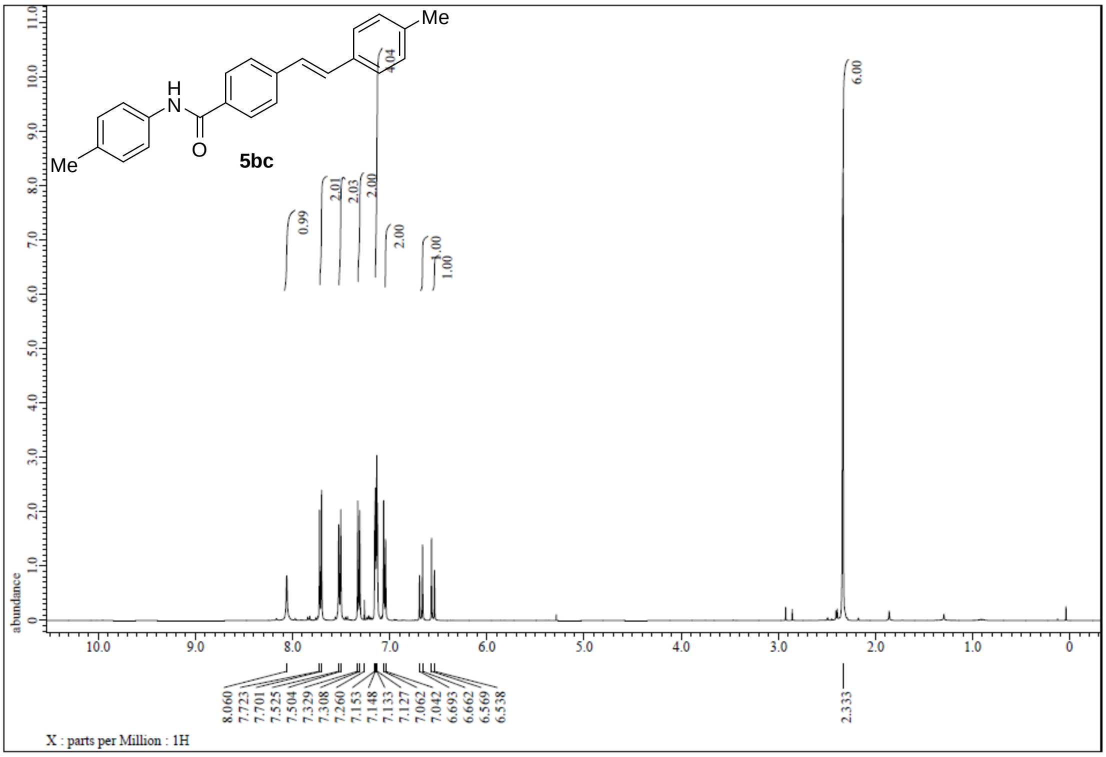

Figure S221. ^{1}H NMR spectrum of 5bc, related to Figure 5.

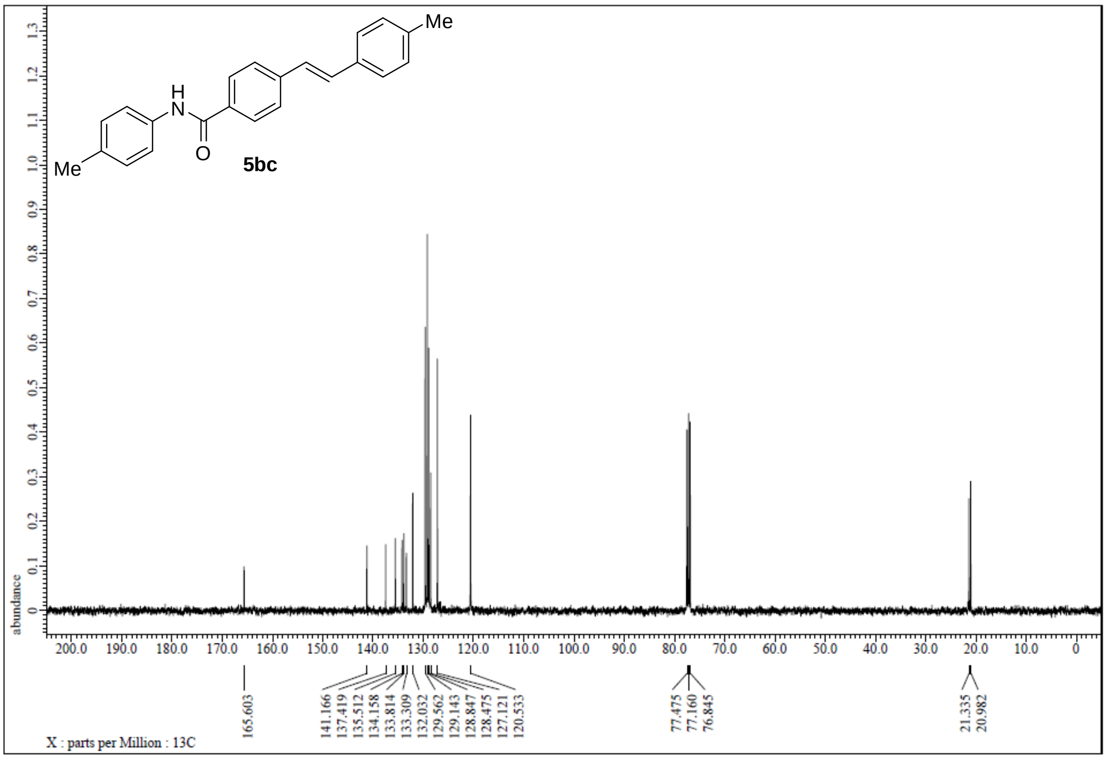

Figure S222. ^{13}C NMR spectrum of 5bc, related to Figure 5.

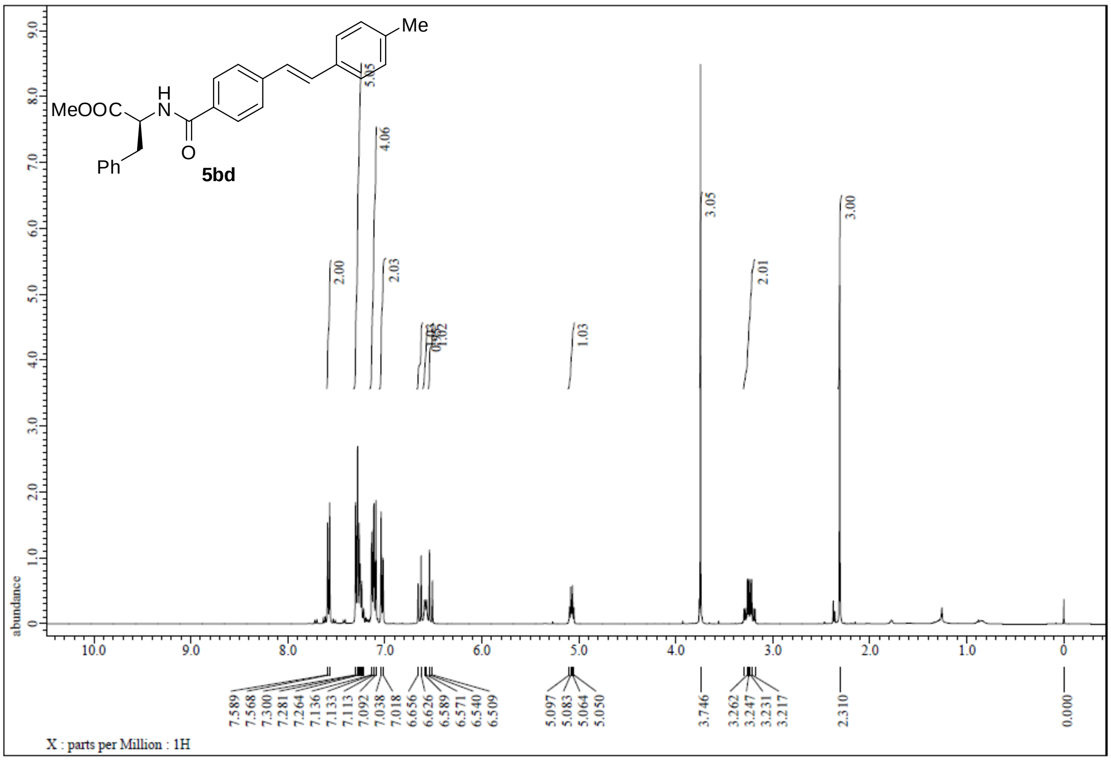

Figure S223. ^{1}H NMR spectrum of 5bd, related to Figure 5.

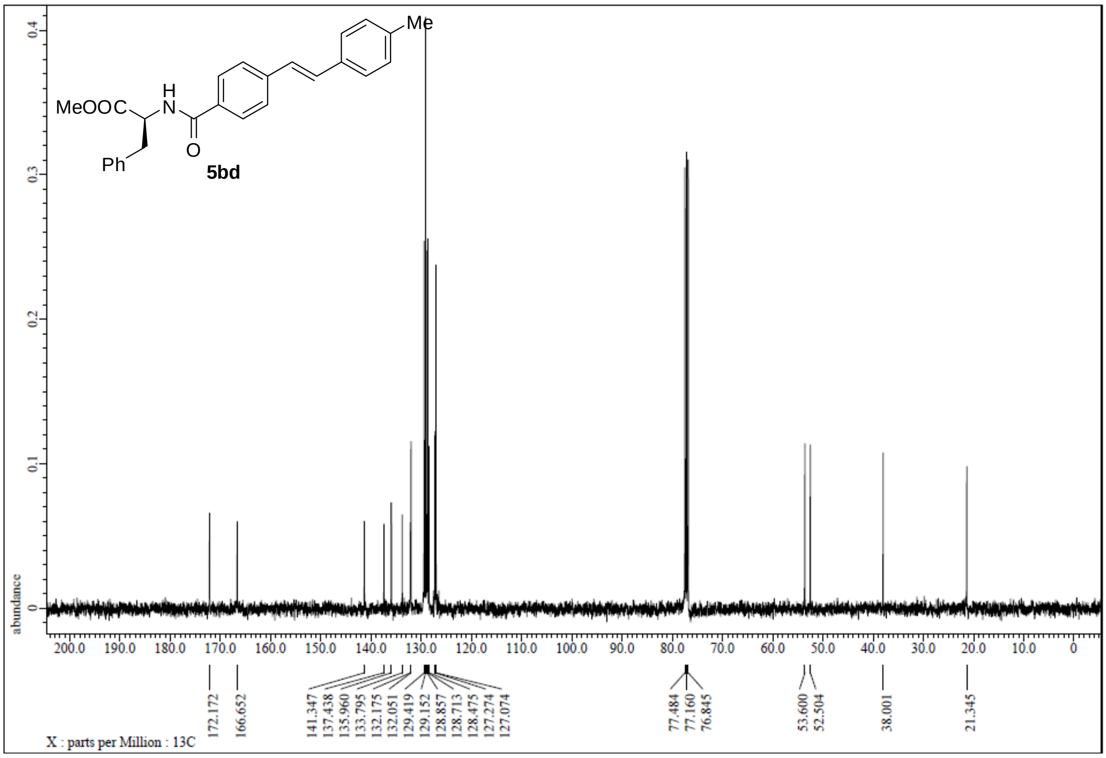

Figure S224. ^{13}C NMR spectrum of 5bd, related to Figure 5.

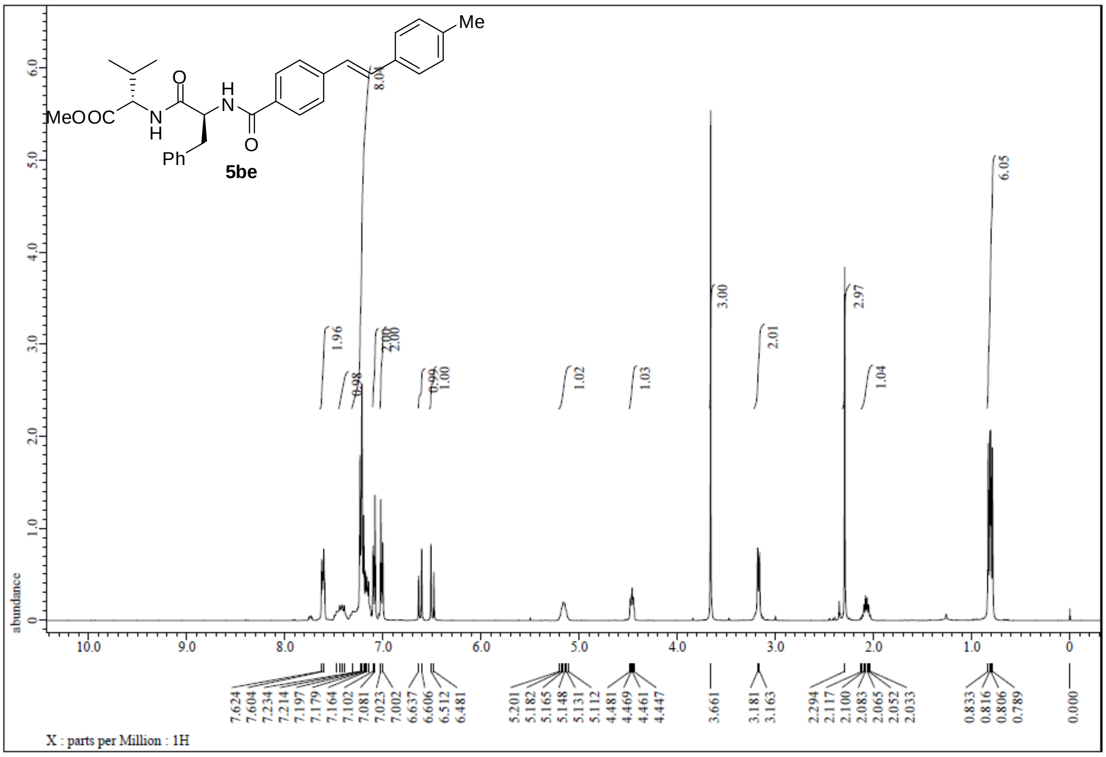

Figure S225. ^{1}H NMR spectrum of 5be, related to Figure 5.

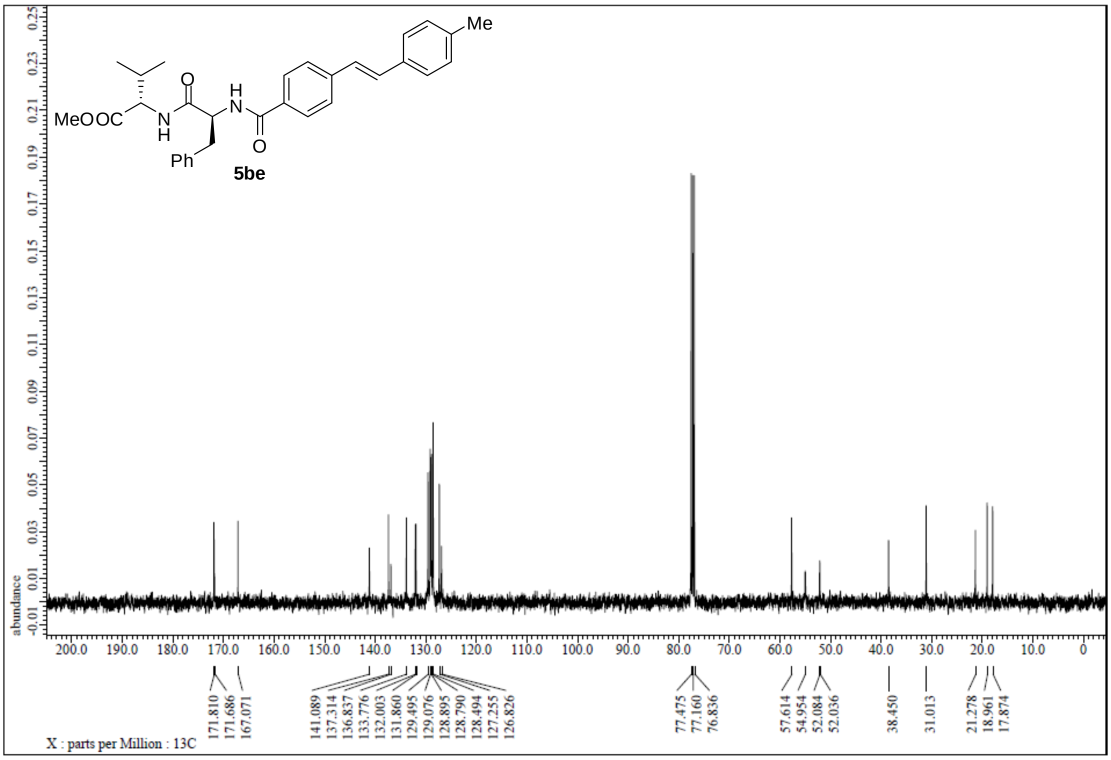

Figure S226. ^{13}C NMR spectrum of 5be, related to Figure 5.

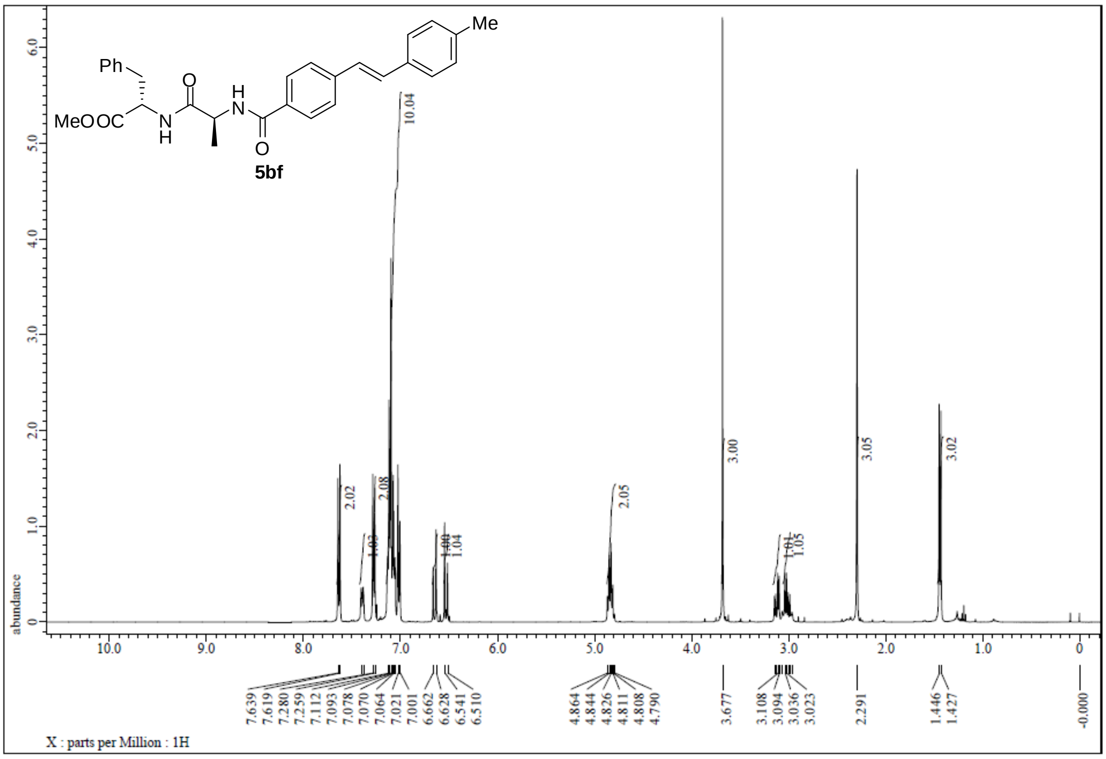

Figure S227. ^{1}H NMR spectrum of 5bf, related to Figure 5.

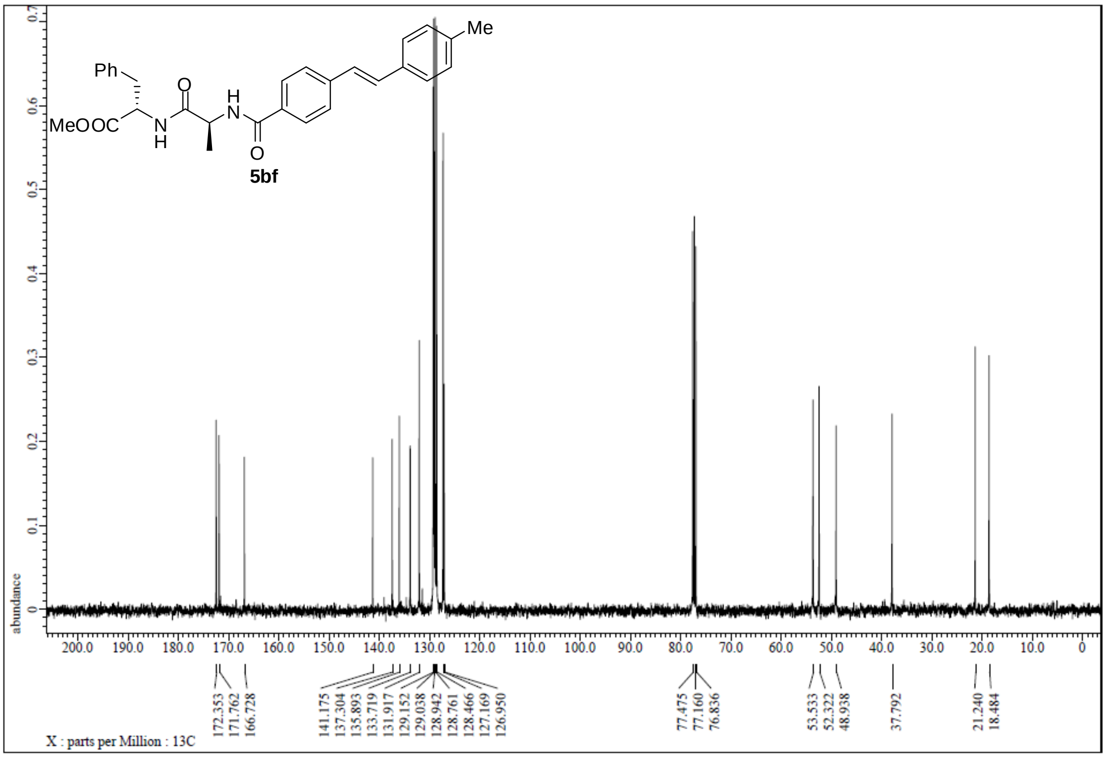

Figure S228. ^{13}C NMR spectrum of 5bf, related to Figure 5.

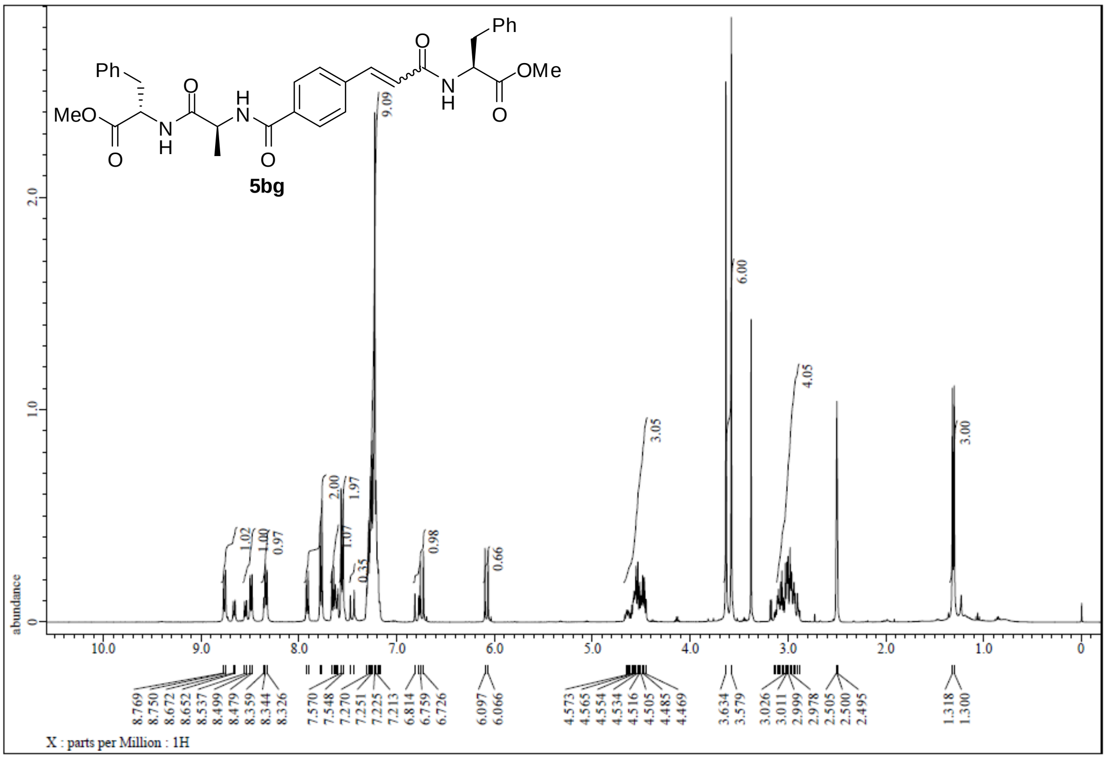

Figure S229. ¹H NMR spectrum of 5bf, related to Figure 5.

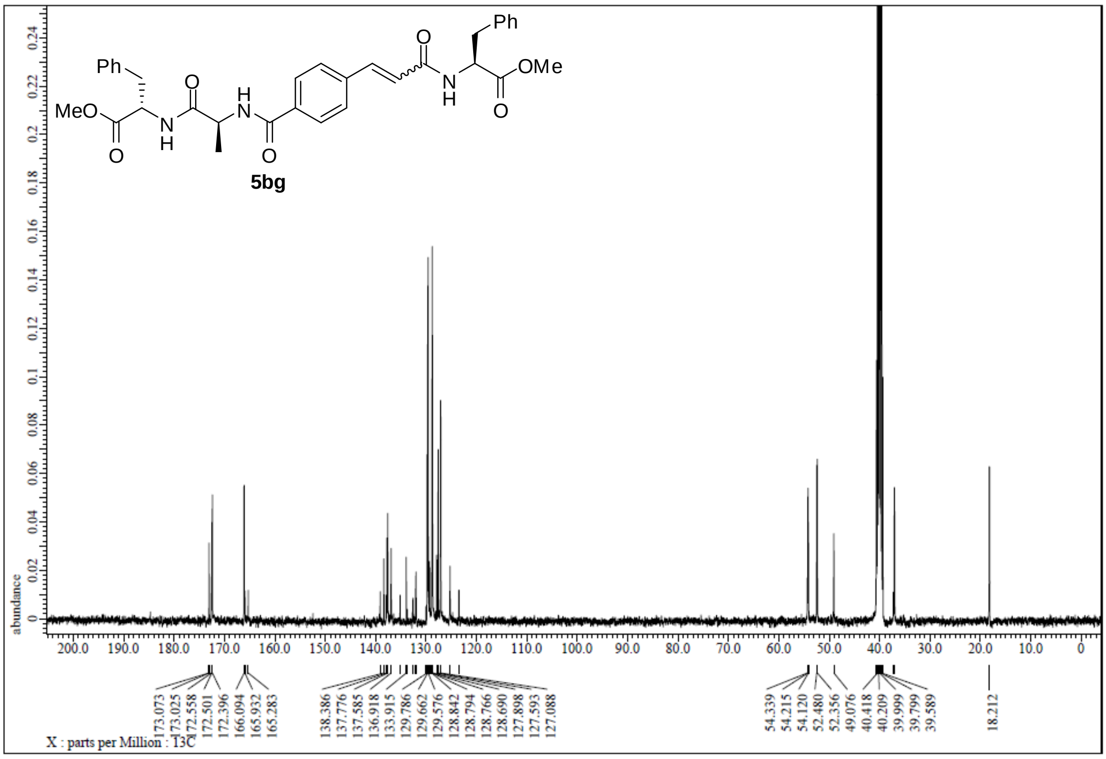

Figure S230. ¹³C NMR spectrum of 5bg, related to Figure 5.

Transparent Methods

1. General procedures

All reactions were carried out in dry solvents under argon atmosphere. Reagents were purchased and used without further purification. Reactions were monitored by thin layer chromatography (TLC), and the products were obtained by column chromatography on silica gel or preparative thin layer chromatography (pTLC). High resolution mass spectra (HRMS) were recorded on a Shimadzu LCMS-IT/TOF quadrupole-time of flight mass spectrometer. Electron-impact mass spectra were recorded on a JEOL JMS-Q1050GC Master Quad GC/MS. NMR spectra were recorded on JOEL JNM-ECA 600, JNM-ECS 400 and JNM-ECA 300 for proton and carbon magnetic resonance spectra (^{1}H NMR and ^{13}C NMR). ^{1}H NMR chemical shifts were referenced to the hydrogen signal of tetramethylsilane (TMS) (δ = 0.00 ppm) or the residual hydrogen signal of deuterated chloroform (δ = 7.26 ppm). In ^{13}C measurements the signal of $CDCl_3$ (δ = 77.0) was used as a reference. The following abbreviations were used to explain the multiplicities: s = singlet, d = doublet, t = triplet, q = quartet, m = multiplet, b = broad.

2. Synthesis of photocatalysts and substrates 1f, 1g and 2g

(1) Synthesis of photocatalysts

$Ru(bpy)_3Cl_2·6H_2O$ was purchased from commercial Energy-Chemical Co. and was used without further treatment.

Synthesis of [*fac*-Ir(ppy)$_3$]: [*fac*-Ir(ppy)$_3$] was synthesized according to the previous report (Tamayo et al., 2003; Sprouse et al., 1984). Iridium trichloride hydrate (0.388 g) and 2-phenylpyridine (0.76 g) were dissolved in a mixed solvent of 2-ethoxyethanol (30 mL) and water (10 mL), and the solution was refluxed for 24 h. The resulting solution was cooled to room temperature to form yellow precipitate, and the yellow precipitate was collected on a glass filter frit and was washed with 95% ethanol (60 mL) and acetone (60 mL). The solid was dissolved in dichloromethane (75 mL), and the solution was filtered. Toluene (25 mL) and hexane (10 mL) were added to the filtrate, which was then reduced in volume by evaporation to 50 mL, and cooled to give $[Ir(ppy)_2Cl]_2$ [*tetrakis*(2-phenylpyridine-C2,N')(μ-dichloro)-diiridium] as crystals (0.428 g, 72%).

A mixture of [Ir(ppy)$_2$Cl]$_2$, 2-phenylpyridine (2.5 equiv, 0.155 g) and K_2CO_3 (10 equiv, 0.544 g) was heated to ~200 ℃ under inert atmosphere in 20 mL of glycerol for 20-24 h. After the mixture was cooled to room temperature, 20 mL of deionized H_2O was added, and the resulting precipitate was filtered off, washed with two portions of methanol, followed by ether and hexane. The crude product was then flash chromatographed on a silica column using dichloromethane as the eluent to provide pure [*fac*-Ir(ppy)$_3$] (0.17 g, 65%).

Synthesis of [Ir(ppy)$_2$dtbbpy]PF$_6$: [Ir(ppy)$_2$dtbbpy]PF$_6$ was prepared according to the previous report (Slinker et al., 2004). A mixture of [Ir(ppy)$_2$Cl]$_2$ (214 mg, 0.2 mmol) and 4,4'-di-*tert*-butyl-2,2'-dipyridyl (118 mg, 0.44 mmol) in 10 mL of 1,2-ethanediol under nitrogen atmosphere was heated at 150℃ for 15 h. The solid was dissolved to yield a clear, yellow solution. After cooling the resulting solution to room temperature, 150 mL of water was added. Excess of bipyridine was removed through extraction with diethyl ether (3×50 mL), and the aqueous layer was subsequently heated to 60-70 ℃. NH_4PF_6 (1.0 g) in 10 mL of water was added, and the PF_6 salt of the chromophore immediately precipitated. After cooling the suspension to 5 ℃, the yellow solid was separated through filtration, dried, and recrystallized through acetonitrile/ether diffusion. Yield: 273 mg (75%).

(2) Synthesis of peptides 1f, 1g and 2g

(A) Synthesis of 1f

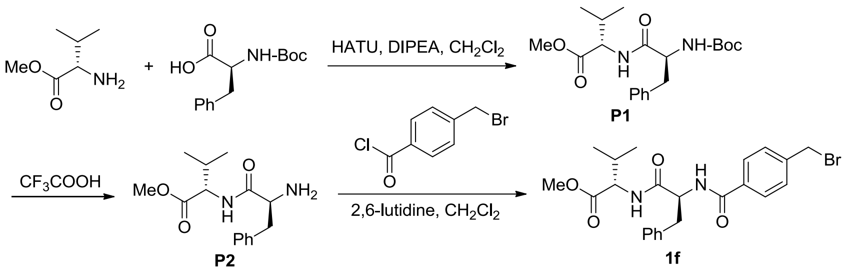

To a 50 mL rounded bottom bottle (*S*)-methyl 2-amino-3-methylbutanoate hydrochloride (1.252 g, 7.5 mmol), (*S*)-2-(*tert*-butoxycarbonylamino)-3-phenylpropanoic acid (1.325 g, 5 mmol) and HATU (2.852 g, 7.5 mmol) were added, followed with dry CH_2Cl_2 (25 mL), the mixture was cooled to 0 ℃, then diisopropylethylamine (2.68 mL, 15 mmol) was added, the mixture was allowed to warm to room temperature and stirred for 5 h. After dilution with CH_2Cl_2 (25 mL), the mixture was washed with 1N HCl (2 × 50 mL), saturated $NaHCO_3$ (2 × 50 mL). The combined aqueous layer was extracted with CH_2Cl_2 (2 × 40 mL). The organic layers were combined and

washed with brine (50mL), dried over Na_2SO_4, filtered and concentrated. The residue was purified by column chromatography to afford product **P1** (1.882 g, 92%). To a 50 mL rounded bottom bottle with **P1** (1.64 g, 4 mmol) in CH_2Cl_2 (30 mL), CF_3COOH (0.75 mL, 10 mmol) was added, the reaction was monitored by TLC. After the reaction completed, the solvent was removed under reduced pressure, and the crude product was used without further purification. 2,6-Lutidine (1.4 mL, 12 mmol) was added to the crude product above in CH_2Cl_2 (20 mL). After the mixture was cooled to 0 ^oC, a fresh synthesized 4-(bromomethyl)benzoyl chloride (5 mmol) in CH_2Cl_2 (10 mL) was added dropwise to the mixture, then the solution was warmed to room temperature and stirred for 4 h. After the reaction completed, the solution was poured into 50 mL of 1 N HCl, another 20 mL of CH_2Cl_2 was added to the mixture, then washed with 1N HCl (2 × 50 mL), the combined aqueous layer was extracted with CH_2Cl_2 (2 × 20 mL). The combined organic layer was washed with brine, dried with Na_2SO_4, filtered and evaporated under reduced pressure, and the residue was purified by silica gel column chromatography to afford desired product (*S*)-methyl 2-((*S*)-2-(4-(bromomethyl)benzamido)-3-phenylpropanamido)-3-methylbutanoate (**1f**), 1.28 g (67% yield). ^1H NMR ($CDCl_3$, 400 MHz) 7.72 (d, *J* = 8.25 Hz, 2H), 7.38 (d, *J* = 8.25 Hz, 2H), 7.23-7.20 (m, 6H), 6.92 (d, *J* = 8.59 Hz, 1H), 5.03 (q, *J* = 7.22 Hz, 1H), 4.57 (s, 2H), 4.46-4.44 (m, 1H), 3.69 (s, 3H), 3.21-3.14 (m, 2H), 2.13-2.05 (m, 1H), 0.83 (q, J = 8.22 Hz, 6H). ^{13}C NMR ($CDCl_3$, 100 MHz) 171.8, 171.4, 166.8, 141.1, 136.7, 133.7, 129.5, 128.74, 128.67, 127.7, 127.0, 57.6, 55.0, 52.2, 45.5, 38.3, 31.1, 19.0, 17.9. ESI-MS: $(M+H)^+$ m/z 475

(*S*)-Methyl 2-((*S*)-2-(4-(bromomethyl)benzamido)propanamido)-3-phenylpropanoate (**1g**) was synthesized through the same procedures described above. Yield: 69%. ^1H NMR ($CDCl_3$, 400 MHz) 7.75 (d, *J* = 8.25 Hz, 2H), 7.43 (d, *J* = 8.25 Hz, 3H), 7.16-7.13 (m, 3H), 7.06 (d, *J* = 7.56 Hz, 2H), 6.98 (d, J = 7.56 Hz, 1H), 6.90 (d, *J* = 7.90 Hz, 1H), 4.86 (q, *J* = 7.22 Hz, 1H), 4.77-4.72 (m, 1H), 4.60 (s, 2H), 3.73 (s, 3H), 3.16-3.03 (m, 2H), 1.44 (d, J = 8.59 Hz, 3H). ^{13}C NMR ($CDCl_3$, 100 MHz) 172.1, 171.8, 166.5, 141.2, 135.7, 133.7, 129.3, 128.8, 128.7, 127.7, 127.2, 53.5, 52.6, 49.1, 45.5, 37.9, 18.5. ESI-MS: $(M+H)^+$ m/z 447.

(B) Synthesis of 2g

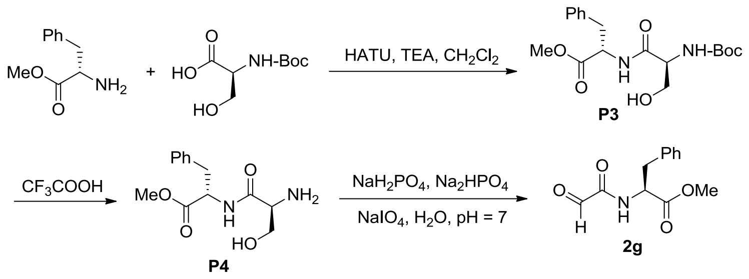

2g was synthesized according to the previous report (Alberti et al., 2009).

To a solution of Boc-L-serine (5.1 g, 25 mmol) in 100 mL of CH_2Cl_2 was added phenylalanine methyl ester hydrochloride (6.45 g, 30 mmol), followed by the addition of triethylamine (TEA) (7.5 mL, 50 mmol) and HBTU (11.4 g, 30 mmol). The mixture was stirred at room temperature overnight. The solvent was evaporated and the residue was dissolved in EtOAc (200 mL). The organic phase was washed with saturated citric acid (150 mL) and saturated aq. $NaHCO_3$ (150 mL) and then dried over anhydrous Na_2SO_4, filtered, and concentrated in vacuo. The residue was purified by flash silica gel column chromatography (petroleum ether/EtOAc, 1:1) affording product **P3** as colorless oil (5.94 g, 65 % yield).

To a solution of **P3** (6.31 g, 17.24 mmol) in 20 mL of CH_2Cl_2 was added 5 mL of TFA dropwise at 0 ℃, and the solution was stirred at room temperature for 3 h. The solvent was evaporated and the white residue was recrystallized affording product **P4** as a white solid (5.13 g, 82 % yield).

Sodium metaperiodate (1.015 g, 5.50 mmol) was added to a solution of peptide **P4** (1.0 g, 2.75 mmol) in 25 mM sodium phosphate buffer (pH 7.0) (5 mL). The solution was stirred at room temperature in the dark for 30 min. TLC revealed that no **P4** was remained. The solution was extracted with EtOAc (3 × 25 mL), and the combined organic phase was concentrated in vacuo. The residue was purified by flash silica gel column chromatography (petroleum ether/EtOAc, 1:1) to provide product **2g** as a colorless oil (380 mg, 59%).

3. General procedures for the visible-light photoredox olefination

General procedures for visible-light photoredox synthesis of terminal alkenes (3)

$[Ru(bpy)_3]Cl_2$•$6H_2O$ (**A**) (3.8 mg, 5.0 μmol) or $[Ir(ppy)_2]dtbbpyPF_6$ (**C**) (4.6 mg, 5.0 μmol), alkyl bromide (**1**) (1.0 mmol) (if solid), paraformaldehyde (**2a**) (4.0 mmol for synthesis of **3n-p**; 2.0 mmol for synthesis of the others, relative to amount of formaldehyde) or aqueous formaldehyde (37% aqueous solution) (**2b**) (2.0 mmol), triphenylphosphine (PPh_3) (786 mg, 3.0 mmol for

synthesis of **3n-p**; 393 mg, 1.5 mmol for synthesis of the others), K_2CO_3 (414 mg, 3.0 mmol for synthesis of **3n-p**; 207 mg, 1.5 mmol for synthesis of the others), MeCN (10 mL) were added to a 25 mL Schlenk tube equipped with a magnetic stir bar, and then the mixture was bubbled with argon through a syringe needle for 5 min. Alkyl halides (1.0 mmol) (if liquid) was added to the mixture under argon flow. The tube was sealed, and then irradiated with a 23 W fluorescent bulb (the tube was approximately 2.5 cm away from the light source) at room temperature (~25 ^oC) in a fume hood with fast stream of air. After completion of the reaction, 20 mL of water was added to the resulting solution, and the solution was exacted with diethyl ether (3 × 20 mL). The combined organic phase was concentrated by rotary evaporator, and the residue was purified by silica gel column chromatography to give the desired product (**3**).

General procedures for visible-light photoredox synthesis of substituted 3,3,3-trifluoropropenes (4)

$Ru(bpy)_3Cl_2{\cdot}6H_2O$ (3.8 mg, 5.0 μmol) or $[Ir(ppy)_2dtbbpy]PF_6$ (4.6 mg, 5.0 μmol), K_2CO_3 (414 mg, 3.0 mmol for synthesis of **4j** and **4k**; 207 mg, 1.5 mmol for synthesis of the others), PPh_3 (786 mg, 3.0 mmol for synthesis of **4j** and **4k**; 393 mg, 1.5 mmol for synthesis of the others), alkyl halide (1.0 mmol) (if solid) and CH_3CN (10 mL) were added to a 25 mL Schlenk tube equipped with a magnetic stir bar, and then the mixture was bubble with argon through a syringe needle for 5 min. Alkyl halides (1.0 mmol) (if liquid), 2,2,2-trifluoroacetaldehyde hydrate (75% aqueous solution) (**2c**) (2.2 mmol for synthesis of **4j** and **4k**; 1.1 mmol for synthesis of the others) or 2,2,2-trifluoro-1-methoxyethanol (**2d**) (1.1 mmol) were added to the mixture under argon flow. The tube was sealed, and then irradiated with a 23 W fluorescent bulb (the tube was approximately 2.5 cm away from the light source) at room temperature (~25 ^oC) in a fume hood with fast stream of air. After completion of the reaction, 20 mL of water was added to the resulting solution, and the solution was exacted with diethyl ether (3 × 20 mL). The combined organic phase was concentrated by rotary evaporator, and the residue was purified by silica gel column chromatography to give the desired product (**4**).

General procedures for visible-light photoredox olefination leading to internal alkenes (5)

$[Ru(bpy)_3]Cl_2{\bullet}6H_2O$ (**A**) (3.8 mg, 5.0 μmol) or $[Ir(ppy)_2]dtbbpyPF_6$ (**C**) (9.1 mg, 10 μmol), K_2CO_3 (207 mg, 1.5 mmol), PPh_3 (393 mg, 1.5 mmol), alkyl bromide (**1**) (1.5 mmol) (if solid), aldehydes (1.2 mmol) and DMF (2.0 mL) were added to a 25 mL Schlenk tube equipped with a magnetic stir

bar, and then the mixture was bubble with argon through a syringe needle for 5 min. Alkyl halides (1.5 mmol) (if liquid) was added to the mixture under argon flow. The tube was sealed, and then irradiated with a 23 W fluorescent bulb (the tube was approximately 2.5 cm away from the light source) at room temperature (~25 ^oC) in a fume hood with fast stream of air. After completion of the reaction, 20 mL of water was added to the resulting solution, and the solution was exacted with diethyl ether (3 × 20 mL). The combined organic phase was concentrated by rotary evaporator, and the residue was purified by silica gel column chromatography to give the desired product (**5**).

4. Gram scale synthesis

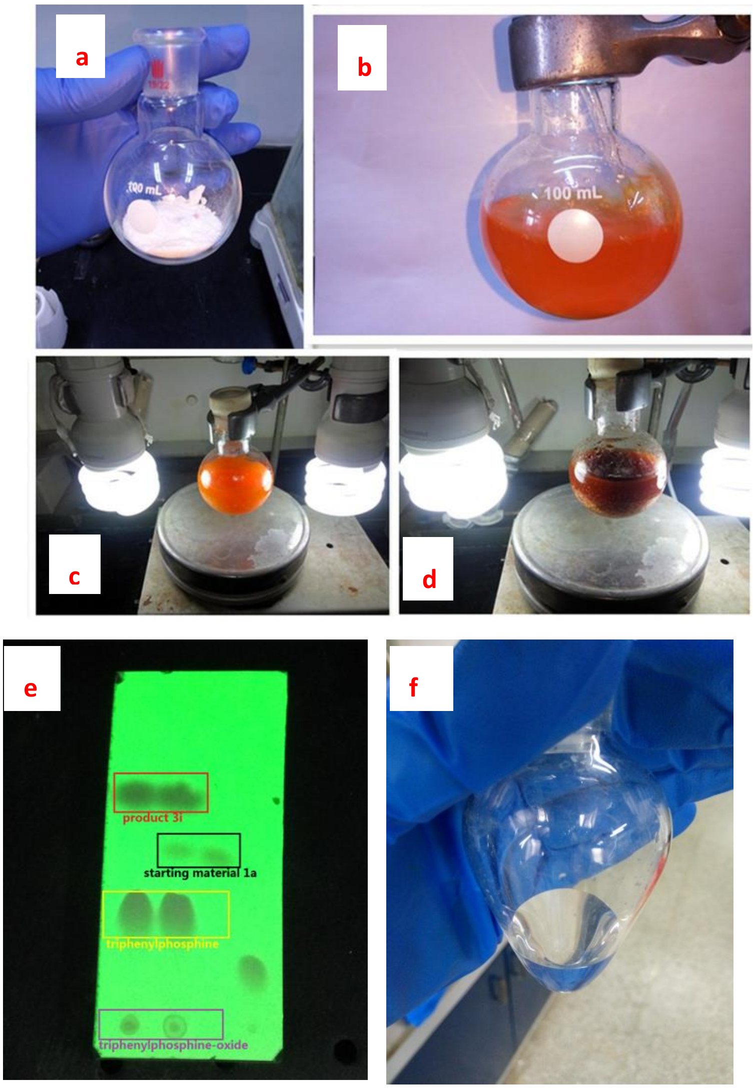

Figure S231 | Procedures for gram scale synthesis

(1) **Gram scale synthesis of 1-bromo-4-vinylbenzene (3i), related to Figure 2**. 4-Bromobenzyl bromide (**1a**) (5 g, 20 mmol), paraformaldehyde (**2a**) (1.2 g, 40 mmol), $[Ru(bpy)_3]Cl_2{\bullet}6H_2O$ (**A**) (37.4 mg, 50 μmol), PPh_3 (7.86 g, 30 mmol) and K_2CO_3 (4.08 g, 30 mmol) were added to a 100

mL rounded bottom bottle (**Fig. S231-a**), then 60 mL of DMF was added to the bottle, and the mixture was bubbled with Ar for 20 min (**Fig. S231-b**). The bottle was sealed and was irradiated with 2 × 23 W fluorescent lamp (approximately 4 cm away from the light source) at room temperature (~25 °C) in a fume hood with fast stream of air for 12 h (**Fig. S231-c**). After the reaction completed, the mixture colour was changed from orange to brown (**Fig. S231-d**). The resulting mixture was determined by TLC using hexane as the eluent (**Fig. S231-e**). The reaction was quenched with water (100 mL), and the aqueous solution was extracted with diethyl ether (3×100 mL). The combined organic phase was evaporated under reduced pressure, and the residue was purified with silica gel column chromatography to get the desired product (**3i**) (3.33 g, 90%) (Fig. S1-f).

(2) **Gram scale synthesis of (*E*)-1-Chloro-4-styrylbenzene (5i):** 4-Chlorobenzaldehyde (**2h**) (14.06 g, 100 mmol), $[Ru(bpy)_3]Cl_2{\bullet}6H_2O$ (**A**) (74.8 mg, 100 μmol), PPh_3 (39.3 g, 150 mmol), K_2CO_3 (20.4 g, 150 mmol) and DMF (200 mL) were added to a 500 mL rounded bottom bottle, and the mixture was bubbled with Ar for 20 min. Benzyl bromide (**1b**) (14.4 mL, 120 mmol) was added to the mixture under Ar. The bottle was sealed and irradiated with 2 × 23W fluorescent lamp (approximately 4 cm away from the light source) at room temperature (~25 °C) in a fume hood with fast stream of air for 36 h. The reaction was quenched with water (200 mL), and the resulting solution was extracted with diethyl ether (3 × 200 mL). The combined organic phase was dried over anhydrous Na_2SO_4 and evaporated under reduced pressure, and the residue was purified with silica gel column chromatography to get the desired product (**5i**) (19.54 g, 91%).

5. Mechanism study

(1) Synthesis and reaction of ^{18}O-labelled benzaldehyde (2j)

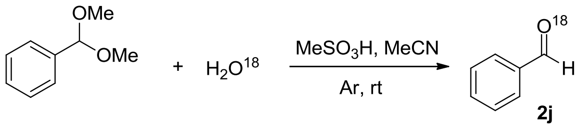

2j

Benzaldehyde dimethyl acetal (304 mg, 2 mmol), dry CH_3CN (2 mL) were added to a 5 mL round bottom bottle, and then H_2O^{18} (60 μL, 3 mmol) and $MeSO_3H$ (384 mg, 4 mmol) were added to the bottle under Ar. The mixture was stirred overnight under Ar at room temperature, then the solvent was removed, and the residue was purified by silica gel column chromatography to get the

^{18}O-labelled benzaldehyde (**2j**), 194.5 mg (90%). ^{1}H NMR ($CDCl_3$, 400 MHz) 10.0 (s, 1H), 7.88 (d, *J* = 7.56 Hz, 2H), 7.63 (t, *J* = 7.56 Hz, 1H), 7.53 (t, *J* = 7.56 Hz, 2H). ^{13}C NMR ($CDCl_3$, 100 MHz) 187.9, 137.1, 130.0, 135.3, 129.0. EIMS: M^+ m/z 108.

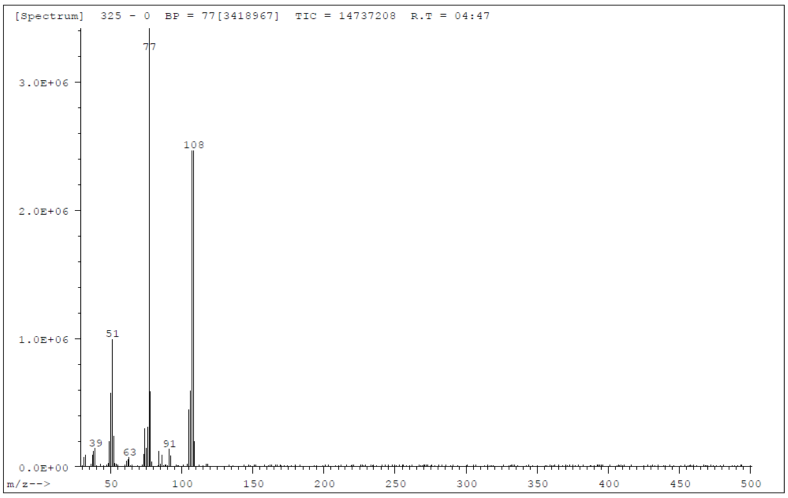

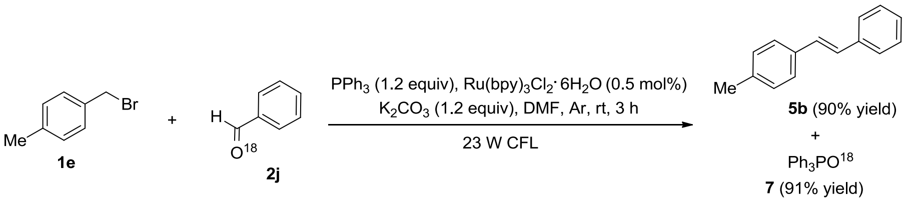

Reaction of ^{18}O-labeled benzaldehyde (**2j**) with 4-methylbenzyl bromide (**1e**) was performed under the standard conditions. ^{18}O-labeled triphenylphosphine-oxide (**7**) was obtained in 91% yield (254 mg) as a white solid. ^{1}H NMR ($CDCl_3$, 400 MHz) 7.66-7.61 (m, 2H), 7.52-7.48 (m, 1H), 7.44-7.40 (m, 2H). ^{13}C NMR ($CDCl_3$, 100 MHz) 133.1, 132.2, 132.1, 132.0, 128.6, 128.5. HRMS (ESI-TOF) calculated for $C_{18}H_{16}P^{18}O$ $[M+H]^+$ m/z 281.0998, found 281.1002.

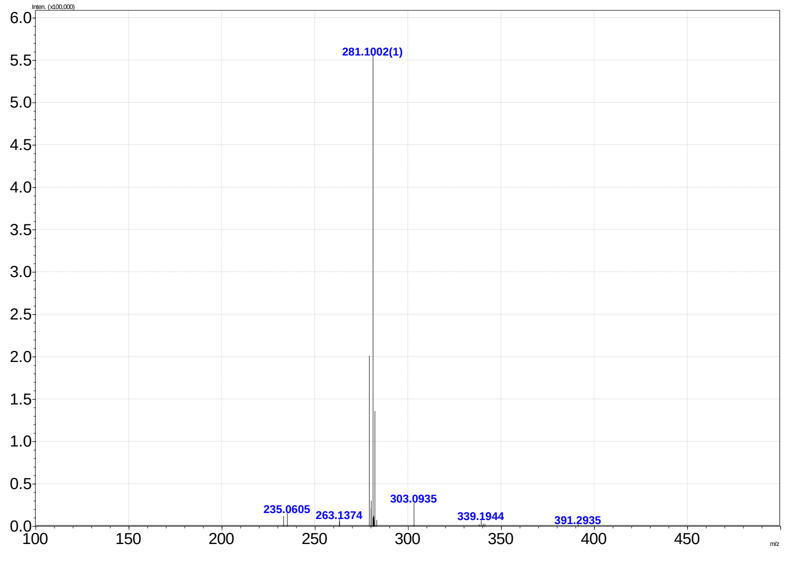

(2) Treatment of (4-methylbenzyl)triphenylphosphonium bromide (6) with 4-methylphenyldehyde (2i) under the standard conditions

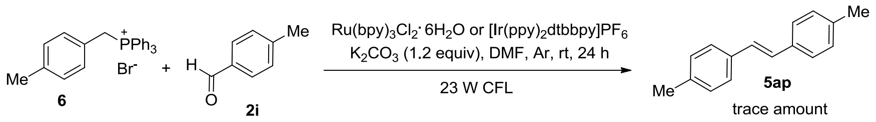

Treatment of 4-methylphenyldehyde (**2i**) (120 mg, 1.0 mmol) with a fresh synthesized (4-methylbenzyl)triphenylphosphonium bromide (**6**) (536 mg, 1.2 mmol) was performed with 0.5 mol% catalyst Ru(bpy)$_3$Cl$_2$•6H$_2$O (**A**) or [Ir(ppy)$_2$dtbbpy]PF$_6$ (**C**) as the photocatalyst under the standard conditions. After irradiation with 23 W CFL for 24 h, only trace amounts of (*E*)-1,2-dip-tolylethene (**5ai**) were observed. The same result was obtained when addition an additional equivalent of PPh$_3$. The result indicated that the mechanism in Fig. 3-5 was not a traditional Wittig coupling.

(3) Treatment of ^{18}O-labelled benzaldehyde (2j) with triphenylphosphine or triphenylphosphine and 1,1-diphenylethylene (8)

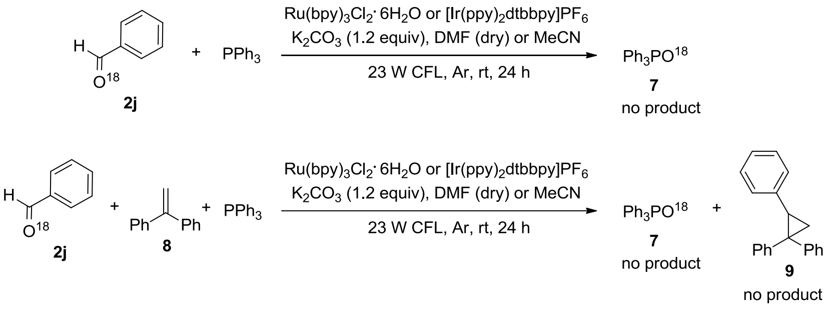

Treatment of ^{18}O-labelled benzaldehyde (**2j**) with triphenylphosphine (*in the absence of alkyl halide*) was performed under the standard reaction conditions for 24 h, and no ^{18}O-labeled triphenylphosphine oxide (**7**) was found. Subsequently, we investigated reaction of ^{18}O-labelled benzaldehyde (**2j**), triphenylphosphine and 1,1-diphenylethylene (**8**), and **7** and **9** were not observed.

(4) Treatment of 4-methylbenzyl bromide (1e) with triphenylphosphine

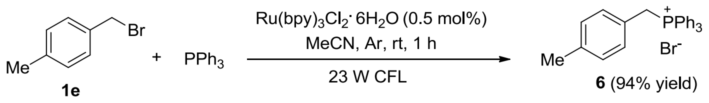

Treatment of 4-methylbenzyl bromide (**1e**) (1.0 mmol) with PPh$_3$ (1.0 mmol) (in the absence of aldehyde and base) was carried out under the standard conditions for 1 h, and (4-methylbenzyl)

triphenylphosphonium bromide (**6**) was obtained in 94% yield (see Fig. S233). A control experiment in the absence of visible light was performed, and only small amounts of **6** were observed.

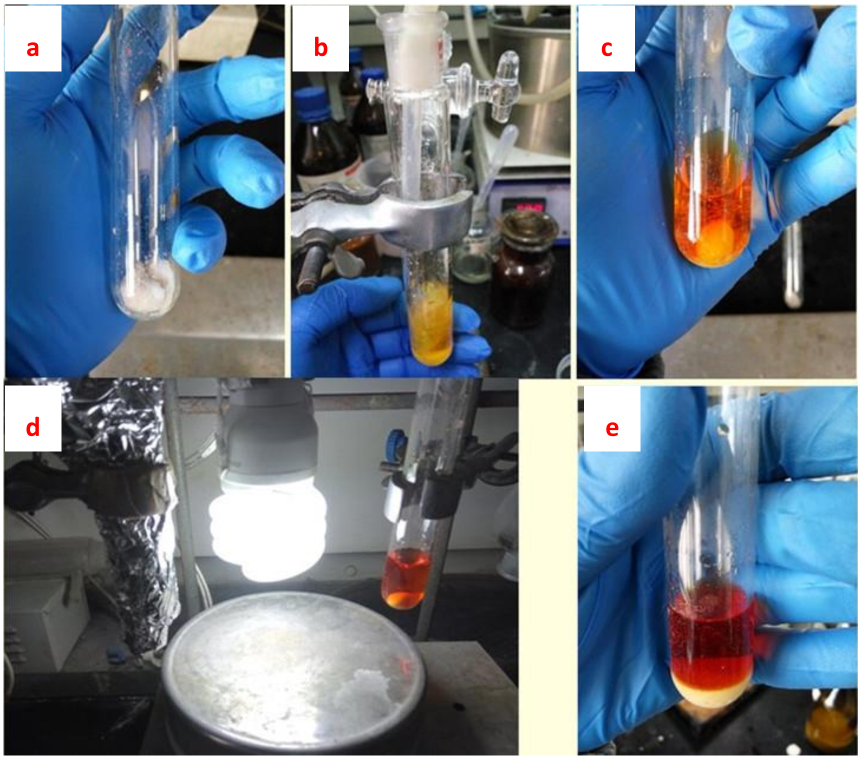

Figure S232 | Procedures for control experiment on reaction of 4-methylbenzyl bromide (1e) with PPh_3 under standard conditions (in the absence of aldehyde and base), related to Figure 1. (a) 4-methylbenzyl bromide (**1e**) (250 mg, 1 mmol) , PPh_3 (262.3 mg, 1 mmol) and $[Ru(bpy)_3]Cl_2$•$6H_2O$ (**A**) (3.8 mg, 5.0 μmol) were added to a 25 mL Schlenk tube; (b) After CH_3CN (6 mL) was added to the tube, the mixture was bubbled with Ar through a needle for over 5 min; (c) The tube was sealed, and small amounts of PPh_3 remained undissolved; (d) The tube was irradiated with a 23 W CFL bulb (the tube was approximately 2.5 cm away from the light source) at room temperature (~25 ^{o}C) in a fume hood with fast stream of air. 5 min later, all PPh_3 was dissolved, and the color changed into dark red from orange; (e) After the mixture was irradiated for 1 h, a white precipitation (4-methylbenzyl)triphenylphosphonium bromide (**6**) appeared.

(5) Electron spin resonance (ESR) determination conditions and HRMS of the intermediates

Electron spin resonance (ESR) experiment was recorded on an X-band JES FA200 (JEOL CO.). The experimental conditions are as follows: frequency 9.068 GHz, power 1 mW, center field

323.124 mT, sweep width 10 mT, modulation width 0.1 mT, sweep time 1 min, time constant 0.1 s.

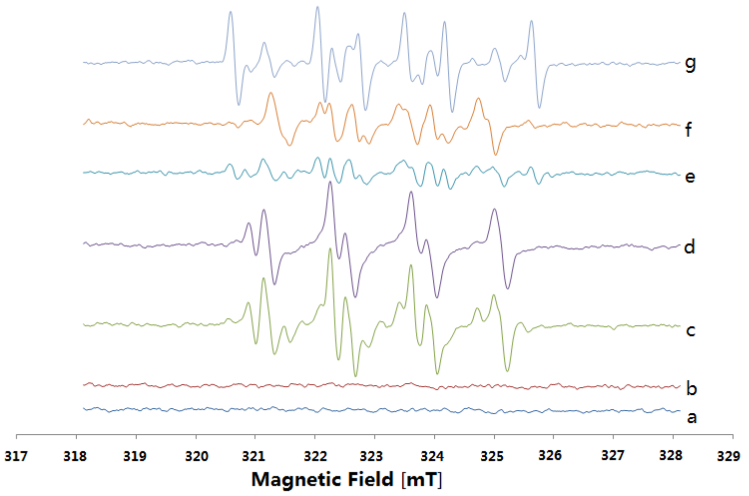

Figure S233 | ESR spectra of the radicals trapped by DMPO under different conditions: **ESR spectra of the radicals trapped by DMPO under different conditions (every experiment was performed in the presence of K_2CO_3 as the base), related to Figure 1.** (a) 4-Methyl benzyl bromide (**1e**) (50 mM), DMPO (100 mM), [Ru(bpy)$_3$]Cl$_2$•6H$_2$O (0.5 mM) in CH$_3$CN irradiated with a 23 W CFL bulb for 5 min; (b) 4-Methylbenzaldehyde (**2i**) (50 mM), DMPO (100 mM), [Ru(bpy)$_3$]Cl$_2$•6H$_2$O (0.5 mM) in CH$_3$CN irradiated with a 23 W CFL bulb for 5 min; (c) Triphenylphosphine (50 mM), DMPO (100 mM), [Ru(bpy)$_3$]Cl$_2$•6H$_2$O (0.5 mM) in CH$_3$CN irradiated with a 23 W CFL bulb for 5 min; (d) 4-Methylbenzaldehyde (**2i**) (50 mM), triphenylphosphine (50 mM), DMPO (100 mM), [Ru(bpy)$_3$]Cl$_2$•6H$_2$O (0.5 mM) in CH$_3$CN irradiated with a 23 W CFL bulb for 5 min; (e) 4-Methyl benzyl bromide (**1e**) (50 mM), triphenylphosphine (50 mM), DMPO (100 mM), [Ru(bpy)$_3$]Cl$_2$•6H$_2$O (0.5 mM) in CH$_3$CN irradiated with a 23 W CFL bulb for 5 min; (f) 4-Methyl benzyl bromide (**1e**) (50 mM), 4-methylbenzaldehyde (**2i**) (50 mM), DMPO (100 mM), [Ru(bpy)$_3$]Cl$_2$•6H$_2$O (0.5 mM) in CH$_3$CN irradiated with a 23 W CFL bulb for 5 min; (g) 4-Methyl benzyl bromide (**1e**) (50 mM), 4-methylbenzaldehyde (**2i**) (50 mM), DMPO (100 mM), triphenylphosphine (50 mM), [Ru(bpy)$_3$]Cl$_2$•6H$_2$O (0.5 mM) in CH$_3$CN irradiated with a 23 W CFL bulb for 5 min.

In order to explore mechanism for the visible-light photoredox olefination, we investigated which types of radicals were produced during the reactions by electron spin resonance (ESR). (a) A mixture of 4-methyl benzyl bromide (**1e**), 5,5-dimethyl-1-pyrroline *N*-oxide (DMPO) and photocatalyst [Ru(bpy)$_3$]Cl$_2$•6H$_2$O in CH$_3$CN was bubbled with Ar for 5 min, and the mixture was transferred to a quartz flat cell and irradiated with a 23 W CFL bulb for 5 min. The resulting

solution was tested by ESR, and no signal was observed (**Fig. S233-a**), which showed that the direct reduction of 4-methylbenzaldehyde (**2i**) is thermodynamically unfavoured. Subsequently, the similar procedures were performed in the following experiments. (b) No signal appeared in ESR spectrum of mixture of 4-methylbenzaldehyde (**2i**), DMPO and $[Ru(bpy)_3]Cl_2$•$6H_2O$ in CH_3CN (**Fig. S233-b**). (c) ESR super-hyperfine spectrum of mixture of triphenylphosphine, DMPO, $[Ru(bpy)_3]Cl_2$•$6H_2O$ in CH_3CN exhibited a signal of nitrogen-centred DMPO radical adduct **I-A** (see **Fig. S234-c**) (g = 2.005, A_N = 1.38 mT, A_H = 1.40 mT) (**Fig. S233-c**), and no phosphorus-centred DMPO radical adduct (A_H = 2.9 mT) (Alberti et al., 2009) was observed, which is attributed to quick reaction of triphenylphosphine cation radical with DMPO to generate a nitrogen-centred radical (see **Fig. S234-c**). (d) For mixture of 4-methylbenzaldehyde (**2i**), triphenylphosphine, DMPO, $[Ru(bpy)_3]Cl_2$•$6H_2O$ in CH_3CN, similar signal was observed (Fig. S3-d), which indicated that 4-methylbenzaldehyde (**2i**) did not participate in the radical reaction. (e) ESR spectrum of mixture of 4-methylphenyl bromide (**1e**), triphenylphosphine and photocatalyst $[Ru(bpy)_3]Cl_2$•$6H_2O$ provided a weak signal of C-centred DMPO radical adduct **I-E** (found in HRMS) (see **Fig. S234-e**) (g = 2.003, A_N = 1.48 mT, A_H = 2.02 mT) (Bunik et al., 2002) (**Fig. S233-e**). Comparing the results in **Fig. S232-a** and **S232-e**, we speculate that PPh_3 radical cation can form a charge-transfer complex with the benzyl bromide, which facilitates the reduction by Ru(I) (see **Fig. S234-e**). (f) Surprisingly, ESR spectrum of mixture of 4-methylphenyl bromide (**1e**), 4-methylbenzaldehyde (**2i**) and photocatalyst $[Ru(bpy)_3]Cl_2$•$6H_2O$ gave a weak sextet signal of oxygen-centred DMPO radical adduct **I-G** (see **Fig. S234-f**) (g = 2.003, A_N= 1.34 mT, A_H =1.15 mT) (Dikalov et al., 2001) (**Fig. S233-f**). A possible explanation is that formation of complex of **I-H** in Fig. **S234-f** with **1e** promotes reduction of **1e** by Ru(II), and subsequent treatment of benzyl radical with aldehyde **2i** gives oxygen-centred radical (see Fig. **S234-f**). (g) When all reagents appeared in the reaction system, a strong signal from carbon-centred DMPO radical adduct **I-E** was observed with a signal from nitrogen-centred DMPO radical adduct **I-A** appearing (see **Fig. S233-g** and **S234-g**). The results above indicate that the process for the visible-light photoredox olefination in Fig. 2 is reasonable. This report is the first example of broadly applicable reduction of simple benzyl halides by visible light photoredox catalysis, and more detailed mechanistic studies are underway to better understand this key step in the catalytic cycle. The intermediates

mentioned above **I-A**, **I-E** and **I-G** were detected by HRMS, **I-E** was found ([M+H]: calculated 219.2623, found 219.1626), but **I-A**, **I-G** were not found in HRMS.

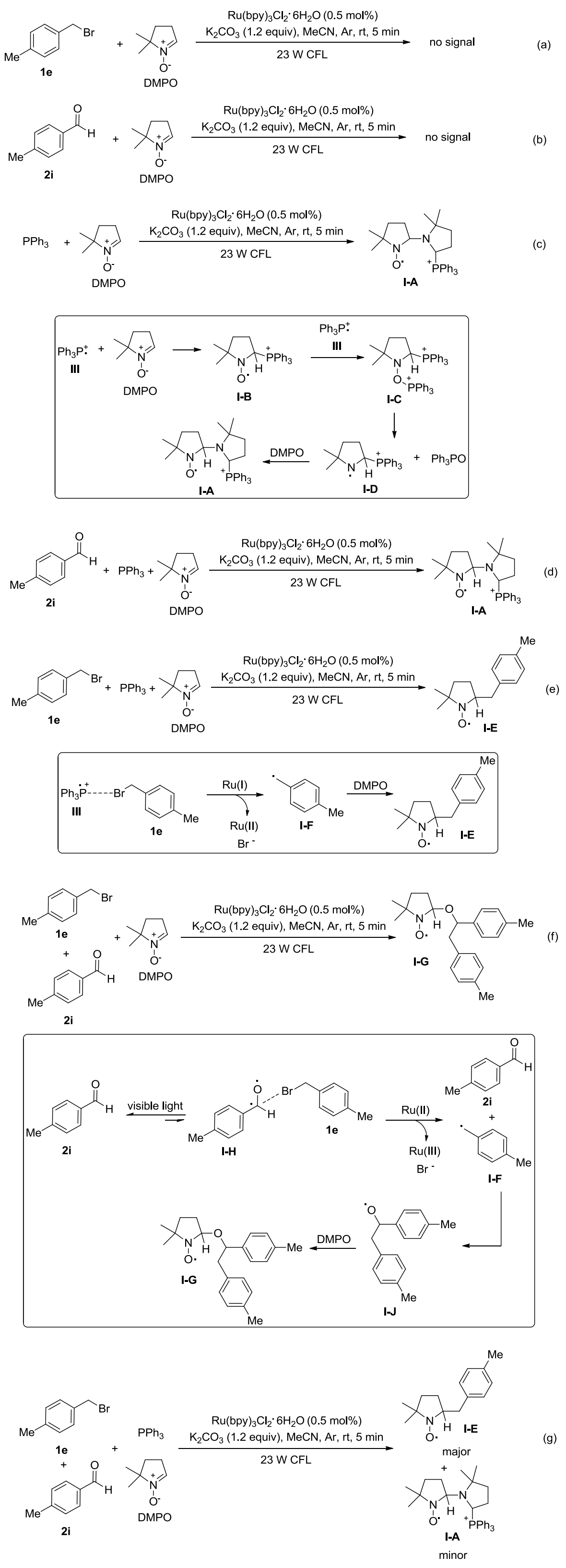

Figure S234 | Formation of various DMPO radical adducts, related to Figure 1.

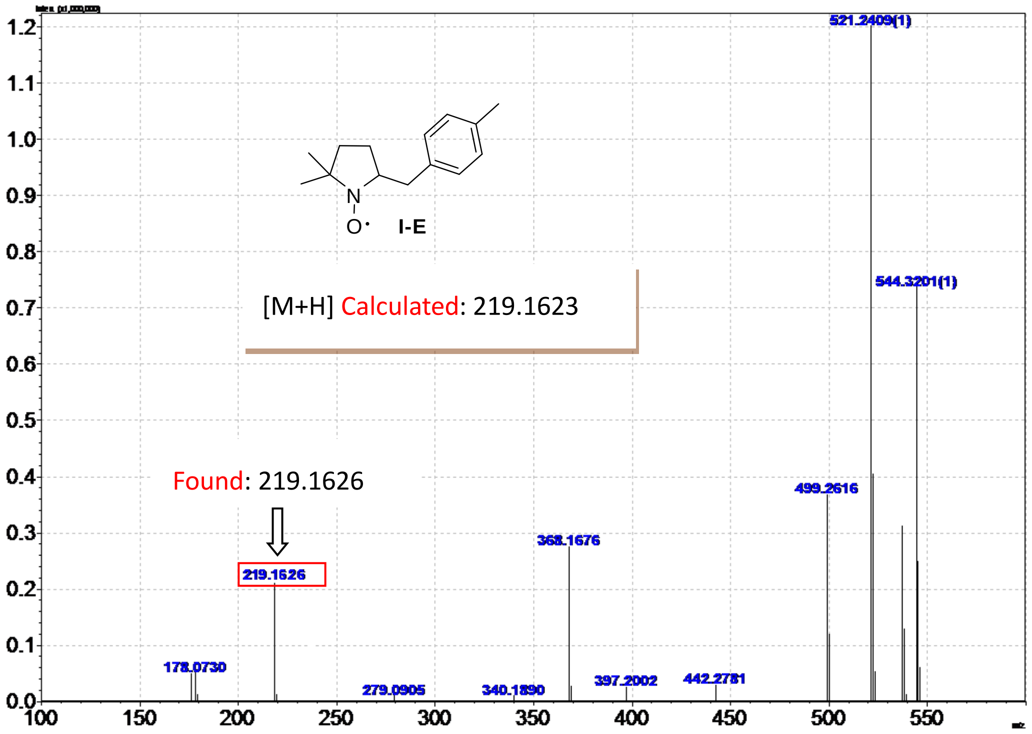

Figure S235 | HRMS of DMPO radical adducts, related to Figure 1.

(6) The CV (Cylic Voltammetry) of the reactants in DMF

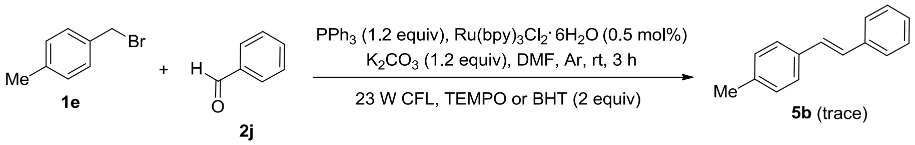

Figure S236 | Reaction of 1e with 2j in the presence of TEMPO or BHT, related to Figure 1.

We attempted reaction of **1e** with **2j** in the presence of TEMPO or BHT (2 equiv), and only trace amount of 5b was observed. The results showed that the reaction underwent a radical process.

(7) The CV (Cylic Voltammetry) of the reactants in DMF

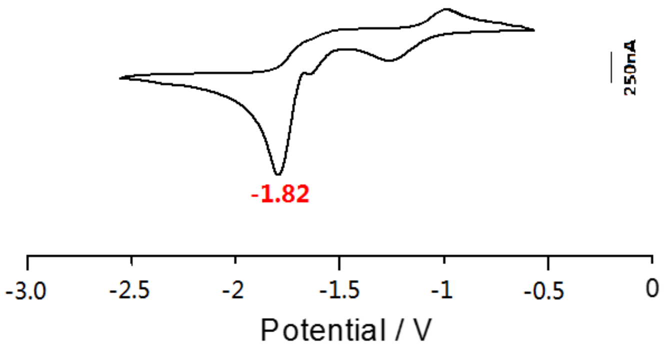

Figure S237. The CV of *p*-Tolualdehyde (0.1M) in 0.1M nBu$_4$NPF$_6$ in DMF at a Pt working electrode with a Pt counter electrode and Ag wire quasireference, related to Figure 1. Potential sweep rate was 50 mV/s.

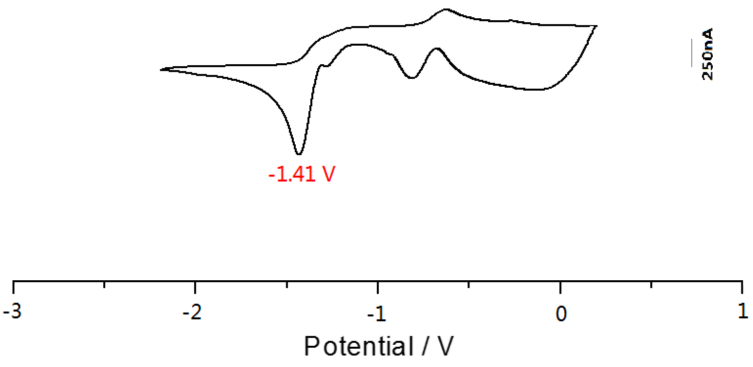

Figure S238 The CV of Triphenylphosphine (0.1M) in 0.1M nBu$_4$NPF$_6$ in DMF at a Pt working electrode with a Pt counter electrode and Ag wire quasireference, related to Figure 1. Potential sweep rate was 50 mV/s.

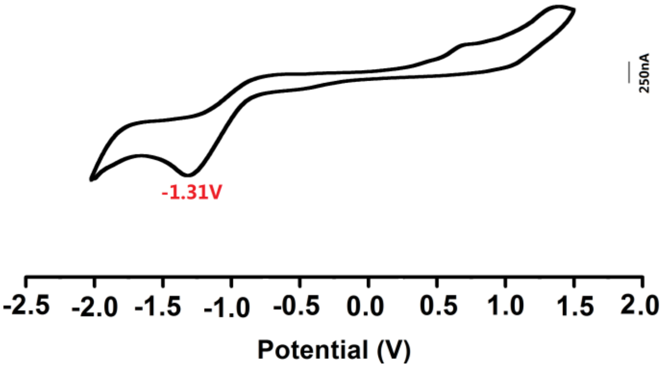

Figure S239. The CV of Ru(bpy)$_3$Cl$_2$ (0.1M) in 0.1M nBu$_4$NPF$_6$ in DMF at a Pt working electrode with a Pt counter electrode and Ag wire quasireference, related to Figure 1. Potential sweep rate was 50 mV/s.

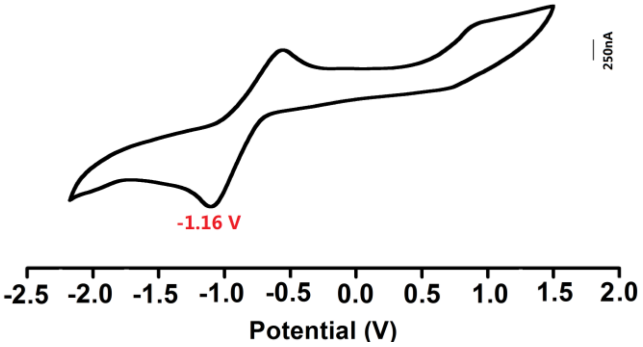

Figure S240. The CV of 4-Methylbenzyl bromide (0.1M) in 0.1M nBu$_4$NPF$_6$ in DMF at a Pt working electrode with a Pt counter electrode and Ag wire quasireference, related to Figure 1. Potential sweep rate was 50 mV/s.

The Cyclic Voltammetry experiment shows that the electron can transfer from triphenylphophine to photocatalyst Ru(bpy)$_3$Cl$_2$ easily, while can hardly to aldehydes.

6. Characterization data of compounds 3, 4 and 5

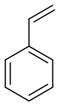

3a

Styrene (**3a**) (related to **Figure 2**) (Gärtner et al., 2015): Eluent: pentane, the solvent was removed at 0 ℃ under reduced pressure. Yield: X=Br, 70 mg (67%) with **2a** as the reactant, 64.8 mg (62%)

with **2b** as the reactant; X=Cl, 54.2 mg (52%) with **2a** as the reactant. Colorless oil. ^{1}H NMR ($CDCl_3$, 400 MHz) δ 7.41 (d, J = 6.87 Hz, 2H), 7.32 (t, J = 6.87 Hz, 2H), 7.24 (t, J = 6.87 Hz, 1H), 6.72 (dd, J_1 = 17.40 Hz, J_2 = 10.99 Hz, 1H), 5.75 (d, J = 18.78 Hz, 1H), 5.24 (d, J = 10.99 Hz, 1H). ^{13}C NMR ($CDCl_3$, 100 MHz) δ 137.7, 137.0, 128.6, 127.9, 126.3, 113.9. EI-MS: M^+ m/z 104.

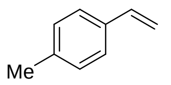

3b

1-Methyl-4-vinylbenzene (**3b**) (related to **Figure 2**) (Gärtner et al., 2015): Eluent: pentane, the solvent was removed at 0 ℃ under reduced pressure. Yield: X=Br, 89.7 mg (76%) with **2a** as the reactant, 82.6 mg (70%) with **2b** as the reactant; X=Cl, 56.6 mg (48%) with **2a** as the reactant. Colorless oil. ^{1}H NMR ($CDCl_3$, 400 MHz) δ 7.33 (d, J = 8.24 Hz, 2H), 7.16 (d, J = 7.79 Hz, 2H), 6.72 (dd, J_1 = 17.40 Hz, J_2 = 10.99 Hz, 1H), 5.72 (d, J = 17.40 Hz, 1H), 5.21 (d, J = 10.99 Hz, 1H), 2.37 (s, 3H). ^{13}C NMR ($CDCl_3$, 100 MHz) δ 137.7, 136.8, 135.0, 129.3, 126.3, 112.9, 21.3. EI-MS: M^+ m/z 118.

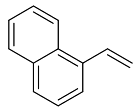

3c

1-Vinylnaphthalene (**3c**) (related to **Figure 2**) (Zhang et al., 2016): Eluent: pentane. Yield: 138.7 mg (90%). Colorless oil. ^{1}H NMR ($CDCl_3$, 400 MHz) δ 8.19 (d, J = 7.79 Hz, 1H), 7.92 (d, J = 7.79 Hz, 1H), 7.85 (d, J = 8.24 Hz, 1H), 7.70 (d, J = 7.33 Hz, 1H), 7.60-7.50 (m, 4H), 5.87 (d, J = 17.40 Hz, 1H), 5.55 (d, J = 10.53 Hz, 1H). ^{13}C NMR ($CDCl_3$, 100 MHz) δ 135.7, 134.5, 133.7, 131.2, 128.6, 128.2, 126.2, 125.9, 125.7, 123.9, 123.7. EI-MS: M^+ m/z 154.

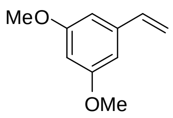

3d

1,3-Dimethoxy-5-vinylbenzene (**3d**) (related to **Figure 2**) (Cao et al., 2014): Eluent: pentane. Yield: 147.7 mg (90%). Colorless oil. ^{1}H NMR ($CDCl_3$, 400 MHz) δ 6.73 (dd, J_1 = 17.63 Hz, J_2 = 10.99 Hz, 1H), 6.66 (s, 2H), 6.48 (s, 1H), 5.81 (d, J = 17.86 Hz, 1H), 5.32 (d, J = 10.99 Hz, 1H), 3.84 (s, 6H). ^{13}C NMR ($CDCl_3$, 100 MHz) δ 161.0, 139.6, 136.9, 114.3, 104.3, 100.1, 55.3. EI-MS: M^+ m/z 154.

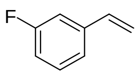

3e

1-Fluoro-3-vinylbenzene (**3e**) (related to **Figure 2**) (Wienhöfer et al., 2012): Eluent: pentane, the solvent was removed at 0 ℃ under reduced pressure. Yield: 91.6 mg (75%). Colorless oil. ^{1}H

NMR ($CDCl_3$, 400 MHz) δ 7.29-7.23 (m, 1H), 7.16-7.09 (m, 2H), 6.94 (t, J = 8.24 Hz, 1H), 6.67 (dd, J_1 = 17.40 Hz, J_2 = 10.99 Hz, 1H), 5.75 (d, J = 17.86 Hz, 1H), 5.29 (d, J = 10.99 Hz, 1H). ^{19}F ($CDCl_3$, 376.5 MHz) δ -112.4. ^{13}C NMR ($CDCl_3$, 100 MHz) δ 163.2 ($J_{F\text{-}C}$ = 245.37 Hz), 140.0 ($J_{F\text{-}C}$ = 7.67 Hz), 136.0, 130.1 ($J_{F\text{-}C}$ = 8.63 Hz), 122.3 ($J_{F\text{-}C}$ = 2.88 Hz), 115.3, 114.7 ($J_{F\text{-}C}$ = 22.04 Hz), 112.8 ($J_{F\text{-}C}$ = 21.09 Hz). EI-MS: M^+ m/z 122.

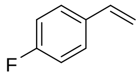

3f

1-Fluoro-4-vinylbenzene (**3f**) (related to **Figure 2**) (Gärtner et al., 2015): Eluent: pentane, the solvent was removed at 0 ℃ under reduced pressure. Yield: 94.0 mg (77%). Colorless oil. ^1H NMR ($CDCl_3$, 400 MHz) δ 7.38-7.35 (m, 2H), 7.01 (t, J = 8.70 Hz, 2H), 6.67 (dd, J_1 = 17.63 Hz, J_2 = 10.99 Hz, 1H), 5.67 (d, J = 17.86 Hz, 1H), 5.23 (d, J = 10.99 Hz, 1H). ^{19}F ($CDCl_3$, 376.5 MHz) δ -114.3. ^{13}C NMR ($CDCl_3$, 100 MHz) δ 162.6 ($J_{F\text{-}C}$ = 247.28 Hz), 135.8, 133.9 ($J_{F\text{-}C}$ = 3.83 Hz), 127.9 ($J_{F\text{-}C}$ = 7.67 Hz), 115.5 ($J_{F\text{-}C}$ = 22.04 Hz), 113.6. EI-MS: M^+ m/z 122.

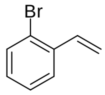

3g

1-Bromo-2-vinylbenzene (**3g**) (related to **Figure 2**) (Zhang et al., 2016): Eluent: pentane, the solvent was removed at 0 ℃ under reduced pressure. Yield: 142 mg (78%) with **2a** as the reactant, 138.36 mg (76%) with **2b** as the reactant. Colorless oil. ^1H NMR ($CDCl_3$, 400 MHz) δ 7.53 (d, J = 8.24 Hz, 2H), 7.26 (t, J = 7.33 Hz, 1H), 7.12-7.02 (m, 2H), 5.69 (d, J = 17.40 Hz, 1H), 5.35 (d, J = 10.99 Hz, 1H). ^{13}C NMR ($CDCl_3$, 100 MHz) δ 137.6, 135.9, 133.0, 129.2, 127.6, 126.9, 123.7, 116.8. EI-MS: M^+ m/z 182.

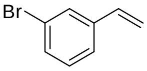

3h

1-Bromo-3-vinylbenzene (**3h**) (related to **Figure 2**) (Planellas et al., 2014): Eluent: pentane, the solvent was removed at 0 ℃ under reduced pressure. Yield: 162.9 mg (89%). Colorless oil. ^1H NMR ($CDCl_3$, 400 MHz) δ 7.56 (s, 1H), 7.53 (d, J = 8.24 Hz, 2H), 7.39 (d, J = 7.79 Hz, 1H), 7.32 (d, J = 7.79 Hz, 1H), 7.20 (t, J = 7.79 Hz, 1H), 6.65 (dd, J_1 = 17.63 Hz, J_2 = 10.99 Hz, 1H), 5.76 (d, J = 17.40 Hz, 1H), 5.30 (d, J = 10.99 Hz, 1H). ^{13}C NMR ($CDCl_3$, 100 MHz) δ 139.8, 135.6, 130.8, 130.2, 129.3, 125.0, 122.9, 115.5. EI-MS: M^+ m/z 182.

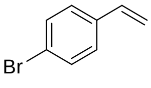

3i

1-Bromo-4-vinylbenzene (**3i**) (related to **Figure 2**) (Zhang et al., 2016): Eluent: pentane, the solvent was removed at 0 ℃ under reduced pressure. Yield: X=Br, 167.5 mg (92%) with **2a** as the reactant, 161.2 mg (89%) with **2b** as the reactant; X=Cl, 111.0 mg (61%) with **2a** as the reactant. Colorless oil. ^{1}H NMR ($CDCl_3$, 400 MHz) δ 7.43 (d, J = 8.70 Hz, 2H), 7.26 (d, J = 8.70 Hz, 2H), 6.64 (dd, J_1 = 17.63 Hz, J_2 = 10.99 Hz, 1H), 5.73 (d, J = 17.40 Hz, 1H), 5.27 (d, J = 10.99 Hz, 1H). ^{13}C NMR ($CDCl_3$, 100 MHz) δ 136.6, 135.9, 131.7, 127.9, 121.7, 114.7. EI-MS: M^+ m/z 182.

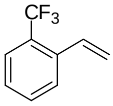

3j

1-(Trifluoromethyl)-2-vinylbenzene (**3j**) (related to **Figure 2**) (Planellas et al., 2014): Eluent: pentane, the solvent was removed at 0 ℃ under reduced pressure. Yield: 123.9 mg (72%). Colorless oil. ^{1}H NMR ($CDCl_3$, 400 MHz) δ 7.65 (t, J = 8.70 Hz, 2H), 7.52 (t, J = 7.79 Hz, 1H), 7.36 (t, J = 7.79 Hz, 1H), 7.16-7.08 (m, 1H), 5.75 (d, J = 16.94 Hz, 1H), 5.43 (d, J = 10.99 Hz, 1H). ^{19}F ($CDCl_3$, 376.5 MHz) δ -59.4. ^{13}C NMR ($CDCl_3$, 100 MHz) δ 136.9, 133.2, 132.0, 127.6, 127.5 ($J_{F\text{-}C}$ = 29.71 Hz), 127.2, 125.8 ($J_{F\text{-}C}$ = 5.75 Hz), 123.1, 118.1. EI-MS: M^+ m/z 172.

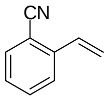

3k

2-Vinylbenzonitrile (**3k**) (related to **Figure 2**) (Xu et al., 2008): Eluent: hexane/ethyl acetate 10:1. Yield: 116.2 mg (90%). Colorless oil. ^{1}H NMR ($CDCl_3$, 400 MHz) δ 7.66 (d, J = 8.24 Hz, 1H), 7.60 (d, J = 7.33 Hz, 1H), 7.54 (t, J = 7.79 Hz, 1H), 7.32 (t, J = 8.70 Hz, 1H), 7.06 (dd, J_1 = 17.40 Hz, J_2 = 10.99 Hz, 1H), 5.93 (d, J = 17.40 Hz, 1H), 5.52 (d, J = 10.99 Hz, 1H). ^{13}C NMR ($CDCl_3$, 100 MHz) δ 140.7, 132.9, 128.9, 128.8, 128.0, 125.5, 119.0, 117.8, 111.2. EI-MS: M^+ m/z 129.

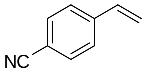

3l

4-Vinylbenzonitrile (**3l**) (related to **Figure 2**) (Gärtner et al., 2015): Eluent: hexane/ethyl acetate 10:1. Yield: X=Br, 114.9 mg (89%) with **2a** as the reactant, 112.3 mg (87%) with **2b** as the reactant; X=Cl, 83.9 mg (65%) with **2a** as the reactant. Light yellow oil. ^{1}H NMR ($CDCl_3$, 400 MHz) δ 7.59 (d, J = 8.24 Hz, 2H), 7.46 (d, J = 8.24 Hz, 2H), 6.70 (dd, J_1 = 17.86 Hz, J_2 = 10.99

Hz, 1H), 5.93 (d, J = 17.86 Hz, 1H), 5.43 (d, J = 10.99 Hz, 1H). ^{13}C NMR ($CDCl_3$, 100 MHz) δ 141.9, 135.4, 132.4, 126.8, 119.0, 117.8, 111.1. EI-MS: M^+ m/z 129.

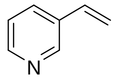

3m

3-Vinylpyridine (**3m**) (related to **Figure 2**) (Gärtner et al., 2015): Eluent: pentane/CH_2Cl_2 3:1, the solvent was removed at 0 ℃ under reduced pressure. Yield: 70.4 mg (67%). Yellow oil. ^{1}H NMR ($CDCl_3$, 400 MHz) δ 8.61 (s, 1H), 8.48 (d, J = 6.41 Hz, 1H), 7.73 (d, J = 8.79 Hz, 1H), 7.28-7.24 (m, 1H), 6.70 (dd, J_1 = 17.86 Hz, J_2 = 10.99 Hz, 1H), 5.83 (d, J = 17.86 Hz, 1H), 5.38 (d, J = 10.99 Hz, 1H). ^{13}C NMR ($CDCl_3$, 100 MHz) δ 148.9, 148.3, 133.5, 133.1, 132.8, 123.5, 116.3. EI-MS: M^+ m/z 105.

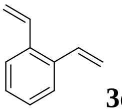

3o

1,2-Divinylbenzene (**3o**) (related to **Figure 2**) (Tanaka et al., 2008): Eluent: pentane, the solvent was removed at 0 ℃ under reduced pressure. Yield: 114.5 mg (88%). Colorless oil. ^{1}H NMR ($CDCl_3$, 400 MHz) δ 7.46-7.43 (m, 2H), 7.25-7.23 (m, 2H), 7.01 (dd, J_1 = 17.40 Hz, J_2 = 10.99 Hz, 2H), 5.62 (d, J = 17.40 Hz, 2H), 5.32 (d, J = 10.99 Hz, 2H). ^{13}C NMR ($CDCl_3$, 100 MHz) δ 136.2, 134.9, 127.9, 126.4, 116.5. EI-MS: M^+ m/z 130.

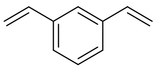

3n

1,3-Divinylbenzene (**3n**) (related to **Figure 2**) (Tanaka et al., 2008): Eluent: pentane, the solvent was removed at 0 ℃ under reduced pressure. Yield: 117.1 mg (90%). Colorless oil. ^{1}H NMR ($CDCl_3$, 400 MHz) δ7.45 (s, 1H), 7.35-7.28 (m, 3H), 6.74 (dd, J_1 = 17.40 Hz, J_2 = 10.99 Hz, 2H), 5.79 (d, J = 17.40 Hz, 2H), 5.28 (d, J = 10.99 Hz, 2H). ^{13}C NMR ($CDCl_3$, 100 MHz) δ 137.9, 136.9, 128.8, 125.7, 124.4, 114.2. EI-MS: M^+ m/z 130.

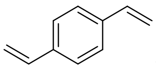

3p

1,4-Divinylbenzene (**3p**) (related to **Figure 2**) (Tanaka et al., 2008): Eluent: pentane, the solvent was removed at 0 ℃ under reduced pressure. Yield: 118.4 mg (91%). Colorless oil. ^{1}H NMR ($CDCl_3$, 400 MHz) δ 7.40 (s, 4H), 6.74 (dd, J_1 = 17.40 Hz, J_2 = 10.99 Hz, 2H), 5.78 (d, J = 17.40 Hz, 2H), 5.27 (d, J = 10.99 Hz, 2H). ^{13}C NMR ($CDCl_3$, 100 MHz) δ 137.2, 136.6, 126.5, 113.9. EI-MS: M^+ m/z 130.

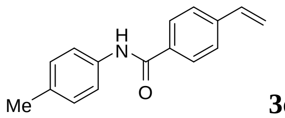

3q

N-*p*-Tolyl-4-vinylbenzamide (**3q**) (related to **Figure 2**) (Pogosyan et al., 1979): Eluent: hexane/ethyl acetate 5:1. Yield: 215.7 mg (91%). White solid, mp. 182-183 ℃. ^{1}H NMR ($CDCl_3$, 400 MHz) δ 7.82 (d, J = 7.33 Hz, 2H), 7.53-7.46 (m, 4H), 7.16 (d, J = 8.24 Hz, 2H), 6.75 (dd, J_1 = 17.40 Hz, J_2 = 10.99 Hz, 1H), 5.85 (d, J = 17.40 Hz, 1H), 5.38 (d, J = 10.99 Hz, 1H), 2.34 (s, 3H). ^{13}C NMR ($CDCl_3$, 100 MHz) δ 165.4, 141.0, 136.0, 135.5, 134.4, 134.2, 129.7, 127.5, 126.6, 120.4, 116.3, 21.0. ESI-MS: $[M+H]^+$ m/z 238.

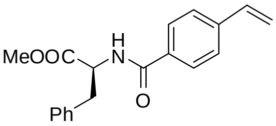

3r

(*S*)-Methyl 3-phenyl-2-(4-vinylbenzamido)propanoate (**3r**) (related to **Figure 2**) (Poulsen et al., 2006): Eluent: hexane/ethyl acetate 5:1. Yield: 278.2 mg (90%). Colorless solid, mp. 56-57 ℃. ^{1}H NMR ($CDCl_3$, 400 MHz) δ 7.69 (d, J = 8.24 Hz, 2H), 7.44 (d, J = 8.24 Hz, 2H), 7.31-7.25 (m, 3H), 7.13 (d, J = 8.24 Hz, 2H), 6.73 (dd, J_1 = 17.63 Hz, J_2 = 10.99 Hz, 1H), 6.60 (d, J = 7.33 Hz, 1H), 5.83 (d, J = 16.94 Hz, 1H), 5.35 (d, J = 11.45 Hz, 1H), 5.09 (dd, J_1 = 13.05 Hz, J_2 = 5.95 Hz, 1H), 3.76 (s, 3H), 3.32-3.20 (m, 2H). ^{13}C NMR ($CDCl_3$, 100 MHz) δ 172.2, 166.5, 141.1, 136.0, 135.9, 133.0, 129.5, 128.7, 127.4, 127.3, 126.5, 116.2, 53.6, 52.6, 38.0. ESI-MS: $[M+H]^+$ m/z 310.

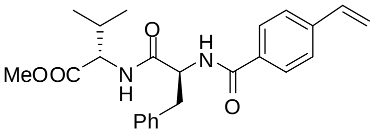

3s

(*S*)-Methyl 3-methyl-2-((S)-3-phenyl-2-(4-vinylbenzamido)propanamido)butanoate (**3s**) (related to **Figure 2**): Eluent: hexane/ethyl acetate 2:1. Yield: 330.5 mg (81%). White solid. ^{1}H NMR ($CDCl_3$, 400 MHz) δ 7.69 (d, J = 8.24 Hz, 2H), 7.41 (d, J = 8.24 Hz, 2H), 7.27-7.08 (m, 6H), 6.79-6.68 (m, 2H), 5.82 (d, J = 17.86 Hz, 1H), 5.35 (d, J = 10.53 Hz, 1H), 5.00 (m, 1H),4.44 (dd, J_1 = 8.47 Hz, J_2 = 5.50 Hz, 1H), 3.71 (s, 3H), 3.26-3.15 (m, 2H), 2.13-2.05 (m, 1H), 0.83 (dd, J_1 = 9.16 Hz, J_2 = 6.87 Hz, 1H). ^{13}C NMR ($CDCl_3$, 100 MHz) δ 171.8, 171.2, 167.0, 141.0, 136.7, 136.0, 132.9, 129.5, 128.7, 127.6, 127.1, 126.4, 116.2, 57.6, 54.9, 52.2, 38.3, 31.2, 19.0, 17.9. HRMS (ESI-TOF) calculated for $C_{24}H_{28}N_2NaO_4$ $[M+Na]^+$ m/z 431.1941, found 431.1942.

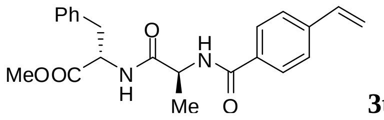

3t

(*S*)-Methyl 3-methyl-2-((S)-3-phenyl-2-(4-vinylbenzamido)propanamido)butanoate (**3t**) (related to **Figure 2**): Eluent: hexane/ethyl acetate 2:1,. Yield: 315.5 mg (83%). White solid. ^{1}H NMR ($CDCl_3$, 400 MHz) δ 7.74 (d, *J* = 6.87 Hz, 2H), 7.40 (d, *J* = 7.79 Hz, 2H), 7.34 (d, *J* = 8.24 Hz, 1H), 7.21 (d, *J* = 7.33 Hz, 1H), 7.14-7.05 (m, 5H), 6.71 (dd, J_1 = 17.63 Hz, J_1 = 10.99 Hz, 1H), 5.81 (d, *J* = 17.86 Hz, 1H), 5.34 (d, *J* = 10.99 Hz, 1H), 4.86-4.79 (m, 2H), 3.68 (s, 3H), 3.13-2.97 (m, 2H), 1.45 (d, *J* = 6.87 Hz, 3H). ^{13}C NMR ($CDCl_3$, 100 MHz) δ 172.4, 171.8, 166.7, 140.8, 135.92, 135.86, 132.7, 129.2, 128.5, 127.6, 127.0, 126.2, 116.1, 53.6, 52.4, 49.0, 37.8, 18.5. HRMS (ESI-TOF) calculated for $C_{22}H_{24}N_2NaO_4$ $[M+Na]^+$ m/z 403.1628, found 403.1629.

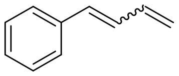

3u

Buta-1,3-dienylbenzene (**3u**) (related to **Figure 2**) (Lishchynskyi et al., 2012): Eluent: pentane. Yield: 93.7 mg (72%). Colorless liquid. ^{1}H NMR ($CDCl_3$, 300 MHz) δ 7.67-7.24 (m, 5H), 7.17-6.22 (m, 3H), 5.61-5.23 (m, 2H). ^{13}C NMR ($CDCl_3$, 75 MHz) δ 137.5, 137.3, 137.2, 133.3, 133.0, 130.9, 130.5, 129.7, 129.1, 128.7, 128.4, 127.8, 127.2, 126.6, 119.8, 117.8. EI-MS: M^+ m/z 130.

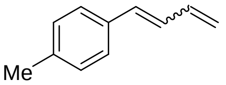

3v

1-(Buta-1,3-dienyl)-4-methylbenzene (**3v**) (related to **Figure 2**) (Lishchynskyi et al., 2012): Eluent: pentane. Yield: 110.9 mg (77%) E/Z 38:62. Colorless liquid. ^{1}H NMR ($CDCl_3$, 400 MHz) δ 7.30-7.20 (m, 2H), 7.15-6.10 (m, 2H), 6.91-6.70 (m, 1H), 6.54-6.18 (m, 2H), 5.36-5.12 (m, 2H), 2.34-2.32 (s, 3H). ^{13}C NMR ($CDCl_3$, 100 MHz) δ 137.6, 137.5, 137.0, 134.6, 134.5, 133.5, 133.0, 130.5, 130.3, 129.5, 129.1, 128.8, 126.5, 119.3, 117.1, 21.4, 21.3. EI-MS: M^+ m/z 144.

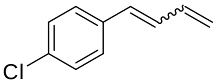

3w

1-(Buta-1,3-dienyl)-4-chlorobenzene (**3w**) (related to **Figure 2**) (Lishchynskyi et al., 2012): Eluent: pentane. Yield: 116.5 mg (71%) E/Z 30:70. Colorless liquid. ^{1}H NMR ($CDCl_3$, 400 MHz) δ 7.32-7.22 (m, 4H), 6.85-6.70 (m, 1H), 6.52-6.23 (m, 2H), 5.41-5.18 (m, 2H). ^{13}C NMR ($CDCl_3$,

100 MHz) δ 137.0, 135.9, 135.7, 133.3, 132.92, 132.86, 131.6, 131.4, 130.4, 130.3, 129.2, 128.9, 128.5, 127.7, 120.4, 118.4. EI-MS: M^+ m/z 164.

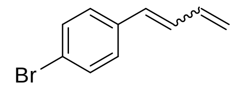

3x

1-Bromo-4-(buta-1,3-dienyl)benzene (**3x**) (related to **Figure 2**) (Mundal et al., 2009): Eluent: pentane. Yield: 181.0 mg (87%) E/Z 50:50. Colorless liquid. ^{1}H NMR ($CDCl_3$, 400 MHz) δ 7.45-7.7.40 (m, 2H), 7.25-7.15 (m, 2H), 6.84-6.72 (m, 1H), 6.52-6.43 (m, 1H), 6.37-6.23 (m, 1H), 5.41-5.19 (m, 2H). ^{13}C NMR ($CDCl_3$, 100 MHz) δ 137.0, 136.3, 136.2, 132.8, 131.8, 131.6, 131.5, 131.48, 130.7, 130.4, 129.2, 128.0, 121.5, 121.1, 120.5, 118.5. EI-MS: M^+ m/z 208.

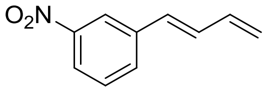

3y

1-(Buta-1,3-dienyl)-3-nitrobenzene (**3y**) (related to **Figure 2**): Eluent: pentane. Yield: 112.6 mg (70%). Yellow liquid. ^{1}H NMR ($CDCl_3$, 400 MHz) δ 8.24 (s, 1H), 8.07 (d, J = 8.24 Hz, 1H), 7.69 (d, J = 7.79 Hz, 1H), 7.48 (t, J = 7.79 Hz, 1H), 6.93-6.87 (m, 1H), 6.62-6.48 (m, 2H), 5.45 (d, J = 16.94 Hz, 1H), 5.30 (d, J = 10.07 Hz, 1H). ^{13}C NMR ($CDCl_3$, 100 MHz) δ 148.8, 139.1, 136.4, 132.6, 132.3, 130.3, 129.6, 122.2, 121.0, 120.2. EI-MS: M^+ m/z 175.

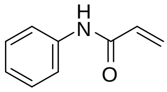

3z

N-Phenylacrylamide (**3z**) (related to **Figure 2**) (Eriksson et al., 2007): Eluent: hexane/ethyl acetate 5:1. Yield: 104.4 mg (71%). White solid, mp. 103-104 ℃. ^{1}H NMR ($CDCl_3$, 400 MHz) δ 8.04 (s, 1H), 7.60 (d, J = 7.33 Hz, 2H), 7.30 (t, J = 7.79 Hz, 2H), 7.11 (t, J = 7.33 Hz, 1H), 6.44- 6.26 (m, 2H), 5.72 (d, J = 9.62 Hz, 1H). ^{13}C NMR ($CDCl_3$, 100 MHz) δ 164.0, 137.9, 131.4, 129.1, 127.8, 124.6, 120.3. EI-MS: M^+ m/z 147.

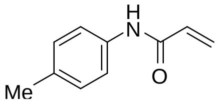

3aa

N-*p*-Tolylacrylamide (**3aa**) (related to **Figure 2**) (Eriksson et al., 2007): Eluent: hexane/ethyl acetate 5:1. Yield: 109.5 mg (68%). White solid, mp. 140-141 ℃. ^{1}H NMR ($CDCl_3$, 400 MHz) δ 7.71 (s, 1H), 7.47 (d, J = 8.24 Hz, 2H), 7.11 (d, J = 8.24 Hz, 2H), 6.40 (d, J = 16.94 Hz, 1H), 6.26

(dd, J_1= 16.72 Hz, J_1= 10.07 Hz, 1H), 5.71 (d, J = 10.07 Hz, 1H), 2.31 (s, 3H). ^{13}C NMR ($CDCl_3$, 100 MHz) δ 163.7, 135.4, 134.3, 131.4, 129.6, 127.6, 120.3, 21.0. EI-MS: M^+ m/z 162.

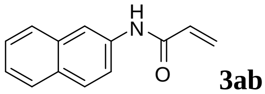

3ab

N-(Naphthalen-2-yl)acrylamide (**3ab**) (related to **Figure 2**) (Eriksson et al., 2007): Eluent: hexane/ethyl acetate 5:1. Yield: 132.1 mg (67%). White solid, mp. 174-175 ℃. ^{1}H NMR ($CDCl_3$, 400 MHz) δ 8.29 (s, 1H), 8.16 (s, 1H), 7.76-7.71 (m, 3H), 7.53 (d, J = 8.70 Hz, 1H), 7.44-7.38 (m, 2H), 6.47 (d, J = 16.94 Hz, 1H), 6.34 (dd, J_1= 16.94 Hz, J_1= 10.07 Hz, 1H), 5.73 (d, J = 10.07 Hz, 1H). ^{13}C NMR ($CDCl_3$, 100 MHz) δ 164.2, 135.4, 133.9, 131.3, 130.9, 128.8, 128.0, 127.8, 127.6, 126.6, 125.2, 120.2, 117.3. ESI-MS: $[m+H]^+$ m/z 198.

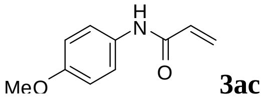

3ac

N-(4-Methoxyphenyl)acrylamide (**3ac**) (related to **Figure 2**) (Eriksson et al., 2007): Eluent: hexane/ethyl acetate 5:1. Yield: 132.1 mg (67%). White solid, mp. 97-98 ℃. ^{1}H NMR ($CDCl_3$, 400 MHz) δ 8.06 (s, 1H), 7.48 (d, J = 8.70 Hz, 2H), 6.80 (d, J = 8.70 Hz, 2H), 6.38 (d, J = 16.94 Hz, 1H), 6.26 (dd, J_1= 16.94 Hz, J_1= 10.07 Hz, 1H), 5.67 (d, J = 9.62 Hz, 1H), 3.76 (s, 3H). ^{13}C NMR ($CDCl_3$, 100 MHz) δ 163.8, 156.6, 131.4, 131.1, 127.4, 122.1, 114.2, 55.6. ESI-MS: $[m+H]^+$ m/z 178.

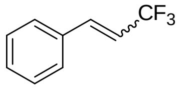

4a

(3,3,3-Trifluoroprop-1-enyl)benzene (**4a**) (related to **Figure 3**) (Kathiravan et al., 2015): Eluent: pentane, the solvent was removed at 0 ℃ under reduced pressure. Yield: 153.1 mg (89%) with **2c** as the reactant, 156.6 mg (91%) with **2d** as the reactant, E/Z = 6:4. Colorless oil. ^{1}H NMR ($CDCl_3$, 400 MHz) δ 7.44-7.31 (m, 5H), 7.14 (d, J = 16.49 Hz, 0.4H), 6.90 (d, J = 12.82 Hz, 0.6H), 6.23-6.14 (m, 0.4H), 5.79-5.69 (m, 0.6H). ^{19}F ($CDCl_3$, 376.5 MHz) δ -57.4, -61.2. ^{13}C NMR ($CDCl_3$, 100 MHz) δ 139.8 ($J_{F\text{-}C}$ = 5.75 Hz), 137.8 ($J_{F\text{-}C}$ = 6.71 Hz), 133.8, 133.5, 130.1, 129.2, 129.1, 129.07, 128.5, 127.7, 125.1, 124.3, 122.5, 121.6, 118.1 ($J_{F\text{-}C}$ = 34.50 Hz), 116.0 ($J_{F\text{-}C}$ = 33.55 Hz). EI-MS: M^+ m/z 172.

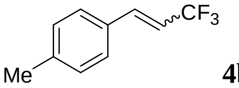

4b

(3,3,3-Trifluoroprop-1-enyl)benzene (**4b**) (related to **Figure 3**) (Kathiravan et al., 2015): Eluent: pentane, the solvent was removed at 0 ℃ under reduced pressure. Yield: 161.9 mg (87%) with **2c** as the reactant, 169.3 mg (91%) with **2d** as the reactant, E/Z = 7:3. Colorless oil. ^{1}H NMR ($CDCl_3$, 400 MHz) δ 7.53-7.50 (m, 2H), 7.39-7.35 (m, 2H), 7.30 (d, J = 16.03 Hz, 0.3H), 7.05 (d, J = 12.82 Hz, 0.7H), 6.38-6.29 (m, 0.3H), 6.93-5.83 (m, 0.7H), 2.55 (S, 3H). ^{19}F ($CDCl_3$, 376.5 MHz) δ -57.4, -63.0. ^{13}C NMR ($CDCl_3$, 100 MHz) δ 140.5, 139.8 ($J_{F\text{-}C}$ = 5.75 Hz), 139.4, 137.7 ($J_{F\text{-}C}$ = 6.71 Hz), 130.9, 130.8, 129.8, 129.2, 127.6, 125.3, 124.5, 122.6, 121.8, 117.1 ($J_{F\text{-}C}$ = 34.50 Hz), 114.9 ($J_{F\text{-}C}$ = 33.55 Hz), 21.44, 21.39. EI-MS: M^+ m/z 186.

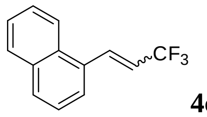

4c

1-(3,3,3-Trifluoroprop-1-enyl)naphthalene (**4c**) (related to **Figure 3**) (Kathiravan et al., 2015): Eluent: pentane. Yield: 197.6 mg (89%) with **2c** as the reactant, 202.1 mg (91%) with **2d** as the reactant. Colorless oil. ^{1}H NMR ($CDCl_3$, 400 MHz) δ 8.09 (d, J = 8.70 Hz, 1H), 8.00 (d, J = 16.03 Hz, 1H), 7.93 (d, J = 8.24 Hz, 2H), 7.66-7.58 (m, 3H), 7.51 (t, J = 7.79 Hz, 1H), 6.37-6.28 (m, 1H). ^{19}F ($CDCl_3$, 376.5 MHz) δ -63.1. ^{13}C NMR ($CDCl_3$, 100 MHz) δ 135.3 ($J_{F\text{-}C}$ = 6.71 Hz), 133.7, 131.2, 131.1, 130.3, 128.9, 127.0, 126.4, 125.5, 124.9, 123.3, 118.9 ($J_{F\text{-}C}$ = 33.55 Hz), 21.44, 21.39. EI-MS: M^+ m/z 222.

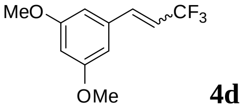

4d

1,3-Dimethoxy-5-(3,3,3-trifluoroprop-1-enyl)benzene (**4d**) (related to **Figure 3**) (Parsons et al., 2012): Eluent: pentane. Yield: 201.9 mg (87%) with **2c** as the reactant, 211.2 mg (91%) with **2d** as the reactant, E/Z = 57:43. Colorless oil. ^{1}H NMR ($CDCl_3$, 400 MHz) δ 7.09 (d, J = 16.03 Hz, 0.43H), 6.87 (d, J = 12.82 Hz, 0.57H), 6.60-6.57 (s, 2H), 6.50-6.48 (s, 1H), 6.24-6.15 (m, 0.43H), 5.82-5.72 (m, 0.57H), 3.81-3.80 (s, 6H). ^{19}F ($CDCl_3$, 376.5 MHz) δ -57.1, -63.2. ^{13}C NMR ($CDCl_3$, 100 MHz) δ 161.2, 160.7, 139.8 ($J_{F\text{-}C}$ = 5.75 Hz), 137.9 ($J_{F\text{-}C}$ = 6.71 Hz), 135.5, 135.4, 125.1, 124.2, 122.4, 121.5, 118.43 ($J_{F\text{-}C}$ = 34.50 Hz), 116.4 ($J_{F\text{-}C}$ = 33.55 Hz), 107.0, 105.6, 102.2, 101.4, 55.43, 55.4. EI-MS: M^+ m/z 232.

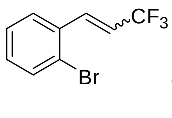

4e

1-Bromo-2-(3,3,3-trifluoroprop-1-enyl)benzene (**4e**) (related to **Figure 3**) (Hafner et al., 2011): Eluent: pentane. Yield: 177.6 mg (71%) with **2c** as the reactant, E/Z = 54:46. Colorless oil. ^{1}H NMR ($CDCl_3$, 400 MHz) δ 7.61-7.50 (m, 2H), 7.39-7.17 (m, 2.55H), 7.00 (d, *J* = 12.36 Hz, 0.57H), 6.19-6.10 (s, 0.54H), 5.92-5.82 (m, 0.46H). ^{19}F ($CDCl_3$, 376.5 MHz) δ -57.7, -63.5. ^{13}C NMR ($CDCl_3$, 100 MHz) δ 138.8 ($J_{F\text{-}C}$ = 5.75 Hz), 136.7 ($J_{F\text{-}C}$ = 6.71 Hz), 134.7, 133.7, 133.5, 132.5, 131.2, 130.5, 130.4, 130.36, 128.0, 127.7, 127.3, 124.8, 124.6, 124.0, 123.1, 122.0, 121.3, 120.0 ($J_{F\text{-}C}$ = 34.50 Hz), 118.7 ($J_{F\text{-}C}$ = 33.55 Hz). EI-MS: M^{+} m/z 250.

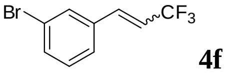

4f

1-Bromo-3-(3,3,3-trifluoroprop-1-enyl)benzene (**4f**) (related to **Figure 3**) (Abdukader et al., 2011): Eluent: pentane. Yield: 222.6 mg (89%) with **2c** as the reactant, 222.5 mg (89%) with **2d** as the reactant, E/Z = 75:25. Colorless oil. ^{1}H NMR ($CDCl_3$, 400 MHz) δ 7.59 (s, 0.25H), 7.50-7.47 (m, 1.75H), 7.37-7.31 (m, 1H), 7.27-7.21 (m, 1H), 7.07 (d, *J* = 16.03 Hz, 0.25H), 6.86 (d, J = 12.36 Hz, 0.75H), 6.24-6.16 (m, 0.25H), 5.86-5.76 (m, 0.75H). ^{19}F ($CDCl_3$, 376.5 MHz) δ -57.4, -63.2. ^{13}C NMR ($CDCl_3$, 100 MHz) δ 138.1, 136.3, 135.7, 135.5, 133.0, 132.0, 131.8, 130.5, 130.4, 129.9, 127.4, 126.6, 126.3, 123.9, 123.1, 122.4, 122.0, 121.2, 119.6 ($J_{F\text{-}C}$ = 34.50 Hz), 117.4 ($J_{F\text{-}C}$ = 33.55 Hz). EI-MS: M^{+} m/z 250.

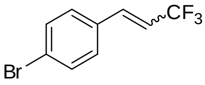

4g

1-Bromo-4-(3,3,3-trifluoroprop-1-enyl)benzene (**4g**) (related to **Figure 3**) (Kathiravan et al., 2015): Eluent: Pentane. Yield: 220 mg (88%) with **2c** as the reactant, 230.1 mg (92%) with **2d** as the reactant, E/Z = 66:34. Colorless oil. ^{1}H NMR ($CDCl_3$, 400 MHz) δ 7.52-7.47 (m, 2H), 7.31-7.24 (m, 2H), 7.01 (d, *J* = 16.03 Hz, 0.34H), 6.84 (d, J = 12.82 Hz, 0.66H), 6.23-6.14 (m, 0.34H), 5.84-5.74 (m, 0.66H). ^{19}F ($CDCl_3$, 376.5 MHz) δ -57.5, -63.3. ^{13}C NMR ($CDCl_3$, 100 MHz) δ 138.6 ($J_{F\text{-}C}$ = 5.75 Hz), 136.6 ($J_{F\text{-}C}$ = 6.71 Hz), 132.6, 132.4, 132.3, 131.7, 130.63. 130.61, 129.1, 124.4, 124.1, 123.5, 121.4. 118.9 ($J_{F\text{-}C}$ = 35.46 Hz), 116.6 ($J_{F\text{-}C}$ = 34.50 Hz). EI-MS: M^{+} m/z 250.

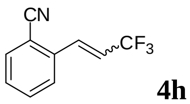

4h

2-(3,3,3-Trifluoroprop-1-enyl)benzonitrile (**4h**) (related to **Figure 3**) (Kathiravan et al., 2015): Eluent: pentane/CH_2Cl_2 5:1. Yield: 159.6 mg (81%) with **2c** as the reactant, 171.4 mg (87%) with

2d as the reactant, E/Z = 71:29. Colorless oil. ^{1}H NMR ($CDCl_3$, 400 MHz) δ 7.69-7.13 (m, 5H), 6.46-5.97 (m, 1H). ^{19}F ($CDCl_3$, 376.5 MHz) δ -58.0, -63.9. ^{13}C NMR ($CDCl_3$, 100 MHz) δ 137.3, 136.1, 135.5 (J_{F-C} = 4.79 Hz), 133.4, 133.2, 132.6, 132.5, 130.1, 129.33, 129.30, 129.2, 126.8, 125.6, 122.3 (J_{F-C} = 34.50 Hz), 120.8, 120.4 (J_{F-C} = 34.50 Hz), 117.0, 116.9, 112.4, 112.0. EI-MS: M^+ m/z 197.

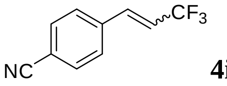

4i

4-(3,3,3-Trifluoroprop-1-enyl)benzonitrile (**4i**) (related to **Figure 3**) (Kathiravan et al., 2015): Eluent: pentane/CH_2Cl_2 5:1. Yield: 165.5 mg (84%) with **2c** as the reactant, 177.4 mg (90%) with **2d** as the reactant, E/Z = 71:29. Colorless oil. ^{1}H NMR ($CDCl_3$, 400 MHz) δ 7.69-7.63 (m, 5H), 7.56-7.45 (m, 2H), 7.15 (d, J = 16.03 Hz, 0.29H), 6.96 (d, J = 12.36 Hz, 0.71H), 6.36-6.27 (m, 0.29H), 5.97-5.87 (m, 0.71H). ^{19}F ($CDCl_3$, 376.5 MHz) δ -57.6, -63.7. ^{13}C NMR ($CDCl_3$, 100 MHz) δ 138.3, 137.7 (J_{F-C} = 5.75 Hz), 136.0 (J_{F-C} = 6.71 Hz), 132.8, 132.1, 129.4, 128.1, 123.7, 121.7, 121.0, 121.95 (J_{F-C} = 34.50 Hz), 119.4 (J_{F-C} = 34.50 Hz), 18.5, 118.3, 113.5, 112.6. EI-MS: M^+ m/z 197.

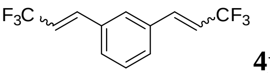

4j

1,3-Bis(3,3,3-trifluoroprop-1-enyl)benzene (**4j**) (related to **Figure 3**) (Prakash et al., 2015): Eluent: pentane. Yield: 215.5 mg (81%) with **2c** as the reactant, E/Z = 60:40. Colorless oil. ^{1}H NMR ($CDCl_3$, 400 MHz) δ 7.50-7.38 (m, 4H), 7.16-7.12 (m, 0.8H), 6.95-6.90 (m, 1.2H), 6.29-6.17 (m, 0.8H), 5.88-5.76 (m, 1.2H). ^{19}F ($CDCl_3$, 376.5 MHz) δ -57.4, -57.6, -63.4, -63.5. ^{13}C NMR ($CDCl_3$, 100 MHz) δ 139.1 (J_{F-C} = 5.75 Hz), 138.9(J_{F-C} = 5.75 Hz), 137.2 (J_{F-C} = 6.71 Hz), 137.0 (J_{F-C} = 6.71 Hz), 134.7, 134.4, 134.0, 133.7, 130.4, 129.8, 129.5, 129.2, 129.0, 128.6, 128.0, 126.9, 125.0, 124.9, 124.2, 122.3, 122.3, 121.5, 119.4 (J_{F-C} = 34.50 Hz), 119.0 (J_{F-C} = 34.50 Hz), 116.8 (J_{F-C} = 34.50 Hz). EI-MS: M^+ m/z 266.

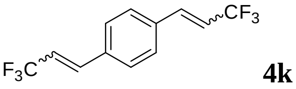

4k

1,4-Bis(3,3,3-trifluoroprop-1-enyl)benzene (**4k**) (related to **Figure 3**) (Satoru et al., 2015): Eluent: pentane. Yield: 236.8 mg (89%) with **2c** as the reactant, 144.7 mg (92%) with **2d** as the reactant, E/Z = 60:40. Colorless oil. ^{1}H NMR ($CDCl_3$, 400 MHz) δ 7.48-7.38 (m, 4H), 7.19-7.13 (m,

1.26H), 6.95-6.91 (m, 0.74H), 6.31-6.22 (m, 1.26H), 5.88-5.78 (m, 0.74H). ^{19}F ($CDCl_3$, 376.5 MHz) δ -51.7, -57.6, -63.5, -69.4. ^{13}C NMR ($CDCl_3$, 100 MHz) δ 139.0 (J_{F-C} = 5.75 Hz), 138.8 (J_{F-C} = 5.75 Hz), 136.9 (J_{F-C} = 6.71 Hz), 136.8 (J_{F-C} = 6.71 Hz), 135.4, 135.1, 134.4, 134.1, 129.7, 129.1, 128.2, 127.6, 125.0, 124.3, 122.3, 121.6, 119.6 (J_{F-C} = 34.50 Hz), 118.9 (J_{F-C} = 34.50 Hz), 117.0 (J_{F-C} = 34.50 Hz), 116.9 (J_{F-C} = 34.50 Hz). EI-MS: M^+ m/z 266.

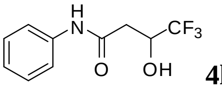

4l

4,4,4-Trifluoro-3-hydroxy-*N*-phenylbutanamide (**4l**) (related to **Figure 3**): Eluent: hexane/ethyl acetate 5:1. Yield: 216.7 mg (93%) with **2c** as the reactant. White solid, mp. 74-75 ℃. ^{1}H NMR (DMSO, 400 MHz) δ 10.1 (s, 1H), 7.62 (d, *J* = 7.33 Hz, 2H), 7.31 (t, *J* = 7.33 Hz, 2H), 7.05 (t, J = 7.79 Hz, 1H), 6.55 (d, *J* = 6.41 Hz, 1H), 4.49-4.41 (m, 1H), 2.70-2.59 (m, 2H). ^{19}F (DMSO, 376.5 MHz) δ -78.1. ^{13}C NMR (DMSO, 100 MHz) δ 167.1, 139.1, 128.8, 125.8 (J_{F-C} = 282.75 Hz), 123.4, 119.1, 66.1 (J_{F-C} = 30.67 Hz), 37.8. HRMS (ESI-TOF) calculated for $C_{10}H_9F_3NO_2$ [M-H]$^-$ m/z 232.0591, found 232.0589.

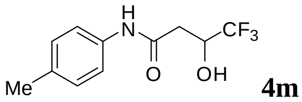

4m

4,4,4-Trifluoro-3-hydroxy-*N*-*p*-tolylbutanamide (**4m**) (related to **Figure 3**): Eluent: hexane/ethyl acetate 5:1. Yield: 234.7 mg (95%) with **2c** as the reactant. White solid, mp. 103-104 ℃. ^{1}H NMR (DMSO, 400 MHz) δ 10.0 (s, 1H), 7.51 (d, *J* = 8.70 Hz, 2H), 7.12 (t, *J* = 8.70 Hz, 2H), 6.54 (d, *J* = 6.41 Hz, 1H), 4.51-4.41 (m, 1H), 2.69-2.58 (m, 2H), 2.26 (s, 3H). ^{19}F (DMSO, 376.5 MHz) δ -78.1. ^{13}C NMR (DMSO, 100 MHz) δ 166.8, 136.6, 132.2, 129.2, 125.8 (J_{F-C} = 281.79 Hz), 119.1, 66.1 (J_{F-C} = 30.67 Hz), 37.7, 20.5. HRMS (ESI-TOF) calculated for $C_{11}H_{11}F_3NO_2$ [M-H]$^-$ m/z 246.0747, found 246.0746.

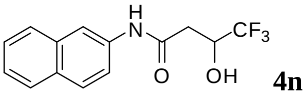

4n

4,4,4-Trifluoro-3-hydroxy-*N*-(naphthalen-2-yl)butanamide (**4n**) (related to **Figure 3**): Eluent: hexane/ethyl acetate 5:1. Yield: 254.8 mg (90%) with **2c** as the reactant. Light yellow solid, mp. 167-168 ℃. ^{1}H NMR (DMSO, 400 MHz) δ 10.3 (s, 1H), 8.35 (s, 1H), 7.88-7.81 (m, 3H), 7.61 (d, *J* = 8.70 Hz, 1H), 7.47 (t, *J* = 7.33 Hz, 1H), 7.40 (t, *J* = 7.79 Hz, 1H), 6.60 (d, *J* = 6.41 Hz, 1H), 4.57-4.47 (m, 1H), 2.78-2.68 (m, 2H). ^{19}F (DMSO, 376.5 MHz) δ -78.0. ^{13}C NMR (DMSO, 100

MHz) δ 167.9, 137.1, 133.9, 130.3, 128.9, 128.0, 127.8, 127.0, 126.3 ($J_{F\text{-}C}$ = 283.71 Hz), 125.2, 120.4, 115.7, 66.7 ($J_{F\text{-}C}$ = 30.67 Hz), 38.4. HRMS (ESI-TOF) calculated for $C_{14}H_{11}F_3NO_2$ $[M\text{-}H]^-$ m/z 282.0747, found 282.0742.

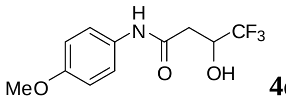

4o

4,4,4-Trifluoro-3-hydroxy-*N*-(naphthalen-2-yl)butanamide (**4o**) (related to **Figure 3**): Eluent: hexane/ethyl acetate 5:1. Yield: 249.9 mg (95%) with **2c** as the reactant. White solid, mp. 59-60 ℃. ^{1}H NMR (DMSO, 400 MHz) δ 9.97 (s, 1H), 7.51 (d, *J* = 8.70 Hz, 2H), 6.88 (d, *J* = 8.70 Hz, 2H), 6.51 (d, *J* = 6.41 Hz, 1H), 4.47-4.40 (m, 1H), 3.71 (s, 3H), 2.65-2.54 (m, 2H). ^{19}F (DMSO, 376.5 MHz) δ -78.1. ^{13}C NMR (DMSO, 100 MHz) δ 167.0, 155.8, 132.7, 126.3 ($J_{F\text{-}C}$ = 281.79 Hz), 121.1, 114.4, 66.6 ($J_{F\text{-}C}$ = 30.67 Hz), 55.6, 38.1. HRMS (ESI-TOF) calculated for $C_{11}H_{11}F_3NO_3$ $[M\text{-}H]^-$ m/z 262.0697, found 262.0698.

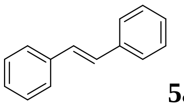

5a

(*E*)-1,2-Diphenylethene (**5a**) (related to **Figure 4**) (McNulty et al., 2009): Eluent: hexane. Yield: 163.9 mg (91%). Colorless oil. ^{1}H NMR ($CDCl_3$, 400 MHz) δ 7.23-7.15 (m, 10H), 6.59 (s, 2H). ^{13}C NMR ($CDCl_3$, 100 MHz) δ 137.4, 130.4, 129.0, 128.3, 127.2. EI-MS: M^+ m/z 180.

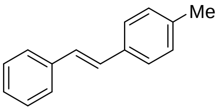

5b

(*E*)-1,2-Diphenylethene (**5b**) (related to **Figure 4**) (Huo et al., 2009): Eluent: hexane. Yield: 174.6 mg (90%). Colorless oil. ^{1}H NMR ($CDCl_3$, 400 MHz) δ 7.26-7.12 (m, 7H), 7.00 (d, *J* = 7.79 Hz, 2H), 6.54 (s, 2H), 2.29 (s, 3H). ^{13}C NMR ($CDCl_3$, 100 MHz) δ 137.6, 137.0, 134.4, 130.3, 129.7, 129.0, 128.97, 128.9, 128.3, 127.1, 21.3. EI-MS: M^+ m/z 194.

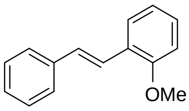

5c

(*E*)-1-Methoxy-2-styrylbenzene (**5c**) (related to **Figure 4**) (McNulty et al., 2009): Eluent: hexane. Yield: 187.0 mg (89%). Colorless oil. ^{1}H NMR ($CDCl_3$, 400 MHz) δ 7.23-7.11 (m, 7H), 6.85 (d, *J* = 8.24 Hz, 1H), 6.74-6.67 (m, 2H), 6.61 (d, *J* = 11.91 Hz, 1H), 3.77 (s, 3H). ^{13}C NMR ($CDCl_3$,

100 MHz) δ 157.3, 137.4, 130.3, 130.1, 128.9, 128.7, 128.1, 127.0, 126.3, 125.9, 120.3, 110.7, 55.5. EI-MS: M^+ m/z 210.

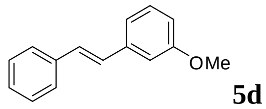

5d

(*E*)-1-Methoxy-3-styrylbenzene (**5d**) (related to **Figure 4**) (Roberts et al., 2004): Eluent: hexane. Yield: 191.2 mg (91%). Colorless oil. ^{1}H NMR ($CDCl_3$, 400 MHz) δ 7.27-7.10 (m, 6H), 6.84-6.72 (m, 3H), 6.63-6.53 (m, 2H), 3.63 (s, 3H). ^{13}C NMR ($CDCl_3$, 100 MHz) δ 159.5, 138.7, 137.4, 130.6, 130.3, 129.3, 129.0, 128.3, 127.3, 121.6, 113.9, 113.4, 55.1. EI-MS: M^+ m/z 210.

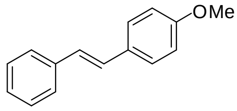

5e

(*E*)-1-Methoxy-4-styrylbenzene (**5e**) (related to **Figure 4**) (McNulty et al., 2009): Eluent: hexane. Yield: 195.4 mg (93%). Colorless solid, mp. 136-137 ℃. ^{1}H NMR ($CDCl_3$, 400 MHz) δ 7.28-7.16 (m, 7H), 6.74 (d, J = 8.60 Hz, 2H), 6.51 (s, 2H), 3.77 (s, 3H). ^{13}C NMR ($CDCl_3$, 100 MHz) δ 158.8, 137.7, 130.3, 129.9, 128.94, 128.88, 128.4, 127.0, 113.7, 55.3. EI-MS: M^+ m/z 210.

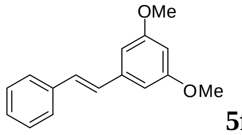

5f

(*E*)-1,3-Dimethoxy-5-styrylbenzene (**5f**) (related to **Figure 4**) (Roberts et al., 2004): Eluent: hexane. Yield: 220.9 mg (92%). Colorless solid, 129-130 ℃. ^{1}H NMR ($CDCl_3$, 400 MHz) δ 7.27-7.14 (m, 5H), 6.60 (d, J = 12.36 Hz, 1H), 6.51 (d, J = 11.91 Hz, 1H), 6.39 (s, 2H), 6.31 (s, 1H), 3.60 (s, 6H). ^{13}C NMR ($CDCl_3$, 100 MHz) δ 160.6, 139.1, 137.3, 130.8, 130.3, 129.0, 128.3, 127.3, 106.8, 100.0, 55.2. EI-MS: M^+ m/z 240.

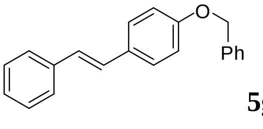

5g

(*E*)-1-(Benzyloxy)-4-styrylbenzene (**5g**) (related to **Figure 4**) (Richmond et al., 2015): Eluent: hexane. Yield: 266.0 mg (93%). Colorless solid, 32-33 ℃. ^{1}H NMR ($CDCl_3$, 400 MHz) δ 7.41-7.16 (m, 12H), 6.82 (d, J = 8.70 Hz, 2H), 6.51 (s, 2H), 5.02 (s, 2H). ^{13}C NMR ($CDCl_3$, 100 MHz) δ 158.0, 137.7, 137.1, 130.3, 130.0, 129.9, 128.9, 128.7, 128.4, 128.1, 127.6, 127.0, 114.6, 70.1. EI-MS: M^+ m/z 286.

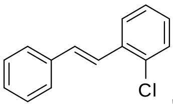

5h

(*E*)-1-Chloro-2-styrylbenzene (**5h**) (related to **Figure 4**) (Heynekamp et al., 2006): Eluent: hexane. Yield: 190.5 mg (89%). Colorless oil. ^{1}H NMR ($CDCl_3$, 400 MHz) δ 7.38 (d, *J* = 7.79 Hz, 1H), 7.20-7.11 (m, 7H), 7.01 (t, *J* = 7.79 Hz, 1H), 6.72-6.65 (m, 2H). ^{13}C NMR ($CDCl_3$, 100 MHz) δ 136.5, 136.1, 133.8, 131.8, 130.8, 129.6, 129.1, 128.6, 128.3, 127.5, 127.4, 126.5. EI-MS: M^+ m/z 214.

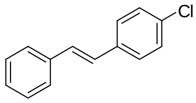

5i

(*E*)-1-Chloro-4-styrylbenzene (**5i**) (related to **Figure 4**) (McNulty et al., 2009): Eluent: hexane. Yield: 199.0 mg (93%). Colorless oil. ^{1}H NMR ($CDCl_3$, 400 MHz) δ 7.26-7.13 (m, 9H), 7.20-7.11 (m, 7H), 6.62 (d, *J* = 12.04 Hz, 1H), 6.51 (d, *J* = 12.04 Hz, 1H). ^{13}C NMR ($CDCl_3$, 100 MHz) δ 137.0, 135.8, 132.9, 131.1, 130.3, 129.0, 128.9, 128.53, 128.47, 127.4. EI-MS: M^+ m/z 214.

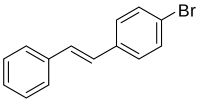

5j

(*E*)-1-Bromo-4-styrylbenzene (**5j**) (related to **Figure 4**) (McNulty et al., 2009): Eluent: hexane. Yield: 234.8 mg (91%). Colorless oil. ^{1}H NMR ($CDCl_3$, 400 MHz) δ 7.33 (d, *J* = 7.57 Hz, 2H), 7.25-7.17 (m, 5H), 7.09 (d, *J* = 7.57 Hz, 2H), 6.62 (d, *J* = 11.70 Hz, 1H), 6.49 (d, *J* = 12.38 Hz, 1H). ^{13}C NMR ($CDCl_3$, 100 MHz) δ 136.9, 136.2, 131.5, 131.1, 130.7, 129.1, 128.9, 128.5, 127.5, 121.1. EI-MS: M^+ m/z 258.

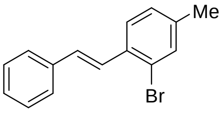

5k

(*E*)-2-Bromo-4-methyl-1-styrylbenzene (**5k**) (related to **Figure 4**) (McNulty et al., 2009): Eluent: hexane. Yield: 244.9 mg (90%). Colorless oil. ^{1}H NMR ($CDCl_3$, 300 MHz) δ 7.40 (s, 1H), 7.14 (s, 5H), 7.04 (d, *J* = 7.79 Hz, 1H), 6.85 (d, *J* = 7.79 Hz, 1H), 6.62 (d, *J* = 11.91 Hz, 1H), 6.57(d, *J* = 12.36 Hz, 1H), 2.26 (s, 3H). ^{13}C NMR ($CDCl_3$, 75 MHz) δ 139.0, 136.6, 134.9, 133.2, 131.1, 130.6, 129.5, 129.1, 128.2, 128.0, 127.3, 123.8, 20.9 EI-MS: M^+ m/z 272.

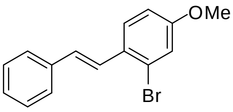

5l

(*E*)-2-Bromo-4-methoxy-1-styrylbenzene (**5l**) (related to **Figure 4**) (McNulty et al., 2009): Eluent: hexane. Yield: 265.0 mg (92%). Colorless oil. ^{1}H NMR ($CDCl_3$, 300 MHz) δ 7.23-7.05 (m, 7H), 6.65-6.53 (m, 3H), 3.76 (s, 3H). ^{13}C NMR ($CDCl_3$, 75 MHz) δ 159.3, 136.8, 131.4, 130.7, 130.1, 129.1, 129.07, 128.3, 127.3, 124.4. 117.6, 113.6, 55.6. HRMS (ESI-TOF) calculated for $C_{15}H_{11}BrF$ $[M+H]^+$ m/z 289.0228, found 289.0230.

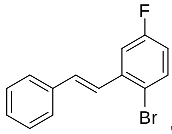

5m

(*E*)-1-Bromo-4-fluoro-2-styrylbenzene (**5m**) (related to **Figure 4**): Eluent: hexane. Yield: 240.2 mg (87%). Colorless oil. ^{1}H NMR ($CDCl_3$, 400 MHz) δ 7.53-7.50 (m, 1H), 7.21-7.12 (m, 5H), 6.88 (d, J = 9.16 Hz, 1H), 6.79 (t, J = 8.24 Hz, 1H), 6.71 (d, J = 11.91 Hz, 1H), 6.54 (d, J = 12.36 Hz, 1H). ^{19}F ($CDCl_3$, 376.5 MHz) δ -114.8. ^{13}C NMR ($CDCl_3$, 100 MHz) δ 162.9 ($J_{F\text{-}C}$ = 247.28 Hz), 139.8 ($J_{F\text{-}C}$ = 7.67 Hz), 135.9, 134.0 ($J_{F\text{-}C}$ = 8.63 Hz), 132.5, 129.0, 128.52, 128.47, 127.8, 118.3, 117.8, 117.6, 116.2, 116.0. HRMS (ESI-TOF) calculated for $C_{14}H_{14}BrF$ $[M+H]^+$ m/z 277.0028, found 277.0026.

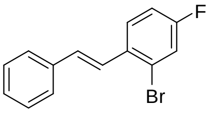

5n

(*E*)-2-Bromo-4-fluoro-1-styrylbenzene (**5n**) (related to **Figure 4**): Eluent: hexane. Yield: 240.1 mg (87%). Colorless oil. ^{1}H NMR ($CDCl_3$, 400 MHz) δ 7.32 (d, J = 8.24 Hz, 1H), 7.24-7.09 (m, 6H), 6.77 (d, J = 8.24 Hz, 1H), 6.66 (d, J = 12.36 Hz, 1H), 6.53 (d, J = 11.91 Hz, 1H). ^{19}F ($CDCl_3$, 376.5 MHz) δ -112.4. ^{13}C NMR ($CDCl_3$, 100 MHz) δ 161.5 ($J_{F\text{-}C}$ = 251.1 Hz), 136.2, 134.1($J_{F\text{-}C}$ = 3.83 Hz), 131.9, 131.8, 129.0, 128.98, 128.5, 128.4, 128.3, 127.5, 124.1 ($J_{F\text{-}C}$ = 9.58 Hz), 119.9 ($J_{F\text{-}C}$ = 24.92 Hz), 114.6 ($J_{F\text{-}C}$ = 21.09 Hz). HRMS (ESI-TOF) calculated for $C_{14}H_{14}BrF$ $[M+H]^+$ m/z 277.0028, found 277.0031.

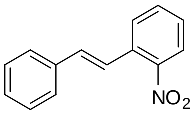

5o

(*E*)-1-Nitro-2-styrylbenzene (**5o**) (related to **Figure 4**) (Roberts et al., 2004): Eluent: hexane. Yield: 189 mg (84%). Yellow solid, mp. 72-73 ℃. ^{1}H NMR ($CDCl_3$, 400 MHz) δ 8.08-8.04 (m, 1H), 7.37-7.34 (m, 2H), 7.26-7.22 (m, 1H), 7.16-7.13 (m, 3H), 7.06-7.03 (m, 2H), 6.88 (d, J = 11.91 Hz,

1H), 6.74 (d, *J* = 11.91 Hz, 1H). ^{13}C NMR ($CDCl_3$, 100 MHz) δ 148.2, 135.9, 133.7, 133.1, 132.3, 131.9, 129.2, 129.0, 128.3, 128.2, 127.6, 127.2, 126.5, 124.7. EI-MS: M^+ m/z 225.

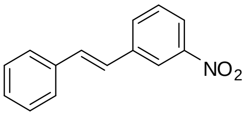

5p

(*E*)-1-Nitro-3-styrylbenzene (**5p**) (related to **Figure 4**) (Nobuaki et al., 2001): Eluent: hexane. Yield: 182.3 mg (81%). Yellow Solid, mp. 106-107 ℃. ^{1}H NMR ($CDCl_3$, 400 MHz) δδ 8.25 (s, 1H), 8.06 (d, *J* = 8.24 Hz, 1H), 7.68 (d, *J* = 7.79 Hz, 1H), 7.49 (t, *J* = 7.79 Hz, 1H), 6.93-6.86 (m, 1H), 6.61-6.48 (m, 2H), 5.45 (d, *J* = 16.94 Hz, 1H), 5.30 (d, *J* = 10.07 Hz, 1H). ^{13}C NMR ($CDCl_3$, 100 MHz) δ 148.4, 139.0, 136.2, 135.0, 133.2, 129.2, 129.0, 128.8, 128.7, 128.0, 127.8, 126.9, 123.9, 122.0. EI-MS: M^+ m/z 225.

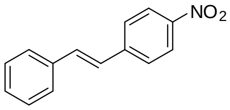

5q

(*E*)-1-Nitro-4-styrylbenzene (**5q**) (related to **Figure 4**) (McNulty et al., 2009): Eluent: hexane. Yield: 184.6 mg (82%). Yellow solid, mp.156-157 ℃. ^{1}H NMR ($CDCl_3$, 400 MHz) δ 8.19 (d, *J* = 8.70 Hz, 2H), 7.60 (d, *J* = 9.16 Hz, 2H), 7.53 (d, *J* = 7.33 Hz, 2H), 7.39 (t, *J* = 7.79 Hz, 2H), 7.32 (t, *J* = 7.33 Hz, 1H), 7.25 (d, *J* = 16.94 Hz, 1H), 7.12 (d, *J* = 16.49 Hz, 1H). ^{13}C NMR ($CDCl_3$, 100 MHz) δ 146.8, 143.9, 136.3, 133.4, 129.0, 128.9, 127.1, 126.9, 126.3, 124.2. EI-MS: M^+ m/z 225.

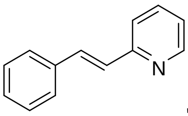

5r

(*E*)-2-Styrylpyridine (**5r**) (related to **Figure 4**) (Heynekamp et al., 2004): Eluent: hexane/ethyl acetate 5:1. Yield: 153.9 mg (85%). Light yellow oil. ^{1}H NMR ($CDCl_3$, 300 MHz) δ 8.57 (d, *J* = 5.04 Hz, 1H), 7.65-7.56 (m, 1H), 7.42-7.34 (m, 2H), 7.30-7.20 (m, 3H), 7.13 (d, *J* = 10.53 Hz, 1H), 7.05 (t, *J* = 6.41 Hz, 1H), 6.82 (d, *J* = 12.36 Hz, 1H), 6.69 (d, *J* = 12.36 Hz, 1H). ^{13}C NMR ($CDCl_3$, 75 MHz) δ 156.4, 149.6, 136.7, 135.6, 133.3, 130.5, 128.9, 128.8, 128.3, 127.6, 127.1, 123.9, 121.8. ESI-MS: $[M+H]^+$ m/z 182.

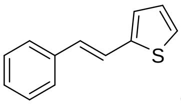

5s

(*E*)-2-Styrylthiophene (**5s**) (related to **Figure 4**) (Yang et al., 2012): Eluent: hexane/ethyl acetate 50:1. Yield: 153.9 mg (82%). Yellow oil. ^{1}H NMR ($CDCl_3$, 300 MHz) δ 7.37-7.17 (m, 5H), 7.07 (d, *J* = 5.16 Hz, 1H), 6.96 (d, *J* = 3.78 Hz, 1H), 6.87 (t, *J* = 3.78 Hz, 1H), 6.69 (d, *J* = 12.04 Hz,

1H), 6.57 (d, J = 11.70 Hz, 1H). ^{13}C NMR ($CDCl_3$, 75 MHz) δ 139.9, 137.5, 129.0, 128.95, 128.7, 128.3, 128.28, 127.6, 127.2, 126.6, 125.7, 123.5. EI-MS: M^+ m/z 186.

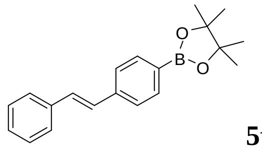

5t

(*E*)-4,4,5,5-Tetramethyl-2-(4-styrylphenyl)-1,3,2-dioxaborolane (**5t**) (related to **Figure 4**) (Li et al., 2015): Eluent: hexane/ethyl acetate 20:1. Yield: 153.9 mg (82%). White solid, mp. 123-124 ℃. ^{1}H NMR ($CDCl_3$, 400 MHz) δ 7.67 (d, J = 8.24 Hz, 2H), 7.26-7.15 (m, 7H), 6.96 (d, J = 3.78 Hz, 1H), 6.63-6.56 (m, 2H), 1.32 (s, 12H). ^{11}B ($CDCl_3$, 102.7 MHz) 29.7. ^{13}C NMR ($CDCl_3$, 100 MHz) δ 140.3, 137.1, 134.8, 131.0, 130.3, 129.0, 128.31, 128.27, 127.3, 126.0, 83.8, 25.0. EI-MS: M^+ m/z 291.

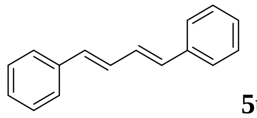

5u

(*1E,3E*)-1,4-Diphenylbuta-1,3-diene (**5u**) (related to **Figure 4**) (Huo et al., 2009): Eluent: hexane. Yield: 162.8 mg (79%). Colorless oil. ^{1}H NMR ($CDCl_3$, 400 MHz) δ 7.46 (d, J = 7.33 Hz, 4H), 7.35 (t, J = 7.33 Hz, 4H), 7.24 (t, J = 8.24 Hz, 2H), 7.01-6.93 (m, 2H), 7.72-6.65 (m, 2H). ^{13}C NMR ($CDCl_3$, 100 MHz) δ 137.5, 133.0, 129.4, 128.8, 127.7, 126.5. EI-MS: M^+ m/z 206.

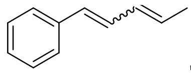

5v

Penta-1,3-dienylbenzene (**5v**) (related to **Figure 4**) (Anton et al., 2012): Eluent: pentane. Yield: 119.6 mg (83%), E/Z = 1:1. Colorless oil. ^{1}H NMR ($CDCl_3$, 400 MHz) δ 7.42-7.16 (m, 5H), 6.77-5.55 (m, 4H), 1.87-1.78 (m, 3H). ^{13}C NMR ($CDCl_3$, 100 MHz) δ 138.0, 137.8, 137.7, 132.7, 132.0, 130.6, 130.4, 129.9, 129.7, 129.5, 129.2, 129.04, 129.0, 128.7, 128.66, 128.31, 128.26, 128.0, 127.5, 127.3, 127.2, 127.0, 126.8, 126.4, 126.2, 125.8, 125.2, 124.3, 18.53, 18.49. EI-MS: M^+ m/z 144.

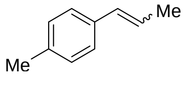

5w

1-Methyl-4-(prop-1-enyl)benzene (**5w**) (related to **Figure 4**) (Monfredini et al., 2012): Eluent: pentane. Yield: 104.3 mg (79%), E/Z = 45:55. Colorless oil. ^{1}H NMR ($CDCl_3$, 400 MHz) δ 7.22-7.07 (m, 4H), 6.41-6.34 (m, 1H), 6.21-6.12 (m, 0.45H), 5.78-5.69 (m, 0.55H), 2.33-2.31 (s,

3H), 1.90-1.84 (m, 3H). ^{13}C NMR ($CDCl_3$, 100 MHz) δ 136.5, 136.2, 135.3, 134.9, 131.0, 129.8, 129.3, 128.9, 128.87, 126.1, 125.8, 124.7, 21.3, 18.6, 14.8. EI-MS: M^+ m/z 132.

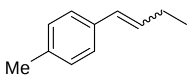

5x

1-Methyl-4-(prop-1-enyl)benzene (**5x**) (related to **Figure 4**) (Monfredini et al., 2012): Eluent: pentane. Yield: 116.9 mg (80%), E/Z = 31: 69. Colorless oil. ^{1}H NMR ($CDCl_3$, 400 MHz) δ 7.23-7.07 (m, 4H), 6.35-6.32 (m, 1H), 6.23-6.16 (m, 0.31H), 5.62-5.56 (m, 0.69H), 2.37-2.17 (m, 5H), 1.09-1.03 (m, 3H). ^{13}C NMR ($CDCl_3$, 100 MHz) δ 136.5, 136.2, 135.3, 135.0, 134.2, 131.7, 129.3, 128.9, 128.8, 128.2, 125.9, 26.2, 22.1, 21.3, 21.2, 14.6, 13.9. EI-MS: M^+ m/z 146.

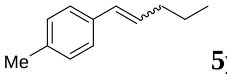

5y

1-Methyl-4-(pent-1-enyl)benzene (**5y**) (related to **Figure 4**) (McNulty et al., 2009): Eluent: pentane. Yield: 129.7 mg (81%), E/Z = 45:55. Colorless oil. ^{1}H NMR ($CDCl_3$, 400 MHz) δ 7.24-7.07 (m, 4H), 6.39-6.32 (m, 1H), 6.19-6.12 (m, 0.55H), 5.64-5.58 (m, 0.45H), 2.33-2.13 (m, 5H), 1.53-1.42 (m, 2H), 0.96-0.91 (m, 3H). ^{13}C NMR ($CDCl_3$, 100 MHz) δ 136.5, 136.2, 135.3, 135.1, 132.5, 130.0, 129.8, 129.3, 128.9, 128.8, 125.9, 35.2, 30.9, 23.3, 22.7, 21.3, 21.2, 14.0, 13.9. EI-MS: M^+ m/z 160.

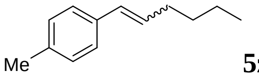

5z

1-(Hex-1-enyl)-4-methylbenzene (**5z**) (related to **Figure 4**) (Andrews et al., 2013): Eluent: pentane. Yield: 142.7 mg (82%), E/Z = 50:50. Colorless oil. ^{1}H NMR ($CDCl_3$, 400 MHz) δ 7.24-7.06 (m, 4H), 6.38-6.32 (m, 1H), 6.20-6.12 (m, 0.5H), 5.64-5.58 (m, 0. 5H), 2.35-2.16 (m, 5H), 1.48-1.32 (m, 4H), 0.94-0.87 (m, 3H). ^{13}C NMR ($CDCl_3$, 100 MHz) δ 136.5, 136.1, 135.3, 135.1, 132.6, 130.3, 129.6, 129.3, 128.9, 128.8, 128.6, 125.9, 32.8, 32.3, 31.7, 28.5, 22.6, 22.4, 21.2, 14.1. EI-MS: M^+ m/z 174.

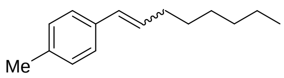

5aa

1-Methyl-4-(oct-1-enyl)benzene (**5aa**) (related to **Figure 4**) (Andrews et al., 2013): Eluent: hexane. Yield: 176.6 mg (91%), E/Z = 65:35. Colorless oil. ^{1}H NMR ($CDCl_3$, 400 MHz) δ 7.35-7.18 (m, 10H), 6.58 (d, J = 11.45 Hz, 0.65H), 6.44 (d, J = 16.03 Hz, 0.35H), 6.38-6.31 (m, 0. 35H), 5.88-5.81 (m, 0.65H), 3.68-3.52 (m, 2H). ^{13}C NMR ($CDCl_3$, 100 MHz) δ 140.9, 140.3, 137.6,

137.4, 131.2, 130.8, 130.2, 129.4, 129.0, 128.9, 128.8, 128.68, 128.66, 128.5, 128.42, 128.37, 127.27, 127.0, 126.3, 126.24, 126.20, 39.5, 34.8. EI-MS: M^+ m/z 194.

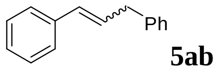

5ab

Prop-1-ene-1,3-diyldibenzene (**5ab**) (related to **Figure 4**) (Andrews et al., 2013): Eluent: hexane. Yield: 175.8 mg (91%), E/Z = 32:68. Colorless oil. ^{1}H NMR ($CDCl_3$, 400 MHz) δ 7.22-7.05 (m, 4H), 6.37-6.30 (m, 1H), 6.18-6.11 (m, 0.32H), 5.63-5.56 (m, 0. 68H), 2.33-2.14 (m, 5H), 1.48-1.27 (m, 8H), 0.90-0.85 (m, 3H). ^{13}C NMR ($CDCl_3$, 100 MHz) δ 136.5, 136.1, 135.3, 135.1, 132.6, 130.2, 129.7, 129.3, 128.9, 128.8, 128.7, 125.9, 33.2, 31.9, 30.2, 29.6, 29.2, 29.1, 28.7, 22.8, 21.2, 14.2. EI-MS: M^+ m/z 202.

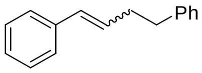

5ac

But-1-ene-1,4-diyldibenzene (**5ac**) (related to **Figure 4**) (Huo et al., 2009): Eluent: hexane. Yield: 181.0 mg (87%), E/Z = 44:56. Colorless oil. ^{1}H NMR ($CDCl_3$, 400 MHz) δ 7.46-7.32 (m, 10H), 6.58-6.52 (m, 1H), 6.41-6.34 (m, 0.56H), 5.86-5.79 (m, 0. 44H), 2.90 (q, J = 7.79 Hz, 2H), 2.78 (q, J = 7.33 Hz, 1H), 2.65 (q, J = 7.33 Hz, 1H). ^{13}C NMR ($CDCl_3$, 100 MHz) δ 141.9, 141.8, 137.8, 137.7, 131.9, 130.5, 130.0, 129.5, 129.0, 128.8, 128.6, 128.5, 128.3, 127.1, 126.7, 126.1, 126.03, 126.0, 36.2, 36.0, 35.0, 30.5. EI-MS: M^+ m/z 208.

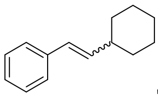

5ad

(2-Cyclohexylvinyl)benzene (**5ad**) (related to **Figure 4**) (McMahon et al., 2013): Eluent: hexane. Yield: 167.5 mg (90%), E/Z = 75:25. Colorless oil. ^{1}H NMR ($CDCl_3$, 300 MHz) δ 7.35-7.15 (m, 5H), 6.37-6.29 (m, 1H), 6.21-6.13 (m, 0.75H), 5.50 (t, J = 10.32Hz, 0.25H), 2.64-2.08 (m, 1H), 1.82-1.67 (m, 5H), 1.38-1.11 (m, 5H). ^{13}C NMR ($CDCl_3$, 75 MHz) δ 139.1, 138.2, 137.0, 128.7, 128.6, 128.3, 127.3, 126.9, 126.8, 126.5, 126.0, 41.3, 37.0, 33.4, 33.1, 26.3, 26.2, 25.8. EI-MS: M^+ m/z 186.

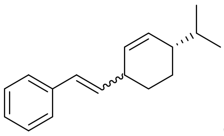

5ae

(*S*)-(2-(4-Isopropylcyclohex-2-enyl)vinyl)benzene (**5ae**) (related to **Figure 4**): Eluent: hexane. Yield: 185.4 mg (82%). Colorless oil. ^{1}H NMR ($CDCl_3$, 300 MHz) δ 7.30-7.16 (m, 6H), 6.33 (d, J

= 12.36Hz, 1H), 6.09 (d, *J* = 12.36Hz, 1H), 5.77 (d, *J* = 3.66 Hz, 1H), 4.70 (d, *J* = 8.24 Hz, 2H), 2.33-1.90 (m, 6H), 1.75-1.69 (m, 4H), 1.43-1.21 (m, 1H). ^{13}C NMR ($CDCl_3$, 75 MHz) δ 149.9, 138.7, 135.4, 133.3, 129.0, 128.7, 127.9, 127.7, 126.6, 108.7, 40.8, 31.3, 28.6, 27.9, 20.9. HRMS (ESI-TOF) calculated for $C_{17}H_{23}$ [M+H]$^+$ m/z 227.1800, found 227.1797.

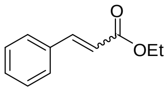

5af

Ethyl 3-phenylacrylate (**5af**) (McNulty et al., 2009) (related to **Figure 4**): Eluent: hexane. Yield: 125.0 mg (71%), E/Z = 45:55. Colorless oil. ^{1}H NMR ($CDCl_3$, 300 MHz) δ 7.68 (d, *J* = 16.03 Hz, 0.54H), 7.58-7.48 (m, 2H), 7.36-7.30 (m, 3H), 6.92 (d, *J* = 12.36Hz, 0.45H), 6.43 (d, *J* = 16.03 Hz, 0.55H), 5.93 (d, *J* = 12.36Hz, 0.45H), 4.28-4.13 (m, 2H), 1.34-1.21 (m, 3H). ^{13}C NMR ($CDCl_3$, 75 MHz) δ 167.0, 166.2, 144.6, 143.0, 134.9, 134.5, 130.2, 129.7, 129.0, 128.9, 128.1, 128.0, 119.9, 118.3, 60.5, 60.3, 14.4, 14.1. EI-MS: M$^+$ m/z 176.

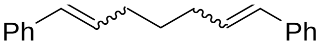

5ag

1,7-Diphenylhepta-1,6-diene (**5ag**) (related to **Figure 4**) (Mojr et al., 2013): Eluent: hexane. Yield: 108.0 mg (87%), EE/ZZ = 1:1. Colorless oil. ^{1}H NMR ($CDCl_3$, 400 MHz) δ 7.34-7.16 (m, 10H), 6.45-6.31 (m, 2H), 6.26-6.13 (m, 1H), 5.70-5.60 (m, 1H), 2.41-2.19 (m, 4H), 1.66-1.58 (m, 2H). ^{13}C NMR ($CDCl_3$, 100 MHz) δ 137.9, 137.8, 132.7, 132.6, 130.7, 130.6, 130.3, 129.3, 129.27, 128.9, 128.6, 128.56, 128.24, 127.0, 126.9, 126.6, 126.1, 32.6, 30.4, 29.7, 29.1, 28.5, 28.1. EI-MS: M$^+$ m/z 248.

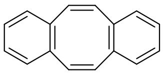

5ah

Dibenzo[*a*,*e*]cyclooctene (**5ah**) (related to **Figure 4**) (Esser et al., 2009): Eluent: Hexane. Yield: 63 mg (62%). White solid, mp. 106-108 ℃. ^{1}H NMR ($CDCl_3$, 400 MHz) δ 7.19-7.16 (m, 4H), 7.11-7.08 (m, 4H), 6.79 (s, 4H). ^{13}C NMR ($CDCl_3$, 100 MHz) δ 137.2, 133.4, 129.2, 127.0. EI-MS: M$^+$ m/z 204.

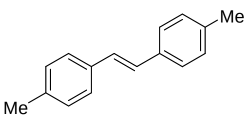

5ai

(E)-1,2-dip-tolylethene (**5ai**) (related to **Figure 5**) (Yuen et al., 2016): Eluent: hexane. Yield: 189.3 mg (91%). Colorless liquid. ^{1}H NMR ($CDCl_3$, 400 MHz) δ 7.15 (d, *J* = 8.24 Hz, 4H), 7.01 (d, *J* =

8.24 Hz, 4H), 6.50 (s, 2H), 2.29 (s, 6H). ^{13}C NMR ($CDCl_3$, 100 MHz) δ 136.8, 134.6, 129.6, 129.0, 128.9, 21.4. EI-MS: M^+ m/z 208.

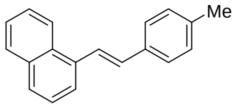

5aj

((*E*)-1-(4-Methylstyryl)naphthalene (**5aj**) (related to **Figure 5**): Eluent: hexane. Yield: 219.7 mg (90%). Colorless solid, mp. 78-79 ℃. ^{1}H NMR ($CDCl_3$, 400 MHz) δ 8.20 (s, 1H), 7.96 (d, *J* = 6.87 Hz, 1H), 7.86 (d, *J* = 6.87 Hz, 1H), 7.61-7.57 (m, 2H), 7.51-7.42 (m, 2H), 7.11-7.08 (m, 3H), 7.00 (d, *J* = 7.79 Hz, 2H), 6.90 (d, *J* = 12.36 Hz, 1H), 2.32 (s, 3H). ^{13}C NMR ($CDCl_3$, 100 MHz) δ 137.0, 135.7, 134.0, 133.8, 132.0, 131.7, 129.1, 128.9, 128.5, 127.7, 127.5, 126.5, 126.1, 126.0, 125.7, 125.1, 21.3. HRMS (ESI-TOF) calculated for $C_{19}H_{17}$ $[M+H]^+$ m/z 245.1330, found 245.1332.

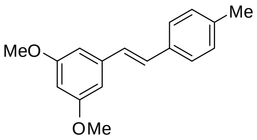

5ak

(*E*)-1,3-Dimethoxy-5-(4-methylstyryl)benzene (**5ak**) (related to **Figure 5**) (Chen et al., 2016): Eluent: hexane/ethyl acetate 20:1. Yield: 236.3 mg (93%). Colorless solid, mp. 62-63 ℃. ^{1}H NMR ($CDCl_3$, 400 MHz) δ 7.16 (d, *J* = 7.79 Hz, 2H), 7.00 (d, *J* = 7.79 Hz, 2H), 6.54 (d, *J* = 12.36 Hz, 1H), 6.45 (d, *J* = 12.36 Hz, 1H), 6.41 (s, 2H), 6.30 (s, 1H), 3.61 (s, 6H), 2.27 (s, 3H). ^{13}C NMR ($CDCl_3$, 100 MHz) δ 160.6, 139.4, 137.0, 134.2, 130.7, 129.6, 128.93, 128.9, 106.7, 99.7, 55.1, 21.2. EI-MS: M^+ m/z 254.

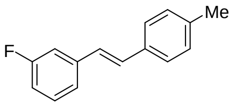

5al

(*E*)-1-Fluoro-3-(4-methylstyryl)benzene (**5al**) (related to **Figure 5**) (Yuen et al., 2016): Eluent: hexane. Yield: 193.0 mg (91%). Colorless liquid. ^{1}H NMR ($CDCl_3$, 400 MHz) δ 7.21 (m, 3H), 7.03-7.00 (m, 3H), 6.94 (d, *J* = 10.07 Hz, 1H), 6.68 (d, *J* = 8.70 Hz, 1H), 6.59 (d, *J* = 12.36 Hz, 1H), 6.48 (d, *J* = 12.36 Hz, 1H), 2.30 (s, 3H). ^{19}F ($CDCl_3$, 376.5 MHz) -113.4. ^{13}C NMR ($CDCl_3$, 100 MHz) δ 162.8 (J_{F-C} = 244.41 Hz), 139.9 (J_{F-C} = 7.67 Hz), 137.4, 133.9, 131.5, 129.8 (J_{F-C} = 8.63 Hz), 129.2, 128.9, 128.4, 115.6 (J_{F-C} = 21.09 Hz), 114.0 (J_{F-C} = 21.09 Hz), 21.4. EI-MS: M^+ m/z 212.

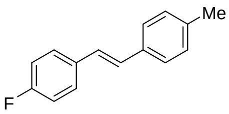

5am

(*E*)-1-Fluoro-4-(4-methylstyryl)benzene (**5am**) (related to **Figure 5**) (Yuen et al., 2016): Eluent: hexane. Yield: 186.6 mg (88%). Colorless liquid. ^{1}H NMR ($CDCl_3$, 400 MHz) δ 7.23-7.19 (m, 2H), 7.11 (d, *J* = 7.33 Hz, 2H), 6.92-6.87 (m, 2H), 6.54 (d, *J* = 11.91 Hz, 1H), 6.48 (d, *J* = 11.91 Hz, 1H), 2.30 (s, 3H). ^{19}F ($CDCl_3$, 376.5 MHz) -114.7. ^{13}C NMR ($CDCl_3$, 100 MHz) δ 161.9 ($J_{F\text{-}C}$ = 246.33 Hz), 137.1, 134.2, 133.6, 130.6 ($J_{F\text{-}C}$ = 7.67 Hz), 130.4, 129.1, 128.9, 128.5, 115.2 ($J_{F\text{-}C}$ = 21.09 Hz), 21.4. EI-MS: M^+ m/z 212.

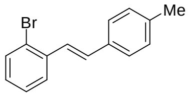

5an

(*E*)-1-Bromo-2-(4-methylstyryl)benzene (**5an**) (related to **Figure 5**) (Yuen et al., 2016): Eluent: hexane. Yield: 243.0 mg (89%). Colorless liquid. ^{1}H NMR ($CDCl_3$, 400 MHz) δ 7.58 (d, *J* = 9.16 Hz, 1H), 7.20 (d, *J* = 9.62 Hz, 1H), 7.09-6.96 (m, 6H), 6.64 (d, *J* = 11.91 Hz, 1H), 6.55 (d, *J* = 12.36 Hz, 1H), 2.27 (s, 3H). ^{13}C NMR ($CDCl_3$, 100 MHz) δ 138.3, 137.3, 133.5, 132.8, 131.4, 131.0, 129.1, 129.0, 128.8, 128.7, 127.1, 124.0, 21.4. EI-MS: M^+ m/z 273.

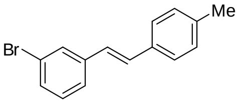

5ao

(*E*)-1-Bromo-3-(4-methylstyryl)benzene (**5ao**) (related to **Figure 5**) (Iwasaki et al., 2014): Eluent: hexane. Yield: 245.8 mg (90%). Colorless liquid. ^{1}H NMR ($CDCl_3$, 400 MHz) δ 7.46 (s, 1H), 7.35 (d, *J* = 8.24 Hz, 1H), 7.23-7.07 (m, 6H), 6.64 (d, *J* = 12.36 Hz, 1H), 6.50 (d, *J* = 11.91 Hz, 1H), 2.36 (s, 3H). ^{13}C NMR ($CDCl_3$, 100 MHz) δ 139.8, 137.4, 133.7, 131.8, 131.7, 130.0, 129.8, 129.1, 128.9, 128.0, 127.5, 122.4, 21.4. EI-MS: M^+ m/z 273.

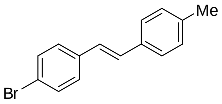

5ap

(*E*)-1-Bromo-4-(4-methylstyryl)benzene (**5ap**) (related to **Figure 5**) (Babudri et al., 2000): Eluent: hexane. Yield: 245.7 mg (90%). Colorless liquid. ^{1}H NMR ($CDCl_3$, 400 MHz) δ 7.39 (d, *J* = 8.70 Hz, 2H), 7.17 (d, *J* = 7.79 Hz, 4H), 7.09 (d, *J* = 7.79 Hz, 2H), 6.64 (d, *J* = 11.91 Hz, 1H), 6.50 (d, *J* = 11.91 Hz, 1H), 2.37 (s, 3H). ^{13}C NMR ($CDCl_3$, 100 MHz) δ 137.3, 136.5, 134.0, 131.4, 131.1, 130.6, 129.2, 128.8, 128.4, 120.9, 21.4. EI-MS: M^+ m/z 273.

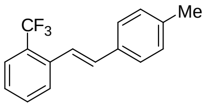

5aq

(*E*)-1-(4-Methylstyryl)-2-(trifluoromethyl)benzene (**5aq**) (related to **Figure 5**): Eluent: hexane. Yield: 235.9 mg (90%). Colorless liquid. ^{1}H NMR ($CDCl_3$, 400 MHz) δ 7.69-7.67 (m, 1H), 7.31-7.22 (m, 3H), 6.94 (s, 4H), 6.76 (d, J = 11.91 Hz, 1H), 6.67 (d, J = 12.36 Hz, 1H), 2.26 (s, 3H). ^{19}F ($CDCl_3$, 376.5 MHz) -61.0. ^{13}C NMR ($CDCl_3$, 100 MHz) δ 137.3, 137.1, 133.4, 132.2, 131.6, 131.4, 129.2, 129.0 ($J_{F\text{-}C}$ = 29.71 Hz), 128.96, 127.1, 126.0 ($J_{F\text{-}C}$ = 4.79 Hz), 123.1, 21.3. HRMS (ESI-TOF) calculated for $C_{16}H_{12}F_3$ $[M\text{-}H]^-$ m/z 261.0891, found 261.0894.

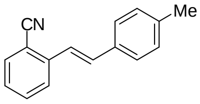

5ar

(*E*)-2-(4-Methylstyryl)benzonitrile (**5ar**) (related to **Figure 5**) (Yang et al., 2016): Eluent: hexane/ethyl acetate 20:1. Yield: 197.2 mg (90%). White Soild, mp. 147-148 ℃. ^{1}H NMR ($CDCl_3$, 400 MHz) δ 7.62 (d, J = 7.79 Hz, 1H), 7.37-7.35 (m, 2H), 7.30-7.26 (m, 1H), 7.04-6.99 (m, 4H), 6.80 (d, J = 11.91 Hz, 1H), 6.70 (d, J = 12.36 Hz, 1H), 2.29 (s, 3H). ^{13}C NMR ($CDCl_3$, 100 MHz) δ 141.6, 137.8, 134.4, 133.1, 133.0, 132.2, 129.8, 129.2, 128.9, 127.4, 125.2, 118.0, 112.3, 21.3. EI-MS: M^+ m/z 219.

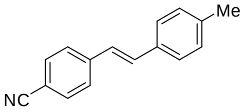

5as

(*E*)-4-(4-Methylstyryl)benzonitrile (**5as**) (related to **Figure 5**) (Kita et al., 2010): Eluent: hexane/ethyl acetate 20:1. Yield: 201.5 mg (92%). White Soild, mp. 178-179 ℃. ^{1}H NMR ($CDCl_3$, 400 MHz) δ 7.50 (d, J = 8.24 Hz, 2H), 7.34 (d, J = 8.24 Hz, 2H), 7.10 (d, J = 8.24 Hz, 2H), 7.06 (d, J = 8.24 Hz, 2H), 6.73 (d, J = 11.91 Hz, 1H), 6.52 (d, J = 12.36 Hz, 1H), 2.33 (s, 3H). ^{13}C NMR ($CDCl_3$, 100 MHz) δ 142.4, 137.9, 133.4, 132.1, 129.6, 129.3, 128.8, 127.8, 119.1, 110.4, 21.4. EI-MS: M^+ m/z 219.

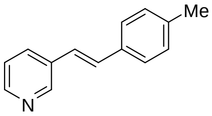

5at

(*E*)-4-(4-Methylstyryl)benzonitrile (**5at**) (related to **Figure 5**) (Hepburn et al., 2016): Eluent: hexane/ethyl acetate 10:1. Yield: 174.5 mg (89%). Light yellow oil. ^{1}H NMR ($CDCl_3$, 400 MHz) δ 8.49 (s, 1H), 8.41 (d, J = 5.04 Hz, 1H), 7.53 (d, J = 7.79 Hz, 1H), 7.13-7.04 (m, 5H), 6.71 (d, J = 11.91 Hz, 1H), 6.49 (d, J = 11.91 Hz, 1H), 2.31 (s, 3H). ^{13}C NMR ($CDCl_3$, 100 MHz) δ 150.3,

148.1, 137.6, 135.9, 133.7, 133.3, 132.8, 129.3, 128.7, 125.8, 123.0, 21.3. ESI-MS: $[M+H]^+$ m/z 196.

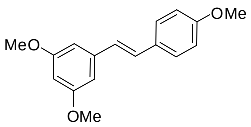

5au

(*E*)-1,3-Dimethoxy-5-(4-methoxystyryl)benzene (**5au**) (related to **Figure 5**) (Roberts et al., 2004): Eluent: hexane/ethyl acetate 20:1. Yield: 248.5 mg (92%). Colorless solid, mp. 55-56 ℃. ^{1}H NMR ($CDCl_3$, 400 MHz) δ 7.25 (d, J = 8.70 Hz, 2H), 6.80 (d, J = 8.70 Hz, 2H), 6.56 (d, J = 12.36 Hz, 1H), 6.49-6.46 (m, 3H), 6.36 (s, 1H), 3.78 (s, 3H), 3.68 (s, 6H). ^{13}C NMR ($CDCl_3$, 100 MHz) δ 160.6, 158.8, 139.5, 130.3, 130.2, 129.6, 128.7, 113.5, 106.5, 106.6, 99.7, 55.2. EI-MS: M^+ m/z 270.

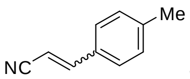

5av

3-*p*-Tolylacrylonitrile (**5av**) (related to **Figure 5**) (Rokade et al., 2012): Eluent: hexane/ethyl acetate 30:1. Yield: 127.3 mg (89%), E/Z 44:56. Colorless liquid. ^{1}H NMR ($CDCl_3$, 400 MHz) δ 7.70 (d, J = 8.24 Hz, 0.87H), 7.35-7.31 (m, 1.67H), 7.25-7.18 (m, 2H), 7.07 (d, J = 11.91 Hz, 0.44H), 5.79 (d, J = 16.94 Hz, 0.56H), 5.36 (d, J = 12.36 Hz, 0.44H), 2.38-2.27 (s, 3H). ^{13}C NMR ($CDCl_3$, 100 MHz) δ 150.5, 148.6, 141.9, 141.6, 131.0, 130.9, 129.8, 129.6, 129.1, 127.4, 118.5, 117.7, 95.0, 93.7, 21.6, 21.5. EI-MS: M^+ m/z 143.

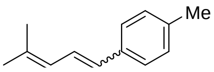

5aw

1-Methyl-4-(3-methylbuta-1,3-dienyl)benzene (**5aw**) (related to **Figure 5**) (Lishchynskyi et al., 2008): Eluent: pentane. Yield: 142.8 mg (83%), E/Z 77:23. Colorless liquid. ^{1}H NMR ($CDCl_3$, 400 MHz) δ 7.42-7.36 (m, 2H), 7.26-7.20 (m, 2H), 7.11-6.47 (m, 2H), 6.43-6.10 (m, 1H), 2.45-2.44 (s, 3H), 1.97-1.94 (m, 6H). ^{13}C NMR ($CDCl_3$, 100 MHz) δ 137.8, 136.7, 136.3, 135.7, 135.4, 135.2, 129.7, 129.3, 129.1, 128.9, 127.1, 126.1, 126.0, 125.8, 124.8, 121.7, 26.4, 26.3, 21.2, 18.6, 18.4. EI-MS: M^+ m/z 172.

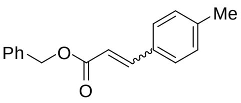

5ax

Benzyl 3-*p*-tolylacrylate (**5ax**) (Wang et al., 2014) (related to **Figure 5**): Eluent: hexane/ethyl acetate 20:1. Yield: 202.0 mg (80%), E/Z 50:50. Colorless oil. ^{1}H NMR ($CDCl_3$, 400 MHz) δ 7.75

(d, J = 16.03 Hz, 0.5H), 7.55 (d, J = 8.24 Hz, 1H), 7.46-7.36 (m, 6H), 7.21-7.15 (m, 2H), 6.95 (d, J = 12.82 Hz, 0.5H), 6.48 (d, J = 16.03 Hz, 0.5H), 5.97 (d, J = 12.82 Hz, 0.5H), 5.28-5.20 (s, 2H), 2.39-2.38 (s, 3H). ^{13}C NMR ($CDCl_3$, 100 MHz) δ 167.1, 166.2, 145.3, 144.0, 140.9, 139.5, 136.3, 136.0, 132.1, 131.8, 130.1, 129.8, 128.9, 128.7, 128.6, 128.5, 128.4, 128.36, 128.3, 128.26, 118.6, 116.9, 66.4, 66.2, 21.6, 21.5. EI-MS: M^+ m/z 252.

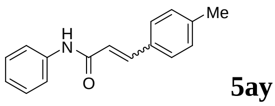

5ay

N-Phenyl-3-*p*-tolylacrylamide (**5ay**) (related to **Figure 5**) (Qiu et al., 2013): Eluent: hexane/ethyl acetate 5:1. Yield: 196.8 mg (83%), E/Z 65:35. White solid, mp. 187-188 ℃. ^{1}H NMR (DMSO, 400 MHz) δ 10.23 (s, 1H), 7.75-6.14 (m, 11H), 2.32-2.28 (s, 3H). ^{13}C NMR (DMSO, 100 MHz) δ 164.6, 163.7, 140.1, 139.6, 139.4, 139.2, 137.8, 132.4, 132.0, 129.9, 129.6, 128.8, 128.7, 128.6, 127.7, 123.3, 123.26, 121.3, 119.4, 119.2, 21.0, 20.9. ESI-MS: $[M+H]^+$ m/z 238.

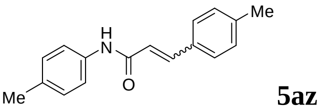

5az

N,3-Dip-tolylacrylamide (**5az**) (related to **Figure 5**) (Qiu et al., 2013): Eluent: hexane/ethyl acetate 5:1. Yield: 204.0 mg (81%), E/Z 60:40. White solid, mp. 192-193 ℃. ^{1}H NMR (DMSO, 400 MHz) δ 10.11 (s, 1H), 7.64-6.13 (m, 10H), 2.32-2.25 (s, 6H). ^{13}C NMR (DMSO, 100 MHz) δ 164.4, 163.5, 139.9, 139.5, 138.2, 137.6, 136.9, 136.7, 132.4, 132.3, 132.2, 132.1, 129.9, 129.6, 129.2, 129.1, 128.6, 127.7, 123.4, 121.4, 119.4, 119.2, 21.0, 20.9, 20.5. ESI-MS: $[M+H]^+$ m/z 252.

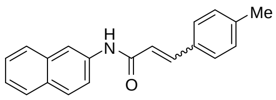

5ba

N-Phenyl-3-*p*-tolylacrylamide (**5ba**) (related to **Figure 5**) (Rajitha et al., 2015): Eluent: hexane/ethyl acetate 5:1. Yield: 218.2 mg (76%), E/Z 68:32. White solid, mp. 237-239℃. ^{1}H NMR (DMSO, 400 MHz) δ 10.48 (s, 1H), 8.47-8.45 (s, 1H), 7.87-6.22 (m, 12H), 2.32-2.28 (s, 3H). ^{13}C NMR (DMSO, 100 MHz) δ 164.8, 164.0, 140.3, 139.7, 138.3, 137.0, 133.5, 132.4, 132.0, 130.0, 129.8, 129.6, 128.6, 128.4, 128.3, 127.8, 127.5, 127.4, 126.4, 124.6, 123.2, 121.3, 120.1, 120.0, 115.4, 115.3, 21.0, 20.9. ESI-MS: $[M+H]^+$ m/z 288.

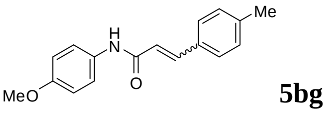

5bg

N-(4-Methoxyphenyl)-3-*p*-tolylacrylamide (**5bb**) (related to **Figure 5**): Eluent: hexane/ethyl acetate 5:1. Yield: 211.0 mg (79%), E/Z 68:32. White solid, mp. 201-202 ℃. ^{1}H NMR (DMSO, 400 MHz) δ 10.07 (s, 1H), 7.65-6.10 (m, 10H), 3.73-3.72 (s, 3H), 2.32-2.28 (s, 3H). ^{13}C NMR (DMSO, 100 MHz) δ 164.2, 163.3, 155.32, 155.3, 139.6, 139.5, 138.1, 137.3, 132.6, 132.4, 132.3, 132.1, 129.9, 129.6, 128.6, 127.6, 123.5, 121.4, 120.9, 120.7, 113.94, 113.86, 55.1, 21.0, 20.9. HRMS (ESI-TOF) calculated for $C_{17}H_{17}NNaO$ $[M+Na]^+$ m/z 290.1157, found 290.1156.

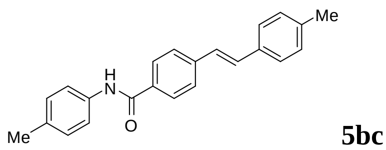

5bc

(*E*)-4-(4-Methylstyryl)-*N*-*p*-tolylbenzamide (**5bc**) (related to **Figure 5**): Eluent: hexane/ethyl acetate 5:1. Yield: 294.4 mg (90%). White solid, mp. 245-246 ℃. ^{1}H NMR ($CDCl_3$, 400 MHz) δ 8.06 (s, 1H), 7.71 (d, *J* = 8.70 Hz, 2H), 7.51 (d, *J* = 8.24 Hz, 2H), 7.32 (d, *J* = 8.24 Hz, 2H), 7.15-7.13 (m, 4H), 7.05 (d, *J* = 7.79 Hz, 2H), 6.68 (d, *J* = 12.36 Hz, 1H), 6.55(d, *J* = 12.36 Hz, 1H), 2.33 (s, 6H). ^{13}C NMR ($CDCl_3$, 100 MHz) δ 165.6, 141.2, 137.4, 135.5, 134.2, 133.8, 133.3, 132.0, 129.6, 129.1, 128.8, 128.5, 127.1, 120.5, 21.3, 21.0. HRMS (ESI-TOF) calculated for $C_{23}H_{21}NNaO$ $[M+Na]^+$ m/z 350.1521, found 350.1520.

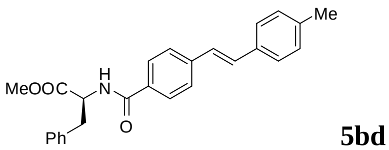

5bd

(*S,E*)-Methyl 2-(4-(4-methylstyryl)benzamido)-3-phenylpropanoate (**5bd**) (related to **Figure 5**): Eluent: hexane/ethyl acetate 5:1. Yield: 305.2 mg (90%). White solid, mp. 154-155 ℃. ^{1}H NMR ($CDCl_3$, 400 MHz) δ 7.58 (d, *J* = 8.24 Hz, 2H), 7.30-7.22 (m, 5H), 7.14-7.09 (m, 4H), 7.03 (d, *J* = 7.79 Hz, 2H), 7.15-7.13 (m, 4H), 6.64 (d, *J* = 12.36 Hz, 1H), 6.58 (d, *J* = 7.33 Hz, 1H), 6.52 (d, *J* = 12.36 Hz, 1H), 5.07 (dd, J_1 = 13.05 Hz, J_2 = 5.95 Hz, 1H), 3.75 (s, 3H), 3.30-3.18 (m, 2H), 2.31 (s, 3H). ^{13}C NMR ($CDCl_3$, 100 MHz) δ 172.2, 166.7, 141.3, 137.4, 136.0, 132.2, 132.1, 129.4, 129.2, 128.9, 128.7, 128.5, 127.3, 127.1, 53.6, 52.5, 38.0, 21.3. HRMS (ESI-TOF) calculated for $C_{26}H_{25}NNaO_3$ $[M+Na]^+$ m/z 422.1732, found 422.1734.

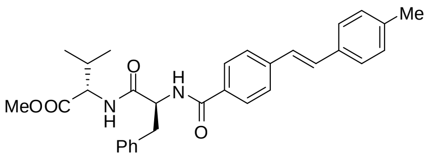

5be

(*S*)-Methyl 3-methyl-2-((*S*)-2-(4-(4-methylstyryl)benzamido)-3-phenylpropanamido)butanoate (**5be**) (related to **Figure 5**): Eluent: hexane/ethyl acetate 2:1. Yield: 423.4 mg (85%). White solid. ^{1}H NMR ($CDCl_3$, 400 MHz) δ 7.61 (d, *J* = 8.24 Hz, 2H), 7.47-7.39 (m, 1H), 7.23-7.15 (m, 8H), 7.09 (d, *J* = 8.24, 2H), 7.01 (d, *J* = 8.24, 2H), 6.62(d, *J* = 12.55 Hz, 1H), 6.50 (d, *J* = 12.36 Hz, 1H), 5.20-5.11(m, 1H), 4.48-4.44 (m, 1H), 3.66 (s, 3H), 3.17 (d, *J* = 7.33 Hz, 2H), 2.29 (s, 3H), 2.13-2.03 (m, 1H), 0.83-0.79 (m, 6H). ^{13}C NMR ($CDCl_3$, 100 MHz) δ 171.8, 171.7, 167.1, 141.1, 137.3, 136.8, 133.8, 132.0, 131.9, 129.5, 129.1, 128.9, 128.8, 128.4, 127.3, 126.8, 57.6, 55.0, 52.1, 52.0, 38.4, 31.0, 21.3, 19.0, 17.9. HRMS (ESI-TOF) calculated for $C_{31}H_{34}N_2NaO_4$ $[M+Na]^+$ m/z 521.2411, found 521.2409.

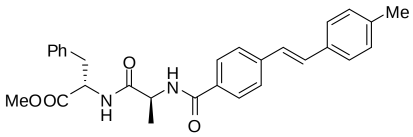

5bf

(*S*)-Methyl 2-((*S*)-2-(4-(4-methylstyryl)benzamido)propanamido)-3-phenylpropanoate (**5bf**) (related to **Figure 5**): Eluent: hexane/ethyl acetate 2:1. Yield: 418.4 mg (89%). White solid. ^{1}H NMR ($CDCl_3$, 400 MHz) δ 7.63 (d, *J* = 8.24 Hz, 2H), 7.39 (d, *J* = 8.24 Hz, 1H), 7.27 (d, *J* = 8.24 Hz, 2H), 7.13-7.00 (m, 10H), 6.64 (d, *J* = 12.55 Hz, 1H), 6.53 (d, *J* = 12.36 Hz, 1H), 4.86-4.79 (m, 2H), 3.68 (s, 3H), 3.14-2.98 (m, 3H), 2.29 (s, 3H), 1.44 (d, *J* = 7.33 Hz, 3H). ^{13}C NMR ($CDCl_3$, 100 MHz) δ 172.4, 171.8, 166.7, 141.2, 137.3, 135.9, 133.7, 131.9, 129.2, 129.0, 128.9, 128.8, 128.5, 127.2, 126.9, 53.5, 52.3, 48.9, 37.8, 21.2, 18.5. HRMS (ESI-TOF) calculated for $C_{29}H_{30}N_2NaO_4$ $[M+Na]^+$ m/z 493.2098, found 493.2102.

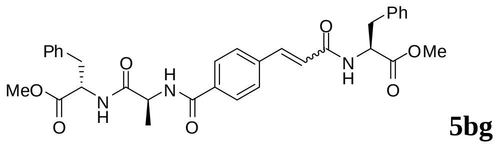

5bg

(*S*)-Methyl

2-(3-(4-((*S*)-1-((*S*)-1-methoxy-1-oxo-3-phenylpropan-2-ylamino)-1-oxopropan-2-ylcarbamoyl)phenyl)acrylamido)-3-phenylpropanoate (**5bg**) (related to **Figure 5**): Eluent: CH_2Cl_2/MeOH 20:1. Yield: 267 mg (91%), E/Z 66:34. White solid. ^{1}H NMR (DMSO, 400 MHz) δ 8.77-8.65 (m, 1H), 8.55-8.50 (m, 1H), 8.36-8.33 (m, 1H), 7.92-7.76 (m, 2H), 7.66-7.60 (m, 1H), 7.56 (d, *J* = 8.70 Hz,

2H), 7.47-6.07 (m, 12H), 4.65-4.45 (m, 3H), 3.63 (s, 3H), 3.58 (s, 3H), 3.12-2.90 (m, 4H), 1.31 (d, J = 7.33 Hz, 3H). ^{13}C NMR (DMSO, 100 MHz) δ 173.1, 173.0, 172.6, 172.5, 172.4, 166.1, 165.9, 165.3, 139.1, 138.4, 137.9, 137.8, 137.7, 137.6, 136.9, 135.1, 133.9, 133.7, 132.6, 132.1, 132.0, 130.0, 129.8, 129.7, 129.6, 129.4, 129.2, 128.84, 128.79, 128.77, 128.69, 127.9, 127.5, 127.1, 125.2, 123.5, 54.3, 53.2, 54.1, 54.5, 52.4, 49.1, 37.3, 37.1, 18.2. HRMS (ESI-TOF) calculated for $C_{33}H_{35}N_3NaO_7$ [M+Na]$^+$ m/z 608.2367, found 608.2369.

SUPPLEMENTAL REFERENCES

Alberti, A., and Macciantelli, D. (2009). Electron Paramagnetic Resonance 2009, Chapter 8, Spin Traps, Wiley-VCH: Weinheim.

Andrews, P., Latham, C. M., Magre, M., Willcox, D., and Woodward, S. (2013). $ZrCl_2(\eta\text{-}C_5Me_5)_2$-$AlHCl_2$ (THF)$_2$: efficient hydroalumination of terminal alkynes and cross-coupling of the derived alanes. Chem. Commun. *49*, 1488-1490.

Anton, L., and Kilian, M. (2012). An approach to the regioselective diamination of conjugated di-and trienes. Chem. Eur. J. *18*, 2212-2216.

Babudri, F., Farinola, G. M., Naso, F., and Panessa, D. (2000). A novel regio-and stereoselective formal cross-coupling reaction of unsaturated silanes with arenediazonium tetrafluoroborates. J. Org. Chem. *65*, 1554-1557.

Bunik, V. I., and Sievers, C. (2002). Inactivation of the 2−oxo acid dehydrogenase complexes upon generation of intrinsic radical species. Eur. J. Biochem. *269*, 5004-5015.

Cao, H., Chen, T., Zhou, Y., Han, D., Yin, S. F., and Han, L. B. (2014). Copper-catalyzed selective semihydrogenation of terminal alkynes with hypophosphorous acid. Adv. Synth. Catal. *356*, 765-769.

Chen, G., Wei, J., Yang, X., and Yao, Z. (2016). Convenient one-step synthesis of benzo[c]phenanthridines by three-component reactions of isochromenylium tetrafluoroborates and stilbenes in acetonitrile. Org. Lett. *18*, 1502-1505.

Dikalov, S. I., and Mason, R. P. (2001). Spin trapping of polyunsaturated fatty acid-derived peroxyl radicals: reassignment to alkoxyl radical adducts. Free Rad. Biol. Med. *30*, 187-197.

Eriksson, J., Åberg, O., and Långström, B. (2007). Synthesis of [^{11}C]/[^{13}C]acrylamides by palladium‐mediated carbonylation. Eur. J. Org. Chem. *3*, 455-461.

Esser, B., Bandyopadhyay, A., Rominger, F., and Gleiter, R. (2009). From metacyclophanes to cyclacenes: synthesis and properties of [6.8]$_3$ cyclacene. Chem. Eur. J. *15*, 3368-3379.

Gärtner, D., Stein, A. L., Grupe, S., Arp, J., and Wangelin, A. J. (2015). Heteroatom-free arene-cobalt and arene-iron catalysts for hydrogenations. Angew. Chem., Int. Ed. *54*, 10545-10549.

Hafner, A., and Bräse, S. (2011). Efficient trifluoromethylation of activated and non-activated alkenyl halides by using (trifluoromethyl)trimethylsilane. Adv. Synth. Catal. *353*, 3044-3048.

Hepburn, H. B., and Melchiorre, P. (2016). Brønsted acid-catalysed conjugate addition of photochemically generated α-amino radicals to alkenylpyridines. Chem. Commun. *52*, 3520-3523.

Heynekamp, J. J., Weber, W. M., Hunsaker, L. A., Gonzales, A. M., Orlando, R. A., Deck, L. M., and Van der Jagt, D. L. (2006). Substituted trans-stilbenes, including analogues of the natural product resveratrol, inhibit the human tumor necrosis factor alpha-induced activation of transcription factor nuclear factor kappa B. J. Med. Chem. *49*, 7182-7189.

Huo, C., He, X., and Chan, T. H. (2008). Zwitterionic phosphonium sulfonates as easily phase-separable ion-tagged Wittig reagents. J. Org. Chem. *73*, 8583-8586.

Iwasaki, T., Miyata, Y., Akimoto, R., Fujii, Y., Kuniyasu, H., and Kambe, N. (2014). Diarylrhodates as promising active catalysts for the arylation of vinyl ethers with Grignard reagents. J. Am. Chem. Soc. *136*, 9260-9263.

Kathiravan, S., and Nicholls, I. A. (2015). Palladium catalyzed vinyltrifluoromethylation of aryl halides through decarboxylative cross-coupling with 2-(trifluoromethyl) acrylic acid. Org. Lett. *17*, 1874-1877.

Kita, Y., Tobisu, M., and Chatani, N. (2010). Rhodium-catalyzed alkenylation of nitriles via silicon-assisted C−CN bond cleavage. Org. Lett. *12*, 1864-1867.

Li, X., Wang, A., Yu, K., Qi, Z., Chen, C., Wang, W., Hu, C., Wu, J., & Zhao, Z. (2015). Discovery of (*R*)-1-(3-(4-Amino-3-(4-phenoxyphenyl)-1*H*-pyrazolo[3,4-d]pyrimidin-1-yl)piperidin-1-yl)-2-(dimethylamino)ethanone (CHMFL-FLT3-122) as a potent and orally available FLT3 kinase inhibitor for FLT3-ITD positive acute myeloid leukemia. J. Med. Chem. *58*, 9625-9638.

Lishchynskyi, A., and Muňiz, K. (2012). An approach to the regioselective diamination of conjugated di-and trienes. Chem. Eur. J. *18*, 2212-2216.

McNulty, J., and Das, P. (2009). Highly stereoselective and general synthesis of (E) ‐ stilbenes and alkenes by means of an aqueous wittig reaction. Eur. J. Org. Chem. *24*, 4031-4035.

Monfredini, A., Santacroce, V., Deyris, P., Maggi, R., Bigi, F., Maestri, G., and Malacria, M. (2016). Boosting catalyst activity in cis-selective semi-reduction of internal alkynes by tailoring the assembly of all-metal aromatic tri-palladium complexes. Dalton Transactions *45*, 15786-15790.

McMahon, C. M., and Alexanian, E. J. (2014). Palladium-catalyzed Heck-type cross-couplings of unactivated alkyl iodides. Angew. Chem. Int. Ed. *53,* 5974-5977.

Mojr, V., Svobodová, E., Straková, K., Neveselý, T., Chudoba, J., Dvořáková, H., and Cibulka, R. (2015). Tailoring flavins for visible light photocatalysis: organocatalytic [2+2] cycloadditions mediated by a flavin derivative and visible light. Chem. Commun. *51*, 12036-12039.

Mundal, D. A., Lutz, K. E., and Thomson, R. J. (2009). Stereoselective synthesis of dienes from N-allylhydrazones. Org. Lett. *11*, 465-468.

Nobuaki, N., Hiroshi, O., Chizuru, S., and Hitomi, S. (2001). The reaction of peroxynitrite with organic molecules bearing a biologically important functionality. the multiplicity of reaction modes as exemplified by hydroxylation, nitration, nitrosation, dealkylation, oxygenation, and oxidative dimerization and cleavage. Bull. Chem. Soc. Jpn. *74,* 2385-2395.

Parsons, A. T., Senecal, T. D., and Buchwald, S. L. (2012). Iron (II)-catalyzed trifluoromethylation of potassium vinyltrifluoroborates. Angew. Chem., Int. Ed. *51*, 2947-2950.

Pogosyan G. M. (1979). Khimiya Nepredel’nykh Soedinenii, *1*, 159-179.

Poulsen, S. A., and Bornaghi, L. F. (2006). Fragment-based drug discovery of carbonic anhydrase II inhibitors by dynamic combinatorial chemistry utilizing alkene cross metathesis. Bioorg. Med. Chem. *14*, 3275-3284.

Prakash, G. K. S., Krishnan, H. S., Jog, P. V., Iyer, A. P., and Olah, G. A.(2012). A domino approach of Heck coupling for the synthesis of β-trifluoromethylstyrenes. Org. Lett. *14,* 1146-1149.

Qiu, J., and Zhang, R. (2013). DDQ-promoted direct transformation of benzyl hydrocarbons to amides via tandem reaction of the CDC reaction and Beckmann rearrangement. Org. Biomol. Chem. *11*, 6008-6012.

Rajitha, G., Priya, S. C., and Latha, T. Y. (2015). Synthesis and biological evaluation of β-amino naphthyl substituted chalcones for anti-inflammatory and antioxidant activities. J. Chem. Phar. Res. *7*, 80-84.

Richmond, E., and Moran, J. (2015). Selectivity in nickel-catalyzed transfer hydrogenative alkyne semireduction. J. Org. Chem. *80*, 6922-6929.

Roberts, J. C., and Pincock, J. A. (2004). The photochemical addition of 2, 2, 2-trifluoroethanol to methoxy-substituted stilbenes. J. Org. Chem. *69*, 4279-4282.

Rokade, B. V., Malekar, S. K., and Prabhu, K. R. (2012). A novel oxidative transformation of alcohols to nitriles: an efficient utility of azides as a nitrogen source. Chem. Commun. *48*, 5506-5508.

Satoru, F., Manabu, K., and Tamejiro, H. (1999). A facile synthesis of trifluoromethyl- and 3,3,3-trifluoropropenyl-substituted aromatic compounds by the oxidative desulfurization-fluorination of the corresponding carbodithioates. Bull. Chem. Soc. Jpn. *72*, 805-819.

Slinker, J. D., Gorodetsky, A . A., Lowry, M. S., Wang, J., Parker, S., Rohl, R., Bernhard, S., and Malliaras, G. G. (2004). Efficient yellow electroluminescence from a single layer of a cyclometalated iridium complex. J. Am. Chem. Soc. *126*, 2763-2767.

Sprouse, S., King, K. A., Spellane, P. J., and Watts, R. J. (1984). Photophysical effects of metal-carbon. sigma. bonds in ortho-metalated complexes of iridium (III) and rhodium (III). J. Am. Chem. Soc. *106*, 6647-6653.

Tamayo, A. B., Alleyne, B. D., Djurovich, P. I., Lamansky, S., Tsyba, I., Ho, N. N., Bau, R., and Thompson, M. E. (2003). Synthesis and characterization of facial and meridional tris-cyclometalated iridium(III) complexes. J. Am. Chem. Soc. *125*, 7377-7387.

Tanaka, S., Matsumoto, M., Goseki, R., Ishizone, T., and Hirao, A. (2013). Living anionic polymerization of 1, 4-divinylbenzene and its isomers. Macromolecules *46*, 146-154.

Wang, L., Wang, Y., Liu, C., and Lei, A. (2014). CO/C-H as an acylating reagent: a palladium-catalyzed aerobic oxidative carbonylative esterification of alcohols. Angew. Chem. Int. Ed. *53*, 5657-5661.

Wang, Z., Pitteloud, J. P., Montes, L., Rapp, M., Derane, D., and Wnuk, S. F. (2008). Vinyl tris (trimethylsilyl) silanes: substrates for Hiyama coupling. Tetrahedron *64*, 5322-5327.

Wienhöfer, G., Westerhaus, F. A., Jagadeesh, R. V., Junge, K., Junge, H., and Beller M. (2012). Selective iron-catalyzed transfer hydrogenation of terminal alkynes. Chem. Commun. *48*, 4827-4829.

Xu, H., Kovi, K. E., and Wolf C. (2008). Palladium-phosphinous acid-catalyzed cross-coupling of aryl and acyl halides with aryl-, alkyl-, and vinylzinc reagents. J. Org. Chem. *73*, 7638-7650.

Xu, P., Abdukader, A., Hu, K., Cheng, Y., and Zhu, C. (2014). Room temperature decarboxylative trifluoromethylation of α, β-unsaturated carboxylic acids by photoredox catalysis. Chem. Commun. *50*, 2308-2310.

Yang, J., Liu, S., Zheng, J. F., and Zhou, J. (2012). Room‐temperature Suzuki–Miyaura coupling of heteroaryl chlorides and tosylates. Eur. J. Org. Chem. *31*, 6248-6259.

Yang, X., Jin, X., and Wang, C. (2016). Manganese‐catalyzed ortho‐C─H alkenylation of aromatic n─h imidates with alkynes: versatile access to mono‐alkenylated aromatic nitriles. Adv. Synth. Catal. *358*, 2436-2442.

Yuen, O. Y., So, C. M., and Kwong, F. Y. (2016). Open-air oxidative Mizoroki-Heck reaction of arylsulfonyl hydrazides with alkenes. RSC. Adv. *6*, 27584-27589.

Zhang, J., and Tang, Y. (2016). Iron-catalyzed regioselective oxo-andhydroxy-phthalimidation of styrenes: access to α-hydroxyphthalimide ketones. Adv. Synth. Catal. *358*, 752-764.
